# Blind Flight? A New Troglobiotic Orthoclad (Diptera, Chironomidae) from the Lukina Jama – Trojama Cave in Croatia

**DOI:** 10.1371/journal.pone.0152884

**Published:** 2016-04-27

**Authors:** Trond Andersen, Viktor Baranov, Linn Katrine Hagenlund, Marija Ivković, Gunnar Mikalsen Kvifte, Martina Pavlek

**Affiliations:** 1 Department of Natural History, University Museum of Bergen, University of Bergen, Bergen, Norway; 2 Lebniz Institute for Freshwater Ecology and Inland Fisheries, Berlin, Germany; 3 Department of Zoology, Division of Biology, Faculty of Science, University of Zagreb, Zagreb, Croatia; 4 Department of Zoology, Institute of Biology, University of Kassel, Kassel, Germany; 5 Department of Molecular Biology, Ruđer Bošković Institute, Zagreb, Croatia; 6 Croatian Biospeleological Society, Zagreb, Croatia; Australian Museum, AUSTRALIA

## Abstract

The genus *Troglocladius* Andersen, Baranov *et* Hagenlund, gen. n. is erected based on *T*. *hajdi* Andersen, Baranov *et* Hagenlund, sp. n. collected at 980 m depth in the Lukina jama—Trojama cave system in Croatia. Morphological features such as pale color, strongly reduced eyes and very long legs make it a typical cave animal. Surprisingly, it has also retained large wings and appears to be capable of flight which would make *T*. *hajdi* the first flying troglobiont worldwide, disproving previous beliefs that bats are the only animals capable of flying in complete darkness. Morphologically the new species does not readily fit within any described genus, but shares characteristics with genera both in the tribes “Metriocnemini” and “Orthocladiini”. Bayesian molecular phylogenetic analysis using the markers COI, 18S rDNAs, 28S rDNA, CADI, and CADIV groups it with the genera *Tvetenia*, *Cardiocladius* and *Eukiefferiella* in the tribe “Metriocnemini”. *Troglocladius hajdi* may be parthenogenetic, as only females were collected. The discovery confirms the position of the Dinaric arch as a highly important hotspot of subterranean biodiversity.

## Introduction

Terrestrial species adapted to cave life are generally characterized as either troglophiles or troglobionts. While troglobionts are obligatory cave-dwellers, spending their entire life in the caves, troglophiles are essentially epigean species that are subdivided to eutroglophiles (able to maintain permanent subterranean populations) and subtroglophiles (dependent on epigean habitats for some biological functions). A third category of cave-dwelling organisms, the trogloxenes, are only accidentally found in caves [1]. Out of more than 21,000 terrestrial cave taxa worldwide [2] all troglobionts are ground dwellers. Bats fly in the complete darkness of caves using echolocation for navigating, they are however, troglophilic, and there are no known flying troglobionts.

The Dinarides or Dinaric Alps stretch along the western part of the Balkan Peninsula in southern Europe and are a part of the Alpine system along the east coast of the Adriatic Sea. According to the most recent census, the Dinaric karst harbors 600 terrestrial and 330 aquatic troglobionts [3]. Comparison of this number to the 1138 obligate cave taxa in United States [4] reveals the Dinarides as the world’s richest area of obligate subterranean fauna when compared to other regions of approximately the same size [4]. The extreme richness of the area can also be portrayed via other fauna, including the world’s sole troglobiotic representatives of Porifera, Cnidaria, Bivalvia and Polychaeta [5].

Although hundreds of different Diptera species have been recorded from caves, it has been stated that Diptera contains few true cave species, most are occasional guests or at most troglophiles (see e.g. [6, 7]). However, some Diptera have evolved morphological and physiological adaptations for a troglobiotic life [7]. These adaptations are possibly most pronounced in the African *Mormotomyia hirsuta* Austen, 1936 (Mormotomyiidae), which is associated with bats in caves and regarded as troglophilic as it sometimes is found outside of caves. It lacks eyes, wings and pigments and has developed elongate, spindly legs densely covered with sensory setae [8]. In the family Sphaeroceridae, several species and subspecies are found in caves and some of these, such as *Crumomyia absoloni* (Bezzi, 1914), which is found in caves in northeastern Herzegovina, are pale and have reduced eyes, long legs and short wings and halteres [9–10]. Some of these adaptations are also found in other taxa including *Allopnyxia patrizii* Freeman, 1952 (Sciaridae) from caves in Italy, the obligate bat parasitic families Nycteribiidae and Strebliidae, and several species in the families Culicidae, Psychodidae, Mycetophilidae, Keroplatidae, Phoridae, and Heleomyzidae [7, 11].

Chironomidae (Diptera) have been recorded from caves in many parts of the world (e.g. [12–17]). Most of these records are from the first few hundred meters from the cave entrance and, if identified beyond family level, the species recorded appear mostly to be trogloxenes. Reeves [18] categorizes *Zavrelimyia* nr *thryptica* (Sublette, 1964) from caves in the Great Smoky Mountains, U.S.A., as a troglophile, but until now there seems to be no records of any true troglobionts among the chironomids.

During expeditions to the Lukina jama—Trojama cave system in the Velebit Mountain Range in Croatia in 2013, several females of a pale chironomid belonging to the subfamily Orthocladiinae were collected in a chamber at 980 m below the surface. The female is described below and placed in a new genus based on morphological and molecular data. Furthermore, its habitat and natural history is outlined based on collection data and *in situ* observations.

### The Lukina Jama—Trojama cave system

The Lukina jama—Trojama cave system is situated in the Hajdučki and Rožanski kukovi Strict Reserve, Velebit National Park, a 109 km^2^ park in the northern parts of the Velebit Mountain in Croatia. There are several deep caves in the park with some of the world's largest subterranean vertical drops. The Lukina jama—Trojama cave system is 1.431 m deep and thus the deepest cave in southeast Europe and the 14^th^ deepest cave in the world.

**Fig 1:**
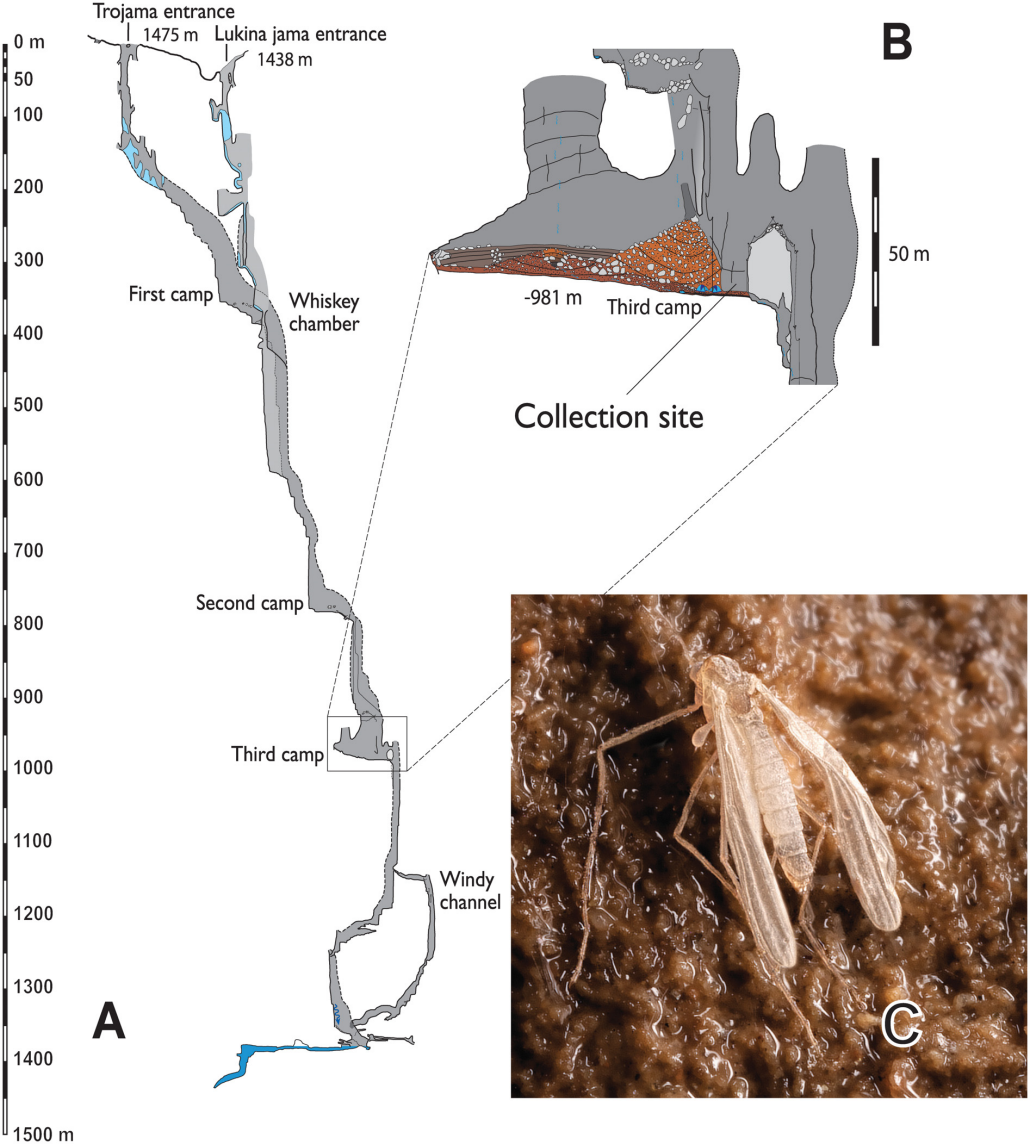
A-C.–A. SE-NW profile of the Lukina jama—Trojama cave system in the Velebit Mountain, Croatia. Explored by Speleological Committee of the Croatian Mountaineering Association, Croatian Speleological Federation and Slovak Speleological Society; map prepared by Darko Bakšić. Positions of the 3 campsites (First, second and third camp) inside the pit are marked on the map.–B. The chamber at 980 m depth, where most of the specimens were caught.–C. Newly emerged specimen of *Troglocladius hajdi* Andersen, Baranov *et* Hagenlund, gen. n., sp. n., sitting on the cave wall (photo J. Bedek).

The cave system has two vertical entrances at 1.438 and 1.475 m above sea-level (Fig 1A) and in winter snow falls into the pits making an icy layer that covers the walls and extends approximately to a depth of 350 m in Lukina jama and 200 m in Trojama; some years, the Lukina jama entrance is completely sealed off by an ice cap. Air temperature gradually sinks and becomes more stable during the first 200 m of the cave system, as influence from the outside decreases (at -180 m it is around -0.7°C). From there, the temperature is very stable year round and slowly rises again to 2.5°C in the middle reaches (around Second Camp, Fig 1), in the chamber at 980 m depth it reaches 3.5°C, and at the bottom of the pit the temperature is around 4.9°C [19].

The first 200 m of the cave system has substantial presence of ice and snow year round. Water and a strong draught of cold air constantly flow down the shaft. These conditions, along with the cold temperatures make this part of the cave system unsuitable for subterranean fauna.

The new species has only been observed at depths between 800 and 980 m. Though it cannot completely be ruled out that the species is present in the upper parts of the Lukina jama—Trojama cave system, it is highly unlikely given the inhospitable conditions. The chamber at 980 m depth (Fig 1B—third camp) is about 85 x 70 meters in size and contains several habitats including dry walls, hygropetric environments with water seeping on the walls, small streams with sediment and a small pond with standing water—potential habitats for the immature stages.

A total of 54 animal taxa have been recorded from the Lukina jama—Trojama cave system, of which 32 are true cave dwellers [20]. Among these, the cave system houses the endemic snail *Zospeum tholussum* Weigand, 2013 and one of the largest known colonies of the subterranean leech *Croatobranchus mestrovi* Kerovec, Kučinić *et* Jalžić, 1999 [21–22].

## Material and Methods

### Collection of specimens

Permission for collecting cave fauna was issued by The Ministry of Environmental and Nature Protection, Croatia. Altogether 16 females have been collected in the Lukina jama—Trojama cave system. During an expedition between 29^th^ July and 3^rd^ August in 2010 one female was found on the wet wall of the cave at about 800 m depth. During a second expedition between 1^th^ and 3^rd^ August 2011, two females were collected on the wall in the chamber at 980 m depth (Fig 1C). During a third expedition between 8^th^ and 12^th^ August in 2013 altogether 13 females were collected using sticky traps placed directly on the walls of the cave. The specimens were preserved in alcohol and later mounted in Canada Balsam following the procedure outlined by Sæther [23].

### Morphological description

The general morphology follows Sæther [24]. The measurements are given as ranges, followed by the mean when four or more specimens were measured, followed by the number of specimens measured in parentheses.

### DNA extraction, PCR amplification and sequencing

The thorax and part of the abdomen of five specimens were kept in alcohol and used for DNA extraction. The DNA was extracted non-invasively using the QIAGEN DNeasy^®^ Blood and Tissue kit and amplified with PCR following protocols as specified in S1 Text and S1 Table. The markers 18S rDNA, 28S rDNA and COI were amplified using primers as specified in Cranston *et al*. [25]; GenBank accession numbers and voucher collection numbers for the two successfully sequenced specimens are listed in the S2 Table. Two further markers, CADI and CADIV, failed to amplify in our study, but were nevertheless included in the final analysis for topological stability with respect to Cranston *et al*. [25].

The gene regions were aligned in Mega v6.0.6. [26] using a combination of MUSCLE [27] and manual alignment. Ambiguous regions were excluded in GBlocks v0.91b [28–29], with all the options set for a less stringent selection. This method was sufficient for 28S rDNA; however, for 18S rDNA the amount of hypervariable regions made it necessary to align manually according to secondary structure. The alignment and reference sequence are available in S2 and S3 Texts.

### Molecular phylogenetic analysis

The sequences were compared with all Orthocladiinae sequences from Cranston *et al*. [25] using Diamesinae and Prodiamesinae as outgroups. The final data set consists of 3.709 characters, 1.560 of which are parsimony informative, in a matrix of 67 taxa. Analyses were carried out in PAUP*, MEGA and mrBayes v3.2.3, using Bayesian methods [26, 30–31]. Models were selected and tested using JModeltest [32] in combination with PAUP*. Bayesian analysis was performed using 10 million generations, 4 MCMC chain reactions and the model GTR+G+I as selected by JModeltest for all genes.

### Type material

The holotype and 3 paratypes are deposited in the Croatian Biospeleological Society (CBSS) collection, which is a part of Croatian Natural History Museum collection in Zagreb, Croatia. Three paratypes, along with DNA extracts are deposited in the University Museum of Bergen, Bergen, Norway (ZMBN) and an additional paratype is deposited in the Zoologische Staatssammlung München, Munich, Germany (ZSM).

### Nomenclatural acts

The electronic edition of this article conforms to the requirements of the amended International Code of Zoological Nomenclature, and hence the new names contained herein are available under that Code from the electronic edition of this article. This published work and the nomenclatural acts it contains have been registered in ZooBank, the online registration system for the ICZN. The ZooBank LSIDs (Life Science Identifiers) can be resolved and the associated information viewed through any standard web browser by appending the LSID to the prefix "http://zoobank.org/". The LSID for this publication is: urn:lsid:zoobank.org:pub:5CA9C540-440A-4439-8961-4D7BB2FBB781. The electronic edition of this work was published in a journal with an ISSN, and has been archived and is available from the following digital repositories: PubMed Central, LOCKSS, and Cristin (www.cristin.no).

## Results

### *Troglocladius* Andersen, Baranov *et* Hagenlund, gen. n.

urn:lsid:zoobank.org:act:96A93798-6392-482D-A07D-993D2BBF1776

*Type species*. *Troglocladius hajdi* Andersen, Baranov *et* Hagenlund, sp. nov. urn:lsid:zoobank.org:pub:5CA9C540-440A-4439-8961-4D7BB2FBB781

*Etymology*. From the Greek *trogle*, hole, and -*cladius*, a common ending of related Orthocladiinae genera. The gender of the name is masculine.

*Diagnosis*. The pale color in combination with hairy eyes without, or with only a few ommatidia, reduced palp with palpomeres 2+3 and 4+5 most often fused, very long legs, and large wings and halteres will separate the genus from all other Orthocladiinae genera.

#### Description

*Female*. Medium sized species, wing length 1.8–2.2 mm.

*Antenna*. Female antenna with 6 flagellomeres, antennal ratio about 0.41. Flagellomere 4 with 1 seta; flagellomere 5 with 2 setae, longest about 5 times as long as flagellomere; flagellomere 6 with 1 apical seta, about twice as long as flagellomere. Sensilla chaetica present on flagellomeres 1–5, sensilla on flagellomeres 2–4 about 1.5 times as long as flagellomere.

*Head*. Eye reniform, strongly hairy, without or with few reduced ommatidia. Temporal setae in single row consisting of inner and outer verticals, postorbitals present or absent. Clypeus with few setae. Frontal tubercle absent. Tentorium and stipes normal. Cibarial pump with anterior margin straight. Palp short, palpomeres 2+3 and 4+5 fused in most specimens; third palpomere with few sensilla clavata apically.

*Thorax*. Antepronotum with lobes meeting medially at anterior margin of scutum, with few lateral antepronotals. Acrostichals short, starting some distance from antepronotum; dorsocentrals absent or few; prealars few; supraalar absent. Scutellum with few setae in single row. Haltere large.

*Wing*. Broad with excavated anal lobe. Membrane without setae, with fine punctation. Costa moderately extended. R_3+4_ ending slightly closer to R_1_ than to R_4+5_; R_4+5_ ending opposite to M_3+4_; FCu about opposite to RM; Cu_1_ weakly sigmoid. Brachiolum with 1 seta, R, R_1_ and R_4+5_ with few setae, other veins bare. Squama with few setae. Sensilla campaniformia about 3–6 basally, 5–6 apically, and 2 above seta on brachiolum and 1 basally on R_1_.

*Legs*. All legs very long. All tibia with only one spur, hind tibial comb normal. Tarsal pseudospurs and sensilla chaetica absent. Pulvilli vestigial.

*Abdomen*. Tergites and sternites with few setae in 3–4 rows.

*Genitalia*. With well-developed gonocoxite with several setae. Tergite IX divided into 2 setigerous protrusions. Segment X normal. Cerci comparatively large. Postgenital plate with slightly emarginated posterior margin. Coxosternapodeme curved. Gonapophysis VIII divided into large, triangular ventrolateral lobe and narrow dorsomesal lobe. Apodeme lobe relatively conspicuous. Seminal capsules pale, large, ovoid, with triangular neck. Spermathecal ducts with loops and common opening; ducts apparently without microtrichia. Labia without microtrichia.

#### Systematics

The species is difficult to place systematically based on morphological characters alone, partly because only the female is known, and partly because it shows several morphological adaptations to cave life. Using the preliminary key to the females of Orthocladiinae in Sæther [33] the species will key to *Cricotopus* van der Wulp, 1874 if the dorsocentrals are regarded as short and decumbent or to *Paratrichocladius* Santos Abreu, 1918 if the dorsocentrals are regarded as long and erect. It clearly has a divided gonapophysis VIII with a large ventrolateral lobe covering most of the dorsomesal lobe, as can be found in the *Cricotopus* group of genera. Further, tergite IX is divided in two setigerous protrusions, which also can be found in the *Cricotopus* group.

**Fig 2:**
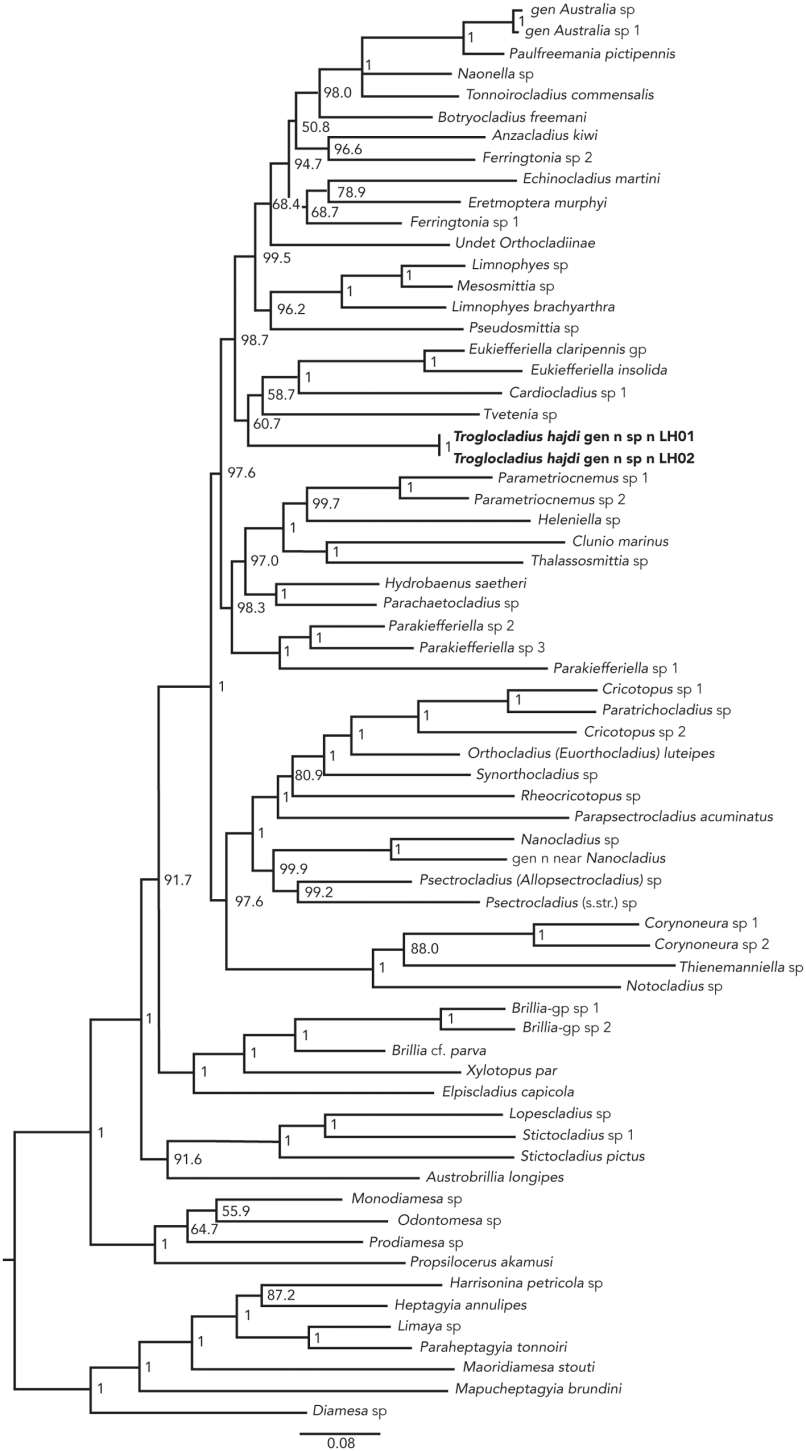
Bayesian tree of Orthocladiinae showing the position of *Troglocladius hajdi* Andersen, Baranov *et* Hagenlund, gen. n., sp. n., within the tribe “Metriocnemini”. Posterior probability (PP) is given in percent for the branches.

The phylogenetic analysis based on DNA sequences yielded a tree which overall is very similar to that given for Orthocladiinae by Cranston *et al*. [25]. The two specimens from the Lukina jama—Trojama cave grouped together and were well separated from all other genera in the analysis. They did not however, group with *Cricotopus* in tribe “Orthocladiini”, but fell within tribe “Metriocnemini” together with the genera *Tvetenia* Kieffer, *Cardiocladius* Kieffer and *Eukiefferiella* Thienemann (Fig 2). Posterior probabilities for the other higher nodes within this clade were all significant (>98%), thus excluding the specimens from these sub-clades with high confidence. Posterior probability for the node within tribe “Metriocnemini” containing the new species, together with *Tvetenia*, *Cardiocladius* and *Eukiefferiella* was however, only 61% possibly due to missing data on CADI and CADIV. The mutual arrangement of the genera within this node is thus uncertain. One should also note that many Orthocladiinae genera remain to be sequenced and the internal relationships within tribe “Metriocnemini” may change.

Morphologically the new species does not group with the genera *Tvetenia*, *Cardiocladius* and *Eukiefferiella*, as all three have an undivided gonapophysis VIII [33] while the new species clearly has a divided gonapophysis VIII. A divided gonapophysis VIII with a large ventrolateral lobe covering most of the dorsomesal lobe and a divided tergite IX are however, traits found in many Orthocladiinae, including both the *Cricotopus* group and several genera within “Metriocnemini”. Based on our current knowledge, the new species apparently does not fit into any described genus, thus supporting our placement of the species in a new genus within tribe “Metriocnemini”.

### *Troglocladius hajdi* Andersen, Baranov *et* Hagenlund, sp. n. (Figs 3–6)

**Fig 3:**
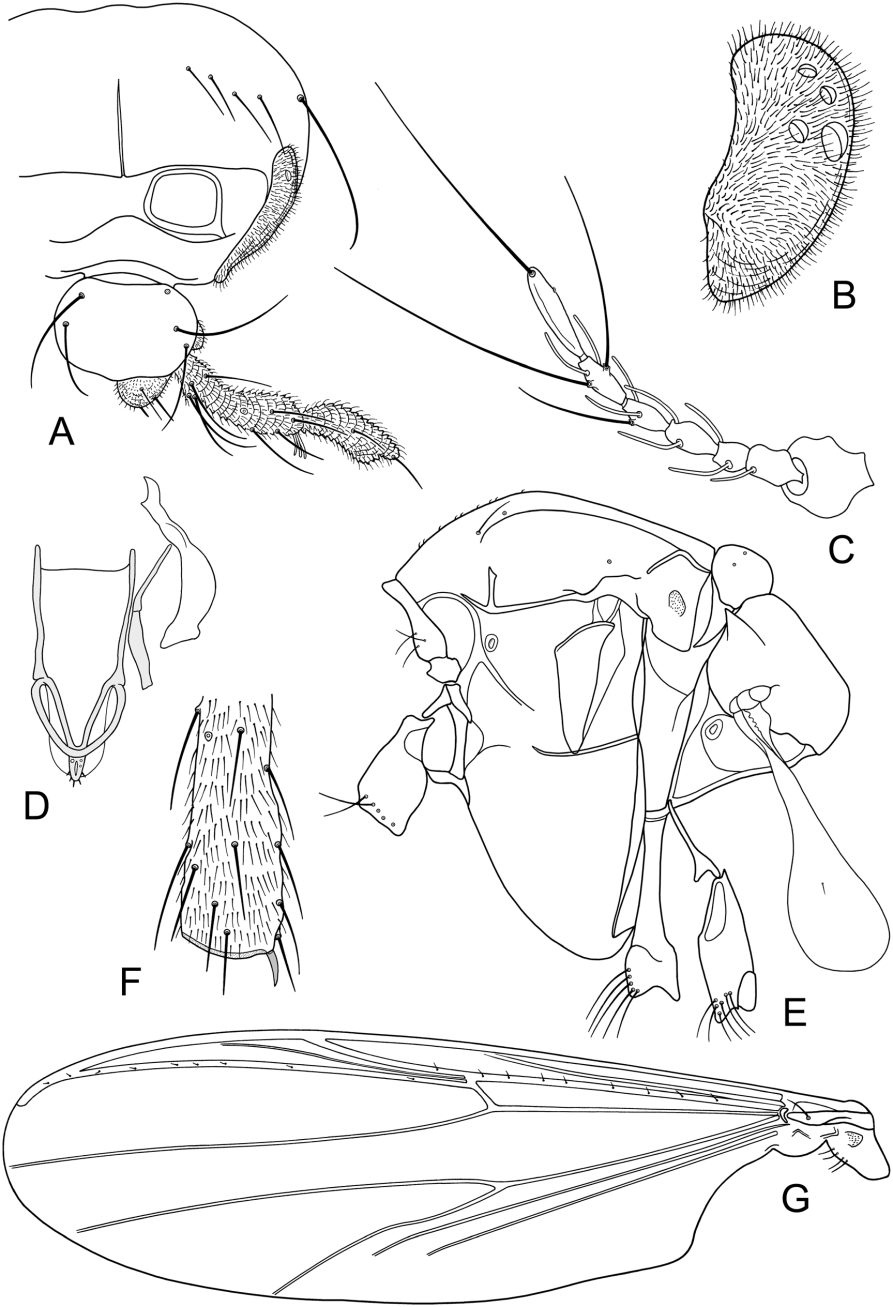
A-G. *Troglocladius hajdi* Andersen, Baranov *et* Hagenlund, gen. n., sp. n., female.–A. Head.–B. Eye.–C. Antenna.–D. Tentorium, stipes and cibarial pump.–E. Thorax.–F. Spur of mid leg.–G. Wing.

**Fig 4:**
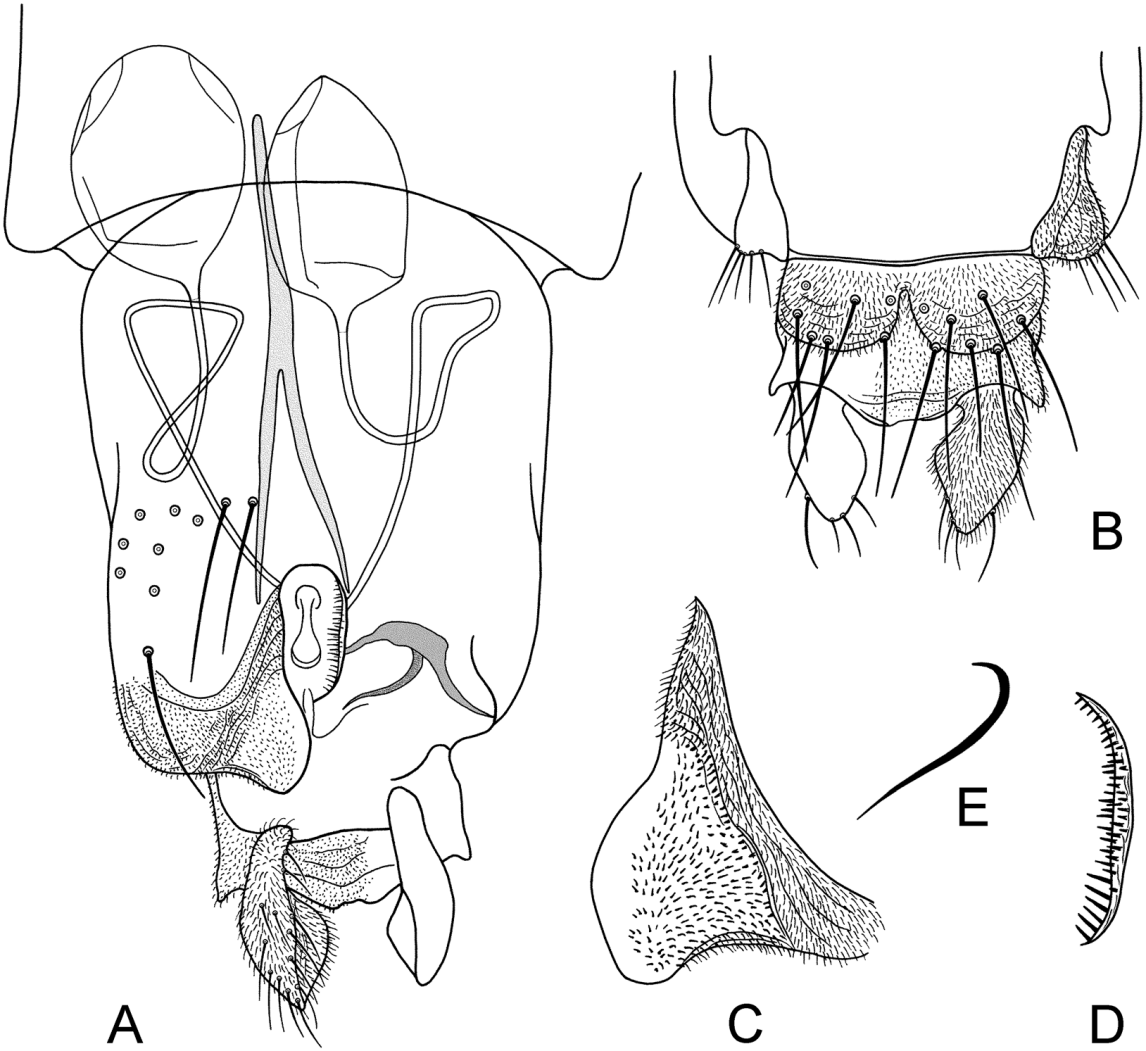
A-E. *Troglocladius hajdi* Andersen, Baranov *et* Hagenlund, gen. n., sp. n., female.–A. Genitalia, ventral view.–B. Tergite IX.–C. Ventrolateral lobe.–D. Dorsomesal lobe.–E. Apodeme lobe.

**Fig 5:**
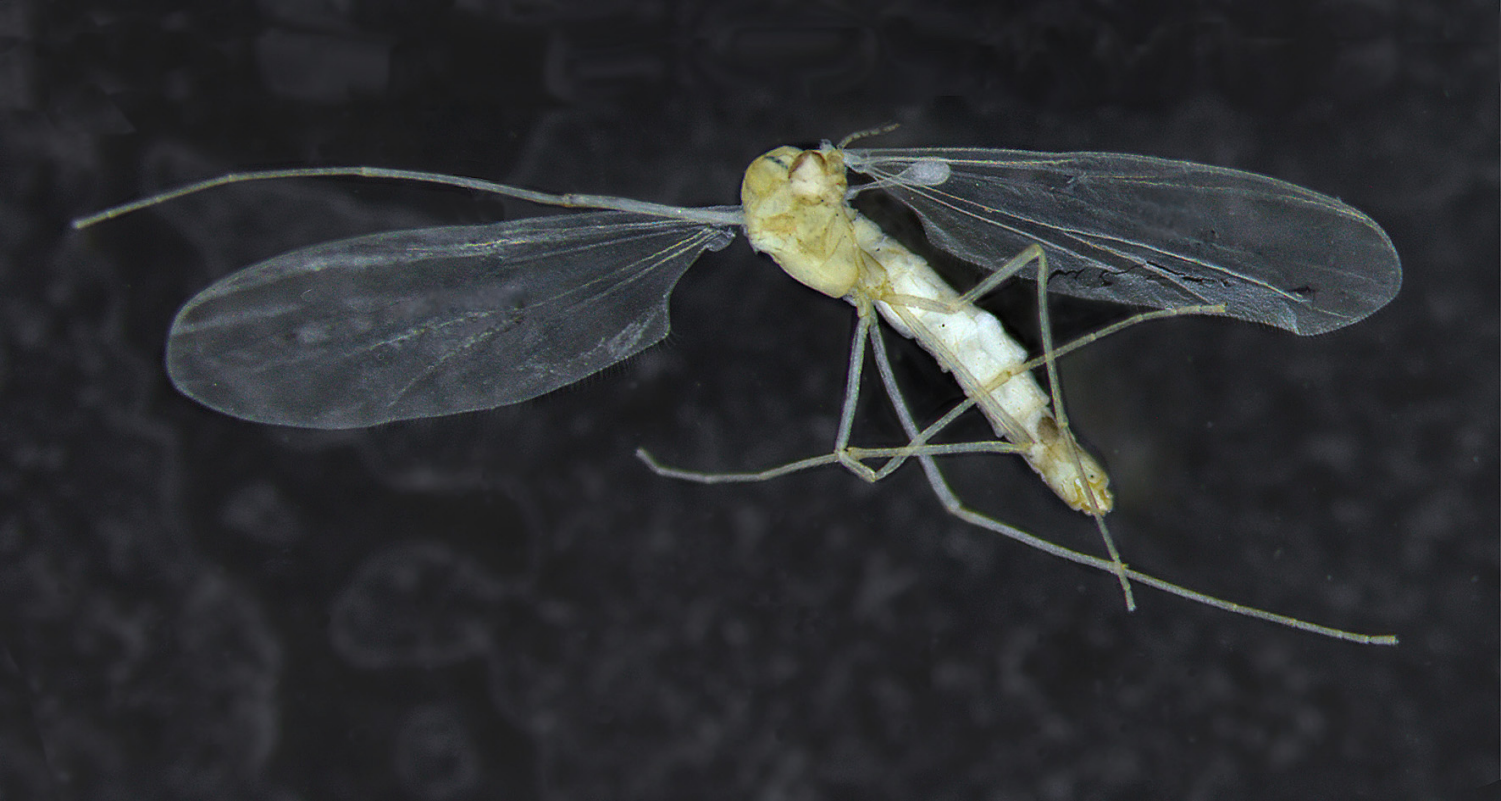
*Troglocladius hajdi* Andersen, Baranov *et* Hagenlund, gen. n., sp. n., female. Photo showing the pale colour and the broad wings.

**Fig 6:**
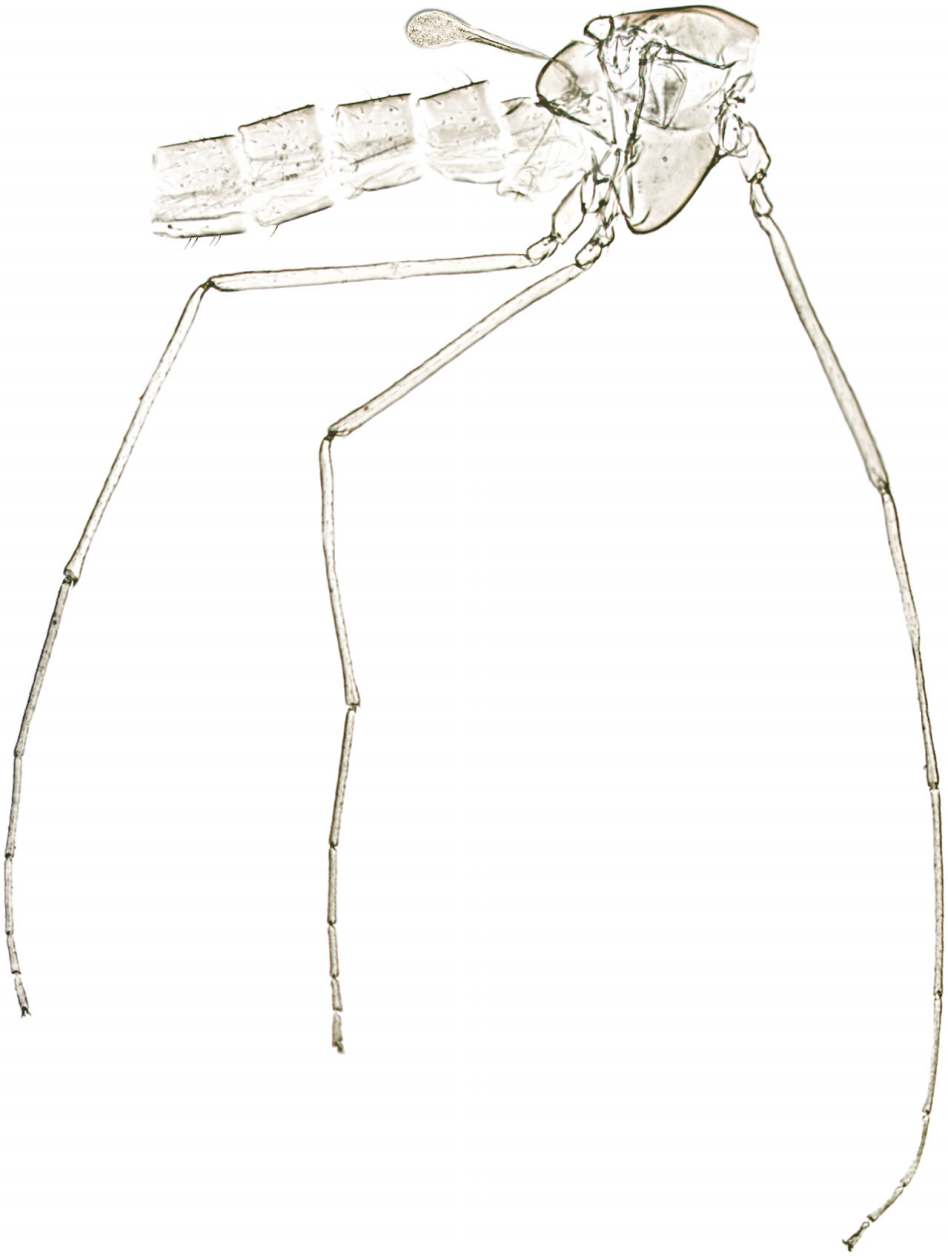
*Troglocladius hajdi* Andersen, Baranov *et* Hagenlund, gen. n., sp. n., female. Photo of slide mounted thorax, showing the long legs and the comparatively large haltere.

*Holotype*, f, CROATIA, Velebit Mountain, Lukina jama—Trojama cave system, N 44.766939, E 15.026571, 980 m depth, 3 August 2010, leg. M. Lukić (CBSS).

*Paratypes*. 2 ff as holotype except 1–3 August 2011, leg. J. Bedek; 4 ff as holotype except 9 August 2013, leg. M. Pavlek; 1 f as holotype except 10 August 2013, leg. M. Pavlek (CBSS, ZMBN, ZSM).

*Etymology*. Named after the Hajdi, a group of winged, dwarf-like creatures from Slavic mythology, where they acted as messengers of fate and were said to dwell in caves. The name is to be regarded as a noun in apposition.

*Diagnosis*. See generic diagnosis.

#### Description

*Female* (n = 4–7, if not otherwise stated). Total length 1.80 (1) mm. Wing length 1.79–2.17, 2.01 mm. Total length / wing length 0.98 (1). Wing length / length of profemur 2.72–2.86, 2.77.

*Coloration*. Pale yellowish.

*Antenna* (Fig 3C). Antennal ratio (AR) 0.39–0.45, 0.41. Pedicel 41–55, 49 μm long; 48–61, 55 μm wide. Flagellomeres 1–6 length / width (in μm): 39–47, 43 / 23–28, 26; 23–28, 25 / 19–21, 20; 29–36, 33 / 18–19, 18; 25–36, 31 / 14–18, 16; 36–43, 40 / 15–19, 17; 63–73, 70 / 14–18, 15. Apical seta 117–144, 128 μm long.

*Head* (Fig 3A). Temporal setae 3–7, 5 including 2–4, 3 inner verticals, 1–2, 1 outer vertical and 0–2, 1 postorbital. Eye (Fig 3B) with 0–4, 2 ommatidia. Clypeus with 4–5, 5 setae. Tentorium, stipes and cibarial pump as in Fig 3D. Tentorium 63–76 (3) μm long, 12–17 (3) μm wide. Stipes 58 (1) μm long. Palpomeres 1, 2+3, and 4+5 lengths (in μm): 11–19, 15; 44–97, 73; 30–79, 54. In one specimen palpomere 4 and 5 distinctly separated, fourth palpomere 37 μm long, fifth palpomere 46 μm long. Third palpomere with 2–3 sensilla clavata apically, longest 20–22 μm long.

*Thorax* (Fig 3E). Antepronotum with 3–5, 3 ventrolateral setae. Acrostichals 9–12, 11; dorsocentrals 0–2, 1; prealars 1–2, 1; supraalars absent. Scutellum with 4–6, 5 setae. Haltere 344–396, 368 μm long.

*Wing* (Fig 3G). Venarum ratio (VR) 1.01–1.02, 1.01. Costal extension 121–133, 126 μm long. R with 6–9, 8 setae; R_1_ with 1–4, 2 setae; R_4+5_ with 6–7, 6 setae, costal extension with 4–7, 6 non-marginal setae; brachiolum with 1 seta. Squama with 3–4, 4 setae.

*Legs*. Spur of fore tibia 21–26, 23 μm long; spur of mid tibia (Fig 3F) 15–17, 16 μm long; spur of hind tibia 33–43, 38 μm long. Width at apex of fore tibia 33–40, 37 μm; of mid tibia 36–39, 37 μm; of hind tibia 39–48, 45 μm. Comb composed of 13–14, 13 setae; longest 34–45, 37 μm long; shortest 23–28, 25 μm long. Length and proportions of legs as in Table 1.

**Table 1:** Lengths (in μm) and proportions of legs of *Troglocladius hajdi* Andersen, Baranov *et* Hagenlund, gen. nov., sp. nov., female (n = 4–5). Morphological abbreviations follow Sæther [24].

	Fe	ti	ta_1_	ta_2_
p_1_	711–776, 745	703–801,758	425–531,493	253–302, 278
p_2_	719–801, 754	670–703, 688	310–359, 343	180–196, 190
p_3_	792–850, 819	735–833, 810	392–474, 441	229–253, 243
	ta_3_	ta_4_	ta_5_	LR
p_1_	172–196,186	106–123, 114	65–74,70	0.61–0.67, 0.65
p_2_	114–131, 127	74–90, 82	49–65, 59	0.45–0.52, 0.50
p_3_	155–188, 178	90–98, 95	57–65, 62	0.53–0.57, 0.54
	BV	SV	BR	
p_1_	3,01–3.23, 3.09	2.95–3.33, 3.06	1.46–1.50, 1,49	
p_2_	3.66–4.12, 3.91	4.09–4.53, 4.21	1.07–1.47, 1.30	
p_3_	3.54–3.64, 3.58	3.53–3.90, 3.70	1.12–1.71, 1.40	

*Genitalia* (Fig 4A–4E). Sternite VIII with 8–11, 9 setae to each side. Gonocoxite IX with 4–6, 5 moderately strong setae. Tergite IX divided, with 14–16, 15 setae in two distinct groups. Cercus 98–103 (2) μm long. Seminal capsule ovoid; 83–105, 94 μm long; 66–71, 68 μm wide; with 10–14, 11 μm long; 12–16, 14 μm wide neck. Notum 107–117, 113 μm long. Ventrolateral lobe subtriangular; 69–76, 73 μm long; 39–48, 43 μm wide at its widest point; covered with microtrichia. Dorsomesal lobe 54–57, 56 μm long from gonocoxapodeme to apex.

*Male and immature stages*. Unknown.

## Discussion

True troglobionts are generally characterized by several morphological features like lack of integumentary pigment; most species are whitish or even translucent. Further, since there is no light in the caves, troglobionts often show some degree of eye reduction. They often have elongated legs and antennae; in cave environments long legs and appendages are clearly an advantage and are often coupled with a well-developed sensory apparatus. Most cave insects also lack wings or the wings are reduced [34].

The new species described here shares several of these adaptations. The body is pale yellowish (Fig 5) and weakly sclerotized. The eyes are strongly reduced with only 0–4 ommatidia present. The legs are comparatively very long (Fig 6). The antenna appears short, but have few, long setae on segments 4–6 and strong sensilla on segments 1–5. Remarkably however, the wings are well developed, long and broad with an excavated anal lobe, and the halteres are also unusually large.

The combination of strongly reduced eyes and large, broad wings appears to be unique among troglobiotic organisms and might indicate that the species is able to fly slowly or hover in the total darkness of the cave. The long forelegs might serve as “feelers” if they are stretched forward during flight and the large halteres might help the insect maintain balance. During the 2013 expedition *T*. *hajdi* was never observed flying, but some of the specimens collected in the sticky traps were sitting in the middle of the strips, suggesting that they fly at least occasionally. During future expeditions, efforts should thus be made to observe the species’ behavior in the cave.

Of all free-living troglophilic or troglobiotic Diptera recorded so far, *T*. *hajdi* has by far the most troglomorphic characteristics. Furthermore, while other taxa are found close to the entrance or in comparatively small caves, *T*. *hajdi* inhabits a deep and highly isolated part of a large cave system with very little possibility to communicate with the outside environment. The discovery of the most troglomorphic winged Diptera described so far provides further support for the Dinaric karst as an area of extreme subterranean biodiversity.

The new species might well be parthenogenetic as only females were collected. In insects, parthenogenesis is most often found in harsh environments, where mating can be difficult. Parthenogenesis is not uncommon among chironomids and is most often found in extreme or isolated habitats [35]. Examples include Antarctic midges like *Eretmoptera murphyi* Schaeffer, 1914 or isolated island populations like populations of *Limnophyes minimus* (Meigen, 1818) on Gough and Nightingale Islands [36–37]. Parthenogenesis can also occur in habitat specialists restricted to fragmented habitats such as *Polypedilum parthenogeneticum* Donato *et* Paggi, 2008 living in water held in the leaf axils of *Eryngium pandanifolium* Chamb *et* Schlecht (Apiaceae) [38]. Although too few specimens of *T*. *hajdi* have been collected to confidently rule out the existence of males, we regard parthenogenesis as the most likely reproductive system considering the species’ isolated habitat.

The larvae and pupae of *T*. *hajdi* are not known. During the expeditions in 2013 several larvae of the mycetophilid *Speolepta leptogaster* Madwar, 1937 were collected on the walls in the chamber at 980 m depth and wrongly thought to represent the larvae of *T*. *hajdi*. However, in the 980 m chamber there are several shallow streams with fine sediment, which were not sampled during the 2013 expedition and the larvae of *T*. *hajdi* might inhabit these streams. During coming expeditions to the Lukina jama—Trojama cave system or to other deep caves in that area, the immatures of *T*. *hajdi* should therefore be searched for. However, if endemic to the Lukina jama—Trojama cave system, great care should be taken not to collect too many specimens, as the populations might be very small and thus vulnerable.

## Supporting Information

S1 Table Master mix for PCR and PCR thermocycling protocols for the genetic markers used in the analysis. (DOCX)

S2 Table GenBank accession numbers for vouchers LH01 and LH02 of *Troglocladius hajdi* Andersen, Baranov *et* Hagenlund, gen. nov., sp. nov. (DOCX)

S1 Text DNA extraction protocol for the specimens of *Troglocladius hajdi* Andersen, Baranov *et* Hagenlund, gen. nov., sp. nov. (DOCX)

S2 Text Concatenated alignment for all the molecular markers used in the analysis. (DOCX)

S3 Text Reference sequence with secondary structure annotation for the alignment of 18S rDNA. (DOCX)
