# Supplementary material for: Blind Flight? A New Troglobiotic Orthoclad (Diptera, Chironomidae) from the Lukina Jama – Trojama Cave in Croatia
Source: PLoS One. 2016 Apr 27;11(4):e0152884. doi: 10.1371/journal.pone.0152884 (PMC4847865; doi:10.1371/journal.pone.0152884)
Supplement: S1 Table — (DOCX) [file pone.0152884.s001.docx]

**S1 table – Master mix for PCR and PCR thermocycling protocols for the genetic markers used in the analysis.**

**Master mix for COI and CAD:**

| **Reagent** | **Volume per reaction** |
| --- | --- |
| ddH2O | 16.35 µL |
| 10X reaction buffer | 2.5 µL |
| dNTPs (10 mM) | 2.0 µL |
| Forward primer (100 µM) | 1.0 µL |
| Reverse primer (100 µM) | 1.0 µL |
| TaKaRa Ex-Taq® | 0.15 µL |
| Template DNA | 2.0 µL |
| Total volume | 25 µL |

**Master mix for 18s & 28s:**

| **Reagent** | **Volume per reaction** |
| --- | --- |
| ddH2O | 15.35 µL |
| 10X reaction buffer | 2.5 µL |
| dNTPs (10 mM) | 2.0 µL |
| Forward primer (100 µM) | 1.0 µL |
| Reverse primer (100 µM) | 1.0 µL |
| TaKaRa Ex-Taq® | 0.15 µL |
| Q-solution | 1.0 µL |
| Template DNA | 2.0 µL |
| Total volume | 25 µL |

**Thermocycling program for COI:**

| 94°C | 4 min |  |
| --- | --- | --- |
| 94°C | 45 sec |  |
| 45°C | 30 sec | x 40 cycles |
| 72°C | 1 min |  |
| 72°C | 10 min |  |
| cool (6°C) |  |  |

**Thermocycling program for 18s and 28s:**

| 94°C | 4 min |  |
| --- | --- | --- |
| 95°C | 30 s |  |
| 57°C | 30 s | x 10 cycles |
| 72°C | 1 min |  |
| 95°C | 30 s |  |
| 47°C | 30s | x 30 cycles |
| 72°C | 1 min |  |
| 72°C | 10 min |  |
| cool (6°C) |  |  |

**Thermocycling program for CADI and CADIV*:**

| 94°C | 4 min |  |
| --- | --- | --- |
| 94°C | 30 s |  |
| 52°C | 30 s | x 5 cycles |
| 68°C | 1 min |  |
| 94°C | 30 s |  |
| 51°C | 1 min | x 7 cycles |
| 68°C | 1 min |  |
| 94°C | 30 s |  |
| 45°C | 20 s | x 36 cycles |
| 68°C | 1 min 30 s |  |
| 68°C | 10 min |  |

*Several different protocols were run for CAD, but none yielded results for the specimens from Lukina jama-Trojama.

**Sequencing reactions:**

| **Reagent** | **Volume per reaction** |
| --- | --- |
| Sequencing buffer | 1.0 µL |
| Primer (3.2 mmol) | 1.0 µL |
| DNA-template | according to calculation |
| H2O | up to 10 µL |

**Sequencing program:**

| 96 °C | 5 min |  |
| --- | --- | --- |
| 96°C | 10 sec |  |
| 50°C | 5 sec | x 24 cycles |
| 60°C | 4 min |  |
| cool (4°C) |  |  |
