# Supplementary material for: Blind Flight? A New Troglobiotic Orthoclad (Diptera, Chironomidae) from the Lukina Jama – Trojama Cave in Croatia
Source: PLoS One. 2016 Apr 27;11(4):e0152884. doi: 10.1371/journal.pone.0152884 (PMC4847865; doi:10.1371/journal.pone.0152884)
Supplement: S2 Table — (DOCX) [file pone.0152884.s002.docx]

**S2 table. GenBank accession numbers for vouchers LH01 and LH02 of** ***Troglocladius hajdi* Andersen, Baranov *et* Hagenlund, gen. nov., sp. nov.**

| Gene fragment | COI | 18s | 28s |
| --- | --- | --- | --- |
| LH01 | KT013220 | KT158647 | KT158648 |
| LH02 | KT013221 |  |  |
|  |  |  |  |
