# Supplementary material for: Blind Flight? A New Troglobiotic Orthoclad (Diptera, Chironomidae) from the Lukina Jama – Trojama Cave in Croatia
Source: PLoS One. 2016 Apr 27;11(4):e0152884. doi: 10.1371/journal.pone.0152884 (PMC4847865; doi:10.1371/journal.pone.0152884)
Supplement: S1 Text — (DOCX) [file pone.0152884.s003.docx]

**S3 text - DNA extraction protocol for the specimens of *Troglocladius hajdi* Andersen, Baranov *et* Hagenlund, gen. nov., sp. nov.**

DNA was extracted using the Qiagen DNeasy® Blood and Tissue kit according to the manufacturers instructions. Modifications to the protocol were as follows: Tissue was digested with proteinase K overnight. Elution was performed twice, once with 100 µl and the second step with 80 µl elution buffer AE. The AE buffer was warmed to 60° C and left on the membrane of the Spin Column for 10 minutes.
