# Supplementary material for: Blind Flight? A New Troglobiotic Orthoclad (Diptera, Chironomidae) from the Lukina Jama – Trojama Cave in Croatia
Source: PLoS One. 2016 Apr 27;11(4):e0152884. doi: 10.1371/journal.pone.0152884 (PMC4847865; doi:10.1371/journal.pone.0152884)
Supplement: S2 Text — (DOCX) [file pone.0152884.s004.docx]

**S4 text – Concatenated alignment for all the molecular markers used in the analysis.**

**Anzacladius kiwi** NNNNNNNNNNNNNNNNNNNNNNNNNNNNNNNNNNNNNNNNNNNNNNNNNNNNNNNNNNNNNNNNNNNNNNNNNNNNNNNNNNNNNNNNNNNNNNNNNNNNNNNNNNNNNNNNNNNNNNNNNNNNNNNNNNNNNNNNNNNNNNNNNNNNNNNNNNNNNNNNNNNNNNNNNNNNNNNNNNNNNNNNNNNNNNNNNNNNNNNNNNNNNNNNNNNNNNNNNNNNNNNNNNNNNNNNNNNNNNNNNNNNNNNNNNNNNNNNNNNNNNNNNNNNNNNNNNNNNNNNNNNNNNNNNNNNNNNNNNNNNNNNNNNNNNNNNNNNNNNNNNNNNNNNNNNNNNNNNNNNNNNNNNNNNNNNNNNNNNNNNNNNNNNNNNNNNNNNNNNNNNNNNNNNNNNNNNNNNNNNNNNNNNNNNNNNNNNNNNNNNNNNNNNNNNNNNNNNNNNNNNNNNNNNNNNNNNNNNNNNNNNNNNNNNNNNNNNNNNNNNNNNNNNNNNNNNNNNNNNNNNNNNNNNNNNNNNNNNNNNNNNNNNNNNNNNNNNNNNNNNNNNNNNNNNNNNNNNNNNNNNNNNNNNNNNNNNNNNNNNNNNNNNNNNNNNNNNNNNNNNNNNNNNNNNNNNNNNNNNNNNNNNNNNNNNNNNNNNNNNNNNNNNNNNNNNNNNNNNNNNNNNNNNNNNNNNNNNNNNNNNNNNNNNNNNNNNNNNNNNNNNNNNNNNNNNNNNNNNNNNNNNNNNNNNNNNNNNNNNNNNNNNNNNNNNNNNNNNNNNNNNNNNNNNNNNNNNNNNNNNNNNNNNNNNNNNNNNNNNNNNNNNNNNNNNNNNNNNNNNNNNNNNNNNNNNNNNNNNNNNNNNNNNNNNNNNNNNNNNNNNNNNNNNNNNNNNNNNNNNNNNNNNNNNNNNNNNNNNNNNNNNNNNNNNNNNNNNNNNNNNNNNNNNNNNNNNNNNNNNNNNNNNNNNNNNNNNNNNNNNNTTCAATATTCTTTTAAACGCAATGTGCACTTCTTTCTTAGTAAGACGCTGTGATCCATTAGAAATTCATTCCAATGATTATTTGGCTATCAATGGGCCGGTATGAAGTTCTTCAGCAAATAACTTAATCTTTTACGGTTGGGTATATTGTTTGTTGAGCGTACCGTAACCTGCATTATAGCTGTCTGATGGATTTTAAAGGTCATATTATTGCATCACTCATGATAACCCGATCAACAGCGGTTAAATTTGGGATACCTTTTGGACCCGTCTTGAAACACGGACCAAGGAGTCTATCTAGTATGCAAGTAAATGAGGAAACTTATTTGCAGAAAAAACTTGACTGATGGGACTACGAGTGCTCCTCAATCCCGGGGTATTTTCATCAACATATGCAAATGTATATGGAAAATATACCATGAGCATACTGGATATGACCCGAAAGATGGTGAACTATGCCTGATCAGGTTGAAGTCAGGGGAAACCCTGATGGAGGACCGAAGCAATTCTGACGTGCAAATCGCTGACTTACCCACTGATTGGAAATTACGGAATTCCGAGCGATGAGGAATTCGACGAGCATAAATTGATGAAACATTTCGAGTCGAACAATAAGATTTGGGTGTCGGGTTTGGTTGTCGGTGAGTTGTGTGAAACTCCATCGCACTGGAGACAGAAATACAAGCTAGCYGAGTGGATGGTCAAACAYAACGTGGTMGGAATCAGCGGAATTGACACACGMGCTTTGACGAAGAAAATTCGYGAGAACGGAACGGTTTTGGGAAAAATAATTCATCAATCGAAA------GGCCCATTTCCGGGG---TTGGAGTTTAAAGATCAAAATGCAAGGAATCTCGTCGACGAAGTTTCTATCAAAAAGCCAATTACGTACAACGCAAWWGGCTCTCCGAGAATTTGTGCCATCGATTGCGGATTGAAATTGAATCAAATTCGCTGTTTTATTCAACGTGGAGCTCGCGTTGATCTYGTGCCATGGGATTATCCCCTCAATGCAAAYGACTTCGACGGGTTGTTTCTAAGCAATGGTCCCGGCGATCCCGTCATGTGCAAGAAAACTGTTCAAAACATTCAAAATTATCTGAAATCGCAGAAAGTTAAGCCGGTTTTTGGTATCTGTTTGGGCCATCAACTGCTYGCWTCTGCCATTGGCTGTAAAACTTACAAAATGAAGTACGGAAATCGTGGCCATAACTTACCGGCTGTYCACCACGGAACCAATCGTTGCTACATGACTTCACAAAATCATGGWTTCGCCGTTGACGCAAAGACAATCGGAAAAGACTGGGAGCCACTTTTCACAAACTTGAATGATTTCATTCGAGTCAGCAAACACATCGGAAGTTCAATGAAATCTGTCGGAGAAGTCATGGCGATTGGCAGAAAATTCGAGGAAGCTTTCCAGAAGGCACTACGAATGGTTGACGAGAATGTCAATGGATTTGATCCGAACTTGAAGCAAGTCAGYGATGAAGAATTGACGCAACCAACTGATAAGCGAATGTTTGTCCTCGCGGCTGCTTTGAAGGCAGGCTACACYGTTGAYCGACTCTACGACTTGACGAAAATCGACCGTTGGTTCCTCGAGAAAATGAAGAATATCAACGCGTGCACRATTGAACTCGAAAARGCTAACAATTGCTCGATTTCCAATGATCTTTTGAAACACGCAAAGCGACTCGGATTTTCTGACAAACAAATCGCGACGGCAATCAAA--ATCTGAGCTAGAAGTAAGAAAACAACGACGAGAAGCTGGAATTCTTCCGTTTGTTAAGCAAATCGACACGGTGGCTGGTGAATGGCCGGCTTCAACRAATTATCTCTATTTGACTTTCAACGCCAGCGCTCACGACATTGAATTCAAC---GAACAAATGGTGATGGTCATCGGYTCRGGMGTTTATCGAATCGGAAGYTCCGTTGAGTTCGACTGGTGYGCCGTTGGATGCTTGCGAGAGCTGAGAAAACTCGGAAAGAAGACAATCATGGTGAATTACAAYCCCGAAACCGTCTCGACTGATTAYGACATGTGCGATCGACTTTATTTTGA------------CATATCATTAGACAAGAAAGAGGGAAAAAAGAGACATTCGGAGCTCTTGGTATAATTTATGCTATATTAGCTATTGGACTCTTAGGATTTATTGTTTGAGCTCATCATATATTTACAATTGGAATAGATGTAGATACACGGGCTTATTTTACTTCTGCTACAATAATTATTGCAGTACCAACTGGAATTAAAATTTTCAGTTGATTAGCTACCCTTCATGGAACTCCTTTAAATAATTCTTCATCATTACTTTGAGCTTTGGGGTTCGTATTTTTATTTACTGTTGGGGGTTTAACAGGAGTTATTTTAGCTAATTCTTCCCTAGATATCGTGTTACATGATACATACTACGTTGTAGCTCACTTTCACTATGTACTATCCATAGGAGCTGTTTTTGCTATTATGGCAGGATTTGTTCACTGATACCCATTATTTACAGGATTAACAATAAATGAAAAATGATTAAAATCCCAATTTTCAATTATATTTTTAGGGGTTAATCTAACATTTTTTCCCCAACATTTTTTAGGATTAGCAGGAATACCTCGTCGATACTCAGACTACCCCGATGCATATACATCTTGAAATATTATATCAACTGTAGGATCAACAATTTCATTGTTTGGAATTTTATTTTTTATTTTTATTATTTGAGAAAGA

**Austrobrillia longipes** --------CTAAGAACGGCCATGCACCACT--ACCCTTAAATTC-G-AGAAAGCGCTCTCAA-ACTGTCCTACATACGTAAGTTCTG-ACCTGGTAAGCTGTCCCGTGTTGAGTCAAATTAAGCCGCAGCACCCAAATC--TTGTGTAACTCTCCGTCAATTCCTTTAAGTTTCAACTTTGCAACCATACTTCCCCCGGAAACT--AG-CTTT-AGTTTCCCTGA-AGCCTCTGAACGCACCATTAAT--AGTAGTGACGCCCAATTGCTAGCTGTCATCGTTTACAGTTAGAACTAGGGCGGTATCTAATCGCCTTCGATCCTCTAACTTTCGTTCTTGATTAATGAAAACATCCTTGGCAAATGCTTTCGCTTTAGTTAGTCTTACGACGGTCTAAGAATTTCACCTCTCGCGCCGTAATACTAATGCCCCCGACTGCTTCTATTAATCATTACCTCT-TGATCTG-TATCAAACCAATAGAAAG-CGACG----------CCCGACCGTGAAGCCGGACG-CCTA-TAAGACCGAGGTCTTGTTCCATTATTCCATGCAAAAATATTCAAGGCATATAGAGCCTGCTTTGAGCACCTTAATTTGTTCAAGGTAAAAGTAAGCCGGACTAAATAGACATCTAGCCTAGTGAAAGGCATCAATGCTATCCATTAA--GTGTCCAGTCATATAGT-CAGGTAG-ACGGAAATGATAGCA-GCCACTTA-TTGGTGGTGCCAC------ACCC-------G-AACCTGACAAAAATCAACTTCGAACGTTTT-AACCGCAACAATTTTAATATACGCTAGTGGAGCTGGAATTACCGCGGCTGCTGGCACCAGACTTGCCCTCCACTTGATCCTCACTAAAGGATTTAAACT-TCGCTCATTCCAATTACAGAAC-TATGTAA-TAGTTCTATATTGTTATTTTTCGTCACTACCTCTCC---TTCAATATTCCGTCAAACGCAATGTGCACTTTTCCCTTAGTAAGACGCTGTGATCCATTAGAAATTCATTCCAATGGCTGTT-GGCTATCCATAGGCCGGTGTGAAGTTCGTTGGCAAATGGGGGGATCTTTTACGGTTCTCTGTATTGCCCGGCGAGCACATCGTCACCTG--ATATAGCTGTCTGATGGATTTTAAAGATCATATCATTGCATTACTC-GGATAATCCGATCAACAGCGGTTAAATTTGGGATACCTTTTGGACCCGTCTTGAAACACGGACCAAGGAGTCTATCTAGTATGCAAGTAAATGGGGAAACCTATTTGCATAAAAAACTTGACTGATGGGATTACGAGTACTCCTCCATCCCGGGGTATTTTCGTCAACATATGCAAATGTATATGGGAAATATACCATGAGCATACTGGATATGACCCGAAAGATGGTGAACTATGCCTGATCAGGTTGAAGTCAGGGGAAACCCTGATGGAGGACCGAAGCAGTTCTGACGTGCAAATCGCTGACTTATCCTCTGATTGGCAATTACGGAATTCCCGACGACGGCGAATTTGACATCAATGGACTTGTACGGAATTTTGAATCGAACAACAAGATTTGGATCTCGGGATTGATCGTTGGCGAGCTGTGCGAAACTCCGTCGCATTGGCGTCAAAAGTATAAGCTGTCCGAATGGATGGAGAAGCACAACATTCCCGGAATTCAAGGCATTGACACACGCGCACTGACCAAGATGATTCGCGAGAATGGCACTGTCTTGGGCAAGATCATTCAACAGCCATCG------GGGCCGTTTCCCGGC---TTAGAGTTCAAGGACCAGAACGAGCGCAATTTGGTGGATGAAGTGTCTATCAAGAAGCCAATCACTTACAATGCATCCGGATCTCCTCGCATTTGTGCCATCGATTGTGGTTTGAAATTGAACCAGATCCGCTGCTTCATTGAGAGAGGAGCTCGCGTTGATGTCGTTCCATGGAACTACGAATTGAATCCCAAGGACTTCGACGGTTTATTCCTCAGCAACGGTCCCGGCGATCCAGTCATGTGTCGCGACACCGTGAAAATCATCAAAAAGTTTCTGACCGACTCGAATGTCAAGCCGGTGTTTGGTATTTGCTTGGGTCATCAGCTTTTAGCGACTGCAATTGGATGCAAAACTTACAAAATGAAATACGGCAATCGTGGCCACAACTTGCCGGCGCGTCACCATGGCACTAATCGCTGTTTCATGACCTCACAAAATCATGGATTTGCTGTTGATGTGAGCGACATCGATGCCAACTGGGAGCCGCTGTTTACGAATTTAAATGATTTCATTCGCGTCAGCAAACACATTGGCAGCTCCATGAAAAGTGTCGGAGAAGTCATGGCGATTGGTCGCAAGTTTGAGGAAGCTTTTCAGAAAGCACTGCGAATGGTCGATGAAAACGTCAACGGGTTTGACCCCAACTTGAAACCAGCCGACGACGAAGAGTTGACAATGCCAACTGATAAAAGGATGTTCGTCCTGGCGGCAGCTCTCAAAGCTGGCTACACTGTGGATCGCCTTTATGACTTGACCAAAATTGACCGCTGGTTCTTGGAGAAGATGAAAAATATCATCGGTATCACCTTGCAGCTGGAGGAATTGAAT---TGTGTCATTCCAAATGACTTGCTGTGTCAGGCGAAAAAATTGGGATTTTCTGACAAGCAAATTGCGACTTACATCAAG--ATCTGAACTGTCAGTTCGTAAACAACGCCGCGAGTCCGGAATTTTGCCATTTGTCAAGCAAATCGACACGGTCGCCGGTGAATGGCCGGCAACAACAAACTATTTATACTTAACATACAATGCAAGTTCACATGACATTGAGTTCACC---GAACAAATGCTGATGGTGATTGGCTCTGGCGTCTACCGCATTGGTAGTTCAGTGGAGTTCGACTGGTGTGCCGTCGGATGCCTTCGTGAATTGAGAAAGTTGGGCAAGAAGACAATCATGGTGAATTACAATCCGGAAACTGTCTCCACTGATTACGACATGTGCGATCGCCTGTACTTTGAAGAGATCTCATTCATATTATTAGACAAGAAAGTGGAAAAAAAGAAACCTTTGGAACACTAGGAATAATTTATGCTATACTAGCAATTGGTTTATTAGGATTTATTGTATGAGCCCATCATATATTCACTATTGGCATAGATGTAGATACACGAGCTTATTTTACATCTGCAACTATAATCATTGCTGTTCCCACAGGGATTAAAATTTTTAGTTGATTAGCAACTCTTCATGGAACTCAACTTAATTATTCCCCTTCTATTTTATGAGCTATTGGTTTTGTTTTTCTATTTACAGTAGGGGGACTTACCGGTGTAATCTTAGCTAACTCTTCTATTGATATTGTTCTTCATGATACATATTATGTTGTTGCTCATTTTCATTATGTTTTATCTATAGGAGCTGTATTTGCTATTATAGCAGGATTTGTTCACTGATATCCTCTATTTACCGGACTATATATAAATAAAAAATGACTAATTTCTCAATTTATTACAATATTTTTAGGAGTAAACTTAACATTTTTTCCCCAACATTTTTTAGGGTTAGCAGGGATACCTCGTCGTTATTCTGATTATCCTGATGCTTATACTTCATGAAATATTTTGTCAACAATTGGATCATCAATTTCTTTATTTAGTATTATTTTTTTTATTTTTATTGTATGAGAAAGA

**Botryocladius freemani** --------------------ATGCACCACT--ACCCTTAATTTC-A-AGAAAACGCTATCAAGTTCGTCATACCCTATTAAGTTCGG-ACCTGGTAAGTTTTCCCGTGTTGAGTCAAATTAAGCCGCAAATTCCAAACC-ATGGTGTGGACTTCCGTCAATTCCTTTAAGTTTCAACTTTGCAACCATACTTCCCCCGGAAACT--AG-CTTT-GGTTTCCCGGAAAGCTACTGAATGCACCATGAAA--AGTAGTGACATCCAATTGCTAGCTGTCATCGTTTACAGTTAGAACTAGGGCGGTATCTAATCGCCTTCGATCCTCTAACTTTCGTTCTTGATTAATGAAAACATCCTTGGCAAATGCTTTCGCTTTAGTTAGTCTTGCGACGGTCTAAGAATTTCACCTCTCGCGCCGCAATACTAATGCCCCCAACTGCTTCTATTAATCATTACCTCT-TGATCTG-TATCAAACCAATAGAAAG-CGGCT-------------ACACAGTTGCCCATGTAACCTA-TAAGACCGAGGTCTTTTTCCATTATTCCATGCAAAAATATTCAAGGCATA-AGAGCCTGCTTTGAGCACCTTAATTTGTTCAAGGTAAAAGTAAGCCGAACTAAATAGACACCTAGCCTAATAAAAGGTATCGATGCTATTCATTAA--GTGTTCAGTCATATAGTTCAAGTAA-TCGGAAATGATAACA-GCCATTTACTTGATGGCGTTAC------ACCC-------G-TACTGAACAATAATCAACTTCGAACGTTTT-AACCGCAACAATTTTAATATACGCTAGTGGAGCTGGAATTACCGCGGCTGCTGGCACCAGACTTGCCCTCCACTGGATCCTCATTAAAGGATTTATACT-TGATTCATTCCAATTACAGAAC-TAAATAATTAGTTCTATATTGTTATTTTTCGTCACTACCTCCC----TTCAATATTCCTTCAACAGCAATGTGCACTTCTTTCTTAGTAAGACGCTGTGATCCATTAGAAATTCATTCCAATGATTATTTGGCTATCAATAGGCCGGCATGAAGTTCTTCAGCAAATAACTTGATCTTTTACGGTTGGGTATATTGTTTGTTGAGCATGTCGATACCTGTATTATAGCTGTCTGATGGATTTTAAAGGTCATATTATTGCATCACTCAGAATA-CCCGATCAACAGCGGTTAAATTTGGGATACCTTTTGGACCCGTCTTGAAACACGGACCAAGGAGTCTATCTAGTATGCAAGTAAATGAGGAAACTTATTTGCAGAAAAAACTTGACTGATGGGACTACGAGTGCTCCTCCATCCCGGGGTATTTTTATCAACATATGCAAATGTATATGGAAAATATACCATGAGCATACTGGATATGACCCGAAAGATGGTGAACTATGCCTGATCAGGTTGAAGTCAGGGGAAACCCTGATGGAGGACCGAAGCAATTCTGACGTGCAAATCGCTGACTTATCCGCTAATAGGAAATTACGGCATCCCAAGTGACGAGGAGTTTGACGAGTACAAGCTGATGAAGCATTTCGAGTCGAATAACAAGGTTTGGGTCACGGGTTTGGTGGTCGGTGAATTGTGTGAAACGCCTTCGCACTGGCGTCGAAAGTACAAGTTAGCCGAATGGATGAAGAAGCACAACGTGGTTGGAATCAGCGGAATTGACACTCGAGCTTTGACCAAGAAGATTCGCGAGAACGGAACTGTCTTGGGTAAAATTATTCATCAATCTTCG------GGACCTTTTCCAGGA---TTGGAGTTCAAAGATCAAAATGCGAGAAACCTCGTTGACGAAGTTTCAATTAAAAAGCCAATTACTTACAACGCTAAAGGCTCCCCTCGAATTTGTGCCATCGATTGCGGATTGAAGTTGAACCAAATCCGTTGTTTCGTCAAACGCGGAGCTCGTGTTGACGTAGTGCCATGGGATCATCCATTGAATCCCGAAGACTTTGACGGCTTGTTCCTTAGCAACGGTCCCGGTGACCCCGTCATGTGCCAAAAGACTGTCAAAAACATTCAAAATTTCCTCGCATTGCCWAAGGTCAGACCAACGTTTGGAATTTGCTTAGGTCATCAGTTGCTGTCAACTGCAATCGGCTGTAAAACTTACAAAATGAAATACGGAAATCGTGGCCATAATCTTCCAGCTCTCCACCATGGAACCAATCGCTGTTTCATGACGTCTCAAAATCACGGCTTCGCTGTTGACGTCACGACAATCGGCAAGGACTGGGAGCCACTTTTCACCAACTTAAATGACTTTATTCGAGTCAGCAAGCATATTGGAAGTTCAATGAAGAGCGTCGGTGAAGTGATGGCAATTGGACGAAACTTTGAAGAAGCTTTCCAGAAGGCATTGCGAATGGTCGATGAGAACGTTGACGGATTTAATCCGAACCTGAAAGCGGTTTGCGACGAGGAGCTGGAGATGCCGACAGACAAGCGAATGTTTGTTCTCGCTGCTGCTCTCAAAGCCGGTTACAGTGTCGACAAGCTTTACGACTTAACAAAGATCGATCGGTGGTTCCTTGAGAAAATGAAGAACATCAACGCGGTTACGATGGAGCTGGAGAAGCTGAAC---TGCGTCATTACCAATGAGCTTCTCATGAAAGCAAAGAAACTTGGATTTTCTGACAAGCAAATCGCTACCTCGATTAAG--ATCGGAACTGGAAGTAAGAAAACAGCGACGTGAGAGCGGAATTCTGCCGTTCGTTAAGCAAATTGACACCGTGGCTGGTGAGTGGCCGGCATCAACCAACTATCTCTACTTGACATATAACGCGAGCTCTCACGACATCGAGTTCAAC---GAGCAAATGGTGATGGTGATCGGGTCCGGCGTGTATCGAATTGGAAGCTCAGTTGAGTTCGATTGGTGCGCTGTTGGTTGTTTGAGAGAGTTGAGGAAGCTCGGCAAGAAGACGATCATGGTGAACTACAATCCCGAAACAGTGTCAACCGATTACGACATGTGCGATCGACTGTACTTCGAAGAGATTTCATTCATATTATTAGTCAAGAAAGAGGAAAAAAAGAAACCTTTGGTTCTTTAGGAATAATCTATGCCATACTTGCTATTGGCCTATTAGGATTTATTGTTTGAGCTCATCATATATTTACTGTTGGGATAGATGTAGATACCCGGGCTTATTTTACTTCTGCTACAATAATTATTGCTGTGCCAACAGGTATTAAAATTTTTAGATGATTAGCTACCTTACATGGGACCCCCCTTAATAATTCTCCTTCTCTTTTATGAGCCTTAGGGTTTGTATTTTTGTTTACTGTTGGAGGGCTCACTGGCGTTATTCTAGCTAATTCTTCTATTGATATTGTTTTACATGACACTTATTATGTAGTTGCTCATTTTCACTATGTTCTATCTATGGGAGCAGTTTTTGCTATTATAGCAGGATTTGTCCACTGATACACTCTATTTACTGGTTTAATTATAAATGAAAAATGACTTAAATCTCAATTTTCAATCATATTTTTAGGAGTAAATTTAACATTTTTCCCTCAACATTTTCTTGGCCTTGCCGGGATACCCCGACGATACTCTGACTACCCTGACGCCTATACTTCATGAAATATCCTATCTACTGTTGGATCTACAATTTCTTTATTTGGTATTTTATTTTTTATTTTTATCATTTGAGAAAGA

**Brillia cf. parva** --------CTAAGAACGGCCATGCACCACT--ACCCTTAACTTC-G-AGAAATCGCTCTCAA-AATGTCTTACCCTGTTAAGTTCGG-ACCTGGTAAGTTTTCCCGTGTTGAGTCAAATTAAGCCGCAAATTCCACTTC-CAGTGATGACCTTCCGTCAATTCCTTTAAGTTTCAACTTTGCAACCATACTTCCCCCGGAAACT--AG-CTTT-GGTTTCCCGGA-AGCTACTGAATTCACCTTAATA--TTAAGTAAAATCCAATTGCTAGCTGTCATCGTTTACAGTTAGAACTAGGGCGGTATCTAATCGCCTTCGATCCTCTAACTTTCGTTCTTGATTAATGAAAACATCCTTGGCAAATGCTTTCGCTTTAGTTAGTCTTACGACGGTCTAAGAATTTCACCTCTCGCGCCGTAATACTAATGCCCCCGACTGCTTCTATTAATCATTACCTCT-TGATCTG-TATCAAACCAATAGAAAG-CGACA----------CGCAACATTGCTGCTACGCA-TCTA-TAAGACCGAGGTCCTTTTCCATTATTCCATGCAAAAATATTCAAGGCATT-AGAGCCTGCTTTGAGCACCTTAATTTGTTCAAGGTAAAAGTAAGCCGAACTAAATAGACATCTATCCTAGTTAAAGGCATCAATGCTATTCATTTA--GTGTTCAGTCATATAGTCCAAGTCA-TCGGAAATGATAGCA-ACCG--CACTAAGCGGTGCCAC------ACCC-------G-TACTGGACAACAATCAACTTCGAACGTTTT-AACCGCAACAATTTTAATATACGCTAGTGGAGCTGGAATTACCGCGGCTGCTGGCACCAGACTTGCCCTCCACTTGATCCTCATTAAAGGATTTATACT-TGATTCATTCCAATTACAGAACATAGTTAACTAGTTCTATATTGTTATTTTTCGTCACTACCTCC-----TTCAATATTCCGTTAAACGCAATGTGCACTTTTCTCTTAGTAAGACGTTGTGATCCATTAGAAATTCATACCAATGGTTAGTGGGCTATCAATAGGCCGGCATGAAGTTCACCGGCAAATGACTGAATCCTAAATGGTTTTGTGTATTGCTTGGTGAGCATGCTGATACCTGCA--ATAGCTGTCTGATGGATTTTAAAGATCATATCACTGCATTACCCTGGATAATCCGATCAACAACGGTTAAATTTGGGATACCTTTTGGACCCGTCTTGAAACACGGACCAAGGAGTCTATCTAGTATGCAAGTAAATGAGGAAACTTATTTGCGTAAAAAACTTGACTGATGGGATTACAAGTGCTTCTCCATCCCGGGGTATTTTTATCAACATATGTAAATATATATGGAAAATATACCATGAGCATACTGGATATGACCCGAAAGATGGTGAACTATGCCTGATCAGGTTGAAGTCAGGGGAAACCCTGATGGAGGACCGAAGCAGTTCTGACGTGCAAATCGCTGACTTACCCGCTGATTGGCAACTACGGCATTCCGGACGCCGACGAACTGGACATTGACGGATTGGTGAAGAACTTTGAATCGAAC---AAGATTTGGATCGCCGGACTGGTTGTGGGTGAGTTGTGTGAAGTGCCTTCGCATTGGCGTCAGAAATACAAGCTCGCCGAATGGATGAAGAAGCACAACATTCCCGGCATCAGCGGCATTGACACGCGCGCTCTCACAAAGACGATTCGCGAAAACGGAACTGTTTTGGGAAAGATCATTCAACAGCCCTCC------GGGCCCTTCCCAGGA---CTCGAATTCAAGGACCAGAACGAGAGGAACCTCGTMGACGAGGTTTCCATCACGAAGCCGGTGACTTACAATGCTTCCGGTTCGCCACGCATCTGTGCCATCGATTGTGGTTTGAAGTTGAATCAGATTCGTTGCTTCACGAAACGCGGAGCGCGAGTTGACGTTGTGCCATGGAACCACGAGCTCAACCCCAACGACTGTGATGGACTCTTCTTGAGCAACGGCCCCGGAGATCCCGTCACGTGCCAAAAGACCGTTAAGAACATTCAGAGCTTCCTGTCCAACGCAAAAGCGAAACCAACTTTCGGCATCTGCTTGGGTCACCAACTGCTGGCCACTGCCGTCGGCTGCAAAACTTACAAGATGAAGTACGGCAATCGCGGTCACAACCTGCCGGCTCTTCATCACGGCTCCAATCGCTGCTTCATGACGTCACAAAATCACGGATTTGCTGTTGACGCCACTACGCTCGGCCGCGATTGGGAGCCACTTTTCACAAATCTTAATGAC------------------------------------------------GAAGTGATGGCGATTGGAAGAAAGTTTGAAGAAGCTTTCCAGAAAGCTCTCCGCATGGTCGACGAGAACGTCAACGGATTCGATCCCAACTTGAAGTTGGTGAATGACGAAGAGCTCCAAACGCCAACAGACAAGCGAATGTTTGTGCTCGCTGCAGCCTTGAAAGCCGGCTACACAGTGGACCGTCTCTACGACTTGACCAAGATCGATCGCTGGTTCCTGGAGAAAATGAAGAACATCATCGGCATCAGCAACGAGCTGGAAGCAGCAAGCAATTGTACRATTTCAAATGAATTGCTGGAAGATGCCAAGAAGCACGGCTTTTCCGACAAGCAGATTGCATCATTCATCAAA--ATCAGAACTGGCAGTGAGAAAGCAACGACGTGACGCTGGAATTCGTCCGTTTGTGAAGCAAATCGATACTGTCGCTGGTGAGTGGCCTGCAACCACAAAYTATCTYTACTTGACYTACAACGCATCAACGCACGACATTGAGTTTGAC---GAAGAGATGGTGATGGTCATTGGATCTGGAGTTTATCGCATTGGCAGCTCAGTTGAGTTTGATTGGTGTGCCGTTGGYTGCTTGCGCGAGCTGCGAAAGCTCGGCAAGAAGACGATTATGGTRAACTACAATCCAGARACT-----------------------------------------------------CATATTATCAGCCAAGAGAGAGGAAAAAAAGAAACTTTTGGGGCTCTGGGAATAATTTATGCCATATTAGCAATTGGTTTATTAGGCTTTGTAGTCTGAGCCCATCACATATTTACAGTCGGAATAGATGTGGATACTCGGGCTTATTTTACCTCTGCAACAATAATTATTGCTGTGCCAACAGGAATTAAAATTTTTAGCTGATTAGCCACATTACACGGAGCCCAATTAAACTATTCACCAGCTCTTTTATGAGCTCTTGGATTTGTATTTTTATTCACAGTCGGAGGATTGACAGGAGTTATTTTAGCTAATTCTTCTATTGATATTGTACTTCATGATACTTATTATGTAGTGGCCCATTTTCACTATGTTCTTTCTATAGGAGCCGTTTTTGCTATTATAGCAGGATTCGTGCACTGATACCCACTATTTACAGGGTTAAATTTAAACGAAAAATGACTAAAATCTCAATTTACAATAATGTTTTTAGGTGTTAATTTAACCTTTTTTCCACAACACTTTTTAGGTTTAGCAGGAATACCTCGTCGATATTCAGATTACCCCGATGCATATACTTCATGAAATATTCTCTCAACAATCGGATCTACAATTTCTTTATTAGGAGTATTATTTTTTATCTTTATTATTTGAGAAAGA

**Brillia gp. sp. 1** -------ACTAAGAACGGCCATGCACCACT--ACCCTTAACTTC-G-AGAAATCGCTCTCAA-AATGTCTTACCCTGTTAAGTTCGG-ACCTGGTAAGTTTTCCCGTGTTGAGTCAAATTAAGCCGCAAATTCCACATC-CGGTGATGACCTTCCGTCAATTCCTTTAAGTTTCAACTTTGCAACCATACTTCCCCCGGAAACT--AG-CTTT-GGTTTCCCGGAAAGCTACTGAATTCGCCATTAAT--ATTAGCGAAATCCAATTGCTAGCTGTCATTGTTTACAGTTAGAACTAGGGCGGTATCTAATCGCCTTCGATCCTCTAACTTTCGTTCTTGATTAATGAAAACATCCTTGGCAAATGCTTTCGCTTTAGTTAGTCTTACGACGGTCTAAGAATTTCACCTCTCGCGCCGTAATACTAATGCCCCCGACTGCTTCTATTAATCATTACCTCT-TGATCTG-TATCAAACCAATAGAAAAACGACA------------------ATATAAATATCA-TCTA-TAAGACCGAGGTCCTTTTCCATTATTCCATGCAAAAATATTCAAGGCATT-AGAGCCTGCTTTGAGCACCTTAATTTGTTCAAGGTAAAAGTAAGCCGAACTAAATAGACGCCTATCCTAATTAAAGGCATCAACGCTATTCATTTA--GTGTTCAGTCATATAGTCCAAGTCA-TCGGAAATGATAGCA-ACCG--CACTAAACGGTGCCAC------ACCC-------G-TACTGGACAAAAATCAACTTCGAACGTTTT-AACCGCAACAATTTTAATATACGCTAGTGGAGCTGGAATTACCGCGGCTGCTGGCACCAGACTTGCCCTCCACTTGATCCTCATTAAAGGATTTATACT-TGATTCATTCCAATTACAGAACATAGTTAACTAGTTCTATATTGTTATTTTTCGTCACTACCTCC-----TTCAATATTCTTGCAAAAGCAATGTGCACTTTTCTCTTAGTAAGACGTTGTGATCCATTAGAAATTTATACCAATAATTATTAGGCTATCAATAGGCCGGCATGAAGTTCATTGGCAAATGACTGAATCTTTAAAGGTTTTGTGTATTGCCTAGTGAGCATGCTGATACCTGAA--ATAGCTGTCTGATGGATTTTAAAGATCATATCACTGCATTACCCAGGATA-TCCGATCAACAACGGTTTAATTTGGGATACCTTTTGGACCCGTCTTGAAACACGGACCAAGGAGTCTATCTAGTATGCAAGTAAATGAGTAAACTTATTTGCGTAAAAAACTTAACTGATGGGATTACAAGTGCTTCTCCATCCCGGGGTATTTTTATCAACATATGCAAATGTATATGGAAAATATACCATGAGCATACTGGATATGACCCGAAAGATGGTGAACTATGCCTGATCAGGTTGAAGTCAGGGGAAACCCTGATGGAGGACCGAAGCAGTTCTGACGTGCAAATCGCTAACTTATCCCTTAATTGGTAATTACGGCATTCCTGATGACGCTGAACTTGACGTTGATGGCTTGATAAAGAATTTCGAATCGAATAACAAGATTTGGGTTGCTGGTCTTGTTGTTGGTGAACTTTGTGAAGTTCCATCGCATTGGCGTCAAAAATACAAACTTGCTGAATGGATGAAGAAGCATAATATTCCTGGAATTAGTGGCATTGATACGCGTGCATTAACGAAAACAATTCGAGAGAATGGAACAATTTTAGGGAAAATCATTCARCAGCCTTCC------GGTCCATTTCCAGGA---CTTGCGTTTGAAGATCAAAACCAACGCAATTTAGTTGATGAAGTKTCAATTAAGAAACCAATAACATACAACGCTTCCGGTTCGCCAAGAATTTGTGCCATTGATTGCGGCTTAAAATTAAATCAAATTCGTTGCTTTACAAAACGCGGTGCAAGAGTTGATGTTGTGCCATGGAATTATGAACTCAATCCGAATGACTGTGATGGACTTTTCTTAAGCAATGGTCCTGGTGATCCTGTCACTTGCCAAAAGACTGTCAAAATTATTCAAAACTTTCTTGCGAATGCCAACGCAAAGCCGACATTTGGAATTTGTTTAGGTCATCAATTATTAGCAACTGCGATTGGATGCAAAACTTACAAAATGAAATATGGCAATCGTGGCCACAACTTGCCAGCACTTCATCATGGAACTAATCGCTGCTTTATGACATCACAAAATCATGGATTTGCTGTTGATGCAACGACACTT---------------------------------------NNNNNNNNNNNNNNNNNNNNNNNNNNNNNNNNNNNNNNNNNNNNNNNNNNNNNNNNNNNNNNNNNNNNNNNNNNNNNNNNNNNNNNNNNNNNNNNNNNNNNNNNNNNNNNNNNNNNNNNNNNNNNNNNNNNNNNNNNNNNNNNNNNNNNNNNNNNNNNNNNNNNNNNNNNNNNNNNNNNNNNNNNNNNNNNNNNNNNNNNNNNNNNNNNNNNNNNNNNNNNNNNNNNNNNNNNNNNNNNNNNNNNNNNNNNNNNNNNNNNNNNNNNNNNNNNNNNNNNNNNNNNNNNNNNNNNNNNNNNNNNNNNNNNNNNNNNNNNNNNNNNNNNNNNNNNNNNNNNNNNNNNNNNNNNNNNNNNNNNNNNNNNNNNNNNNNNNNNNNNNNNNNNNNNNNNNNNNNNNNNNNNNNNNNNNNNNNNNNNNNNNNNNNNNNNNNNNNNNNNNNNNNNNNNNNNNNNNNNNNNNNNNNNNNNNNNNNNNNNNNNNNNNNNNNNNNNNNNNNNNNNNNNNNNNNNNNNNNNNNNNNNNNNNNNNNNNNNNNNNNNNNNNNNNNNNNNNNNNNNNNNNNNNNNNNNNNNNNNNNNNNNNNNNNNNNNNNNNNNNNNNNNNNNNNNNNNNNNNNNNNNNNNNNNNNNNNNNNNNNNNNNNNNNNNNNNNNNNNNNNNNNNNNNNNNNNNNNNNNNNNNNNNNNNNNNNNNNNNNNNNNNNNNNNNNNNNNNNNNNNNNNNNNNNNNNNNNNNNNNNNNNNNNNNCATATTATTAGTCAAGAAAGTGGAAAAAAGGAAACTTTTGGAGCTTTAGGAATAATTTATGCTATACTTGCAATTGGTTTATTAGGTTTTGTTGTATGAGCTCATCACATATTTACAGTTGGAATAGACGTAGATACACGGGCTTATTTTACATCTGCAACTATAATTATTGCTGTACCAACAGGAATTAAAATTTTTAGATGATTAGCTACTCTTCACGGAACTCAATTAAATTACTCTCCTGCATTATTATGAGCTTTAGGGTTTGTCTTTTTATTTACTGTAGGAGGATTAACTGGAGTAATTTTAGCTAATTCTTCTATTGATATTGTTCTTCATGATACTTATTATGTAGTTGCTCATTTCCATTATGTGCTATCAATAGGAGCTGTATTTGCTATTATAGCAGGATTTGTTCACTGATATCCTCTATTTACAGGTCTTAATTTAAACGAAAACTGATTAAAGTCTCAATTTTTAATGATATTCTTAGGAGTAAATTTAACTTTTTTTCCTCAACATTTTTTAGGGTTAGCAGGAATACCTCGTCGATATTCTGATTATCCAGATGCTTACACTTCATGAAACATTCTCTCTTCAATTGGATCAACAATTTCTTTATTAGGAGTTTTATTTTTTTTATTTATTATTTGAGAAAGT

**Brillia gp. sp. 2** --------CTAAGAACGGCCATGCACCACT--ACCCTTAACTTC-G-AGAAATCGCTCTCAA-AATGTCTTACCCTGTTAAGTTCGG-ACCTGGTAAGTTTTCCCGTGTTGAGTCAAATTAAGCCGCAAATTCCACTTC-CGGTGATGACCTTCCGTCAATTCCTTTAAGTTTCAACTTTGCAACCATACTTCCCCCGGAAACT--AG-CTTT-GGTTTCCCGGAAAGCTACTGAATTCGCCATTAAT--ATTAGCGAAATCCAATTGCTAGCTGTCATTGTTTACAGTTAGAACTAGGGCGGTATCTAATCGCCTTCGATCCTCTAACTTTCGTTCTTGATTAATGAAAACATCCTTGGCAAATGCTTTCGCTTTAGTTAGTCTTACGACGGTCTAAGAATTTCACCTCTCGCGCCGTAATACTAATGCCCCCGACTGCTTCTATTAATCATTACCTCT-TGATCTG-TATCAAACCAATAGAAAAACGATA--------TAACATATTTGCATACATTACA-TCTA-TAAGACCGAGGTCCTTTTCCATTATTCCATGCAAAAATATTCAAGGCATT-AGAGCCTGCTTTGAGCACCTTAATTTGTTCAAGGTAAAAGTAAGCCGAACTAAATAGACGCCTATCCTAATTAAAGGCATCAACGCTATTCATTTA--GTGTTCAGTCATATAGTCCAAGTCA-TCGGAAATGATAGCA-ACCA--CACTAAATGGTGCCAC------ACCC-------G-TACTGGACAACAATCAACTTCGAACGTTTT-AACCGCAACAATTTTAATATACGCTAGTGGAGCTGGAATTACCGCGGCTGCTGGCACCAGACTTGCCCTCCACTTGATCCTCATTAAAGGATTTATACT-TGATTCATTCCAATTACAGAACATAGTTAACTAGTTCTATATTGTTATTTTTCGTCACTACCTCC-----TTCAATATTCTTGCAAAAGTAATGTGCACTTTTCTTTTAGTAAGACGTTGTGATCCATTAGAAATTTATACCAATAATTAGTGGGCTATCAATAGGCCAGCATGAAGTTCATTGGCAAATGACTGAATCTTTAATGGTTTTGTGTATTGCCTAGTGAGCATGTTGATACCTGAA--ATAGCTGTCTGATGGATTTTAAAGATCATATCACTGCATTACCCAGGATA-TCCGATCAACAACGGTTAAATTTGGGATACCTTTTGGACCCGTCTTGAAACACGGACCAAGGAGTCTATCTAGTATGCAAGTAAATGAGTAAACTTATTTGCGTAAAAAACTTAACTGATGGGATTACAAGTGCTTCTCCATCCCGGGGTATTTTTATCAACATATGCAAATGTATATGGAAAATATACCATGAGCATACTGGATATGACCCGAAAGATGGTGAACTATGCCTGATCAGGTTGAAGTCAGGGGAAACCCTGATGGAGGACCGAAGCAGTTCTGACGTGCAAATCG------------------GGAAATTATGGCATACCTGATGACACTGAACTTGATATTGATGGATTGGTAAAGAATTTTGAATCGAATAATAAAATTTGGGTTGCTGGTCTTGTTGTTGGTGAGCTGTGTGAAGTTCCATCGCATCGGCGTCAAAAATACAAGCTAGCTGAATGGATGAAGAAGCATAATATTCCTGGAATTAGTGGCATTGATACGCGTGCATTAACAAAAACAATCCGTGAAAATGGAACAATTTTAGGGAAAATCATTCAACAACCTTCC------GGTCCATTTCCTGGA---CTTGCCTTTGAGGACCAAAACCAACGAAACTTGGTCGATGAAGTGTCAATTATGAAGCCAATAACCTACAATGCTTCCGGTTCACCAAGAATTTGTGCCATTGATTGTGGTTTGAAATTAAATCAAATTCGTTGCTTTACTAAACGTGGGGCACGTGTTGATGTTGTGCCATGGGATTATGAAATCAATCCAAATGACTGTGATGGACTCTTTTTGAGCAATGGTCCTGGTGATCCTGTCACTTGCCAAAAGACTGTCAAAAACATTCAAAATTTTCTTGCCAATGTTAAAGCAAAACCAACTTTTGGAATTTGTTTGGGTCATCAACTTTTAGCGACTGCAATTGGATGCAAAACATATAAAATGAAGTATGGTAATCGTGGCCATAATTTGCCAGCACTACATCATGGA---------------------------------------------------------------------------------------------------------CGTGTCAGCAATCACATTGGAAGTTCCATGAAGAGTGTTGGTGAAGTAATGGCGATTGGTAGGAAATTTGAAGAAGCTTTTCAGAAAGCTTTACGAATGGTTGATGAGAACGTCAATGGTTTTGATCCAAATTTAAAGACTGTTAATGATGAAGAACTTCAAACACCAACTGATAAGCGCATGTTTGTACTTGCTGCAGCATTAAAAGCTGGCTATACGGTGGATCGTTTGTATGACTTGACAAAAATTGATCGTTGGTTTTTGGAGAAAATGAAAAATATTATTGGAATTTCGAATCAATTAGAAGCAGCAATTAATTGCACAATTCCCAATGAGTTATTGGAAGAAGCCAAGCAACATGGATTTTCTGATAAACAAATTGCGACATTCATTAAA--TTCCGAATTGGCTGTAAGAAAGCAACGACGCGAAGCTGGAATTCTTCCATTTGTTAAACAAATCGACACAGTCGCTGGCGAATGGCCAGCAACAACAAATTATCTTTACTTGACATACAATGCAAGTTCACATGACATTGAATTTRAT---GAAGAAATGGTGATGGTGATTGGTTCGGGAGTTTATCGTATTGGAAGTTCCGTAGAATTTGATTGGTGCGCTGTTGGTTGTTTGCGTGAACTCAGAAAGTTGGGCAAGAAGACAATCATGGTGAATTACAATCC------------------------------------------------------------CACATTATTAGACAAGAAAGAGGAAAAAAGGAAACCTTTGGTGCCTTAGGTATAATTTACGCCATACTTGCTATTGGTTTATTAGGTTTCGTAGTTTGAGCTCACCATATATTTACTGTAGGAATAGATGTTGATACACGAGCTTATTTTACTTCAGCAACTATAATTATTGCTGTACCTACTGGAATTAAAATTTTTAGATGATTAGCTACACTTCACGGCACTCAACTTAACTATTCTCCAGCTTTACTATGAGCTTTAGGGTTTGTCTTTTTATTTACCGTAGGGGGATTAACAGGAGTAATTTTAGCTAATTCTTCTATTGACATTGTTCTTCACGATACTTATTATGTAGTAGCCCATTTTCACTATGTTTTATCTATAGGGGCAGTATTTGCTATTATAGCAGGATTTGTACACTGATATCCTCTATTTTCAGGATTAAACTTAAATGAAAAATGATTAAAGTCTCAATTTTTAATAATATTCCTAGGAGTTAATTTAACCTTTTTTCCTCAACACTTTTTAGGTTTAGCAGGAATACCTCGTCGTTATTCTGATTATCCAGATGCTTACACATCTTGAAACATTTTATCATCTATTGGATCAACTATTTCCTTATTAGGAGTATTATTCTTTTTATTTATTATTTGAGAAAGA

**Cardiocladius sp. 1** TCCACGAACTAAGAACGGCCATGCACCACT--ACCCTTAACTTC-A-TGAAAACGCTCTCAA-GATGTCATACCCTGTTAAGTTCTG-ACCTGGTAAGTTTTCCCGTGTTGAGTCAAATTAAGCCGCAAATTCCATTCT--TTGGGTGGTCTTCCGTCAATTCCTTTAAGTTTCAACTTTGCAACCATACTTCCCCCGGAAACT--AG-CTTT-GGTTTCCCGGAAAGCTACTGAATGCACCATGAAT--AGTAGTGACATCCAATTGCTAGCTGTCATCGTTTACAGTTAGAACTAGGGCGGTATCTAATCGCCTTCGATCCTCTAACTTTCGTTCTTGATTAATGAAAACATCCTTGGCAAATGCTTTCGCTTTAGTTAGTCTTGCGACGGTCTAAGAATTTCACCTCTCGCGCCGCAATACTAATGCCCCCAACTGCTTCTATTAATCATTACCTCT-TGATCCG-TATCAAACCAATAGAAAG-CGACG------------CCACACGTGAATGCAACG-CCTA-TAAGACCGAGGTCTTTTTCCATTATTCCATGCAAAAATATTCAAGGCATA-AAAGCCTGCTTTGAGCACCTTAATTTGTTCAAGGTAAAAGTAAGCCGAACTAAATAGACGTCTAGCCTAGTGAAAGGCATCGGCGCTATTCATTAA--GTGTCCGGTCATATAGTTCGAGCAATCCGGAAATGATGGCA-GCCA--CACTTGATGGTGCCAC------ACCC-------G-TGCTGAACAACAATCAACTTCGAACGTTTT-AACCGCAACAATTTTAATATACGCTAGTGGAGCTGGAATTACCGCGGCTGCTGGCACCAGACTTGCCCTCCACTTGATCCTCATTAAAGGATTTATACT-TGATTCATTCCAATTACAGAAC-TAAATAATTAGTTCTATATTGTTATTTTTCGTCACTACCTCCC----TTCAATATTCTGTTAAAGGCAATGTGCACTTCTCTCTTAGTAAGACGCCGTGATCCATTAGAAATTCATTTCAATGATTATTTGGCTATCAATAGGCCAGCACGAAGTTCATCAACAAATAACTCGATCTTATATGGCTGGGTATATTGTATGGTGAGCGTGTTGTAACCTGCTTAATAGCTGTCTGATGGATTTTAAAGGCCATATTATT-CATCACTCAAGATA-CCCGATCAACGGCGGTCAAATTTGGGATACCTTTTGGACCCGTCTTGAAACACGGACCAAGGAGTCTATCTAGTGTGCAAGTAAATGAGGAAACTTATTTGCAAAAGAAACTTGACTGATGGGACTACGAGTGCTCCTCAATCCCGGGGTATTTTTGTCAACATATGCAAATGTATATGGAAAATATACCATGAGCATACTGGATATGACCCGAAAGATGGTGAACTATGCCTGATCAGGTTGAAGTCAGGGGAAACCCTGATGGAGGACCGAAGCAATTCTGACGTGCAAATCG---------------------------GGCATCCCGAGTGACGAGGAGTTCGATGAGCATAAATTGATGAAGCATTTCGAGTCCAACAACAAGATTTGGGTGTCTGGATTGGTTGTTGGAGAGCTCTGTGAGACTCCTTCACATTGGAGGCGCAAGTACAAACTCGCCGAATGGATGAAGAAGCATAACATCGTTGGTATCAGTGGCATCGACACCAGAGCATTGACGAAGAAGATTCGAGAAGAAGGAACTGTCTTAGGCAAGATCATTCAGCAATCTAGC------GGACCATTCGCTGGT---TTAGAGTTCGTCGACCAGAATGAGCGAAACCTCGTCAAGGAAGTCTCCATCAAAAAGCCGGTTGTCTACAATCCAAATGGCTCACCTCGAATCTGCGCTGTCGATTGCGGTCTAAAATCTAATCAGATTCGCTGCTTCATTGAACGAGGAGCTCGTGTCGATGTTGTTCCATGGGACCATCCATTGAACCCCGACGACTGCGACGGACTCTTCCTGAGCAACGGTCCTGGTGACCCTGTRGTATGTCAAAATACTGTCGAAAATATTCGAAAATTCATCGCTTTGCCTAAAATGAGGCCAATCTTTGGCATTTGCCTCGGTCATCAACTCCTGTCAACTGCTATCGGATGCAAAACTTTTAAATTGAAATACGGAAATCGAGGCCATAACTTGCCGGCTATTCATCATGGAACCAACCGATGCTTCATGACTTCGCAGAACCACGGATTTGCCGTTGATATCAAGTCACTTCCAAAAGACTGGGAGCCGCTTTTCACCAATTTGAATGACTTCACCAGAGTCAGCAAACACATCGGAAGCTCAATGAAGAGCGTCGGCGAAGTTATGGCGATTGGACGAAAATTCGAAGAAGCTTTCCAAAAGGCATTGAGGATGGTCGATGAAAATGTCAACGGATTTGACCCAAATCTAAAGCCAGTCAGCGACGAAGATCTCAGAACGCCAACTGACAAGCGAATGTTTGTCCTCGCAGCTGCCTTGAAGGCAGGATACACCGTGGATCGTCTCTATGAYTTAACAAAGATTGATCGCTGGTTCTTGGAGAAGATGAGAAACATCAACAACATCACGCTCGAGTTGGAGAAGCTGAAC---TGCGTCATCACTGATGATTTGCTGCGACAAGCTAAGCGACTTGGATTCTCTGATAAACAAATCGCGAGCCACATTCGA--ATCAGAACTTGCCGTTCGAAAACAGCGACGAGAGAGTGGAATCTTGCCTTTTGTGAAGCAAATCGACACTGTAGCTGGAGAATGGCCAGCCTCCACCAATTACCTTTACTTGACGTACAACGCGAGTGCTCATGACGTTGAGTTCAAT---GATCAGATGGTGATGGTAATTGGTTCAGGTGTGTATCGTATCGGAAGCTCCGTGGAGTTCGATTGGTGCGCTTGTGGATGTCTCAGAGAGCTCCGCAATCTCGGCAAGAAGACCATCATGGTGAACTACAATCCCGAGACAGTATCTACCGATTACGACATGTGCGACCGATTATACTTTGAGGAAATTTCCTTCACATCATTAGACAAGAAAGAGGAAAAAAAGAAACATTTGGGGCTTTAGGAATAATTTATGCCATACTAGCTATYGGCCTTTTAGGATTTGTCGTATGAGCTCATCATATATTTACTGTCGGCATAGATGTTGATWCCCGGGCYTACTTTACCTCAGCAACTATAATTATTGCAGTRCCTACCGGAATTAAAATTTTTAGATGACTRGCTACATTACATGGAACTCAAATAAATAATTCCCCYTCTTTACTTTGAGCTTTAGGATTTGTATTTTTATTCACCGTTGGGGGTTTAACCGGAGTAGTATTAGCAAATTCTTCTATTGACATTGTACTTCATGACACTTATTATGTTGTGGCACATTTTCATTATGTTCTTTCTATAGGAGCAGTATTCGCAATTATAGCTGGATTTGTCCACTGATACCCTTTATTTACTGGATTAATAATAAATGAAAAATGACTAAAATCCCAATTTGCAATTATGTTCTTAGGTGTAAATCTAACCTTTTTTCCCCAACACTTTTTAGGATTAGCAGGAATACCTCGTCGTTACTCTGATTACCCAGATGCTTACACATCGTGAAATATCATTTCTACTATTGGCTCAACAATTTCTTTATTTGGGATTTTATATTTCTTATTTATTATTTGAGACAGA

**Clunio marinus** -------------------------CCACT--ACCCTTAATTTC-A-TGAAAACGCTATCAA-ATTGTCATACCCTATCAAGTTCGG-ACCTGGTAAGTTTTCCCGTGTTGAGTCAAATTAAGCCGCATATTCCAAGTC-ATTGTGTGATCTTCCGTCAATTCCTTTAAGTTTCAACTTTGCAACCATACTTCCCCCGGAAACT--AG-CTTT-GGTTTCCCGGAAAGCTACTGAATGCACCATGAAA--AGTAGTGACATCCAATTGCTAGCTGTCATCGTTTACAGTTAGAACTAGGGCGGTATCTAATCGCCTTCGATCCTCTAACTTTCGTTCTTGATTAATGAAAACATCCTTGGCAAATGCTTTCGCTTTAGTTAGTCTTGCGACGGTCTAAGAATTTCACCTCTCGCGCCGCAATACTAATGCCCCCAACTGCTTCTATTAATCATTACCTCT-TGATCTG-TATCAAACCAATAGAAAC-CGATA----------AGTATCTCAAAGAGATACAAATCTA-TAAGACCGAGGTCTTTTTCCATTATTCCATGCAAAAATATTCAAGGCATA-AGAGCCTGCTTTGAGCACCTTAATTTGTTCAAGGTAAAAGTAAGCCGAACTAAATAGACGCCTAGCCTAATTAAAGGCATCAGCGCTATTCATTAA--GTGTTCAGTCATATAGTTCAAGCAA-TCGAAAATGATAACA-GCCATTTACTTGATGGCGTTAC------ATCC-------G-TGCTGAACAATAATCAACTTCGAACGTTTT-AACCGCAACAATTTTAATATACGCTAGTGGAGCTGGAATTACCGCGGCTGCTGGCACCAGACTTGCCCTCCACTGGATCCTCATTAAAGGATTTATACT-TGACTCATTCCAATTACAGAAC-TAAATAATCAGTTCTATATTGTTATTTTTCGTCACTACCTCCT----TTCAATATTCCCTTAAAAGCAATGTGCACTTCTCTCTTAGTAAGACGCTGTGATCCATTAGAAATTTGTATCAACAATCGTTTGGCTATCGATAGGCCGATGTGAAGTTCATTGACAAATAACTTGATCTTTTACGGTTAGGTATATTGTTTAGTGAGCACATTGTAACCTG-ATAATAGCTGTCTGATGGATTTTAAAGGTCATATTATTACATTACTCATGATAACCCGATCAACAGCGGTTAAATTTAGCATACCTTCTGGACCCGTCTTGAAACACGGACCAAGGAGTCTATCTAGTATGCAAGTAAATGAGAAAACTTATTTGCATAAAAAACTTGACTGATGGGACTACGAATATTCTTCCATCCCGGGGTATTTTTATCAACATATACAAATGTATATGGAAAATATACCATGAGCATACTGGATATGACCCGAAAGATGGTGAACTATGCCTGATCAGGTTGAAGTCAGGGGAAACCCTGATGGAGGACCGAAGCAATTCTGACGTGCAAATCGCTGACTTATCCATTGATTGGAAATTATGGCGTTCCGAGTGATGAAGAATTTGACGTACATAAATTGATGAAAAACTTTGAAGCGAATGACAAGATTTGGATTTCCGGACTTGTTGTTGGCGAACTATGTGAAACTCCCTCGCATTGGCGTCAAAAATACAAACTTGCTGAATGGATGAAGAAGCATAATATTGTTGGAATTAGTGGCATTGATACTCGTGCTTTAACTAAAAGGATTCGTGAAAATGGAACCGTTTTAGGTAAAATTATCCAGCAATCAGGA------GGACCATTTCCTGGT---TTAGAGTTCAAAGATCAAAATGAAAGGAATCTTGTCGATGAAGTGTCAATTAAAAAACCGATAACTTACAATCCAAAAGGTTCGCCACGTATTTGTGCTGTTGATTGTGGCTTGAAACTAAATCAAATTCGTTGCTTCATTCAACGTGGAGCTCGTGTTGATCTAGTGCCTTGGGATCATCCATTAAATGTCAATGATTTTGATGGGCTCTTCTTAAGCAACGGTCCTGGCGATCCCGGAATGTGCAACAAAACTGTCGAGAACATTCGAAAGATTCTTTCAGCTCAAAACATCAAACCAATTTTTGGGATTTGTTTGGGTCATCAACTTTTAGCAACTGCAATTGGATGTAAGACTTACAAGTTGAAATATGGTAATCGTGGCCATAATTTACCAGCTTTACATCATGGAACAAAACGATGCTTTATGACGTCACAAAATCATGGATTTGCTGTTGATGTATCAACAATTGCCAAGGATTGGGAGCCTCTTTTTACAAACCTTAACGATTTCATCCGAGTCAGCAATCACATTGGAAGTTCCATGAAAAGTGTCGGCGAAGTTATGGCGATTGGCAGGAAGTTCGAAGAAGCTTTTCAAAAAGCACTTCGAATGGTCGATGAGAATGTCAATGGATTTGATCCAAATTTAAAGCCTGTGAATGAAGAAGAATTGAAGACGCCAACAGACAAACGAATGTTTGTACTTGCAGCAGCTTTAAAGAGCGGCTACACAGTCGACAAGTTGTATGACTTGACAAAAATTGATCGATGGTTCCTAGAGAAGATGAAAAACATCATTGTCGTTACAATGGAATTGGAGAAACTTAAT---TGTGTGATAACTGACGAACTTTTACTTGAATCGAAGAAACTTGGATTCTCTGATAAGCAAATCGCAACTTACATCAAA--ATCGGAACTTGCTGTTCGAAAACAGAGGATCGAGAATGGATTTATGCCWTTTGTGAAGCAAATTGACACTGTCGCTGGTGAATGGCCAGCTTCAACTAATTATCTTTACTTCACTTACAATGCTTCATCTCATGACATCACATTCGAT---GAACAAATGGTGATGGTGATTGGTTCAGGAGTATATCGAATTGGAAGTTCAGTTGAGTTTGATTGGTGTGCTGTTGGATGTTTAAGAGAACTTCGYAACCTTGGAAAGAAGACGATCATGGTGAATTACAATCCAGAAACTGTCTCTACTGATTATGATATGTGTGATCGACTTTATTTCGAAGAGATTTCATTCATATTATTAGTCAAGAAAGAGGAAAAAAAGAAACTTTTGGAGCTTTAGGAATAATTTATGCTATATTAGCTATTGGATTATTAGGTTTCGTTGTTTGAGCTCATCATATATTTACAGTAGGTATAGATGTTGATACTCGTGCTTATTTTACTTCTGCAACAATAATTATTGCAGTTCCAACAGGCATTAAAATTTTTAGTTGATTAGCCACAATCCATGGTACTCAAATAAATAACTCTCCCTCTCTATTATGAACTTTAGGTTTTATTTTTTTATTTACAGTAGGAGGATTAACAGGAGTTATTTTAGCAAACTCCTCTATTGATATTATTTTACACGATACCTATTATGTTGTAGCTCATTTTCATTATGTATTATCAATGGGAGCAGTATTCGCTATTATAGCCGGATTTGTTCACTGATACCCTCTATTTTCAGGTCTAATAATAAATGATAATTGATTAAAATCCCAATTTTATATTATATTTTTAGGAGTAAATTTAACATTTTTTCCCCAACATTTTTTAGGATTAGCTGGAATACCCCGACGATACTCCGATTATCCTGATGCCTATAGTTCTTGAAACATTATTTCTACCTTAGGCTCTACTATCTCTTTATTAGGAATTATTATATTTTTAATAATTATATGAGAAAGA

**Corynoneura sp. 1** -----------AGAACGGCCATGCACCACT--ACCCTTAATTTC-A-TGAAAGCGCTATCAA-GCTGTCAAACCCTATTAAGTTCTG-ACCTGGTAAGTTGTCCCGTGTTGAGTCAAATTAAGCCGCAACATCCACTGC-CGGTGATGATCTTCCGTCAATTCCTTTAAGTTTCAACTTTGCAACCATACTTCCCCCGGAAACT--AG-CTTT-GGTTTCCCGAAGAGCTACTGAATGCACCATGAAA--AGTAGTGACATCCAATCGCTAGCTGTCATCGTTTACAGTTAGAACTAGGGCGGTATCTAATCGCCTTCGATCCTCTAACTTTCGTTCTTGATTAATGAAAACATCCTTGGCAAATGCTTTCGCTTTAGTTAGTCTTGCAACGGTCTAAGAATTTCACCTCTCGCGCTGCAATACTAATGCCCCCAACTGCTTCTATTAATCATTACCTCT-TGATCTGGTATCAAACCAACAGAAAATCGACA--------------ACATTTTGCAACATTG-CCGAATAAGACCGAGGTCTTTTTCCATTATTCCATGCAAAAATATTCAAGGCATA-AGAGCCTGCTTTGAGCACCTTAATTTGTTCAAGGTAAAATTAAGCCGAACTAAATAGACACTTAGCCTAATGAAAGGCATCGGTGCTATTCATTTA--GTGTTCAGTCAAATAGTTCAAGTAA-TCGGAAATGATGGTA-GTCATGTACTTTATGACACCAC------ACCC-------G-TACTGAACAATAATCAACTTCGAACGTTTT-AAACGCAACAATTTTAATATACGCTAGTGGAGCTGGAATTACCGCGGCTGCTGGCACCAGACTTGCCCTCCACTTGATCCTCAACAAAGGATTTATACT-TGATTCATTCCAATTACAGAACATAGTTAACTAGTTCTATATTGTTATTTTTCGTCACTACCTCC-----TTCAATATTCTGTCAAAGGCAATGTGCACTTCTCGCTTAGTAGGACGCTGTGATCCATTAGAAATTTGCGCCAGCAATCGTAAGGCTGTCAATAGAGCGGCATGAAGTTCATTGGCGACTAACGCGGTCTTTT--GGCTGTGTCTATCGTTTGGTGAGCATGCCTGCTTCTGAA--ATAGCTGTCTGATGGATTTTAAGGATTGTATCATTGCATCACTCAAGATA-TCTGATCAACAGCGGTTAAATTTGGGATACCTTTTAGACCCGTCTTGAAACACGGACCAAGGAGTTTATCTAATACGCGAGTAAATGAGAAAACTTATTTGCGCAAATAAATTAACTGATGGGATTACGAGCGCTCCTCCATCCCGGAGTATTTTCATCAACATATGCT--TGCATATGGAAAATATATCATGAGCGTACTGGATATGACCCGAAAGATGGTGAACTATGCCTGATCAGGTTGAAGTCAGGGGAAACCCTGATGGAAGACCGAAGCAATTCTGACGTGCAAATCGCTGACTTATCCGCTCATTGGCAACTATGGTATACCCGGYGACGATGAGTACGACGTCGACGGTCTGATCAAGAAYTTTGAGTCGAACAACAAGATTTGGATATCGGGCTTGGTGGTGGGCGAGCTGTGTTCGACGCCATCGCATTGGCGTGTCAAGTACAAGCTGGCCGAGTGGATGAAGAAGCACAACATTGTGGGCATAAGTGGCGTTGACACCCGCGCACTCACTAAGCGTATTCGTGAGAATGGCACGATGCTCGGCAAGATCATACAGAAGTCGTGC------GGACCATTTGCTGGA---CTCGAATTCAAGGATCAGAATGAACGGAATCTCGTCGATGAGGTGTCGATCAAGAAACCGGTGACTTACAATGCGAARGGYGCGCCGAAGATCTGCGTCGTCGACTGTGGGTTAAAATTGAATCAAATTCGATGCCTGATCAAGCGGGGTGCGCGTGTCGATGTCGTGCCATGGGACTTTAAGCTCAAACCGGAGGATTACGATGGATTGTTCTTGTCGAATGGACCCGGCGATCCGGAAAAGTGTCAAAAAACCGTCCAAAATATCCAGARTGTGCTCAAGACGAAATTCACCAAACCGATTTTCGGCATCTGTCTCGGTCATCAACTTCTCGCAACGGCTGTGGGCTGTAAGACATACAAGTTGAAGTATGGCAATCGCGGCCACAATCTGCCGGCAATTCATCATGGCACAAATCGCTGCTTTATGACGTCGCAGAATCACGGCTTTGCTGTCGATGTGGCGACACTGGGCAGTGACTGGGAGCCGCTG------------------TTCTCGCGTGTCACAAAGACCATTGGCAGCTCGATGAAGAGTGTCGGCGAGGTGATGGCGATCGGCAGGAACTTTGAGGAAGCATTCCAGAAGGCATTGAGAATGGTCGACGAGACGGTGACTGGATTTGATCCGAATCTCAAGGATGTCGACGAAAGTGGCCTAAAAATTCCAACTGATAAGCGAATGTTTGTGCTCGCTGCTGCACTGAAGGCCGGCTACACTGTCGACAAGATCTACGATCTCACCAAAATCGATCGTTGGTTCCTGGCAAAGTTGAAGAACATCAATGACCTGACCATGGAGCTTGAGAAGATGAGT---GGAACGATCACAACGRCACAGTTGATGAGAGCCAAGAAGATTGGGTTTTCAGACAAACAGATTGCTGGTTGCATCAAA--ATCTGAACTCGCTGTGAGAAAGCAACGACGTGAAGCTGGAATTGTTCCATTTGTGAAACAAATCGACACAGTCGCCGGTGAATGGCCAGCATCGACCAACTATTTGTATTTGACTTACAACGCAGACTCACACGATGTGGAGTTCAAT---GATCAGATGATGATGGTAATCGGCTCCGGARTTTATCGCATTGGCAGCTCGGKWGAGTTYGACTG---------------------------------------------------------------------------------------------------------------------------------CATATTATTAGCCAAGAAAGAGGAAAAAAAGAAACCTTTGGAGCCCTAGGAATAATTTATGCTATATTAGCAATTGGTCTTTTAGGATTTATTGTTTGAGCTCACCACATATTTACTGTTGGAATAGATGTAGATACTCGTGCTTATTTTACTTCTGCTACTATAGTAATTGCAGTGCCCACAGGAATCAAAATTTTTAGTTGACTAGCTACACTTCATGGAACACAAATTAATAATTCACCTTCAATATTATGAGCACTAGGATTTGTATTTTTATTTACTGTAGGAGGATTAACTGGTGTGATTTTAGCTAACTCCTCAATTGATATTGTACTTCATGATACCTATTATGTTGTTGCTCATTTTCACTATGTTCTTTCTATAGGAGCGGTGTTTGCAATTATAGCAGGGTTTGTTCATTGATACTCATTATTCACAGGATTAATTTTAAATGAACAATGATTAAAATCTCAATTTTTCATTATATTCTTTGGAGTAAATTTAACATTTTTTCCTCAACACTTTTTAGGATTAGCCGGAATACCTCGACGATATTCAGACTACCCTGACGCTTTTACATCTTGAAACATTGTTTCATCTATCGGATCAACAATTTCTTTAGTAGGAATCCTTTATTTTATTTTTATTATCTGAGAAAGA

**Corynoneura sp. 2** TCCACGAACTAAGAACGGCCATGCACCACT--ACCCTTAATTTC-A-TGAAAGCGCTATCAA-GCTGTCAAACCCTATTAAGTTCTG-ACCTGGTAAGTTGTCCCGTGTTGAGTCAAATTAAGCCGCAACATCCACTGC-CGGTGATGATCTTCCGTCAATTCCTTTAAGTTTCAACTTTGCAACCATACTTCCCCCGGAAACT--AG-CTTT-GGTTTCCCGAAGAGCTACTGAATGCACCATGAAA--AGTAGTGACATCCAATCGCTAGCTGTCATCGTTTACAGTTAGAACTAGGGCGGTATCTAATCGCCTTCGATCCTCTAACTTTCGTTCTTGATTAATGAAAACATCCTTGGCAAATGCTTTCGCTTTAGTTAGTCTTGCAACGGTCTAAGAATTTCACCTCTCGCGCTGCAATACTAATGCCCCCAACTGCTTCTATTAATCATTACCTCT-TGATCTG-TATCAAACCAACAGAAAATCGACA--------------ACAGTTTGCACCACTG-CCGAATAAGACCGAGGTCTTTTTCCATTATTCCATGCAAAAATATTCAAGGCATA-AGAGCCTGCTT-----------------------------------------------------------------------------------------------------------------------------------------------------------------------------------------------------------------------------------------------------------------------------------------------------------------------------------------------------------------------------------TTCAATATTCTCTTAACAGCAATGTGCACTTCTTCCTTAGTAAGACGCTGTGATCCATTAGAAATTTGTNTCAACAATCATAAGGCTATCAAAAGAGCGGCGTGAAGTTCACTAGCGACTAATACAGTCTTTT--GGCTGTGTCTATCGTCTGGTGAGCACGCCTGCTTCTGAA--ATAGCTGTCTGATGGATTTTAAGGATTGTATCATTGCATCACTCAAGATA-TCTGATCGACAGCGGTTAAATTTGGGATACCTTTTAGACCCGTCTTGAAACACGGACCAAGGAGTTTATCTAATACGCGAGTAAATGAGAAAACTTATTTGCGCAAATAAATTAACTGATGGGATTACGAGCGCTCCTCCATCCCGGAGTATTTTCATCAACATATGCTCGGGTATATGGAAAATATATCATGAGCGTACTGGATATGACCCGAAAGATGGTGAACTATGCCTGATCAGGTTGAAGCCAGGGGAAACCCTGGTGGAAGACCGAAGCAATTCTGACGTGCAAATCGCTGACTTATCCACTCATTGGCAACTATGGAATACCCAACGACGATGAATTCGACATTCATGGTATCATCAAGAACTTTGAGTCGAACAATAAAATTTGGATATCTGGTCTGATCGTGGGTGAACTCTGTGAGACGCCTTCGCATTGGCGTGTCAAGTATAAGCTTGCCGAGTGGATGAAGAAGCACAATGTCGTCGGTATTAGTGGCGTTGACACGCGCTCCCTTACCAAGAAAATTCGCGAGAATGGAACGGTACTCGGCAAGATCATTCAAAAATCATGT------GGACCTTTCATTGGA---CTCGAATTCAAAGATCAGAATGAGCGCAATCTTGTCGATGAAGTTTCAATCAAGAAACCGATGACTTATAATTCTACAGGAGCGCCGAAGATTTGCGTCGTCGACTGTGGATTGAAGTTGAATCAGATTCGATGTTTGATCAAACGCGGAGCTCGTGTCGATGTTGTGCCATGGGACTTCAAGTTGAAGCCCGAAAATTATGATGGGTTGTTCCTGTCAAATGGTCCCGGTGATCCAGCGATATGCCACAAAACCGTTGAAAATATCCGGAATGTGCTCAAGACGAAATTCACTAAACCAATTTTCGGCATTTGTCTCGGCCATCAGCTTCTCGCCAGCGCAGTAGGCTGTAAGACCTACAAAATGAAGTACGGAAATCGCGGTCACAATCTACCTGCGATGCATCACGGCACAAAGCGCTGTTTCATGACATCGCAAAATCACGGCTTTGCTGTCGATGTGGCAACTTTGGGCGAAGATTGGGAGCCTTTGTTCACAAATTTGAACGATTTCGCACGTGTCACAAAAACCATCGGCAGTTCAATGAAGAGTGTCGGTGAAGTTATGGCGATTGGCCGGAATTTTGAGGAAGCATTTCAGAAAGCTTTGAGAATGGTCGATGAAACGGTAAATGGATTTGATCCTGATCTTAAGCCGGTCGATGAGGAAGGACTAAAGACGCCAACTGATAAACGAATGTTCGTTCTTGCTGCTGCACTTAAGGCGGGCTACACCGTCGATAAAATTTATGAGCTCACAAAAATCGATTGTTGGTTCCTGGCGAAATTGAAGAATATCATCGACTTGACCATGGATCTGGAAAAGATGAGT---GGAACGATCACAAACTCTCAGTTGATGAGGGCTAAGAAAATTGGTTTCTCCGACAAACAAATTGCGGGTTGTATCAAA--CTCAGAGCTTGCAGTAAGAAAGCAGAGACGTGAAGCAGGAATTGTGCCATTTGTGAAGCAAATCGACACTGTCGCAGGTGAATGGCCGGCATCGACAAATTATTTGTACTTGACATACAATGCCAACTCGCACGACATCGAGTTCAAT---GATCAAATGGTTATGGTGATTGGCTCGGGAGTTTATCGAATTGGAAGTTCGGTTGAGTTTGACTGGTGTGCAGTTGGCTGCTTGAGAGAATTGAGAAATCTTGGCAAACAGACAATTATGGTGAATTACAATCCTGAGACGGTGTCAACAGATTATGACATGTGCGACAGATTGTATTTTGAGGAGATTTCATTCATATTATTAGCCAAGAAAGAGGAAAAAAAGAAACATTTGGTGCTTTAGGAATAATTTACGCAATACTTGCAATTGGTTTATTAGGATTTATTGTTTGAGCTCATCATATATTTACAGTCGGAATAGACGTAGATACACGAGCTTATTTTACTTCAGCTACAATAGTAATTGCTGTACCTACAGGAATTAAAATTTTTAGATGACTAGCTACTTTACATGGAACTCAAATAAATAATTCTCCTTCTATACTTTGAGCATTAGGTTTTGTATTTTTATTTACTGTAGGAGGATTAACAGGTGTTATTTTAGCAAATTCTTCAATTGATATTGTACTTCATGATACTTATTATGTTGTTGCACATTTTCATTATGTTTTATCAATAGGAGCAGTATTTGCAATTATAGCAGGCTTTGTTCATTGATATACTTTATTTTCTGGTTTAATATTGAATGAACAATGACTAAAATCTCAATTTTTTATTATATTTTTTGGAGTAAATTTAACCTTTTTTCCCCAACATTTTTTAGGATTATCAGGAATACCTCGTCGTTATTCAGATTTTCCAGATGCTTTTACTTCTTGAAATATTATTTCATCAATTGGATCTACCATTTCATTAATAGGAATTTTATATTTTATTTTTATTATTTGAGAAAGT

**Cricotopus sp. 1** ---------------CGGCCATGCACCACT--ACCCTTAATTTA-G-CGAAAGCGCTATCAA-ACTGTCGTACCCTATTAAGTTCGG-ACCTGGTAAGTTTTCCCGTGTTGAGTCAAATTAAGCCGCAAACTCCAAATC--TTGTGTCAAATTCCGTCAATTCCTTTAAGTTTCAACTTTGCAACCATACTTCCCCCGGAAACT--AG-CTTT-GGTTTCCCTGAAAGCTACTGAATGCACCATATAA--AGTAGTGACATCCAATTGCTAGCTGTCATCGTTTACAGTTAGAACTAGGGCGGTATCTAATCGCCTTCGATCCTCTAACTTTCGTTCTTGATTAATGAAAACATCCTTGGCAAATNNNNNNNNNNNNNNNNNNNNNNNNNNNNNNNNNNNNNNNNNNNNNNNNNNNNNNNNNNNNNNNNNNNNNNNNNNNNNNNNNNNNNNNNNNNNNNN----NNNNNNNNNNNNNNNNNNNNNNNNNNNNNNNNNNNNNNNNNNNNNNNNNNNNNNNNNNN-NNNN-TAAAACCGAGGTCTTGTTCCATTATTCCATGCAAAAATATTCAAGGCATT-AGAGCCTGCTTTGAGCACCTTAATTTGTTCAAGGTAAAATTAAGCCGAATTAAATAGACACTTAGCCTAGTGAAAGGCATCGGTGCTATTCATTAA--GTGTTCAGTCATGTAGTTCAAGCAA-TCGAAAGTGATAGCA-GCCATTTACTTGATGGCGCCAC------ACTC-------G-TGCTGAACAAAAATGAACTTCGAACGTTTT-AACCGCAACAATTTTAACATACGCTAGTGGAGCTGGAATTACCGCGGCTGCTGGCACCAGACTTGCCCTCCACTTGATCCTCATTGAAGGATTTATACT-CGATTCATTCCAATTACAGAAC-TAAATAATTAGTTCTATATTGTTATTTTTCGTCACTACCTCCC----TTCAATATTTTGGTAACAACAATGTGCACTTCTCATTTAGTAAGACGTTGTGATCCATTAGAAATTTGTATCAACAATTATGAGGTTATCAAAAGGCTGATGTAAAGTTCATCAATAAATGACTTGATCTTTGATGGTTGGGTGTATTATATGGTGAGTATATCATGTTCTGC-TTATAACTGTCTGATGGATTTTAAAGGCTATAATATTGCATCACCCTGATTA-CCCGATCAACAACGGTTAAATTTGGGATACCTTTTGGACCCGTCTTGAAACACGGACCAAGGAGTCTATCTATTGCGCGAGTAAATGGGGAAACCTATTTGCGTAAAAAACTTGACTGATGGGATTACAAGT-CTTCTCCATCCCGGGGTATTTTTATCAACACATACAAATGTATGTGGAAAATATACCATGAGCGTAGTGGATATGACCCGAAAGATGGTGAACTATGCCTGATCAGGTTGAAGTCAGGGGAAACCCTGATGGAGGACCGAAGCAATTCTGACGTGCAAATCG------------------------------------------------------------------------------------------------------------------------------------------------TGGCGTCAAAAGTACAAACTCGCTGAATGGATGAAGAAGCACAATGTGGCTGGAATTAGCGGAATCGACACTCGCGCTTTGACGAAAAAAATTCGAGAGAACGGAACGATTTTGGGAAAAATTATTCAACAATCCGCT------GGACCTTTTCCTGAA---CTCGAATTTAAGGACCAAAATCAAAGGAATTTGGTTGACGAAGTTTCCATAAAGAAATCGATCACGTACAACGCCACTGGATCGCCTCGCATTTGTGCTGTCGATTGTGGCTTAAAGTTGAATCAAATCCGCTGCTTTGTGAAACGCGGKGCAAGAGTTGATGTCGTGCCTTGGGATCATGCTCTAAACGTAAAAGATTTTGACGGCCTCTTCCTCTCCAACGGCCCAGGTGATCCAGTTATGTGTGCCAAAACTGTTGCGAACATTCAAAAAGTTTTGTCATCTTCACAACTCAAACCGATTTTCGGTATTTGTCTTGGTCACCAGCTGCTCGCTACCGCAGTCGGTTGCAAGACTTATAAAATGAAATACGGCAATCGTGACCATAATCTACCAGCG---------------------------------------------------------------------------------------------------------------TTCATCAGAGTCAGTAAGAACATCGGAAGTTCTATGAAAAGTGTCGGCGAAGTCATGGCAATCGGAAGAAATTTCGAAGAAGCTTTCCAGAAAGCGCTGCGAATGGTCGATGAAAATGTCAACGGATTCGATCCAAATCTGAAGCCGGTGGTGGATGAAGAACTCAAGACACCGACGGATAAACGAATTTTCGTTCTAGCTGCTGCGTTGAAAGCTGGCTACACGGTGGATCGTCTCTACGATYTAACAAAAATCGACCGATGGTTCCTTGAAAAAATGAAGAACATCATCAATGTCACGTTGGAGCTGGAAAARCTCAAT---TGCATCGTTTCTGATGAGCTGCTCAGACAATCGAAAAAGTTTGGATTTTCTGACAAGCAAATTGCCAAATACATCAAA--ATCAGAACTTGCCGTGAGAAAGCAGCGACGTGAGAGCGGAATTCTTCCTTTTGTCAAGCAAATCGACACTGTCGCTGGTGAATGGCCAGCATCAACCAACTATCTCTATTTGACGTACAACGCATCAGCTCATGACATCGACTTCACT---GAACCGATGGTGATGGTGATYGGATCTGGAGTTTATCGCATTGGAAGTTCGGTGGAGTTTGATTGGTGCGCTGTTGGATGTTTGCGGGAGYTGAGGAATCTTGGCAAAAAGACAATCATGGTGAACTACAATCCTGAAACGGTTTCAACTGATTACGACATGTGCGATCGTTTATACTTTGAGGAAATATCATTCATATTATTAGTCAAGAAAGAGGAAAAAAAGAAACCTTCGGTTCTTTAGGTATAATTTATGCTATATTAGCAATTGGGTTATTAGGATTTGTTGTTTGAGCACATCATATATTTACAGTAGGTATAGACGTAGATACACGAGCTTATTTTACTTCGGCAACAATAATTATTGCTGTTCCAACTGGAATTAAAATTTTTAGTTGACTTGCTACATTACATGGAACCCAATTAAATAATTCACCTTCTTTACTTTGAGCTCTTGGATTTGTTTTTTTATTTACAGTTGGTGGATTGACTGGTGTTGTTCTAGCAAATTCTTCTATTGATATTGTTTTACATGACACATATTATGTAGTAGCTCATTTTCATTATGTATTATCAATAGGCGCAGTATTTGCTATTATAGCTGGATTTGTCCACTGATACACATTATTTACAGGCTTAACTATAAACGAAAAATGATTAAAATCTCAATTTTCTATTATATTTTTAGGAGTTAATTTAACATTTTTCCCTCAACATTTTTTAGGATTAGCAGGTATGCCTCGACGATATTCTGATTATCCTGACGCATATACTTCATGAAATATCGTGTCAACAATTGGATCAACAATTTCTTTATTCGGAATTTTATTTTTTCTTTTTATTATTTGAGAAAGA

**Cricotopus sp. 2** ------------------CCATGCACCACT--ACCCTTAATTTT-AGCGAAAGCGCTATCAA-ACTGTCGTACCCTATTAAGTTCGG-ACCTGGTAAGTTTTCCCGTGTTGAGTCAAATTAAGCCGCAAATTCCAAATC--TGGTGTCGAATTCCGTCAATTCCTTTAAGTTTCAACTTTGCAACCATACTTCCCCCGGAAACT--AG-CTTT-GGTTTCCCTGAAAGCTACTGAATGCACCATATAA--AGTAGTGACATCCAATTGCTAGCTGTCATCGTTTACAGTTAGAACTAGGGCGGTATCTAATCGCCTTCGATCCTCTAACTTTCGTTCTTGATTAATGAAAACATCCTTGGCAAATGCTTTCGCTTTAGTTAGTCTTGCGACGGTCTAAGAATTTCACCTCTCGCGCCGCAATACTAATGCCCCCGACTGCTTCTATTAATCATTACCTCT-TGATCTG-TATCAAACCAATAGAAAG-CGATA----------CTAACACCGAAGCGCCAGCA-CCTA-TAAGACCGAGGTCTTTTTCCATTATTCCATGCAAAAATATTCAAGGCATT-AGAGCCTGCTTTGAGCACCTTAATTTGTTCAAGGTAAAATTAAGCCGAACTAAATAGACACTTAGCCTAGTGAAAGGCATCGGTGCTATTCATTAA--GTGTTCAGTCATGTAGTTCAAGCAA-TCGAAAGTGATGGCA-GCCATTTACTTGATGGCGCCAC------ACTC-------G-TGCTGAACAAAAATGAACTTCGAACGTTTT-AACCGCAACAATTTTAACATACGCTAGTGGAGCTGGAATTACCGCGGCTGCTGGCACCAGACTTGCCCTCCACTTGATCCTCATTGAAGGATTTATACT-CGATTCATTCCAATTACAGAAC-TAAATAATTAGTTCTATATTGTTATTTTTCGTCACTACCTCCC----TTCAATACTTTGGCAACAACAGTGTGCACTTCTCGCTTAGTAAGACGTTGTGATCCATTAGAAATTTATTCCAATAGCTATGAGGTTATCAATAGGCTGGTGTAAAGTTCATCAGTAAATGTCTAGATCTTTAATGGTCGAGTGTATTATATGTTGAGTGCATCATG-TCTGC-CTATAACTGTCTGATGGATTTTAAAGATCATAACATTGCATCACCCAGGTTAATCCGATCAACAACGGTTAAATTTGGGATACCTTTTGGACCCGTCTTGAAACACGGACCAAGGAGTCTATCTATTGCGCGAGTAAATGGGGAAACCTATTTGCGGAAAAAACTTGACTGATGGGATTACGAGT-CTCCTCCATCCCGGGGTATTTTCATCAACACATGCAAATGTATGTGGAAAATATACCATGAGCGTAGTGGATATGACCCGAAAGATGGTGAACTATGCCTGATCAGGTTGAAGTCAGGGGAAACCCTGATGGAGGACCGAAGCAATTCTGACGTGCAAATCGCTTACTTATCCGCTGATTGGTAACTATGGAATTCCTAGTGATGAAGAATTCGATGCAAACAAGATAATAAAACACTTTGAGTCTGANAATAANATTTGGGTGTCAGGACTTGTTGTCGGGGAATTATGCNAAACACCTTCNCACTGGCGTCNGAAGTATAAGCTTGCCGAATGGATGAAAAAGCACAACGTGGTCGGTATCAGTGGAATTGACACGCGTGCTTTAACTAAGAAAATTCGGGAAAATGGGACGATTTTAGGAAAAATAATTCAGCAGTCAAGC------GGACCTTTCCCTGGT---CTTGAGTTTAAGGATCAAAATCAGCGGAATCTTGTAGATGAAGTTTCTATAAAAAAATCGATAACTTACAACGCTTCCGGTTCTCCAAGAATATGTGCCGTTGATTGCGGATTGAAGCTAAATCAAATTCGGTGCTTTGTTAATCGCGGTGCTAGGGTTGATTTAGTACCATGGGATCATGATCTGGATCCGAGAGAATTCGACGGACTCTTTCTCTCAAATGGCCCAGGTGATCCAGTCATGTGCGAAAAAACCGTTAACAATATTCAAAAAGTTCTTGAATCTCCACAACTAAAACCAATATTTGGCATTTGCCTCGGTCACCAACTCTTGGCTACATCAGTTGGCTGCAAGACTTACAAAATGAAATATGGCAACCGTGGCCATAACTTACCGGCTCTTCATCATGGTACTAATCGCTGCTTTATGACATCTCAAAACCACGGATTCGCTGTTGATGCTAATACCATCGCAAAGGACTGGGAACCTCTTTTCACAAATCTCAATGACTTCATTAGAGTCAGTAAAAATATTGGTAGCTCAATGAAAAGTGTCGGCGAAGTTATGGCAATCGGAAGAAATTTCGAAGAAGCCTTTCAAAAAGCTTTGCGAATGGTAGACGAAAATGTTAACGGTTTTGACCCAAATCTGAAGCTGGTAAATGACGAAGAACTTAAAACACCAACCGACAAAAGAATTTTTGTACTTGCTGCTGCTTTAAAGGCTGGATATACTGTCGACCGCCTCTACGATTTGACTAAAATAGATCGATGGTTCCTTGAAAAAATGAAAAATATCATAAATATCACTTTGTGCCTAGAAAAATTGAAC---TGTGTTGTATCAGATGATCTTCTCCGGCAGTCGAAGAAAATGGGATTCTCAGACAAGCAAATAGCAAAGTATATTAAA--ATCAGAACTTGCTGTAAGAAAGCAACGACGGGAAAGTGGAATCCTACCATTCGTGAAGCAAATTGACACGGTCGCTGGAGAGTGGCCAGCCTCAACCAATTATCTTTATATGACTTATAATGCATCTGCACATGACGTTGACTTTAAC---GAGCAAATGGTAATGGTTATAGGATCAGGAGTCTATCGCATTGGTAGTTCAGTTGAGTTTGATTGGTGTGCTGTTGGATGTTTGAGAGAATTGAGAAAATTAGGCAAGAAAACAATTATGGTGAACTACAACCCTGAAACTGTATCAACTGATTACGACATGTGCGACCGCTTGTACTTTGAAGAAATATCTTTCACATTATTAGTCAAGAAAGAGGAAAGAAGGAAACATTTGGGTCTTTAGGAATAATCTATGCTATGCTAGCAATTGGTTTATTAGGATTTGTTGTTTGAGCTCACCATATATTTACAGTAGGTATGGATGTAGATACTCGAGCTTACTTTACTTCAGCAACTATAATTATTGCTGTTCCTACAGGAATTAAAATTTTTAGTTGACTAGCTACTCTACATGGAGCTCAATTAAATAATTCTCCATCCTTATTATGAGCTTTAGGATTTGTGTTCTTATTTACTGTAGGAGGTCTGACAGGTGTTGTTTTAGCTAATTCTTCAATTGATATTGTACTACATGACACTTATTATGTAGTTGCTCATTTTCATTACGTATTATCAATAGGAGCAGTATTTGCTATTATAGCTGGATTTGTACATTGATATCCTTTATTCACAGGATTAACTATAAATGAAAAATGATTAAAGTCTCAATTTGCTATTATATTCTTAGGAGTTAATTTAACATTTTTCCCTCAACATTTCTTAGGGTTAGCTGGAATACCTCGACGATATTCAGATTACCCTGACGCTTACACGTCCTGAAACATTGTATCAACTATTGGTTCAACTATTTCATTATTTGGTATTTTATTCTTCTTATTTATTATTTGAGAAAGT

**Diamesa sp.** --------CTAAGAACGGCCATGCACCACT--ACCCTTAATTTC-G-AGAAAGAGCTATTAA-TCTGTCTTACCCTATTAAGTTCGG-ACCTGGTAAGTTTTCCCGTGTTGAGTCAAATTAAGCCGCAAAATCCACGCC-TTGTGGTGCTCTTCCGTCAATTCCTTTAAGTTTCAACTTTGCAACCATACTTCCCCCGGAAACT--AG-CTGT-GGTTTCCCGGA-AGCTACTGAATGCGCCAT-AAT--AGTAGCGACATCCAATTGCTGGCTGTCATAGTTTACAGTTAGAACTAGGGCGGTATCTAATCGCCTTCGATCCTCTAACTTTCGTTCTTGATTAATGAAAACATCCTTGGCAAATGCTTTCGCTTTAGTTAGTCTTACGACGGTCTAAGAATTTCACCTCTCGCGCCGTAATACTAATGCCCCCAACTGCTTCTATTAATCATTACCTCT-TGATCTG-TATCAAACCAATAGAAACTAAATA---------------------------------TA-TAAGACCGAGGTCTTTTTCCATTATTCCATGCAAAAATATTCAAGGCATATAAAGCCTGCTTTGAGCACCTTAATTTGTTCAAGGTAATAGTAAGCTGAACTAAATAGACG-ATAACCTAATTAAAGGCGTCT-CGCTATTCATTAATAGTGTTCAGTCATATAGTCCAAGTAA-TCGGAAATGACTGCA-ACCA-TAACTTGATGGTGCAGC------ACCC-------G-TACTGGACAATAATCAACTTCGAACGTTTT-AACCGCAACAATTTTAATATACGCTAGTGGAGCTGGAATTACCGCGGCTGCTGGCACCAGACTTGCCCTCCACTAGATCCTTGTTAAAGGATTTATAAT-TAACTCATTCCAATTACAGAACATAGTTAACTAGTTCTATATTGTTATTTTTCGTCACTACCTCC-----TTCAATATTCGGTTAAACGCAATGTGCACTTTTCTCTTAGTAAGACGTTGTGATCCATTAGGAATTAGTGTCAACTATTATTGGGCTACCAATAGACCTGTATGAAGTTTATCAGCAAATAATCCGATCGTTTATGGTTGTATATATTGTCTGGTAAGCGTATGGTGTTCTG--GTGTAGCTGCCTGATGGATTTTAAAGGTCACATCATTGCATTACCCATGATAACCCGATCAACAGCGGTCAAATTTGGGATACCTTTAAGACCCGTCTTGAAACACGGACCAAGGAGTCTATCTAGTATGCAAGTAAATGGG-AAACCTATTTGCGTAAATAACTTGACTAATGGGATTACGAATGTTCCTCCATCCCGGGGTATTTTTATCAACATATGCT--TGCATATGGAAAATATACCATGAGCATACTGGATATGACCCGAAAGATGGTGAACTATGCCTGATCAGGTTGAAGTCAGGGGAAACCCTGATGGAGGACCGAAGCAATTCTGACGTGCAAATCGTTGACTTACCCATTGATTGGAAACTATGGAATTCCT---GATGAAGATGTAGACGAAAACAASATTTCGAAACATTTTGAATCTAACAATAAGATCTGGATATCGGCTYTAGTAGTAGGAGAGCTTTGTGAAACTCCATCTCACTGGCGTCAAAAATATAAATTRTCTGAATGGATGAAAAAGCATAACATCCCTGGTATCAGTGGCATTGATACACGAGCTCTRACAAAGCTGATCCGAGATAATGGAACAGTATTGGGAAAGATCATTCAACAACAATCT------GGTCCTTTTCCAAGC---TTGAAATTCAATGACCAAAATGAACGTAACCTTGTGAATGAAGTTTCAGTGAAAAAAACTATTATTTACAATGCTTCTGGATCTCCCAGGATTTGTGTTGTAGACTGTGGGTTGAAGTTGAATCAGCTTCGCTGCTTCATTAAACGTGGTGCTCGTGTGGACGTCGTTCCGTGGGACCACGAGTTGAATTGTAAAGACTTTGATGGATTATTTTTGAGTAATGGTCCAGGCGATCCTGTAATGTGCCAAAAAACTGTTMAAAACATTCAAAGTGTTTTAAACTCACCTAACGTAAAACCAATATTTGGAATTTGTTTGGGTCATCAACTACTCTCTTCTGCTATTGGGTGCAAAACGTACAAGCTGAAGTATGGTAACCGTGGTCATAATTTGCCTTGTATCCATCATGGAACAAACAGGTGCTTCATGACTTCCCAAAATCATGGATTTGCTGTTGATGTGGAAAAGATGAATCCTGAGTGGGAACCTCTTTTCACTAAT---------------------------------------------------------GAAGTTATGGCAATTGGTAGAAAGTTCGAAGAAGCATTCCAGAAAGCCTTGCGTATGGTTGATGAAAGTATTAATGGTTTTGATCCCAACGTAAAACTAGTAAATGATGATGAACTAAGTACTCCAACAGACAAAAGAATGTTTGTTTTAGCTGCTGCTCTGAAAGCTGGTTATACAGTCGATAAATTGTATGACTTAACAAAAATTGACCGTTGGTTCTTGGATAAAATGAAGAACATCATTGATCTCACTTTGCARTTAGAAATGTTAGAT---TGCAAGCTTCCAGTTGAGTTATTGAAAACAGCTAAGAAAATGGGTTTCTCAGACAAACAGATTGCTACATTTATCAAA--TTCCGAGCTAGCAGTAAGAAAACAACGTCGCGAGAATAAAATAATTTCGTTTGTTAAGCAAATTGATACTGTTGCTGGAGAATGGCCAGCTTCCACAAATTATTTATACCTGACATACAACGCTTCTTGTCATGATGTTAAATTTGAY---GATGAATTTATTATGGTCATTGGCTCAGGAGTATATCGAATAGGGAGCTCTGTGGAATTTGATTGGTGTGCCTGTGGATGTCTAAGAGAGTTAAGAAATCTTGGCAAAAAAACAATCATGGTGAACTATAATCCAGAAACA-----------------------------------------------------CATATTATTAGTCAAGAAAGTGGAAAGAAGGAAACATTCGGAGCACTTGGAATAATCTACGCTATATTAGCTATTGGATTATTAGGATTTGTCGTTTGAGCTCATCATATATTTACAGTAGGAATAGACGTGGATACTCGAGCTTATTTTACATCTGCAACTATGATTATTGCGGTACCTACTGGTATTAAAATTTTTAGTTGATTAGCTACTCTTCACGGAACACAAATAAACTATTCTCCTTCTCTACTTTGAGCTTTAGGATTTGTATTTTTATTTACTGTAGGTGGATTAACTGGAGTAGTTTTAGCTAATTCTTCTATTGATATTGTTCTTCATGATACGTATTACGTTGTTGCTCATTTTCATTATGTACTTTCTATAGGAGCTGTATTTGCTATTATAGCAGGATTTGTCCATTGATACCCTCTATTTACAGGTTTAACCTTAAATGAAGAATGATTAAAGTCTCAATTTGTTATTATATTTTTAGGTGTAAATTTAACTTTTTTTCCGCAACATTTTTTAGGGTTAGCTGGAATACCTCGACGATACTCTGATTATCCAGATGCTTATACTTCTTGAAATGTAGTTTCTACAATTGGGTCTACTATTTCTTTATTTGGAATTTTATTTTTCTTATTTATTATTTGAGAAAGT

**Echinocladius martini** ------------------CCATGCACCACT--ACCCTTAATTTC-A-AGAAAACGCTATCAA-GTTGTCATACCCTATTAAGTTCGG-ACCTGGTAAGTTTTCCCGTGTTGAGTCAAATTAAGCCGCAAACTCCAAACC--TGATGTAGGCTTCCGTCAATTCCTTTAAGTTTCAACTTTGCAACCATACTTCCCCCAGAAACT-AGCTTAAT-GGTTTCCCGGA-AGCTACTGAATGCACCATGAAA--AGTAGTGACATCCAATTGCTAGCTGTCATAGTTTACAGTTAGAACTAGGGCGGTATCTAATCGCCTTCGATCCTCTAACTTTCGTTCTTGATTAATGAAAACATCCTTGGCAAATGCTTTCGCTTTTGTTAGTCTTGCGACGGTCTAAGAATTTCACCTCTCGCGCCGCAATACCAATGCCCCCAACTGCTTCTATTAATCATTACCTCT-TGATCTG-TTTCAAACCAATAGAAAG-CGACT-----------GCACGTTTTAACGCGCACAGCCTA-TAAGACCGAGGTCTTTTTCCATTATTCCATGCAAAAATATTCAAGGCATA-AGAGCCTGCTTTGAGCACCTTAATTTGTTCAAGGTAAAAGTAAGCCGAACTAAATAGACATCTAGCCTAATAAAAGGTATCGATGCTATTCATTAA--GTGTTCAGTCATATAGTTCAAGTAA-TCGGAAATGATAGCA-GCCATTTACTTGATGGCGCTAC------ACCC-------G-TACTGAACAAAAATCAACTTCGAACGTTTT-AACCGCAACAATTTTAATATACGCTAGTGGAGCTGGAATTACCGCGGCTGCTGGCACCAGACTTGCCCTCCACTTGATCCTCATTAAAGGATTTATACT-TGATTCATTCCAATTACAGAAC-TAAATAATTAGTTCTATATTGTTATTTTTCGTCACTACCTCC-----TTCAATATTCCTTTAAAAGCAATGTGCACTTCTTCCTTAGTAAGACGCTGTGATCCATTAGAAATTCATTCCAATGATTATTTGGCTATCAATGGGCCGGTATGAAGTTCATTAGCAAACAACTTGATCTTTAATGGTTGGGTATATTGTTTGGTGAGCATGCCGTAACCTGTATTATAGCTGTCTGATGGATTTTAAAGGTCATATTATTGCATCACTCAGAATAACCCGATCAACAGTGGTTAAATTTGGGATACCTTTTGGACCCGTCTTGAAACACGGACCAAGGAGTCTATCTAGTATGCAAGTAAATGAGGAAACTTATTTGCAGAAAAAACTTGACTGATGGGACTACGAGTGCTCCTCCATCCCGGGGTATTTTCATCAACATATGCAAATGTATATGGAAAATATACCATGAGCATACTGGATATGACCCGAAAGATGGTGAACTATGCCTGATCAGGTTGAAGTCAGGGGAAACCCTGATGGAGGACCGAAGCAATTCTGACGTGCAAATCGCTCACTTATCCATTAATTGGAAATTATGGAATCCCGAGTGACGAAGAATTCGATGAACACAAGATTATGAAGCATTTTGAATCGAACAACAAGATTTGGGTCTCGGGATTAGTGGTGGGCGAATTGTGTGAGACACCATCGCACTGGCGTCAAAAATACAAATTGGCTGAATGGATGAAGAAGCATAATGTGGTTGGAATCAGCGGCATAGACACGCGCGCTTTGACCAAAAAGATTCGCGAGAACGGAACTGTCTTGGGTAAAATCATTCAGCAGTCTTCG------GGTCCATTTCCAGGA---TTGGAATTCAAAGACCAGAATCAACGAAATCTGGTTGATGAGGTGTCAATCAAGAAGAAAGTTATTTTCAATGCAAAAGGCTCTCCAAGAATCTGTGCCATTGACTGCGGCTTGAAGTCAAATCAAATTCGTTGCTTTGTTCAAAGAGGAGCTCGCGTTGATGTTGTGCCATGGGATCATCCAATAAATTCTGATGATTTTGACGGATTATTCCTCAGCAATGGACCCGGCGACCCGGTTATGTGCAAGAAAACTGTTGCAAATCTGCAAAAATTCCTCTCGTCCCAGAAAGTCAAACCAATTTTTGGTATTTGCTTGGGTCATCAACTGCTCTCAACTGCAATCGGCTGTAAAACTTATAAATTGAAGTACGGAAATCGAGGACACAATCTACCAGCTCTCCATCATGGCACCAATCGATGTTTCATGACGTCTCAAAATCATGGATTCGCTGTTGACGTAAGCACGATTGCCAAGGATTGGGAACCTCTTTTCACCAATTTGAATGACTTCATTCGTGTCAGCAAACACATTGGCAGCTCTATGAAGAGCGTCGGCGAAGTAATGGCTATTGGACGAAAATTCGAGGAGGCTTTCCAGAAAGCTCTTCGTATGGTTGATGAGAACGTTGATGGATTCAATCCAAACTTGAAACCAGTAAGCGACGAAGAGTTGCAGACTCCCACGGATAAAAGAATGTTTGTTTTGGCTGCTGCACTTAAAGCTGGCTACAGCGTTGACAGGCTTTACGATTTAACCAAAATCGATCGCTGGTTTCTTGACAAGATGAAAAACATCAACGCAATCACGCTAGAACTTGAGCAATTAAAT---TGTGCCATCTCTGATGAACTCTTGGGACGAGCAAAGAAATTTGGATTTTCAGATAAACAAATTGCAAACTTAATCAAA--CTCCGAATTGGCTGTGAGAAAACAACGACGCGAAGGTGGAATTTTACCATTTGTGAAGCAAATTGACACCGTCGCAGGTGAATGGCCGGCTTCCACAAATTATCTTTATTTAACGTATAATGCGAGCACACATGATATTGAATTCAAT---GATGAAATGGTCATGGTGATCGGATCTGGTGTTTATCGAATTGGAAGTTCAGTTGAGTTTGACTGGTGTGCTGTAGGTTGTTTGAGAGAATTGAGAAATTTGGGAAAGAAGACGATCATGGTGAATTACAATCCGGAAACAGTTTCAACTGATTACGATATGTGCGATCGTTTGTATTTTGAAGAAATTTCATTCACATTATTAGACAAGAAAGAGGAAAAAAAGAAACTTTTGGGGCTTTAGGAATAATCTATGCTATATTAGCTATTGGACTTTTAGGTTTTATTGTTTGAGCTCACCACATATTTACAGTTGGAATAGACGTTGACACACGAGCTTATTTTACGTCTGCAACTATAATTATTGCTGTACCAACCGGAATTAAAATTTTTAGATGATTAGCAACATTACATGGTACCCCATTAAATAATTCTCCTTCTTTACTATGAGCTTTAGGATTTGTTTTTTTATTTACAGTTGGTGGTTTAACAGGAGTAATTTTAGCAAATTCTTCTATTGATATTGTATTACACGATACCTATTATGTAGTTGCTCACTTTCACTATGTTTTATCAATAGGAGCCGTGTTTGCTATCATAGCTGGATTTGTCCACTGATATTCTCTATTTACTGGACTTTCAATAAATAAAACTTGATTAAAATCTCAATTTTCTATTATATTTTTAGGAGTAAATCTAACATTTTTTCCACAACACTTTTTAGGCTTAGCAGGTATACCTCGGCGATATTCAGATTATCCAGATTCCTATACATCTTGAAATATTATTTCTACTGTTGGTTCTACAATTTCATTAATTGGAATCTTATTTTTTATTTTTATTATTTGAGAAAGA

**Elpiscladius capicola** ------------------------------------------------------------------------CCCTATTAAGTTCGG-ACCTGGTA-GTTTCCCCGTGTTGAGTCAAATTAAGCCGCAACATCCACTTC-CGGTGATGCTCTTCCGTCAATTCCTTTAAGTTTCAACTTTGCAACTATACTTCCCCCAGAAACT--AG-CTTT-AGTTTCCCGAAAAGCTACTGAATGCACCATATTTTTAGTAGTGACATCCAATTGCTAGCTGTCATCGTTTACAGTTAGAACTAGGGCGGTATCTAATCGCCTTCGATCCTCTAACTTTCGTTCTTGATTAATGAAAACATCCTTGGCAAATGCTTTCGCTTTTGTTAGTCTTACGACGGTCTAAGAATTTCACCTCTCGCGCCGTAATACTAATGCCCCCAACTGCTTCTATTAATCATTACCTCT-TGATCTG-TATCAAACCAATAGAAAATCGATG------CCACAGCCGCAAGCGGCGGACACC-TTAA-TCAGACCGAGGTCTTTTTCCATTATTCCATGCAAAAATATACAAGGCATATAGAGCCTGCTTTGAGCACCTTAATTTGTTCAAGGTAAAAGTAAGCCGAACTAAATAGACGGCTAGCCTAGTGAAAGGCATCACCGCTATTCATCAA--GTGTTCAGTCATATAGTCCAAGTCA-GCGGAAATGATGGCA-ACCGTTTTCATGACGGTGCCTC------ACCC-------G-TACTGGACAATAATCAACTTCGAACGTTTT-AACCGCAACAATTTTAATATACGCTAGTGGAGCTGGAATTACCGCGGCTGCTGGCACCAGACTTGCCCTCCACTTGATCCTCACTAAAGGATTTATACT-TGATTCATTCCAATTACAGAACATAGTTAACTAGTTCTATATTGTTATTTTTCGTCACTACCTCC-----TTCAATATTCTGTCAAAGGCAATGTGCACTTTTCCTTTAGTAAGACGCTGTGATCCATTAGAAATTCGTTCCAACGGTTAATGGGCCATCAATAGGCCGACACGAAGTTCATCGGCGAATGTGTGGCTCTTCAATGGGTCGCTGTATCGCTTGGTGAGCGTGCCGGTACCTGTA--ATGGCTGTCTGATGGATTTTAAAGATCATATCACTGCACTGCCCCGGATAATCCGATCGACAGCGGTTAAATTTGGGATACCTTTTGGACCCGTCTTGAAACACGGACCAAGGAGTCTATCTAGTATGCGAGTAAATGGGGAAACCTATTTGCGCAATAAACTTGACTGATGGGATTACGAGTACTCTTCCATCTCGGGGTATTTCCATCAACATATGCAAATGTATATGGGAAATATACCATGAGCGTACTGGATACGACCCGAAAGATGGTGAACTATGCCTGATCAGGTTGAAGTCAGGGGAAACCCTGATGGAGGACCGAAACAGTTCTGACGTGCAAATCG---------------------AATTATGGAGTTCCGAGCGATGAGGAGCTCGACGGCAACAAGTTGGTCAAAAACTTCGAGTCGAACAACAAGATCTGGGTGTCGGCTCTAGTCGTTGGTGAGATTTGCGATGTCCCCTCCCATTGGCGTCAAAAGTACAAACTTGCGGAATGGATGAAGAAGCATCACATTCCTGGAATCAGTGATATCGATACTCGCGCTCTAACTAAGAAGATTCGAGAAAACGGCACTATTCTGGGAAGAATCATCCAGCAGCCTTCC------GGGCCCTTTCCTGGC---TTAGAGTTTAACGACCAGAACGAGCGGAATTTAGTTGATGAAGTTCCCATTAAGAAGCCAGTAACGTACAATCAGTCCGGCTCGCCTCGCATCTGTGCAATCGATTGCGGCTTGAAGCTCAATCGAATTCGCTGCTTCACAGAAAGAGGAGCTCGAGTGGATGTCGTGCCGCGGAACCAGGAGCTCAATTTGAAGGAATTTGATGGACTCTTCCTGAGCAACGGCCCAGGCGATCCCGTGACGTGCCAATCAACGGTGAAGAACATTCAGAAGTACTTAGCCAGTTCAAAAGTCAAACCGATCTTCGGTATTTGCTTGGGCCATCAACTGCTAGCCACTGCAATCGGCTGTAAGACTTATAAAATGAAGTATGGGAACCGCGGACACAACTTGCCAGCGCTCCATCATGACACAAATCGCTGTTTTATGACGTCCCAAAA----------------------------------------------------------------------TTCGTTCGAGTCAGCAAGCACATAGGCAGCTCCATGAAGAGTGTCGGAGAAGTCATGGCCATCGGGCGGAAGTTCGARGAAGCTTTTCAGAAAGCCCTCCGCATGGTCGATGAAAACGTGAATGGCTTCGATCCGAATTTGAAGTCGGTGAGTGAAGAAGAGCTCACAACGCCAACAGACAAGCGCATGTTTGTGCTGGCAGCTGCCTTGAAGGCCGGCTATTCTGTTGACCGTCTCTATGACTTGACCAAAATTGACCGCTGGTTCCTGGAGAAAATGAAGAACATCATTGACATCACCAAGCAGCTGGAGCAGCTGAAC---TGCGCCATTCCGGACGAGCTTCTAGCGCAAGCAAAGAAGTACGGCTTCTCTGACAAGCAAATTGCGGTCTACATCAAG--ATCGGAGCTGGCAGTAAGAAAACAAAGGCGCGAGCTGGGAATTCTGCCATTCGTGAAGCAAATCGACACCGTCGCTGGAGAATGGCCAGCTACCACAAATTACCTTTACTTAACGTACAACGCACGCTCGCATGACATCGTGACTAAT---GAGCAAATGATTATGGTAATTGGCTCGGGTGTTTATCGGATCGGAAGCTCTGTCGAGTTCGACTGGTGTGCTGTTGGATGTTTACGAGAGCTRCGGAAACTGGGCAAGAAGACGATCATGGTGAACTACAATCCCGAGACTGTTTCGACTGACTACGACATGTGCGATCGACTGTACTTTGAGGAGATTTCCTTCATATCATTAGTCAAGAAAGAGGAAAAAAAGAAACATTTGGTACATTAGGAATAATTTATGCTATATTAGCTATTGGATTACTAGGATTTATTGTTTGAGCTCATCATATATTTACAGTAGGTATAGATGTTGATACTCGAGCTTATTTTACATCAGCTACTATGATTATTGCAGTACCAACGGGTATTAAAATTTTTAGTTGATTAGCAACTCTTCATGGAACACAAATAAATTATTCTCCAGCCTTACTATGAGCTTTAGGGTTCGTATTTTTATTTACAGTAGGAGGACTAACAGGGGTAATTTTAGCTAATTCCTCTATTGATATTATTTTACATGATACCTATTATGTAGTAGCTCATTTTCACTATGTTCTTTCTATAGGAGCAGTATTTGCTATTATATCAGGATTTATTCACTGATACCCTTTATTCTCAGGATTAACAATAAACAAAAAATGACTAAAATCTCAATTTATTATTATATTTTTAGGTGTTAATTTAACATTTTTTCCTCAACATTTTCTAGGATTGGCAGGTATACCACGACGATACTCAGATTACCCAGACTCTTATATTTCATGAAATATTATTTCTTCATTAGGATCAACAATTTCTGTAATTGGAATTTTATTTTTTATTTTTATTATTTGAGAAAGA

**Eretmoptera murphyi** -----------------------CACCACT--ACCCTTAATTTC-A-AGAAAAAGCTGTCAA-TTTGTCATACCCTATCAAGTTCGG-ACCTGGTAAGTTTTCCCGTGTTGAGTCAAATTAAGCCGCAAAATCCAAGCC-ATGGTGTGGACTTCCGTCAATTCCTTTAAGTTTCAACTTTGCAACCATACTTCCCCCGGAAACT--AG-CTTT-GGTTTCCCGGAAAGCTACTGAATGCACCATGAAA--AGTAGTGACATCCAATTGCTAGCTGTCATCGTTTACAGTTAGAACTAGGGCGGTATCTAATCGCCTTCGATCCTCTAACTTTCGTTCTTGATTAATGAAAACATCCTTGGCAAATGCTTTCGCTTTAGTTAGTCTTGCGACGGTCTAAGAATTTCACCTCTCGCGCCGCAATACTAATGCCCCCAACTGCTTCTATTAATCATTACCTCT-TGATCTG-TATCAAACCAATAGAAAG-CGATA--------------GCATGCAAGCACACCA-TCTA-TAAGACCGAGGTCTTTTTCCATTATTCCATGCAAAAATATTCAAGGCATA-AGAGCCTGCTTTGAGCACCTTAATTTGTTCAAGGTAAAAGTAAGCCGAACTAAATAGACACCTAGCCTAATAAAAGGTATCGATGCTATTCATTAA--GTGTTCAGTCATATAGTTCAAGTAA-TCGGAAATGATAACA-GCCATTTACTTGATGGCGTTAC------ACCC-------G-TACTGAACAATAATCAACTTCGAACGTTTT-AACCGCAACAATTTTAATATACGCTAGTGGAGCTGGAATTACCGCGGCTGCTGGCACCAGACTTGCCCTCCACTGGATCCTCATTAAAGGATTTATACT-TGATTCATTCCAATTACAGAAC-TAAATAATTAGTTCTATATTGTTATTTTTCGTCACTACCTCC-----TTCAATATTCCGTCAAAAGCAATGTGCACTTCTTCCTTAGTAAGACGCTGTGATCCATTAGAAATTCATTCCAATGATTATTTGGCTATCAATGGGCCGGCATGAAGTTCATCAGCAAATAACTTGATCNTTTATGGTTGGGTATATTGTCTGGTGAGCATGTCGCAACCTGTATTATAGCTGTCTGATGGATTTTAAAGGTCATATTATTGCATCACTCAGAATAACCCGATCAACAGCGGTTAAATTTGGGATACCTTTTGGACCCGTCTTGAAACACGGACCAAGGAGTCTATCTAGTATGCAAGTAAATGAGGAAACTTATTTGCAGAAAAAACTTGACTGATGGGACTACGAGTGCTCCTCCATCCCGGGGTATTTTCATCAACATATGCAAATGTATATGGAAAATATACCATGAGCATACTGGATATGACCCGAAAGATGGTGAACTATGCCTGATCAGGTTGAAGTCAGGGGAAACCCTGATGGAGGACCGAAGCAATTCTGACGTGCAAATCGCTTACGTATCCTCTGATCGGCAACTACGGAATTCCGAGTGATGAAGAATTCGATGAGTACAAGTTGATGAAACATTTTGAGTCGAATGACAAGATTTGGGTRTCAGGCTTGGTTGTCGGAGAATTGTGTGAGACTCCGTCGCACTGGCGCCAAAAATACAAGCTTGCCGAATGGATGAAGAAGCATAACATCGTTGGCATTAGCGGCATCGACACTCGAGCTTTGACTAAGAAAATTCGTGAAAACGGAACCGTCTTGGGAAAAATTATCCATCAATCGTCA------GGACCGTTCCCGGGA---TTGGAATTCAAGGACCAGAATGAGCGAAATCTCGTCGACGAAGTTTCAATCAAGAAGCCGATTACTTACAACGCGACTGGCTCGCCAAGAATTTGTGCCGTTGACTGCGGATTGAAGTTGAATCAAATCCGCTGTTTTGTTAAGCGAGGAGCTCGCGTTGATGTAGTGCCATGGGACCACCCTTTAAATCCAGAYGAATGTGATGGGTTGTTCCTCAGCAATGGTCCCGGTGACCCCGTAATGTGCCAAACAACCGTCAAAAATATTCAGAAATTCCTCGCCGCTCAAAGAGTCAGGCCAATCTTCGGTATCTGCCTGGGTCATCAGCTCCTGTCAACCGCTGTTGGCTGCGAAACCTACAAGATGAAGTATGGCAATCGTGGTCACAATCTACCGGCTCTTCACCATGGAACCAATCGATGCTTCATGACATCTCAAAATCACGGATTCGCGGTCGATGTTAAGACGCTTGGCAAGGATTGGGAGCCGCTCTTCACCAACTTAAACGATTTTACCAGAGTCAGCAACAACATTGGAAGTTCCATGAAGAGCGTCGGTGAGGTGATGGCCATCGGACGAAAGTTTGAAGAAGCTTTCCAGAAAGCATTGCGAATGGTCGATGAGAATGTTTGTGGATTCGATCCCAACTTGAAAGCAGTCAGCGATGAGGAACTCCAAACACCAACAGACAAGCGAATGTTTGTTCTCGCTGCTGCTCTCAAGGCTGGCTACACCGTTGACCGGCTTTACGACTTGACAAAGATCGATCGTTGGTTCTTGGAAAAAATGAAAAACATCAATGCGATCACAATGGAGCTGGAAAAGCTGAAC---TGCGTCATTCCCGACGAGCTTCTAAAACATGCCAAGCAATACGGATTCTCTGACAAACAAATCGCCAATGCCATCAAA--ATCAGAATTAGCTGTTAGAAAGCAACGACGTGAGAGCAAAATTTTACCATTCGTCAAGCAAATCGACACCGTGGCGGGCGAATGGCCGGCATCAACGAATTATCTCTATCTGACTTACAACGCAAGCACTCATGATATCGAGTTTAAC---GACCAAATGGTGATGGTGATCGGGTCAGGCGTCTACCGCATCGGCAGCTCAGTTGAGTTCGACTGGTGCGCTGTTGGATGTTTGAGAGAATTGCGAAAGCTGGGTAAGAAGACAATCATGGTGAACTACAACCCAGAGACGGTCTCAACCGATTACGACATGTGTGATCGACTTTACTTTGAGGAGATTTCATTCATATTATTAGACAAGAAAGAGGAAAAAAAGAAACTTTTGGGGCTTTAGGAATAATTTATGCTATATCAGCAATTGGATTTTTAGGCTTTATTGTCTGAGCACACCACATATTTACAGTGGGTATAGACGTAGACACCCGAGCCTACTTTACGTCAGCAACTATAATTATTGCTGTTCCAACAGGAATTAAAATTTTCAGCTGATTAGCTACCTTACATGGTACCCCTTTAAATAACTCTCCTTCATTAATATGAGCCTTAGGTTTTGTATTTTTATTCACCGTAGGTGGTCTAACAGGAGTAGTTCTAGCAAATTCTTCTCTAGATATCGTACTTCATGATACTTATTATGTTGTCGCTCATTTTCACTATGTTTTATCAATAGGAGCAGTATTTGCCATTATGGCAGGATTTGTACACTGATACCCCTTGTTTACAGGACTATCTATAAATGAAAAATGATTAAAATCACAATTCTTAATTATATTTTTAGGGGTTAATTTAACATTTTTCCCTCAACATTTTTTAGGGTTAGCAGGTATACCCCGACGATACTCAGATTATCCAGATGCCTATACTTCTTGAAATATTATATCTACAGTAGGGTCAACAATTTCTCTTTTTGGTATTTTATTTTTTATTTTTATTATTTGAGAAAGA

**Eukiefferiella claripennis gp.**

--CACGAACTAAGAACGGCCATGCACCACT--ACCCTTAATTTC-A-AGAAAACGCTCTCAA-GTTGTCATACCCTATTAAGTTCGG-ACCTGGTAAGTTTTCCCGTGTTGAGTCAAATTAAGCCGCAAATTCCACATC--TGGTGTGATCTTCCGTCAATTCCTTTAAGTTTCAACTTTGCAACCATACTTCCCCCGGAAACT--AG-CTTT-GGTTTCCCGGAAAGCTACTGAATGCGCCATGAAA--AGTAGCGACATCCAATTGCTAGCTGTCATCGTTTACAGTTAGAACTAGGGCGGTATCTAATCGCCTTCGATCCTCTAACTTTCGTTCTTGATTAATGAAAACATCCTTGGCAAATGCTTTCGCTTTAGTTAGTCTTGCGACGGTCTAAGAATTTCACCTCTCGCGCCGCAATACTAATGCCCCCAACTGCTTCTATTAATCATTACCTCT-TGATCTG-TTTCAAACCAATAGAAAG-CGCTC-------------TCGCCGTGAAGCGAGCG--CGA-TAAGACCGAGGTCTTTTTCCATTATTCCATGCAAAAATATTCAAGGCATA-AGAGCCTGCTTTGAGCACCTTAATTTGTTCAAGGTAAAAGTAAGCCGGACTAGAGAGACACCTAGCCTAGTGAAAGGCATCGATGCTATCCATAA---GTGCCCGGTCATATAGTTCGAGTCG-GCGGAAACGAGAGCG-ACCA-CAACTTGATGGCGCCCC------GCCC-------G-GACCGAACAACAATCAACTTCGAACGTTTT-AACCGCAACAATTTTAATATACGCTAGTGGAGCTGGAATTACCGCGGCTGCTGGCACCAGACTTGCCCTCCACTGGATCCTCATTGAAGGATTTATACT-CGATTCATTCCAATTACAGAAC-CAAATAATTGGTTCTATATTGTTATTTTTCGTCACTACCTCC-----TTCAATATCCTCGCAAGGGCAATGTGCACTTCTCTCTTAGTAAGACGCTGTGATCCATTAGAAATTCGTTCCCTCGGTTATTTGGCTATCAATAGGCCAGCATGAAGTTCCTCAGCAAACAACTCAATCTTCAATGGTAGGGTATATTGTCTGGGGAGCATGTTGTGTCCTGTCTAATAGCTGTCTGATGGATTTTAAAGGCCATATTATCGCACCGCTCGTGATA-CCCGATCAACAGCGGTTCACTTTGGGATACCTTTTGGACCCGTCTTGAAACACGGACCAAGGAGTCTATCTAGTATGCGAGTAAATGAGGAAACTTATTTGCAGAAAAAACTTGACTGATGGGACTACGAGCGCTCCTCAATCCCGGGGTATTTTTATCAACATATGCAAATGTATATGGAAAATATACCATGAGCATACTGGATACGACCCGAAAGATGGTGAACTATGCCTGATCAGGTTGAAGTCAGGGGAAACCCTGATGGAGGACCGAAGCAGTTCTGACGTGCAAATCGCTGACTTATCCGTTGATCGGAAACTACGGCGTCCCGAGCGACGAGGAGTTTGACGAGCACAAACTGATGAAGCACTTCGAGTCGAACAACAAGATTTGGGTGTCGGGACTGGTCGTTGGCGAGCTTTGCGAAACGCCATCGCACTGGCGACAAAAGTACAAACTCGCCGAGTGGATGAAGAAGCACAACGTGGTCGGCATCAGTGGCATCGACACTCGCGCATTGACGAAGAAGATCCGCGAGGAGGGAACCGTTCTCGGTAAAATCATCCAGAAAACCAGC------GGTCCCTTCCCTGGC---CTGGAGTTTAAGGACCAAAACGAGAGGAATCTCGTCGCCGAAGTTTCCATCAAGAAGCCGATGACCTACAACGCGAAAGGCTCGCCACGCATCTGTGCCATTGACTGCGGACTTAAGCTCAACCAGATTCGATGCTTCATCAAGCGCGGAGCTCGAGTCGACGTCGTGCCATGGGATCATCCCTTGAACGCCGACGAATTCGATGGACTCTTCCTGAGCAACGGTCCAGGTGATCCGGTTATGTGCAAAAAGACCGTAAAAAATCTTCAAGACTTCTTCGCGCAGCCCAAAATGCGACCTGTTTTCGGTATCTGCCTCGGCCATCAGCTCCTGTCAACGGCTGTCGGATGTAAGACTTACAAGCTCAAATATGGAAATCGTGGGCACAACTTGCCGGCCATTCATCAYGGCACCGACCGCTGCTTTATGACGTCACAGAATCATGGATTCGCCGTGGACGTCAAGTCGATCGGRAAGGACTGGGAGCCGCTCTTCACGAACCTCAACGAC------------------------------------AAAAGTGTCGGCGAAGTCATGGCCATCGGAAGGAAATTCGAAGAAGCTTTCCAGAAAGCGCTGCGCATGGTCGACGAGAATGTCAACGGATTCGATCCAAACATCAAACCGGTAGTCGACGAGGAGCTCACTCAGCCAACAGACAAGCGAATGTTTGTTCTTGCCGCTGCCTTGAAGGCTGGTTACACCGTCGATCGCCTCTACGAGCTGACTAAGATCGACCGMTGGTTCCTCGAGAAGATGAAGAACATCAACAACGTCACGATGGAGCTTGAGAAGTTGAGC---TGCGGCATCACTGACGACCTCCTGAAGCAGGCTAAGAAACTTGGCTTTTCTGACAAGCAAATCGCCAAYCACATCAAA--CTCAGAGCTCGCCGTGCGCAAGCAACGACGCGAAGCAGGAATCTTGCCTTTCGTGAAGCAAATCGATACGGTGGCTGGTGAGTGGCCAGCGTCRACCAACTACCTCTATCTCACCTACAACGCGAGCGAACACGACATTGAGTTCAAC---GATCAGATGGTGATGGTCATTGGATCAGGAGTTTACCGCATCGGAAGCTCAGTGGAGTTTGACTGGTGCGCCGTGGGATGCTTGAGGGAGCTYAGAGCTCTGGGWAAGAAGACRATCATGGTGAACTACAACCCGGAGACTGTCTCCACCGACTACGACATGTGCGATCGATTGTACTT---------------CATATTATTAGTCAAGAAAGAGGAAAAAAGGAAACTTTTGGGGCCTTAGGAATAATCTATGCAATGTTAGCTATTGGTCTATTAGGTTTTGTTGTATGAGCTCATCATATATTTACAGTTGGAATAGATGTAGATACCCGAGCTTATTTTACCTCAGCTACTATAATTATTGCTGTGCCTACAGGAATCAAGATTTTTAGTTGATTGGCCACTTTACATGGAACTCAAATTAATAACTCTCCATCTATACTTTGAGCTTTAGGATTCGTTTTTTTATTTACAGTTGGAGGATTAACAGGAGTAATTTTAGCTAATTCATCTATTGACATTGTATTACATGACACTTACTACGTTGTAGCTCATTTTCACTATGTTCTTTCTATGGGAGCAGTATTTGCCATTATAGCGGGGTTCGTTCATTGATACCCCCTATTTTCAGGATTAACAATAAATGAAAAGTGACTAAAATCTCAATTTTTGATTATATTTTTTGGAGTTAATCTAACATTTTTCCCCCAACATTTTTTAGGATTAGCAGGAATACCTCGACGTTACTCAGATTATCCAGACGCCTACACTTCATGAAATATTTTGTCTTCGATTGGATCTACAATTTCTTTATTCGGGATTTTATTTTTTATTTTTATTATTTGAGAAAGA

**Eukiefferiella insolida** ------------------------------------------------------------------------------------------------------------------------------------------------------------------------------------------------CCCCCGGAAACT--AG-CTTT-GGTTTCCCGGAAAGCTACTGAATGCGCCATGAAA--AGTAGCGACATCCAACTGCTAGCTGTCATCGTTTACAGTTAGAACTAGGGCGGTATCTAATCGCCTTCGATCCTCTAACTTTCGTTCTTGATTAATGAAAACATCCTTGGCAAATGCTTTCGCTTTAGTTAGTCTTGCGACGGTCTAAGAATTTCACCTCTCGCGCCGCAATACTAATGCCCCCAACTGCTTCTATTAATCATTACCTCT-TGATCTG-TTTCAAACCAATAGAAAG-CGCTC-------------TCGCCGTGAAGCGAGCG--CGA-TAAGACCGAGGTCTTTTTCCATTATTCCATGCAAAAATATTCAAGGCATA-AGAGCCTGCTTTGAGCACCTTAATTTGTTCAAGGTAAAAGTAAGCCGGACTAGAGAGACACCTAGCCTAGTTAAAGGCATCGATGCTATCCATAA---GTGCCCGGTCATATAGTTCGAGTCG-GCGGAAACGAGAGCG-ACCA-CAACTTGATGGCGCCCC------GCCC-------G-GACCGAACAACAATCAACTTCGAACGTTTT-AACCGCAACAATTTTCATATACGCTAGTGGAGCTGGAATTACCGCGGCTGCTGGCACCAGACTTGCCCTCCACTGGATCCTCATTGAAGGATTTATACT-CGATTCATTCCAATTACAGAAC-CAAATAATTGGTTCTATATTGTTATTTTTCGTCACTACCTCC-----TTCAATATCCTCGCAAGGGCAATGTGCACTTCTCTCTTAGTAGGACGCTGTGATCCATTAGAAATTCGTTCCCTCGGTTATTTGGCTATCAATAGGCCAGCATGAAGTTCCTCAGCAAACAACTCAATCTTCAATGGTAGGGTATATTGTCTGGGGAGCATGTTGTGTCCTGTCTAATAGCTGTCTGATGGATTTTAAAGGCCATATTATCGCACCGCTCGTGATA-CCCGATCAACAGCGGTTCACTTTGGGATACCTTTTGGACCCGTCTTGAAACACGGACCAAGGAGTCTATCTAGTATGMGAGTAAATGAGGAAACTTATTTGCAGAAAAAACTTGACTGATGGGACTACGAGCGCTCCTCAATCCCGGGGTATTTTTATCAACATATGCAAATGCATATGGAAAATATACCATGAGCATACTGGATACGACCCGAAAGATGGTGAACTATGCCTGATCAGGTTGAAGTCAGGGGAAACCCTGATGGAGGACCGAAGCAGTTCTGACGTGCAAATCGCTGACTTATCCGTTGATCGGAAATTACGGCATCCCGAGCGACGAGGAGTTTGACGAACACAAACTGATGAAGCACTTCGAGTCGAACAACAAGATTTGGGTGTCTGGACTGGTGGTTGGTGAACTTTGCGAAACGCCATCGCATTGGCGACAAAAGTACAAGCTCGCAGAATGGATGAAAAAGCACAACGTGGTCGGCATCAGTGGAATTGATACTCGCGCATTGACGAAAAAGATCCGCGAGGAGGGAACCGTTCTCGGTAAAATCATTCAGAAATCTTGC------GGACCTTTTCTTGGC---TTAGAGTTCAAGGATCAGAACGAAAGAAACCTCGTCGCTGAAGTTTCAATCAAAAAGCCAATGACCTATAATCCCAAGGGTTCACCGCGCATCTGTGCCATTGATTGCGGACTTAAGCTTAACCAGATTCGATGCTTTATCAAGCGCGGAGCTCGAGTTGACGTCGTGCCGTGGGACCATCCCTTGAACGCAGAAGATTTTGATGGACTCTTTTTGAGCAACGGTCCAGGTGATCCGGTTATGTGCCAGAAGACTGTTAAGAATATTCAAAACTTCTTCGCGCAACCGAAAATGCGGCCTGTTTTCGGTATCTGCCTTGGCCATCAGCTATTGGCGACGGCTGTCGGATGTAAAACATACAAGCTAAAATATGGAAACCGTGGGCACAATTTGCCAGCCATTCATCATGGTTCCGACCGCTGCTTCATGACTTCACAAAACCATGGCTTTGCCGTTGATGTTAAATCAATTGTGAAGGACTGGGAGCCGTTGTTCACGAACCTTAATGATNNNNNNNNNNNNNNNNNNNNNNNNNNNNNNNNNNNNNNNNNNNNNNNNNNNNNNNNNNNNNNNNNNNNNNNNNNNNNNNNNNNNNNNNNNNNNNNNNNNNNNNNNNNNNNNNNNNNNNNNNNNNNNNNNNNNNNNNNNNNNNNNNNNNNNNNNNNNNNNNNNNNNNNNNNNNNNNNNNNNNNNNNNNNNNNNNNNNNNNNNNNNNNNNNNNNNNNNNNNNNNNNNNNNNNNNNNNNNNNNNNNNNNNNNNNNNNNNNNNNNNNNNNNNNNNNNNNNNNNNNNNNNNNNNNNNNNNNNNNNNNNNNNNNNNNNNNNNNNNNNNNNNNNNNNNNNNNNNNNNNNNNNNNNNNNNNNNNNNNNNNNNNNNNNNNNNNNNNNNNNNNNNNNNNNNNNNNNNNNNNNNNNNNNNNNNNNNNNNNNNNNNNNNNNNNNNNNNNNNNNNNNNNNNNNNNNNNNNNNNNNNNNNNNNNNNNNNNNNNNNNNNNNNNNNNNNNNNNNNNNNNNNNNNNNNNNNNNNNNNNNNNNNNNNNNNNNNNNNNNNNNNNNNNNNNNNNNNNNNNNNNNNNNNNNNNNNNNNNNNNNNNNNNNNNNNNNNNNNNNNNNNNNNNNNNNNNNNNNNNNNNNNNNNNNNNNNNNNNNNNNNNNNNNNNNNNNNNNNNNNNNNNNNNNNNNNNNNNNNNNNNNNNNNNNNNNNNNNNNNNNNNNNNNNNNNNNNNNNNNNNNNNNNNNNNNNNNNNNNNNNNNNNNNNCATATTATTAGTCAAGAAAGAGGAAAAAAGGAAACTTTTGGGGCTCTAGGAATAATTTATGCTATGTTAGCTATTGGACTTTTAGGATTCGTAGTTTGAGCTCATCATATATTTACTGTTGGTATAGACGTAGATACACGAGCTTATTTTACTTCTGCAACAATAATTATTGCTGTTCCAACAGGAATTAAAATTTTCAGATGATTAGCAACGCTTCATGGTACCCAAATTAATAATTCTCCATCAATATTATGAGCATTAGGATTTGTATTTTTATTTACTGTTGGGGGATTAACTGGTGTTGTTTTAGCTAATTCTTCTATTGACATTGTTCTTCATGATACTTATTATGTTGTAGCACATTTCCATTATGTACTTTCTATAGGAGCTGTATTTGCTATCATGGCAGGATTTGTACACTGATACCCCCTATTTTCAGGACTTTCAATAAATGAAAAATGATTAAAATCTCAATTTGTAATTATATTCTTAGGTGTAAATTTAACATTTTTTCCTCAGCATTTCTTAGGATTAGCAGGAATACCTCGACGATATTCTGATTATCCTGACGCTTATACTTCTTGAAATATTTTATCAACAATTGGATCTACTATCTCTTTATTTGGTATTTTATTTTTTATTTTTATTATTTGAGAAAGT

**Ferringtonia sp. 1** NNNNNNNNNNNNNNNNNNNNNNNNNNNNNNNNNNNNNNNNNNNNNNNNNNNNNNNNNNNNNNNNNNNNNNNNNNNNNNNNNNNNNNNNNNNNNNNNNNNNNNNNNNNNNNNNNNNNNNNNNNNNNNNNNNNNNNNNNNNNNNNNNNNNNNNNNNNNNNNNNNNNNNNNNNNNNNNNNNNNNNNNNNNNNNNNNNNNNNNNNNNNNNNNNNNNNNNNNNNNNNNNNNNNNNNNNNNNNNNNNNNNNNNNNNNNNNNNNNNNNNNNNNNNNNNNNNNNNNNNNNNNNNNNNNNNNNNNNNNNNNNNNNNNNNNNNNNNNNNNNNNNNNNNNNNNNNNNNNNNNNNNNNNNNNNNNNNNNNNNNNNNNNNNNNNNNNNNNNNNNNNNNNNNNNNNNNNNNNNNNNNNNNNNNNNNNNNNNNNNNNNNNNNNNNNNNNNNNNNNNNNNNNNNNNNNNNNNNNNNNNNNNNNNNNNNNNNNNNNNNNNNNNNNNNNNNNNNNNNNNNNNNNNNNNNNNNNNNNNNNNNNNNNNNNNNNNNNNNNNNNNNNNNNNNNNNNNNNNNNNNNNNNNNNNNNNNNNNNNNNNNNNNNNNNNNNNNNNNNNNNNNNNNNNNNNNNNNNNNNNNNNNNNNNNNNNNNNNNNNNNNNNNNNNNNNNNNNNNNNNNNNNNNNNNNNNNNNNNNNNNNNNNNNNNNNNNNNNNNNNNNNNNNNNNNNNNNNNNNNNNNNNNNNNNNNNNNNNNNNNNNNNNNNNNNNNNNNNNNNNNNNNNNNNNNNNNNNNNNNNNNNNNNNNNNNNNNNNNNNNNNNNNNNNNNNNNNNNNNNNNNNNNNNNNNNNNNNNNNNNNNNNNNNNNNNNNNNNNNNNNNNNNNNNNNNNNNNNNNNNNNNNNNNNNNNNNNNNNNNNNNNNNNNNNNNNNNNNNNNNNNNNNNNNNNNNNNNNNNNNNNNNNNNNNNNNNNNNNNNNNTTCAATATTCCCTTAAAAGCAATGTGCACTTCTTCCTTAGTAAGACGCTGTGATCCATTAGAAATTCATTCCAATGATTATTTGGCTATCAATGGGCCGGTATGAAGTTCATCAGCAAATAACTTGATCCTTTATGGTTGGGTATATTGTTTGGTGAGCGTGCCGTAACCTGCATTATAGCTGTCTGATGGATTTTAAAGGTCATATTATTGCATCACTCAGAATA-CCCGATCAACAGCGGTTAAATTTGGGATACCTTTTGGACCCGTCTTGAAACACGGACCAAGGAGTCTATCTAGTATGCAAGTAAATGAGTAAACTTATTTGCAGAAAAAACTTGACTGATGGGACTACGAGTGCTCCTCCATCCCGGGGTATTTTTATCAACATATGCAAATGTATATGGAAAATATACCATGAGCACACTGGATATGACCCGAAAGATGGTGAACTATGCCTGATCAGGTTGAAGTCAGGGGAAACCCTGATGGAGGACCGAAGCAATTCTGACGTGCAAATCGNNNNNNNNNNNNNNNNNNNNNNNNNNNNNNNNNNNNNNNNNNNNNNNNNNNNNNNNNNNNNNNNNNNNNNNNNNNNNNNNNNNNNNNNNNNNNNNNNNNNNNNNNNNNNNNNNNNNNNNNNNNNNNNNNNNNNNNNNNNNNNNNNNNNNNNNNNNNNNNNNNNNNNNNNNNNNNNNNNNNNNNNNNNNNNNNNNNNNNNNNNNNNNNNNNNNNNNNNNNNNNNNNNNNNNNNNNNNNNNNNNNNNNNNNNNNNNNNNNNNNNNNNNNNNNNNNNNNNNNNNNNNNNNNNNNNNNNNNNNNNNNNNNNNNNNNNNNNNNNNNNNNNNNNNNNNNNNNNNNNNNNNNNNNNNNNNNNNNNNNNNNNNNNNNNNNNNNNNNNNNNNNNNNNNNNNNNNNNNNNNNNNNNNNNNNNNNNNNNNNNNNNNNNNNNNNNNNNNNNNNNNNNNNNNNNNNNNNNNNNNNNNNNNNNNNNNNNNNNNNNNNNNNNNNNNNNNNNNNNNNNNNNNNNNNNNNNNNNNNNNNNNNNNNNNNNNNNNNNNNNNNNNNNNNNNNNNNNNNNNNNNNNNNNNNNNNNNNNNNNNNNNNNNNNNNNNNNNNNNNNNNNNNNNNNNNNNNNNNNNNNNNNNNNNNNNNNNNNNNNNNNNNNNNNNNNNNNNNNNNNNNNNNNNNNNNNNNNNNNNNNNNNNNNNNNNNNNNNNNNNNNNNNNNNNNNNNNNNNNNNNNNNNNNNNNNNNNNNNNNNNNNNNNNNNNNNNNNNNNNNNNNNNNNNNNNNNNNNNNNNNNNNNNNNNNNNNNNNNNNNNNNNNNNNNNNNNNNNNNNNNNNNNNNNNNNNNNNNNNNNNNNNNNNNNNNNNNNNNNNNNNNNNNNNNNNNNNNNNNNNNNNNNNNNNNNNNNNNNNNNNNNNNNNNNNNNNNNNNNNNNNNNNNNNNNNNNNNNNNNNNNNNNNNNNNNNNNNNNNNNNNNNNNNNNNNNNNNNNNNNNNNNNNNNNNNNNNNNNNNNNNNNNNNNNNNNNNNNNNNNNNNNNNNNNNNNNNNNNNNNNNNNNNNNNNNNNNNNNNNNNNNNNNNNNNNNNNNNNNNNNNNNNNNNNNNNNNNNNNNNNNNNNNNNNNNNNNNNNNNNNNNNNNNNNNNNNNNNNNNNNNNNNNNNNNNNNNNNNNNNNNNNNNNNNNNNNNNNNNNNNNNNNNNNNNNNNNNNNNNNNNNNNNNNNNNNNNNNNNNNNNNNNNNNNNNNNNNNNNNNNNNNNNNNNNNNNNNNNNNNNNNNNNNNNNNNNNNNNNNNNNNNNNNNNNNNNNNNNNNNNNNNNNNNNNNNNNNNNNNNNNNNNNNNNNNNNNNNNNNNNNNNNNNNNNNNNNNNNNNNNNNNNNNNNNNNNNNNNNNNNNNNNNNNNNNNNNNNNNNNNNNNNNNNNNNNNNNNNNNNNNNNNNNNNNNNNNNNNNNNNNNNNNNNNNNNNNNNNNNNNNNNNNNNNNNNNNNNNNNNNNNNNNNNNNNNNNNNNNNNNNNNNNNNNNNNNNNNNNNNNNNNNNNNNNNNNNNNNNNNNNNNNNNNNNNNNNNNNNNNNNNNCATATTATTAGACAAGAAAGAGGAAAAAAAGAAACTTTTGGGGCTTTAGGAATAATTTACGCTATATTAGCTATTGGGCTATTGGGGTTTATTGTATGAGCACATCATATATTTACTGTTGGCATAGATGTAGATACACGAGCTTATTTTACGTCTGCTACAATAATTATTGCTGTTCCTACCGGAATTAAAATTTTTAGATGATTAGCGACACTTCATGGAACCCCTATAAATAATTCACCATCTTTATTATGAGCTTTAGGATTTGTATTTTTATTTACAGTAGGAGGTTTAACAGGAGTAATTTTAGCTAACTCATCTATTGATATTGTTCTTCATGATACTTATTATGTAGTAGCTCATTTTCACTATGTTCTTTCAATAGGAGCAGTATTTGCTATTATAGCAGGATTTGTTCATTGATATACCTTATTTACAGGTCTAACAATAAATGAAAAATGATTAAAATCTCAATTTGCAGTAATATTCCTAGGTGTAAATTTAACATTTTTCCCACAACATTTTTTAGGATTAGCTGGTATACCTCGACGATACTCAGATTATCCTGACGCTTACACTTCTTGAAATATTATTTCAACTGTAGGTTCAACTATTTCTCTATTTGGTATTTTATTTTTTATTTTTATTATCTGAGAAAGA

**Ferringtonia sp. 2** -----------------------------------------------------------------------------------------------------------------------------------------------------------------------------------------------------------------------------------------------------------------------------------------------------------------------------------------------------------------------------------------------------------------------------------------------------------------TCATTACCTCT-TGATCTG-TATCAAACCAATAGAAAG-CGACT-----------GCACGTTTTAACGCGCACAGNCTA-TAAGACCGAGGTCTTTTTCCATTATTCCATGCAAAAATATTCAAGGCATA-AGAGCCTGCTTTGAGCACCTTAATTTGTTCAAGGTAAAAGTAAGCCGAACTAAATAGACACCTAGCCTAATTAAAGGTATCGATGCTATTCATTAA--GTGTTCAGTCATATAGTTCAAGTAA-TCGGAAATGATAGCA-GCCATTTACTTGATGGCGCTAC------ACCC-------G-TACTGAACAATAATCAACTTCGAACGTTTT-AACCGCAACAATTTTAATATACGCTAGTGGAGCTGGAATTACCGCGGCTGCTGGCACCAGACTTGCCCTCCACTGGATCCTCATTAAAGGATTTATACT-TGATTCATTCCAATTACAGAAC-TAAATGATTAGTTCTATATTGTTATTTTTCGTCACTACCTCC-------------------------------------------AGTAAGACGCTGTGATCCATTAGAAATTCATTCCAATGATTATTTGGCTATCAATGGGCCGATATGAAGTTCAGCAGCAAATAACTTGATCCTTTATGGTTGGGTATATTGTCTGTTGAGCATGTCGATACCTGCATTATAGCTGTCTGATGGATTTTAAAGGTCATATTATTGCATCACTCAGAATAACCCGATCAACAGTGGTTAAATTTGGGATACCTTTTGGACCCGTCTTGAAACACGGACCAAGGAGTCTATCTAGTATGCAAGTAAATGAGGAAACTTATTTGCAGAAAAAACTTGACTGATGGGACTACGAGTGCTCCTCCATCCCGGGGTATTTTTATCAACATATGCAAATGTATATGGAAAATATACCATGAGCATACTGGATATGACCCGAAAGATGGTGAACTATGCCTGATCAGGTTGAAGTCAGGGGAAACCCTGATGGAGGACCGAAGCAATTCTGACGTGCAAATCGNNNNNNNNNNNNNNNNNNNNNNNNNNNNNNNNNNNNNNNNNNNNNNNNNNNNNNNNNNNNNNNNNNNNNNNNNNNNNNNNNNNNNNNNNNNNNNNNNNNNNNNNNNNNNNNNNNNNNNNNNNNNNNNNNNNNNNNNNNNNNNNNNNNNNNNNNNNNNNNNNNNNNNNNNNNNNNNNNNNNNNNNNNNNNNNNNNNNNNNNNNNNNNNNNNNNNNNNNNNNNNNNNNNNNNNNNNNNNNNNNNNNNNNNNNNNNNNNNNNNNNNNNNNNNNNNNNNNNNNNNNNNNNNNNNNNNNNNNNNNNNNNNNNNNNNNNNNNNNNNNNNNNNNNNNNNNNNNNNNNNNNNNNNNNNNNNNNNNNNNNNNNNNNNNNNNNNNNNNNNNNNNNNNNNNNNNNNNNNNNNNNNNNNNNNNNNNNNNNNNNNNNNNNNNNNNNNNNNNNNNNNNNNNNNNNNNNNNNNNNNNNNNNNNNNNNNNNNNNNNNNNNNNNNNNNNNNNNNNNNNNNNNNNNNNNNNNNNNNNNNNNNNNNNNNNNNNNNNNNNNNNNNNNNNNNNNNNNNNNNNNNNNNNNNNNNNNNNNNNNNNNNNNNNNNNNNNNNNNNNNNNNNNNNNNNNNNNNNNNNNNNNNNNNNNNNNNNNNNNNNNNNNNNNNNNNNNNNNNNNNNNNNNNNNNNNNNNNNNNNNNNNNNNNNNNNNNNNNNNNNNNNNNNNNNNNNNNNNNNNNNNNNNNNNNNNNNNNNNNNNNNNNNNNNNNNNNNNNNNNNNNNNNNNNNNNNNNNNNNNNNNNNNNNNNNNNNNNNNNNNNNNNNNNNNNNNNNNTTCATTCGAGTCAGTAAACACATTGGAAGTTCGATGAAAAGTGTTGGCGAAGTTATGGCGATTGGCAGAAAGTTTGAAGAAGCTTTTCAAAAAGCTCTTCGCATGGTCGACGAGAATGTCAATGGATTCGATCCAAACTTGAAACCCGCAAACGATGAGGATTTGAAATCACCAACCGACAAGCGAATGTTCGTTCTTGCAGCAGCTTTAAAAGCTGGCTACACTGTTGATCGTCTCTACGATTTGACGAAGATTGATCGCTGGTTTCTCGAGAAACTCAAAAATATCAACGCAGTTACAGTCGAATTGGAGAAGCTCAAT---TGCATGATCTCCGACGAACTTTTGACTGAAGCGAAAAAACTCGGATTTTCTGATAAGCAAATTGCGTCATTGATCAAG--ATCAGAACTTGAAGTACGTAAACAGAGACGTGACGCTGGAATTCTTCCGTTTGTAAAACAAATCGACACAGTTGCAGGCGAGTGGCCCGCATCAACAAATTATCTTTACTTGACTTACAATGCAAGCACTCACGACATCGAATTCAAT---GAACAAATGGTGATGGTCATCGGCTCGGGAGTTTATCGAATTGGAAGTTCAGTTGAGTTTGACTGGTGCGCTGTTGGATGTTTAAGAGAACTCAGAAAGCTGGGCATGAAGACCATCATGGTGAATTACAATCCGGAAACTGTCTCAACCGATTATGACATGTGCGATCGACTTTACTTTGAAGAAATTTCATTCATATTATCAGCCAAGAAAGAGGAAAAAAAGAAACTTTTGGGGCATTAGGAATAATCTATGCCATATTAGCTATTGGTCTTTTAGGGTTTATTGTTTGGGCTCACCATATATTCACAGTAGGAATAGATGTAGATACCCGAGCTTACTTCACTTCTGCAACAATAATTATTGCTGTCCCAACAGGAATTAAAATTTTCAGTTGATTAGCCACTCTTCACGGAACCCCTCTTAATAACTCTCCTTCATTACTTTGAGCTTTAGGGTTTGTTTTTTTATTCACTGTTGGGGGACTTACCGGAGTTATTTTAGCTAACTCCTCTCTTGACATTGTTCTTCATGACACCTATTATGTTGTAGCACATTTTCACTATGTTCTATCTATGGGAGCAGTATTTGCTATTATAGCGGGATTTGTTCATTGATACTCTCTTTTCACAGGATTAACTATAAACGAAAAATGACTAAAATCACAATTTGCCATTATATTTTTAGGGGTTAATTTAACATTTTTTCCACAACATTTTTTAGGGCTTGCCGGGATACCCCGACGCTACTCTGATTATCCTGATGCCTATACTACATGAAATATTATTTCAACAGTTGGGTCCACTATTTCTTTATTTGGAATCTTATTTTTTATTTTTATTATCTGAGAAAGT

**Harrisonina petricola** ---------TAAGAACGGCCATGCACCACT--ACCCTTAATTTC-G-AGAAAGAGCTATTAA-TCTGTCTTACCCTATTAAGTTCGG-ACCTGGTAAGTTTTCCCGTGTTGAGTCAAATTAAGCCGCAGGCTCCACTCC-TGGTGGTGCCCTTCCGTCAATTCCTTTAAGTTTCAACTTTGCAACCATACTTCCCCCGGAAACT--AG-CTTT-GGTTTCCCGGA-AGCTACTGAATGCGCCAT-AAT--AGTAGCGACATCCAATTGCTGGCTGTCATAGTTTACAGTTAGAACTAGGGCGGTATCTAATCGCCTTCGATCCTCTAACTTTCGTTCTTGATTAATGAAAACATCCTTGGCAAATGCTTTCGCTTTAGTTAGTCTTACGACGGTCTAAGAATTTCACCTCTCGCGCCGTAATACTAATGCCCCCAACTGCTTCTATTAATCATTACCTCT-TGATCTG-TATCAAACCAATAGAAA--CTAAA---------------------------------TA-TAAGACCGAGGTCTTTTTCCATTATTCCATGCAAAAATATTCAAGGCGTATATAGCCTGCTTTGAGCACCTTAATTTGTTCAAGGTAATAGTAAGCTGAACTAAATAGGCA-TTAACCTAGTGAAAGGTGTCA-TGCTATTCATTAATAGTGTTCAGTCAAATAGTCCAAGTAA-TCGGAAATGACTACA-ACCA-TAACTTGATGGTGTAAC------ACCC-------G-TACTGGACAATAATCAACTTCGAACGTTTT-AACCGCAACAATTTTAATATACGCTAGTGGAGCTGGAATTACCGCGGCTGCTGGCACCAGACTTGCCCTCCACTAGATCCTTGTTAAAGGATTTATAAT-TAACTCATTCCAATTACAGAACATAGTTAACTAGTTCTATATTGTTATTTTTCGTCACTACCTCC-----TTCAATACTTCGTTAAACACGGTGTGCACTTTTCTCTTAGTAAGACGTTGTGATCCATTAGGAATTTGTATTGAT-ATTATTGGGCTACCAATAGACCGCTATGAAGTTCATCTGCAAATAATACAATCGTTTATGGTTGTGTATATTGTCGGGTGAGCATAGCGTGTTCTG--GTGTAGCTGCCTGATGGATTTTAAAGGTCATATCACTGCATTACCC-GGATATCCCGATCAACAACGGTTAAATTTGGGATACCTTTGGGACCCGTCTTGAAACACGGACCAAGGAGTCTATCTAGTATGCAAGTAAATGGGTAAACCTATTTGCGTAAATAACTTGACTGATGGGATTACGAATGTTCCTCCATCCCGGGGTATTTTTATCAACATATA-AAAT-TATATGGAAAATATACCATGAGCATACTGGATATGACCCGAAAGATGGTGAACTATGCCTGATCAGGTTGAAGTCAGGGGAAACCCTGATGGAGGACCGAAGCAATTCTGACGTGCAAATCGCTGACCTATCCACTYATTGGAAATTACGGCATTCCC---GATGAGCAGTTTGACAAAAATAAGCTGTCTGTAAACTTTGAGTCAAACAATAAAATCTGGATATCGGCTTTGGTTGTCGGTGAGCTATGTGATACACCATCGCATTGGCGTATGAAGTATAAGCTTTCAGAATGGATGAAAAAACATGGAATCCCCGGAATATCTGGAATCGATACMCGAGCGCTTACTAARAAAATCCGTGAAAATGGAACGGTCTTAGGCAAAATCTTTCAAAAATCATCTAATGACATTATCACTATGGGA---TTGACCTTCACAGATCAAAATCAACGTAATCTTGTCGATGAGGTCTCGACTAAAAAAGTGATTACTTATAATGCCGAAGGGTCTCCTCGAATTTGTGCCATTGATTGYGGTTTAAAGCTTAATCAAATCCGATGCTTTATTAATCGTGGCGCTCGTGTTGATGTCGTTCCATGGGATCATGAATTAAATTCTAAGGATTTCGATGGACTATTTTTGTCKAACGGACCCGGTGATCCCTCGATGTGCAAAAARACTATTGATAACATTAAAAGCGTAATATCCTCAAAAAACATTAAACCTATTTTTGGCATTTGCTTGGGACATCAATTGCTTGGAACGGCCATTGGTTGTAAGACTTACAAGTTAAAATATGGTAATCGTGGCCATAACCTACCGTGTTTGCATCATGGCACTAATCGTTGCTTTATGACGTCACAAAATCATGGTTTTGCTGTTGACGTCACAAATATTGCTGCCGATTGGGAACCATTGTTTACAAATGCAAATGAC------------------------------------------------GAAGTGATGGCCATTGGTCGGAAATTCGAAGAAGCCTTCCAAAAGGCACTAAGAATGGTGGATGAAAGCATCAATGGCTTTGATCCTAACTTAAARCCAGTAAATGATGAAGAGCTGTCAACACCGACTGATAAACGGATGTTTGTTCTTGCTGCTGCACTTAAAGCTGGTTATTCTGTTGATAAGTTGTATGATTTAACAAAAATTGATCGATGGTTTCTGGAAAAAATGAAAAATATCATTGATGTTACTTTAAAATTGGAAAAGTTTGAT---ATGAAATTACCAGTTGACGTTCTTTCAACGGCAAAAAAACTTGGTTTTTCCGATAAACAAATTGCCACATTTATCAAG--TACTGAATTGGCAGTAAGAAAGCAACGTCGTGAGAACCGTATTCTACCATTTGTAAAACAAATAGATACAGTTGCCGGAGAATGGCCAGCGTCAACAAATTATCTTTATTTCACTTACAATGCGTCTACTCACGATATCCAATTTGAA---GATCAGCATGTTATGGTCATCGGATCCGGTGTTTATAGAATCGGTAGCTCTGTTGAATTCGACTGGTGTGCTTGCGGTTGCCTTCGAGAGTTAAGAAATCTCGGAAAACGCACGATTATGGTTAATTACAATCCAGAGACT-----------------------------------------------------CATATTATTAGTCAAGAAAGAGGAAAAAAGGAAACTTTTGGTTCTTTAGGAATAATTTATGCTATACTTGCTATTGGTCTTTTAGGGTTTGTTGTTTGAGCTCATCATATATTTACAGTTGGAATAGATGTAGATACCCGAGCCTACTTTACATCAGCCACTATAATTATTGCTGTACCAACAGGAATTAAAATTTTTAGTTGACTAGCTACTTTACATGGAACTCAATTAAATTATTCTCCTTCTCTTCTTTGAGCTTTAGGATTTGTATTTTTATTCACTGTGGGAGGATTAACAGGAGTTGTATTAGCTAATTCTTCAATCGATATTGTTCTTCATGATACATATTATGTAGTTGCTCATTTTCACTATGTTCTTTCTATAGGAGCAGTATTTGCTATTATAGCAGGATTTGTTCACTGATACCCCTTATTTACAGGACTAACTTTAAATGAAGAATGATTAAAATCTCAATTTACTATTATATTTTTAGGGGTAAATTTAACGTTTTTTCCTCAACACTTTCTTGGGCTTGCTGGGATACCTCGACGATATTCTGACTACCCAGACGCGTATACTTCTTGAAATGTTGTATCAACTATTGGTTCTACAATTTCTTTATTTGGAATTATTTTCTTTTTATTTATTATTTGAGAAAGT

**Heleniella sp.** ---------------------TGCACCACTTACCCTTTAATTTC-A-TGAAAACGCTGTCAAAATTGTCATACCCTATCAAGTTCGG-ACCTGGTAAGCTTTCCCGTGTTGAGTCAAATTAAGCCGCAAATTCCAAGTC-ATGGTGTGGTCTTCCGTCAATTCCTTTAAGTTTCAACTTTGCAACCATACTTCCCCCGGAAACT--AG-CTTT-GGTTTCCCGGTAAGCTACTGAATGCACCATGAAA--AGTAGTGACATCCAATTGCTAGCTGTCATCGTTTACAGTTAGAACTAGGGCGGTATCTAATCGCCTTCGATCCTCTAACTTTCGTTCTTGATTAATGAAAACATCCTTGGCAAATGCTTTCGCTTTAGTTAGTCTTGCGACGGTCTAAGAATTTCACCTCTCGCGCTGCAATACTAATGCCCCCAACTGCTTCTATTAATCATTACCTCT-TGATCTG-TTTCAAACCAATAGAAAG-CGATA------------TGACGCGCAAACGCCACA-TCTA-TAAGACCGAGGTCTTTTTCCATTATTCCATGCAAAAATATTCAAGGCATA-AGAGCCTGCTTTGAGCACCTTAATTTGTTCAAGGTAAAAGTAAGCCGAACTAAATAGGCACCTAGCCTAGTGAAAGGCATCAGTGCTATTCATTAA--GTGTTCAGTCATATAGTTCAAGTAA-TCGGAAATGATGACA-GCCATTTACTTGATGGCGTCAC------ACCC-------G-TACTGAACAATAATCAACTTCGAACGTTTT-AACCGCAACAATTTTAATATACGCTAGTGGAGCTGGAATTACCGCGGCTGCTGGCACCAGACTTGCCCTCCACTTGATCCTCATTAAAGGATTTATACT-TGATTCATTCCAATTACAGAAC-TAAGTAATTAGTTCTATATTGTTATTTTTCGTCACTACTCCT-----TTCAATATTTCCTTAAAAGCAATGTGCACTTCTTTCTTAGTAAGACGCTGTGATCCATTAGAAATTTGTTTCAACAATCGTATGGCTGTCAATAGGCCGATGTGAAGTTCATTGGTAAATAACTAGATCTTTAACGGTTTAGTATATTGCTTGATGAGCGCATCGTAACCTGTACAATGGCTGTCTGATGGATTTTAAAGGTCATAATATTGCATCACTCAGATTAACCCGATCAACAGCGGTTAAATTTGGGATACCTTTTGGACCCGTCTTGAAACACGGACCAAGGAGTCTATCTAGTATGCGAGTAAATGAGGAAACTTATTTGCGTAAAAAACTTGACTAATGGGACTACGAGTATTCCTCCATCCCGGGGTATTTTCATCAACATATGCAAATGTATATGGAAAATATACCATGAGCATACTGGATATGACCCGAAAGATGGTGAACTATGCCTGATCAGGTTGAAGTCAGGGGAAACCCTGATGGAGGACCGAAGCAGTTCTGACGTGCAAATCGTTAACTTATCCATTGATTGGTAATTATGGAATTCCGAGTGACGACGAAATCGATGAGCATGGATTGATGAGGAACTTCGAGTCAAACGATAAGATTTGGGTGTCRGCTTTGGTTGTCGGTGAATTATGTGAAACTCCTTCGCATTGGCGACAAAAATACAAACTTGCTGAATGGATGAAGAAGCATAATGTCGTGGGAATTAGTGGAATTGATACACGTGCATTGACTAAAAAGATTCGCGAGAATGGAACGGTTCTCGGGAAGATTATTCAACAATCTAGT------GGACCTTTTCCGGGA---CTCGAATTTAAAGATCAGAACGAGAGGAATTTAGTCGACGAAGTTTCAATTAAGAAACCGATAACTTATAATCCGAAAGGATCGCCTCGCATTTGCGCTGTCGATTGCGGATTGAAATTGAATCAAATTCGTTGCTTTATCGATCGAGGAGCTCGTGTTGATTTAGTTCCATGGGATCATGCACTCAATCCTGATGATTTCGATGGTCTTTTCCTCAGTAACGGTCCAGGTGATCCCGTCACTTGTCAAAAGACTGTGAAGAATCTTCAAGCGTTTCTCGCGTCACCAAAAGTTAAGCCGGTTTTTGGTATTTGCTTGGGACATCAACTTTTAGCAACTGCCGCCGGTTGCAAGACTTATAAATTAAAGTATGGCAATCGAGGTCATAATTTGCCGGCTCTTCATCACGGCACAAACCGTTGCTTCATGACATCACAAAATCATGGCTTTGCTGTTGACGTAGCAACAATTGGAAAAGATTGGGAG------------------------TTCATTCGTGTCAGCAAAAATATTGGCAGTTCAATGAAGTCGGTTGGAGAAGTAATGGCAATTGGAAGAAAGTTTGAAGAAGCTTTTCAAAAAGCTTTRAGAATGGTTGATGAGAATGTAAATGGATTTGATCCGAATATCAAAGCTGTTAATGACGAAGAGTTGAAAACTCCAACTGACAAGAGAATGTTCGTYTTAGCAGCAGCTTTAAAAGCTGGTTATACAGTTGATCGTCTTTATGATTTAACAAAAATTGATCGTTGGTTTTTGGAGAAAATGCATAACATCATTGTCATTACAAAAGAATTGGAAATGCTCAAT---TGTATTGTTCCCGAAGAACTTTTAAAACAAGCAAAGAAATTGGGATTTTCTGATAAGCAAATTGCAAATTATATCAAA--ATCAGAACTTGCTGTAAGAAAACAAAGACGTGAAGCAGGAATCTTGCCATTTGTTAAACAAATCGATACAGTTGCTGGTGAATGGCCGGCATCAACAAATTATTTATATTTAACWTACAATGCAAGTTCACATGACATCGAATTCAAT---GATCAAATGGTGATGGTGATTGGATCGGGAGTTTATCGAATTGGAAGTTCAGTTGAATTTGATTGGTGCGCTGTTGGTTGTTTACGTGAACTTCGTAATCTTGGAAAGAAGACAATCATGGTGAATTATAATCCGGAAACAGTTTCCACCGATTACGATATGTGTGATCGTTTATATTTCGAAGAGATTTCATTCACATTATTAGTCAAGAAAGAGGAAAAAAGGAAACTTTTGGTAGTTTAGGAATAATTTATGCAATACTAGCTATTGGGCTATTAGGCTTTGTAGTATGAGCCCACCACATATTTACTGTTGGAATGGATGTTGATACTCGAGCCTACTTTACTTCTGCTACAATAATTATTGCAGTACCAACTGGAATTAAAATTTTTAGATGATTAGCTACACTTCATGGAACTCAAATTAATAACTCCCCTTCATTATTATGAGCATTAGGATTTGTATTTTTATTTACTGTTGGAGGATTAACTGGAGTTGTTCTAGCTAACTCTTCTATTGATATTGTATTACATGATACTTATTACGTAGTAGCTCATTTTCATTACGTTTTATCAATAGGGGCTGTATTTGCAATTATGGCAGGATTTGTTCATTGATATCCTTTATTTACAGGATTAACAATAAATGAATTCTGATTAAAAACTCAATTTACTATTATATTCTTAGGGGTTAATCTAACATTTTTCCCTCAACATTTTCTTGGATTAGCTGGAATACCTCGTCGATACTCTGATTATCCTGATGCTTATACTTCTTGAAATATTATTTCTACTGTAGGATCAACAATTTCTTTATTTGGTATCTTATTTTTTATTTTCATCATTTGAGAAAGA

**Heptagyia annulipes** ------------GAACGGCCATGCACCACT--ACCCTTAATTTC-G-AGAAAGAGCTATTAA-TCTGTCTTACCCTATTAAGTTCGG-ACCTGGTAAGTTTTCCCGTGTTGAGTCAAATTAAGCCGCAGGCTCCACTCC-TGGTGGTGCCCTTCCGTCAATTCCTTTAAGTTTCAACTTTGCAACCATACTTCCCCCGGAAACT--AG-CTTT-GGTTTCCCGGA-AGCTACTGAATGCGCCAT-AAT--AGTAGCGACATCCAATTGCTGGCTGTCATAGTTTACAGTTAGAACTAGGGCGGTATCTAATCGCCTTCGATCCTCTAACTTTCGTTCTTGATTAATGAAAACATCCTTGGCAAATGCTTTCGCTTTAGTTAGTCTTACGACGGTCTAAGAATTTCACCTCTCGCGCCGTAATACTAATGCCCCCAACTGCTTCTATTAATCATTACCTCT-TGATCTG-TATCAAACCAATAGAAA--CTAAA---------------------------------TA-TAAGACCGAGGTCTTTTTCCATTATTCCATGCAAAAATATTCAAGGCGTATATAGCCTGCTTTGAGCACCTTAATTTGTTCAAGGTAATAGTAAGCTGAACTAAATAGGCA-TTAACCTAGTGAAAGGTGTCA-TGCTATTCATTAATAGTGTTCAGTCATATAGTCCAAGTAA-TCGGAAATGATTACA-ACCA-TAACTTGATGGTGTAAC------ACCC-------G-TACTGGACAATAATCAACTTCGAACGTTTT-AACCGCAACAATTTTAATATACGCTAGTGGAGCTGGAATTACCGCGGCTGCTGGCACCAGACTTGCCCTCCACTAGATCCTTGTTAAAGGATTTATAAT-TAACTCATTCCAATTACAGAACATAGTTAACTAGTTCTATATTGTTATTTTTCGTCACTACCTCC-----TTCAATACTTCGTTAAACACAGTGTGCACTTTTCTCTTAGTAAGACGTTGTGATCCATTAGGAATTTGTATAGGGTATCATTGGGCTACCAATAGACCTCTATGAAGTTCACTAGCAAACAATACGATCGTTTATGATTGTGTACATTGTCTGGTGAGCATAGGGTGTTCTG--GTGTAGCTGCCTGATGGATTTTAAAGGTCACATCACTGCATTACCCCGGATAACCCGATCAACAACGGTTAAATTTGGGATACCTTTGGGACCCGTCTTGAAACACGGACCAAGGAGTCTATCTAGTATGCAAGTAAATGGGGAAACCTATTTGCGTAAATAACTTGACTGATGGGATTACGAATGTTCCTCCATCCCGGGGTATCTTTATCAACATATA-AAAT-TATATGGAAGATATACCATGAGCATACTGGATATGACCCGAAAGATGGTGAACTATGCCTGATCAGGTTGAAGTCAGGGGAAACCNTGATGGAGGACCGAAGCAATTCTGACGTGCAAATCGCTAACATATCCATTAATTGGCAATTATGGAATTCCC---GATGAACAACTCGATGACAATCAGCTGGCAGTGAACTTTGAATCGAACAATAAAATATGGATATCTGCATTGATTGTCGGTGAATTGTGTGAAACGCCATCACATTGGCGCATGAAATATAAATTGTCAGAATGGATGAAGAAACATGGCATTCCTGGCATTTCGGGCATTGACACTCGCCAATTGACTAAAAAGATTCGTGAAAATGGAACTGTCTTGGGAAAGATCGTTCAAGGTGCTGCAAATGTTGGACTTGTCGCCGAA---TTAAAGTTTCAAGATCAAAATCAACGTAATCTTGTTGATGAAGTGTCAATAAAAAAAACAGTGACCTACAATGCTGGTGGCTCACCACGAATTTGTGCTGTCGATTGTGGATTGAAGTTGAATCAAATTCGTTGCTTCGTTAAGCGTGGCGCACGTGTCGATCTCGTGCCGTGGAATCATAAATTGAATTCTAATGATTATGATGGTTTATTCTTGTCAAATGGTCCTGGTGATCCTGTCATGTGTCAAAAAACTGTTGAAAACATTAAAGATGTTTTAGCAGCGAAAAATGTGAAGCCAATCTTTGGCATTTGCTTGGGACATCAATTGTTGGCAACGGCAATTGGTTGCAAAACTTACAAGCTCAAGTATGGTAATCGTGGTCAYAATTTGCCGTGTCTTCATCATGGCACTAATCGTTGCTTTATGACGTCACAAAATCACGGTTTTGCCGTTGACGTCAACAGTATCGACGCCGATTGGGAGCCTTTATTTACGAATGCTAATGAT------------------------------TCAATGAAATCTGTTGGCGAGGTGATGGCCATTGGACGGAAATTCGAAGAAGCTTTCCAGAAAGCGTTGCGAATGGTAGACGAGAATGTTAATGGATTCGATCCCAACTTAAAGGCAGTAAATGATGAGGAACTTTCTACACCGACCGATAAACGGATGTTTGTTCTGGCGGCGGCTCTGAAAGCAGGATATTCCGTTGATAAACTTTACGATTTGACGAAGATTGATCGCTGGTTCTTGGACAAAATGAGGAACATCATTGATGTTACATTAGAGCTGGAAAAGTTGGAC---ACAAAACTACCAGTGGAACTTCTTTCGACGGCAAAGAAACTCGGATTTTCCGATAAACAAATTGCGACCTTCATCAAA--CTCAGAATTGGCCGTGCGAAAGCAACGCCGTGAAAATCAAATTTTACCATTTGTCAAACAAATCGATACCGTAGCTGGTGAATGGCCAGCAACAACAAATTATCTTTATTTCACTTATAATGCGTCAGCTCATGACATCCAATTTGAA---GATCAGCATGTCATGGTCATTGGTTCGGGAGTTTATCGGATTGGAAGTTCAGTTGAATTTGATTGGTGTGCTTGTGGATGTCTCCGTGAATTGAGGAAACTTGGCAAGCGAACAATTATGGTCAACTATAATCCGGAAACCGTATCGACCGATTAC--------------------------------------CATATTATTAGTCAAGAAAGAGGAAAAAAGGAAACTTTTGGATCTTTAGGAATAATTTATGCTATATTAGCTATTGGATTATTAGGTTTCGTAGTATGAGCTCACCATATATTTACTGTTGGAATAGATGTTGATACACGAGCTTATTTTACTTCTGCTACAATAATTATTGCCGTACCAACAGGTATTAAAATTTTTAGTTGATTAGCTACACTTCATGGAGCTCAATTAAATTATTCACCTTCTTTATTGTGAGCTTTAGGGTTTGTATTCTTATTTACAGTAGGAGGATTAACAGGAGTAGTTCTTGCAAATTCTTCTATTGATATTGTATTACACGATACTTACTATGTTGTTGCTCATTTCCATTATGTATTATCTATAGGAGCTGTATTTGCAATTATAGCAGGATTTGTACATTGATATCCTTTATTTACAGGATTAACTTTAAACGAAGAATGATTAAAGTCTCAATTTGCTATTATATTTTTAGGGGTAAATTTAACATTTTTCCCGCAACATTTTTTAGGATTAGCAGGAATACCTCGACGATATTCAGATTATCCCGATGCTTATACATCATGAAACGTAGTTTCTACTGTAGGATCAACAATTTCATTATTTGGAATTATTTTTTTCTTATTTATTATTTGAGAAAGT

**Troglocladius hajdi n.gen. n.sp. LH01** -------------GACGGCCATGCACCACT--ACCCTTAATTTC-A-AGAAAACGCTATCAA-GTTGTCATACCCTATTAAGTTCGG-ACCTGGTAAGTTTTCCCGTGTTGAGTCAAATTAAGCCGCAARTTCCAAGTC-AKGGKGTGATCTTCCGTCAATTCCTTTAAGTTTCAACTTTGCAACCATACTTCCCCCGGAAACT--AG-CTTT-GGTTTCCCGGAAAGCTACKGAATGCACCATGAAA--AGTAGTGACATCCAATTGCTAGCTGTCATCGTTTACAGTTAGAACTAGGGCGGTATCTAATCGCCTTCGATCCTCTAACTTTCGTTCTTGATTAATGAAAACATCCTTGGCAAATGCTTTCGCTTTAGTTAGTCTTGCGACGGTCTAAGAATTTCACCTCTCGCGCCGCAATACTAATGCCCCCAACTGCTTCTATTAATCATTACCTCT-TGATCTG-TATCAAACCAATAGAAAG-CGATA--------------CCGTCTGACGACGGCA-TCTA-TAAGACCGAGGTCTTTTTCCATTATTCCATGCAAAAATATTCAAGGCATT-AGAGCCTGCTTTGAGCACCTTAATTTGTTCAAGGTAAAAGTAAGCCGAACTAAATAGACACCTAGCCTAGTTAAAGACATCAGTGCTATTCATTAA--GTGTTCAGTCATATAGTTCAAGTAA-TSGGAAATGATAACA-GCCA-TAACTTGATGGCGTTAC------ACCC-------G-TACTGAACMATAATCAACTTCGAACGTTTT-AACCGCAACAATTTTAATATACGCTAGTGGAGCTGGAATTACCGCGGSTGCTGGCACCMGACTTGCCCTCCACWTGATCCTCATTAAAGGATTTATACK-TGATTCATTCCAATTACAGAAS-TAAATAATTAGTTCTATATTGTTATTTTTCGTCACTACCTCCCCGTGTTCAATATTCTGTCAAAGGCAATGTGCACTTCTCCCTTAGTAAGACGCTGTGATCCATTAGAAATTCATTTCAATGATTATTTGGCTATCAATAGGCCGACATGAAGTTCACCTACAAATAAGTCAATCCTCAATGATTGGGTATATTGTATGGTGAGCATGTCGTCACCTGTGTAATAGCTGTCTGATGGATTTTAAAGGTTGCATTACCGCATCGCTCAGAATGACCCGATCAACAGCGGTTAAATTTGGGATACCTTTTGGACCCGTCTTGAAACACGGACCAAGGAGTCTATCTAGTATGCAAGTAAATGAGGAAACTTATTTGCATAAAAAACTTGACTGATGGGACTACGAGTGCTCCTCCATCCCGGGGTATTTTTGTCAACATATACAAATGTATATGGAAAATATACCATGAGCATACTGGATATGACCCGAAAGATGGTGAACTATGCCTGATCAGGTTGAAGTCAGGGGAAACCCTGATGGAGGACCGAAGCAATTCTGACGTGCAAATCGNNNNNNNNNNNNNNNNNNNNNNNNNNNNNNNNNNNNNNNNNNNNNNNNNNNNNNNNNNNNNNNNNNNNNNNNNNNNNNNNNNNNNNNNNNNNNNNNNNNNNNNNNNNNNNNNNNNNNNNNNNNNNNNNNNNNNNNNNNNNNNNNNNNNNNNNNNNNNNNNNNNNNNNNNNNNNNNNNNNNNNNNNNNNNNNNNNNNNNNNNNNNNNNNNNNNNNNNNNNNNNNNNNNNNNNNNNNNNNNNNNNNNNNNNNNNNNNNNNNNNNNNNNNNNNNNNNNNNNNNNNNNNNNNNNNNNNNNNNNNNNNNNNNNNNNNNNNNNNNNNNNNNNNNNNNNNNNNNNNNNNNNNNNNNNNNNNNNNNNNNNNNNNNNNNNNNNNNNNNNNNNNNNNNNNNNNNNNNNNNNNNNNNNNNNNNNNNNNNNNNNNNNNNNNNNNNNNNNNNNNNNNNNNNNNNNNNNNNNNNNNNNNNNNNNNNNNNNNNNNNNNNNNNNNNNNNNNNNNNNNNNNNNNNNNNNNNNNNNNNNNNNNNNNNNNNNNNNNNNNNNNNNNNNNNNNNNNNNNNNNNNNNNNNNNNNNNNNNNNNNNNNNNNNNNNNNNNNNNNNNNNNNNNNNNNNNNNNNNNNNNNNNNNNNNNNNNNNNNNNNNNNNNNNNNNNNNNNNNNNNNNNNNNNNNNNNNNNNNNNNNNNNNNNNNNNNNNNNNNNNNNNNNNNNNNNNNNNNNNNNNNNNNNNNNNNNNNNNNNNNNNNNNNNNNNNNNNNNNNNNNNNNNNNNNNNNNNNNNNNNNNNNNNNNNNNNNNNNNNNNNNNNNNNNNNNNNNNNNNNNNNNNNNNNNNNNNNNNNNNNNNNNNNNNNNNNNNNNNNNNNNNNNNNNNNNNNNNNNNNNNNNNNNNNNNNNNNNNNNNNNNNNNNNNNNNNNNNNNNNNNNNNNNNNNNNNNNNNNNNNNNNNNNNNNNNNNNNNNNNNNNNNNNNNNNNNNNNNNNNNNNNNNNNNNNNNNNNNNNNNNNNNNNNNNNNNNNNNNNNNNNNNNNNNNNNNNNNNNNNNNNNNNNNNNNNNNNNNNNNNNNNNNNNNNNNNNNNNNNNNNNNNNNNNNNNNNNNNNNNNNNNNNNNNNNNNNNNNNNNNNNNNNNNNNNNNNNNNNNNNNNNNNNNNNNNNNNNNNNNNNNNNNNNNNNNNNNNNNNNNNNNNNNNNNNNNNNNNNNNNNNNNNNNNNNNNNNNNNNNNNNNNNNNNNNNNNNNNNNNNNNNNNNNNNNNNNNNNNNNNNNNNNNNNNNNNNNNNNNNNNNNNNNNNNNNNNNNNNNNNNNNNNNNNNNNNNNNNNNNNNNNNNNNNNNNNNNNNNNNNNNNNNNNNNNNNNNNNNNNNNNNNNNNNNNNNNNNNNNNNNNNNNNNNNNNNNNNNNNNNNNNNNNNNNNNNNNNNNNNNNNNNNNNNNNNNNNNNNNNNNNNNNNNNNNNNNNNNNNNNNNNNNNNNNNNNNNNNNNNNNNNNNNNNNNNNNNNNNNNNNNNNNNNNNNNNNNNNNNNNNNNNNNNNNNNNNNNNNNNNNNNNNNNNNNNNNNNNNNNNNNNNCATATTATTAGACAAGAAAGTGGAAAAAAGGAAACTTTTGGAACCTTAGGTATAATTTACGCAATATTAGCTATTGGCCTACTAGGATTTATTGTATGAGCTCATCATATATTTACAGTAGGAATAGATGTAGATACCCGAGCTTATTTTACTTCTGCTACAATAATTATTGCAGTTCCTACTGGAATTAAAATTTTTAGTTGATTAGCAACTCTTCATGGAACTCAATTTAATAACTCACCTTCTTTACTATGATCATTAGGTTTTGTTTTCTTATTTACAGTAGGAGGTTTAACCGGTGTTATTTTAGCCAATTCCTCTATTGATATTGTCCTTCATGACACTTATTATGTTGTTGCTCATTTTCATTATGTTCTTTCTATAGGAGCTGTATTTGCAATTATAGCAGGATTTGTTCATTGATATCCTTTATTTTCTGGATTAATAATAAACGAAAAATGATTAAAATCTCAATTTTTAATTATATTTTTAGGTGTTAATTTAACTTTTTTCCCTCAACATTTCTTAGGTCTTGCTGGAATACCCCGTCGTTATTCAGACTATCCAGATGCTTATACTTCATGAAATATTATATCAACAATCGGATCAACTATTTCTCTATTTGGAATTATTTTTTTTCTTTTTATTATTTGAGAAAGA

**Troglocladius hajdi n.gen. n.sp. LH02** NNNNNNNNNNNNNNNNNNNNNNNNNNNNNNNNNNNNNNNNNNNNNNNNNNNNNNNNNNNNNNNNNNNNNNNNNNNNNNNNNNNNNNNNNNNNNNNNNNNNNNNNNNNNNNNNNNNNNNNNNNNNNNNNNNNNNNNNNNNNNNNNNNNNNNNNNNNNNNNNNNNNNNNNNNNNNNNNNNNNNNNNNNNNNNNNNNNNNNNNNNNNNNNNNNNNNNNNNNNNNNNNNNNNNNNNNNNNNNNNNNNNNNNNNNNNNNNNNNNNNNNNNNNNNNNNNNNNNNNNNNNNNNNNNNNNNNNNNNNNNNNNNNNNNNNNNNNNNNNNNNNNNNNNNNNNNNNNNNNNNNNNNNNNNNNNNNNNNNNNNNNNNNNNNNNNNNNNNNNNNNNNNNNNNNNNNNNNNNNNNNNNNNNNNNNNNNNNNNNNNNNNNNNNNNNNNNNNNNNNNNNNNNNNNNNNNNNNNNNNNNNNNNNNNNNNNNNNNNNNNNNNNNNNNNNNNNNNNNNNNNNNNNNNNNNNNNNNNNNNNNNNNNNNNNNNNNNNNNNNNNNNNNNNNNNNNNNNNNNNNNNNNNNNNNNNNNNNNNNNNNNNNNNNNNNNNNNNNNNNNNNNNNNNNNNNNNNNNNNNNNNNNNNNNNNNNNNNNNNNNNNNNNNNNNNNNNNNNNNNNNNNNNNNNNNNNNNNNNNNNNNNNNNNNNNNNNNNNNNNNNNNNNNNNNNNNNNNNNNNNNNNNNNNNNNNNNNNNNNNNNNNNNNNNNNNNNNNNNNNNNNNNNNNNNNNNNNNNNNNNNNNNNNNNNNNNNNNNNNNNNNNNNNNNNNNNNNNNNNNNNNNNNNNNNNNNNNNNNNNNNNNNNNNNNNNNNNNNNNNNNNNNNNNNNNNNNNNNNNNNNNNNNNNNNNNNNNNNNNNNNNNNNNNNNNNNNNNNNNNNNNNNNNNNNNNNNNNNNNNNNNNNNNNNNNNNNNNNNNNNNNNNNNNNNNNNNNNNNNNNNNNNNNNNNNNNNNNNNNNNNNNNNNNNNNNNNNNNNNNNNNNNNNNNNNNNNNNNNNNNNNNNNNNNNNNNNNNNNNNNNNNNNNNNNNNNNNNNNNNNNNNNNNNNNNNNNNNNNNNNNNNNNNNNNNNNNNNNNNNNNNNNNNNNNNNNNNNNNNNNNNNNNNNNNNNNNNNNNNNNNNNNNNNNNNNNNNNNNNNNNNNNNNNNNNNNNNNNNNNNNNNNNNNNNNNNNNNNNNNNNNNNNNNNNNNNNNNNNNNNNNNNNNNNNNNNNNNNNNNNNNNNNNNNNNNNNNNNNNNNNNNNNNNNNNNNNNNNNNNNNNNNNNNNNNNNNNNNNNNNNNNNNNNNNNNNNNNNNNNNNNNNNNNNNNNNNNNNNNNNNNNNNNNNNNNNNNNNNNNNNNNNNNNNNNNNNNNNNNNNNNNNNNNNNNNNNNNNNNNNNNNNNNNNNNNNNNNNNNNNNNNNNNNNNNNNNNNNNNNNNNNNNNNNNNNNNNNNNNNNNNNNNNNNNNNNNNNNNNNNNNNNNNNNNNNNNNNNNNNNNNNNNNNNNNNNNNNNNNNNNNNNNNNNNNNNNNNNNNNNNNNNNNNNNNNNNNNNNNNNNNNNNNNNNNNNNNNNNNNNNNNNNNNNNNNNNNNNNNNNNNNNNNNNNNNNNNNNNNNNNNNNNNNNNNNNNNNNNNNNNNNNNNNNNNNNNNNNNNNNNNNNNNNNNNNNNNNNNNNNNNNNNNNNNNNNNNNNNNNNNNNNNNNNNNNNNNNNNNNNNNNNNNNNNNNNNNNNNNNNNNNNNNNNNNNNNNNNNNNNNNNNNNNNNNNNNNNNNNNNNNNNNNNNNNNNNNNNNNNNNNNNNNNNNNNNNNNNNNNNNNNNNNNNNNNNNNNNNNNNNNNNNNNNNNNNNNNNNNNNNNNNNNNNNNNNNNNNNNNNNNNNNNNNNNNNNNNNNNNNNNNNNNNNNNNNNNNNNNNNNNNNNNNNNNNNNNNNNNNNNNNNNNNNNNNNNNNNNNNNNNNNNNNNNNNNNNNNNNNNNNNNNNNNNNNNNNNNNNNNNNNNNNNNNNNNNNNNNNNNNNNNNNNNNNNNNNNNNNNNNNNNNNNNNNNNNNNNNNNNNNNNNNNNNNNNNNNNNNNNNNNNNNNNNNNNNNNNNNNNNNNNNNNNNNNNNNNNNNNNNNNNNNNNNNNNNNNNNNNNNNNNNNNNNNNNNNNNNNNNNNNNNNNNNNNNNNNNNNNNNNNNNNNNNNNNNNNNNNNNNNNNNNNNNNNNNNNNNNNNNNNNNNNNNNNNNNNNNNNNNNNNNNNNNNNNNNNNNNNNNNNNNNNNNNNNNNNNNNNNNNNNNNNNNNNNNNNNNNNNNNNNNNNNNNNNNNNNNNNNNNNNNNNNNNNNNNNNNNNNNNNNNNNNNNNNNNNNNNNNNNNNNNNNNNNNNNNNNNNNNNNNNNNNNNNNNNNNNNNNNNNNNNNNNNNNNNNNNNNNNNNNNNNNNNNNNNNNNNNNNNNNNNNNNNNNNNNNNNNNNNNNNNNNNNNNNNNNNNNNNNNNNNNNNNNNNNNNNNNNNNNNNNNNNNNNNNNNNNNNNNNNNNNNNNNNNNNNNNNNNNNNNNNNNNNNNNNNNNNNNNNNNNNNNNNNNNNNNNNNNNNNNNNNNNNNNNNNNNNNNNNNNNNNNNNNNNNNNNNNNNNNNNNNNNNNNNNNNNNNNNNNNNNNNNNNNNNNNNNNNNNNNNNNNNNNNNNNNNNNNNNNNNNNNNNNNNNNNNNNNNNNNNNNNNNNNNNNNNNNNNNNNNNNNNNNNNNNNNNNNNNNNNNNNNNNNNNNNNNNNNNNNNNNNNNNNNNNNNNNNNNNNNNNNNNNNNNNNNNNNNNNNNNNNNNNNNNNNNNNNNNNNNNNNNNNNNNNNNNNNNNNNNNNNNNNNNNNNNNNNNNNNNNNNNNNNNNNNNNNNNNNNNNNNNNNNNNNNNNNNNNNNNNNNNNNNNNNNNNNNNNNNNNNNNNNNNNNNNNNNNNNNNNNNNNNNNNNNNNNNNNNNNNNNNNNNNNNNNNNNNNNNNNNNNNNNNNNNNNNNNNNNNNNNNNNNNNNNNNNNNNNNNNNCATATTATTAGACAAGAAAGTGGAAAAAAGGAAACTTTTGGAACCTTAGGTATAATTTACGCAATATTAGCTATTGGCCTACTAGGATTTATTGTATGAGCTCATCATATATTTACAGTAGGAATAGATGTAGATACCCGAGCTTATTTTACTTCTGCTACAATAATTATTGCAGTTCCTACTGGAATTAAAATTTTTAGTTGATTAGCAACTCTTCATGGAACTCAATTTAATAACTCACCTTCTTTACTATGATCATTAGGTTTTGTTTTCTTATTTACAGTAGGAGGTTTAACCGGTGTTATTTTAGCCAATTCCTCTATTGATATTGTCCTTCATGACACTTATTATGTTGTTGCTCATTTTCATTATGTTCTTTCTATAGGAGCTGTATTTGCAATTATAGCAGGATTTGTTCATTGATATCCTTTATTTTCTGGATTAATAATAAACGAAAAATGATTAAAATCTCAATTTTTAATTATATTTTTAGGTGTTAATTTAACTTTTTTCCCTCAACATTTCTTAGGTCTTGCTGGAATACCCCGTCGTTATTCAGACTATCCAGATGCTTATACTTCATGAAATATTATATCAACAATCGGATCAACTATTTCTCTATTTGGAATTATTTTTTTTCTTTTTATTATTTGAGAAAGA

**Hydrobaenus saetheri** ------------------CCATGCACCACT--ACCCTTAATTTTCATAGAAAACGCTATCAA-ATTGTCATACCCTATTAAGTTCGG-ACCTGGTAAGCTTTCCCGTGTTGAGTCAAATTAAGCCGCAAATTCCACGCC-ATGGTGTGGTCTTCCGTCAATTCCTTTAAGTTTCAACTTTGCAACCATACTTCCCCCGGAAACT--AG-CTTT-GGTTTCCCGGAAAGCTACTGAATGCACCATGAAA--AGTAGTGACATCCAATTGCTAGCTGTCATCGTTTACAGTTAGAACTAGGGCGGTATCTAATCGCCTTCGATCCTCTAACTTTCGTTCTTGATTAATGAAAACATCCTTGGCAAATGCTTTCGCTTTAGTTAGTCTTGCGACGGTCTAAGAATTTCACCTCTCGCGCCGCAATACTAATGCCCCCAACTGCTTCTATTAATCATTACCTCT-TGATCTG-TATCAAACCAATAGAAAG-CGATA----------CAGCATCTTGCGACACTGCA-TCTA-TAAGACCGAGGTCTTTTTCCATTATTCCATGCAAAAATATTCAAGGCATT-AGAGCCTGCTTTGAGCACCTTAATTTGTTCAAGGTAAAAGTAAGCCGAACTAAATAGACACCTAGCCTAGTGAAAGGCATCAGTGCTATTCATTAA--GTGTTCAGTCATATAGTTCAAGTAA-TCGGAAATGATGGCA-GCCATTTACTTGATGGCGCCAC------ACCC-------G-TACTGAACAATAATCAACTTCGAACGTTTT-AACCGCAACAATTTTAATATACGCTAGTGGAGCTGGAATTACCGCGGCTGCTGGCACCAGACTTGCCCTCCACTTGATCCTCATTAAAGGATTTATACT-TGATTCATTCCAATTACAGAAC-TAAATAATTAGTTCTATATTGTTATTTTTCGTCACTACCTCC-----TTCAATATTTCCTTAACAACAATGTGCACTTCTCTCTTAGTAAGACGCTGTGATCCATTAGAAATTCATTCCAATGATCATTTGGCTATCAATAGGCCGGCGTGAAGTTCATCGACAAATAACTCGATCTTTTACGGTTGGGTATATTGTTTGGTGAGCGCGTCGTAACCTGTCTAATAGCTGTCTGATGGATTTTAAAGGCCATAACATTGCATCACTCAGGTTA-CCCGATCAACAGCGGTTCAATTTGGGATACCTTTTGGACCCGTCTTGAAACACGGACCAAGGAGTCTATCTAGTATGCAAGTATTTGAGTAAACTCATTTGCAGAAAAAACTTAACTGATGGGACTACGAGTGCTCCTCCATCCCGGGGTATTTTCATCAACATATGCAAATGCATATGGAAAATATACCATGAGCATACTGGATATGACCCGAAAGATGGTGAACTATGCCTGATCAGGTTGAAGTCAGGGGAAACCCTGATGGAGGACCGAAGCAATTCTGACGTGCAAATCG---ACTTATCCGCTGATCGGAAATTATGGAATTCCGAGCGACGAAGARTTCGACGAACATAAGCTGATGAAGCATTTCGAGTCGAACAACAAGATWTGGGTTTCCGGATTGGTTGTCGGTGAACTCTGCGAAACTCCATCGCACTGGCGTCAAAAGTACAAGCTCGCCGAGTGGATGAAAAAACATAAAATTGTCGGCATCAGCGGCATCGACACACGCGCATTGACCAAGAAGATTCGTGAGGATGGAACCGTTCTGGGAAAGATCGTCCAACAATCTGCT------GGGCCGTTTCCAGGT---TTGGAATTTAAGGATCAGAACCAACGGAACCTCGTCGCGGAAGTTTCCATCAAGAAGGCGATAACTTACAACGCATCTGGTTCGCCGCGAATTTGCGCTGTCGATTGCGGATTAAAACTTAATCAGATTCGTTGCTTCATCAAACGTGGAGCGCGAGTCGATGTCGTGCCATGGGATCAGCCTTTGAACCCCGACAACTTTGATGGACTCTTCCTCAGCAACGGCCCCGGCGATCCCGTCATGTGTCAAAAGACCGTCGCGAACCTTCAAAAGTATCTCGCGTCGCCCCAAGTGAAACCGACGTTCGGCATTTGCTTGGGTCATCAGCTGCTGGCGACCGCAGTCGGATGCAAAACTTACAAGATGAAGTACGGCAATCGTGGGCATAACTTGCCAGCTCTCCACCACGGATCCAACCGGTGCTTCATGACGTCACAGAATCACGGGTTCGCGGTTGATGTCGCCACGATTGGCAAGGATTGGGAGCCGCTCTTC---------------TTCATCCGAGTTAGCAAGAAYATTGGCAGCTCCATGAAGAGCGTTGGTGAGGTGATGGCGATCGGACGCAAGTTCGAGGAGGCTTTTCAGAAGGCGCTGCGAATGGTTGACGAGAACGTTGAGGGATTCGATCCGAACTTAAAACCGGTCAACGACGAAGAGCTGACAACGCCAACAGATAAGCGAATGTTCGTGCTCGCAGCGGCATTGAAGGCTGGCTACACCGTCGATCGTCTTTACGATCTGACGAAGATCGATCGCTGGTTCTTGCACAAGATGAAGAACATCATCGACATCACAATCGACTTGGAGAAGTTAAAC---TGCACAATCACCGACGGTGTCTTGCACCATGCTAAGAAGTTGGGATTCTCCGACAAGCAAATCGCTTCGTGCATCAAA--GTCAGAGCTGGCGGTGCGCAAGCAACGACGTGAGGCTGGAATTCTACCTTTCGTGAAGCAGATCGACACCGTGGCTGGCGAGTGGCCGGCATCGACGAACTATCTTTACTTGACGTACAATGCAAGCGCTCACGACGTCGAGTTCGGC---GAGCAGATGGTGATGGTGATTGGATCCGGAGTTTACCGCATCGGCAGCTCCGTGGAGTTCGATTGGTGCGCTGTTGGATGCTTGAGGGAGTTGCGAAAGTTRGGCAAGAAGACGATCATGGTGAACTACAATCCGGAAACCGTTTCCACCGACTACGACATGTGCGATCGCTTGTACTTCGAGGAGATTT----CATATTATTAGTCAAGAAAGAGGAAAAAAGGAAACTTTCGGAGCTTTAGGGATAATTTACGCTATATTAGCTATTGGACTTTTAGGGTTTGTTGTTTGAGCTCATCATATATTTACAGTTGGAATAGATGTAGATACTCGAGCTTACTTTACTTCTGCTACTATAATTATTGCTGTGCCTACAGGTATTAAAATTTTTAGTTGATTAGCTACGCTTCATGGAACTCAATTAAATAATTCTCCTTCTTTATTATGAGCTTTAGGGTTTGTATTTTTATTTACAGTAGGGGGATTAACTGGAGTAATTTTAGCTAACTCTTCTATTGATATTATTCTTCACGATACTTATTATGTAGTTGCCCATTTTCATTATGTATTATCTATAGGAGCTGTATTTGCTATTATAGCAGGTTTTATCCATTGATACTCTTTATTTACTGGGCTAATTATAAATGAAACTTGATTAAAATCTCAATTTGCTATTATATTTTTAGGTGTTAATATGACATTTTTTCCTCAACATTTTTTAGGTTTAGCTGGAATGCCTCGTCGTTATTCAGATTATCCTGATGCTTATACTTCTTGAAATATTGTTTCTACAATTGGATCTACAATTTCTTTATTTGGAATTTTATTCTTTTTATTTATTATTTGAGAAAGT

**Limaya sp.** --------CTAAGAACGGCCATGCACCACT--ACCCTTAATTTC-G-AGAAAGAGCTATTAA-TCTGTCTTACCCTATTAAGTTCGG-ACCTGGTAAGTTTTCCCGTGTTGAGTCAAATTAAGCCGCAGGCTCCACTCC-TGGTGGTGCCCTTCCGTCAATTCCTTTAAGTTTCAACTTTGCAACCATACTTCCCCCGGAAACT--AG-CTTT-GGTTTCCCGGA-AGCTACTGAATGCGCCAT-AAT--AGTAGCGACATCCAATTGCTGGCTGTCATTGTTTACAGTTAGAACTAGGGCGGTATCTAATCGCCTTCGATCCTCTAACTTTCGTTCTTGATTAATGAAAACATCCTTGGCAAATGCTTTCGCTTTAGTTAGTCTTACGACGGTCTAAGAATTTCACCTCTCGCGCCGTAATACTAATGCCCCCAACTGCTTCTATTAATCATTACCTCT-TGATCTG-TATCAAACCAATAGAAA--CTAAA---------------------------------TA-TAAGACCGAGGTCTTTTTCCATTATTCCATGCAAAAATATTCATAGCGTATATAGCCTGCTTTGAGCACCTTAATTTGTTCAAGGTAATAGTAAGCTGAACTAAATAGGCA-TTAACCTAGTGAAAGGTGTCA-TGCTATTCATTAATAGTGTTCAGTCATATAGTCCAAGTAA-TCGGAAATGATTACA-ACCA-TAACTTGATGGTGTAAC------ACCC-------G-TACTGGACAATAATCAACTTCGAACGTTTT-AACCGCAACAATTTTAATATACGCTAGTGGAGCTGGAATTACCGCGGCTGCTGGCACCAGACTTGCCCTCCACTGGATCCTTGTTAAAGGATTTATAAT-TAACTCATTCCAATTACAGAACATAGTTAACTAGTTCTATATTGTTATTTTTCGTCACTACCTCC-----TTCAATACTTCGTTAAACATGGTGTGCACTTTTCTCTTAGTAAGACGTTGTGATCTATTAGGAATTTGTATAGGATATCATTGGGTTACCAATAGACTATTATGAAGTTCAACAGCAAATAACATAATCGTTTATGGTTGTGTATATTGTCTGGTGAGCATAGTATGTTCTG--GTGTAACTGCCTGATAGATTTTAAAGGTTATATCACTGCATTACCCCGGATAACCCGATCAACAACGGTTAAATTTGGGATACCTTTGGGACCCGTCTTGAAACACGGACCAAGGAGTCTATCTAGTATGCAAGTAAATGGGGAAACCTATTTGCGTAAATAACTTGACTGATGGGATTACGAGTATTCCTCCATCCCGGGGTATTTTTATCAACATATA-AAAT-TATATGGAAAATATACCATGAGCATACTGGATATGACCCGAAAGATGGTGAACTATGCCTGATCAGGTTGAAGTCAGGGGAAACCCTGATGGAGGACCGAAGCAATTCTGACGTGCAAATCG---------CCACTTATTGGGAATTATGGCATTCCC---GATGAAGAATTAGACGAACATCAATTGGCAGTAAACTTTGAATCGAATAATAAAATTTGGATCTCGGCATTAGTAGTTGGAGAACTGTGTGAAACGCCTTCCCATTGGCGCATGAAGTATAAGTTATCGGAATGGATGAAAAAACATGGTATCCCTGGTATTTCTGGTATTGACACTCGTGCATTAACAAAACAAATTCGTGAAAACGGTACGGTGTTGGGAAAGATTATTCAAAAGTCTGGAACTGATACCATTACAAATGGA---TTAATATTTCAAGATCAGAATGAGCGTAATCTTGTTGATGAAGTTTCAATTAAAAAACCGATAACTTACAATGCTGGAGGATCACCCCGTATTTGTGYCGTCGATTGTGGATTGAAATTGAATCAAATACGTTGTTTTGTCAATCGTGGAGCTCGGGTTGATCTTGTTCCGTGGGATTATAAATTGAATTGTAACGATTTTGATGGACTTTTTTTATCTAATGGTCCTGGAGATCCAGTTATGTGTAGAAAAACTGTAGAAAACATTAAAGGTATAATCGCATTGCCGAATGCTAAACCGATTTTTGGTATTTGCTTGGGTCATCAGTTATTATCGACGGCAATCGGTTGTAATACCTACAAACTTAAGTACGGTAATCGAGGACATAATTTGCCATGTCTTCATCATGGTACAAATCGATGCTTTATGACTTCACAAAATCATGGTTTCGCCGTTGAT---------------------------------------------------------------------AATATTGGCAGTTCTATGAAAAGTGTTGGTGAAGCAATGGCAATCGGTCGAAAGTTTGAAGAAGCWTTCCAGAAAGCATTGCGAATGGTAGATGAAAATGTTAATGGGTTTGATCCAAATTTGAAGGCAGTGAATGATGAAGAACTTTCAACTCCTACCGACAAGAGAATGTTTGTATTGGCTGCTGCATTAAAAGCTGGTTATTCGGTTGATAAATTGTACGATTTGACGAAAATTGACCGCTGGTTTCTAGAAAAAATGAAAAACATCATAGATGTTACCTTAGAGCTAGAATCTTTGGAT---ACAAAACTGTCGGTTGAATTGCTTTCAGTAGCTAAGAAACTTGGTTTCTCCGATAAACAGATTGCGGTATTTATCAAA--TTCAGAGTTGGCAGTCAGAAAACARCGTCGTGAGAATAAGATACTTCCGTTTGTAAAACAAATTGATACTGTTGCTGGTGAATGGCCTGCAACTACCAACTATCTTTACTTCACATATAATGCTTCAACTCATGATATTAAATTTGAA---AATCARCATGTCATGGTTATAGGATCTGGAGTTTATCGTATTGGTAGTTCTGTTGAATTTGATTGGTGTGCTTGTGGATGCCTGCGTGAGCTGAGAAAAATTGGTAAAAAAACAATTATGGTGAATTATAATCCTGAAACTGTTTCCACGGAYTACGATATGTGTGATCGACTYTATTTTGAAGAGATTTCTTTCATATTATTAGTCAAGAAAGAGGAAAAAAGGAAACATTTGGATCCTTAGGAATAATTTATGCTATACTTGCTATTGGTTTATTAGGATTTGTTGTTTGAGCTCATCATATATTTACAGTAGGAATAGACGTTGATACTCGAGCATATTTCACTTCAGCTACTATAATTATTGCTGTACCGACTGGAATTAAAATTTTTAGCTGACTTGCTACTTTACACGGAGCACAGTTAAATTATTCTCCTTCTTTACTTTGAGCTTTAGGATTTGTCTTTTTATTTACAGTAGGAGGATTAACAGGAGTTGTTCTTGCTAATTCTTCTATTGATATTGTTCTTCATGACACTTATTATGTAGTTGCTCATTTTCACTATGTCCTTTCAATAGGAGCTGTTTTTGCTATTATAGCAGGATTTGTTCACTGATACCCTTTATTTACGGGATTAACTTTAAATGAAGAATGATTAAAATCTCAATTTTCAATTATATTTTTAGGAGTAAATTTAACTTTTTTTCCTCAACATTTTTTAGGATTAGCTGGAATACCACGACGATATTCTGATTATCCTGATGCTTACACTTCATGAAATGTTGTTTCAACAATTGGTTCAACTATTTCTTTATTTGGAATTATCTTTTTTTTATTTATTATTTGAGAAAGA

**Limnophyes brachyarthra** NNNNNNNNNNNNNNNNNNNNNNNNNNNNNNNNNNNNNNNNNNNNNNNNNNNNNNNNNNNNNNNNNNNNNNNNNNNNNNNNNNNNNNNNNNNNNNNNNNNNNNNNNNNNNNNNNNNNNNNNNNNNNNNNNNNNNNNNNNNNNNNNNNNNNNNNNNNNNNNNNNNNNNNNNNNNNNNNNNNNNNNNNNNNNNNNNNNNNNNNNNNNNNNNNNNNNNNNNNNNNNNNNNNNNNNNNNNNNNNNNNNNNNNNNNNNNNNNNNNNNNNNNNNNNNNNNNNNNNNNNNNNNNNNNNNNNNNNNNNNNNNNNNNNNNNNNNNNNNNNNNNNNNNNNNNNNNNNNNNNNNNNNNNNNNNNNNNNNNNNNNNNNNNNNNNNNNNNNNNNNNNNNNNNNNNNNNNNNNNNNNNNNNNNNNNNNNNNNNNNNNNNNNNNNNNNNNNNNNNNNNNNNNNNNNNNNNNNNNNNNNNNNNNNNNNNNNNNNNNNNNNNNNNNNNNNNNNNNNNNNNNNNNNNNNNNNNNNNNNNNNNNNNNNNNNNNNNNNNNNNNNNNNNNNNNNNNNNNNNNNNNNNNNNNNNNNNNNNNNNNNNNNNNNNNNNNNNNNNNNNNNNNNNNNNNNNNNNNNNNNNNNNNNNNNNNNNNNNNNNNNNNNNNNNNNNNNNNNNNNNNNNNNNNNNNNNNNNNNNNNNNNNNNNNNNNNNNNNNNNNNNNNNNNNNNNNNNNNNNNNNNNNNNNNNNNNNNNNNNNNNNNNNNNNNNNNNNNNNNNNNNNNNNNNNNNNNNNNNNNNNNNNNNNNNNNNNNNNNNNNNNNNNNNNNNNNNNNNNNNNNNNNNNNNNNNNNNNNNNNNNNNNNNNNNNNNNNNNNNNNNNNNNNNNNNNNNNNNNNNNNNNNNNNNNNNNNNNNNNNNNNNNNNNNNNNNNNNNNNNNNNNNNNNNNNNNNNNNNNNNNNNNNNNNNNNNNNNNNNNNN------------------------------------TTAGTAAGACGCTGTGATCCATTAGAAAC-CATTCCAATGGTTATTTGGCTGTCAATAGGCCGGTACGAAGTTCTCCGGCAAATGGCTTGGTCTNNGATNNCCGGGTGTATTGTTTGGGGAGCGTGCCGTCACCTGTGTTATAGCTGTCTGATGGATTTTAAAGGTCATATTATTGCACCACTCAGAATAACCCGATCAACAGTGGTTAAATTTGGGATACCTTTTGGACCCGTCTTGAAACACGGACCAAGGAGTCTATCTAATATGCGAGTAAATGAGGAAACTTATTTGCAGAAAAAACTTGACTGATGGGACTACGAGTGCTCCTCCATCCCGGGGTATTTCTATCAACACACGCAAATGTGTGTGGGAAATATACCATGAGCATACTGGATATGACCCGAAAGATGGTGAACTATGCCTGATCAGGTTGAAGTCAGGGGAAACCCTGATGGAGGACCGAAGCAGTTCTGACGTGCAAATCGNNNNNNNNNNNNNNNNNNNNNNNNNNNNNNNNNNNNNNNNNNNNNNNNNNNNNNNNNNNNNNNNNNNNNNNNNNNNNNNNNNNNNNNNNNNNNNNNNNNNNNNNNNNNNNNNNNNNNNNNNNNNNNNNNNNNNNNNNNNNNNNNNNNNNNNNNNNNNNNNNNNNNNNNNNNNNNNNNNNNNNNNNNNNNNNNNNNNNNNNNNNNNNNNNNNNNNNNNNNNNNNNNNNNNNNNNNNNNNNNNNNNNNNNNNNNNNNNNNNNNNNNNNNNNNNNNNNNNNNNNNNNNNNNNNNNNNNNNNNNNNNNNNNNNNNNNNNNNNNNNNNNNNNNNNNNNNNNNNNNNNNNNNNNNNNNNNNNNNNNNNNNNNNNNNNNNNNNNNNNNNNNNNNNNNNNNNNNNNNNNNNNNNNNNNNNNNNNNNNNNNNNNNNNNNNNNNNNNNNNNNNNNNNNNNNNNNNNNNNNNNNNNNNNNNNNNNNNNNNNNNNNNNNNNNNNNNNNNNNNNNNNNNNNNNNNNNNNNNNNNNNNNNNNNNNNNNNNNNNNNNNNNNNNNNNNNNNNNNNNNNNNNNNNNNNNNNNNNNNNNNNNNNNNNNNNNNNNNNNNNNNNNNNNNNNNNNNNNNNNNNNNNNNNNNNNNNNNNNNNNNNNNNNNNNNNNNNNNNNNNNNNNNNNNNNNNNNNNNNNNNNNNNNNNNNNNNNNNNNNNNNNNNNNNNNNNNNNNNNNNNNNNNNNNNNNNNNNNNNNNNNNNNNNNNNNNNNNNNNNNNNNNNNNNNNNNNNNNNNNNNNNNNNNNNNNNNNNNNNNNNNNNNNNNNNNNNNN------------------------------------------GTCGGAGAAGTCATGGCGATAGGCAGAAAGTTTGAGGAAGCTTTCCAAAAAGCTCTGCGAATGGTCGACGAGAGTGTGAATGGCTTTGATCCGAACCTGAAGCCAGTGTCCGACGAAGAACTGATGACACCCACCGACAAACGCATGTTTGTTCTCGCCGCTGCGCTCAAAGCTGGTTACTCCGTCGACAAACTGTACGATTTGACTAAAATTGATCGCTGGTTCCTCGAAAAGATGAAGAACATCAACGCAATCACTCTTGAACTTGAAAAGTTGAACGCTTGCGTAATTTCCGATGAATTGTTGAAGAAAGCCAAGCAATACGGCTTTTCGGACAAACAAATTGCTACGGCGATTCGA--ATCTGARCTTGCAGTGCGGAAACAGCGACGTGAAGCCGGAATCATTCCGATTGTCAAGCAAATCGACACTGTCGCCGGTGAATGGCCAGCTTCGACCAACTATCTGTACCTGACGTACAACGCCACCTCTCACGACATCGAATTCAAC---GAACAGATGGTGATGGTGATTGGATCGGGTGTTTACCGAATCGGAAGTTCAGTCGAGTTTGACTGGTGCGCCGTTGGATGCTTGAGAGAG--------------------------------------------------------------------------------------------------------CACATTATTAGCCAAGAAAGAGGAAAAAAGGAAACTTTTGGGGCTTTAGGAATAATTTATGCTATACTTGCTATTGGTCTTCTTGGATTTATTGTCTGAGCTCATCATATATTTACAGTGGGAATAGATGTTGATACCCGAGCCTATTTTACTTCTGCTACAATAATTATTGCAGTTCCAACAGGAATTAAAATTTTTAGTTGATTAGCTACTCTTCATGGGACCCCCTTAAATAATTCTCCTTCTTTACTTTGAGCATTAGGATTTGTATTTTTATTCACTGTAGGAGGATTAACAGGAGTAATTTTAGCAAACTCTTCTCTTGACATTGTACTTCATGATACTTATTATGTAGTAGCCCATTTTCATTATGTCTTATCGATGGGAGCTGTGTTTGCGATTATAGCAGGGTTTGTTCATTGATACCCTTTATTCACAGGACTAACAATAAATGAAAAATGATTAAAATCTCAATTTGCTATTATATTCTTAGGTGTAAATTTAACGTTTTTTCCCCAACATTTTTTAGGCCTAGCTGGTATACCTCGACGATATTCAGACTATCCTGATGCTTATACCTCTTGAAACATCGTATCAACAGTTGGATCAACTATTTCACTATTTGGAATCCTATTTTTTATTTTTATTATCTGAGAAAGC

**Limnophyes sp.** ----------------GGCCATGCACCACT--ACCCTTAATTTC-A-AGAAAACGCTATCAA-GTTGTCATACCCTATTAAGTTCGG-ACCTGGTAAGTTTTCCCGTGTTGAGTCAAATTAAGCCGCAAAATCCAAACC-ATGGTGTGAATTTCCGTCAATTCCTTTAAGTTTCAACTTTGCAACCATACTTCCCCCGGAAACT--AG-CTTT-GGTTTCCCGGAAAGCTACTGAATGCACCATGAAA--AGTAGTGACATCCAATTGCTAGCTGTCATCGTTTACAGTTAGAACTAGGGCGGTATCTAATCGCCTTCGATCCTCTAACTTTCGTTCTTGATTAATGAAAACATCCTTGGCAAATGCTTTCGCTTTAGTTAGTCTTGCGACGGTCTAAGAATTTCACCTCTCGCGCCGCAATACTAATGCCCCCAACTGCTTCTATTAATCATTACCTCT-TGATCTG-TATCAAACCAATAGAAAG-CGGCC-------------ACACAGTTGCCCATGCGACCTA-TAAGACCGAGGTCTTTTTCCATTATTCCATGCAAAAATATTCAAGGCATA-AGAGCCTGCTTTGAGCACCTTAATTTGTTCAAGGTAAAAGTAAGCCGAACTAAATAGACACCTAGCCTAGTAAAAGGCATCAATGCTATTCATTAA--GTGTTCAGTCATATAGTTCAAGCAA-CCGGAAATGATGGCA-GCCATTTACTTGATGGCGCCAC------ACCC-------G-TGCTGAACAACAATCAACTTCGAACGTTTT-AACCGCAACAATTTTAATATACGCTAGTGGAGCTGGAATTACCGCGGCTGCTGGCACCAGACTTGCCCTCCACTGGATCCTCATTAAAGGATTTATACT-TGATTCATTCCAATTACAGAAC-TAAATGATTAGTTCTATATTGTTATTTTTCGTCACTACCTCC-----TTCAATATTCCCTCAGAAGCAATGTGCACTTCTTCCTTAGTAAGACGCTGTGATCCATTAGAAAC-CATTCCCTTGGTTATTTGGCTATCAATAGGCCGACACGAAGTTCTCCGACAAATGGCTTGGTCTTTGATGGCCGGGTGTATTGTTTGGGGAGCGTGCCGGTACCTGTGTTATAGCTGTCTGATGGATTTTAAAGGTCATATTATTGCACCACTCAGAATA-CCCGATCAACAGCGGTTAAATTTGGGATACCTTTTGGACCCGTCTTGAAACACGGACCAAGGAGTCTATCTAACATGCGAGTAAATGAGGAAACTTATTTGCAGAAAAAACTTGACTGATGGGACTACGTGTGCACCTCCATCCCGTGGTATTTCTATCAACATACGCAAATGTGTATGGGAAATATACAATGAGCATGCTGGATGGGACCCGAAAGATGGTGAACTATGCCTGATCAGGTTGAAGTCAGGGGAAACCCTGATGGAGGACCGAAGCAGTTCTGACGTGCAAATCGCTGACGTATCCGCTGATCGGAAACTACGGCATCCCGAGTGACGAAGAGTTCGACGAGCACAAGCTGATGAAGCACTTTGAGTCTAACAACAAGATTTGGGTGTCGGGKCTGGTGGTCGGCGAGCTTTGCGAGACTCCCTCGCACTGGCGCCGAAAGTACAAGCTRGCCGAGTGGATGAAGAAGCACAATGTGGTGGGAATCAGCGGAATTGACACGCGAGCTCTCACTAAGAAGATTCGCGAAGACGGTACCGTTCTGGGAAAAATCATTCATCAGTCAGCG------GGTCCWTTCCCGGGT---CTGAAGTTTGAGGACCARAACCAACGGAACCTCGTTGATGAAGTGTCCATCAAAAAGCCRATAACGTACAACGCAAAAGGCTCGCCGCGAATCTGCGCAGTAGACTGCGGCTTGAAACTCAACCAGATTCGGTGCTTCGTGAAACGCGGAGCCCGCGTCGACCTCGTGCCATGGGATCACAAGCTGAATCCCGAAGACTTTGACGGACTGTTYCTTAGCAACGGCCCTGGAGATCCCGTCATGTGCAARAAGACAGTCGACAACATTCGGAAGTACCTCGGTTCGAAGAACGTGAAGCCTGTGTTCGGTATCTGCTTGGGCCATCAGCTTCTTTCYASTGCCGTTGGCTGCAAAACYTACAAAATGAAGTACGGAAATCGTGGTCACAACCTCCCGGCTCTCCACCATGGTACTAGTCGCTGCTTCATGACTTCGCAGAATCACGGATTCGCGGTTGACGCAAAAACCCTCGGGAAAGACTGGGAGCCACTTTTCACTAATCTCAACGACTTCATTCGAGTAAGCAACCACATCGGAAGCTCCATGAAGAGTGTCGGCGAGGTAATGGCGATAGGAAGGAGGTTTGAAGAAGCCTTCCAAAAGGCACTCCGAATGGTAGATGAGAATGTCAACGGCTTCGACCCAAATTTGAAGCTAGTGTCTGAGGAAGAACTCATGACACCAACTGACAAGCGCATGTTCGTGCTCGCAGCAGCACTTAAAGCCGGATACACCGTTGATAAGTTGTACGAGTTGACCAAAATAGACCGCTGGTTCCTCGAGAAGATGAAAAACATCAACGCGATTACGGTAGAGCTGGAAAAACTCAACCCTTGCGTGATTTCTAATGAATTGCTGAAGCAAGCTAAACAGTTCGGTTTTTCGGACAAGCAAATTGCGAACGCAATTCGA--ATCAGAACTCGCAGTACGAAAGCAGCGTCGCGAAGCTGGAATTCTGCCGATCGTGAAGCAAATCGACACAGTCGCTGGTGAGTGGCCTGCTTCAACAAACTACCTCTACTTGACCTACAACGCTACGACCCACGACATCGATTTCAAC---GAGGAGATGGTAATGGTCATCGGATCGGGGGTGTATCGTATCGGAAGCTCGGTCGAGTTCGACTGGTGTGCTGTGGGGTGTCTGCGAGAGTTGCGAGCGCTCGGCAAAAAAACAATCATGGTGAACTACAATCCAGAGACTGTGTCCACTGACTACGACATGTGCGATCGACTTTACTTTGAGGAAATCTC-------------------------------------CNTTTGGAGCTTTAGGTATAATTTATGCCATATTAGCTATTGGACTATTAGGATTTATTGTTTGAGCTCATCACATATTTACTGTAGGAATAGATGTTGATACTCGAGCTTACTTTACTTCTGCAACAATGATTATTGCTGTTCCTACAGGAATTAAAATTTTTAGTTGATTAGCAACTTTACACGGAACTCCTTTAAATAACTCCCCTTCCTTATTATGAGCCTTAGGGTTTGTATTTTTATTCACAGTTGGAGGATTAACAGGAGTAATTTTAGCTAACTCTTCTTTAGATATTGTTCTACATGATACTTATTATGTGGTAGCTCACTTTCATTATGTCCTTTCAATAGGAGCAGTATTCGCAATTATAGCAGGATTTGTTCATTGATATCCTCTATTTACTGGATTAGTAATAAATGAAAAATGATTAAAATCCCAATTCGCAATTATATTTTTAGGGGTAAATTTAACATTTTTTCCTCAACATTTTTTAGGATTAGCCGGAATACCTCGACGATACTCAGACTACCCTGATGCATACACTTCCTGAAATATTATTTCAACAGTAGGGTCTACTATTTCTTTATTCGGAATTTTATTCTTCATTTTTATTATTTGAGAGAGT

**Lopescladius sp.** TCCACGAACTAAGAACGGCCATGCACCACT--ACCCTTTATTTT-A-TGCAAGCGCTATAAA-GCTGGCAAACCCCATAAAGTTCTG-ACCTGGTAAGTTGTCCCGTGTTGAGTCAAATTAAGCCGCAGGTTCCATTTC--TGGTGTAAACTTCCGTCAATTCCTTTAAGTTTCAACTTTGCAACCATACTTCCCCCGGAAACT--AA-CTTT-AGTTTCCCGGAAAGCTACTGAATGCACCATGAAT--AGTAGTGACATCCAATTGCTAGTTGTCATCGTTTACTGTTTAAACTAGGACGGTATCTAATCGTCTTCGATCCTTAAACTTTCGTTCTTGATTAATGAAAACATCCTTGACAAATGCCTTCGCTTTAGTTAGTCGTACGACGGTCTAAGAATTTCACCTCTCGCGCCGTAATACTAATGCCCCCGACTGCTTCTATTAATCATTACCTCT-TGATCTG-TATCAAACCAATAGAAAG-CGGCA-------AGTGCAATATTTCATACACATCA-CCTA-TAAGACCGAGGTCTTTTTCCATTATTCCATGCAAAAATATTCAAGGCATATAGAGCCTGCTTTGAGCACCTTAATTTGTTCAAGGTAATAGTAAGCCGGACTAAACGGACGCCTAGCCTAATGAAAGGCATCAGCGCCGTTCATTAA--GTGCCCGGTCATATAGTCCAAGTCA-CCGAAAACGACAGCA-ACCA-TTACTTGATGGTGCCGC------GCTC-------G-AACCGGACAAAAATCAACTTCGAACGTTTT-AACCGCAACAATTTTAACATACGCTAGTGGAGTCGGAATTACCGCGGCTGCTGGCACCGAACTTGCCCTCCACTTGATCCTCATATATGAGTTTAGAAT-AGATTCATTCCAATTACAGAACATAGTTAACTAGTTCTATATTGTTATTTTTCGTCACTACCTCC-----TTCAATATTCCTTCAAACGCAATGTGCACTTTTCTCTCAGTAAGACACTGTGATCCATTAGAAATTCGTCTACACGGTYATTGGGCTATCAATGGGCCAGTATGAAGTTCGTTGGCAAATGGCAAGATCTTTAACGGTCATGTGTATTGCCCATCGAGCGTACTGTTACTCGTA--ATAGCCGTCTGATGGATTTTAAAAGTCACATCACTGCATCACCCAGGATG-CTCGATCAACAGTGGTTAAATTTGCGATACCTTTGGGACCCGTCTTGAAACACGGACCAAGGAGTCTATCTAGTGTGCAAGTAAATGGGGAAACCTATTTGCGTAAAAAACTTGACTTATGGGATTACGAGTTCTCCTCCATCCCGGGGTATTTTCATCAACATATGCAAATGTATATGGAAAATATACCATGAGCATACTGGATATGACCCGAAAGATGGTGAACTATGCCTGATCAGGTTGAAGTCAGGGGAAACCCTGATGGAGGACCGTAGTTGTTCTGACGTGCAAATCG---------------ATTGGAAATTATGGCATTCCCGATGACAATGAATTTGACGAGTTTAAATTGATCAAGAACTTTGAGTCGAACGGCAAGATCTGGGTGTCGGGATTGGTGGTGGGCGAGCTGTGCGAAACGCCGTCGCATTGGCGTCTGAAATTCACGCTGTCGGAGTGGATGAAGAAGCACAAAATCCCCGGCATCAGCGGAGTCGACACGCGCGCCTTGACCAAGAAAATTCGGGAGAATGGAACCATCTTGGGGAAGATCATTCAGCAGCCGTCG------GGGCCCTTCCCCGGT---CTTGAGTTCAAGGACCAGAACGAGAGGAACTTGGTGGACGAAGTGTCCACAAAGAAAGTCACCGTCTACAATCCCAATGGCTCCCCGAGAATCTGCGCTGTGGACTGCGGCCTGAAACTCAATCAAATCCGATGCTTTGTSAAACGAGGAGCTCGCGTTGATGTCGTKCCATGGAACCATGCCTTGGACGGAAAGACTTTTGACGGGCTGTTCCTGTCGAACGGCCCTGGTGATCCCGTTATTTGCCAAGCTACCGTTAAGAACATTCAAACGTTTCTGAAGTCGTCGACTGTAAAACCGATTTTCGGCATTTGCCTCGGCCACCAACTTCTGTCAACAGCGATTGGATGCAAAACGTACAAAATGAAGTACGGAAATCGCGGGCACAACTTGCCCGCCATCCACCACGGCTCCAATCGCTGCTTCATGACCTCGCAGAACCACGGATTTGCTGTCGATGTCACGAGCATTCACGCCGACTGGGAGCCGCTTTTCACCAAC---------TTCATTCGAGTCAGTAAACACATCGGCAGTTCCATGAAGAGCGTCGGCGAGGTGATGGCCATTGGCCGCAAGTTCGAGGAAGCTTTCCAGAAAGCCCTGCGCATGGTCGACGAAAACGTCACCGGCTTCGATCCCAACTTGAAGGCGCCAAAAGACGAGGAGCTAAAAATGCCGACGGATAAGCGAATGTTTGTGCTGGCRGCGGCACTGAAAGCTGGCTATTCCGTCGATCGCCTGTATGACCTGACCAAGATCGATCGCTGGTTCCTCGAGAAGATGAAGAACATCATCTCGATCACWGTCGAGCTGGAGAAGCTCAGCTCGATCATGCTCCCCAACGACCTCCTCCATCGCGCCAAAAAGTTTGGGTTTTCCGACAAGCAAATTGCGCAGTACACCAAC--AACTGAGCTCGCGGTGAGGAAGCAACGACGGGAAGTGGGMATCCTCCCGTTTGTGAAGCAAATCGACACCGTMGCTGGGGAGTGGCCAGCGACAACCAACTATTTGTACTTGACGTACAACGCCAACGAGCACGACATCAAGTTCAAC---GGCAAGATGATGATGGTGATCGGTTCGGGAGTTTATCGCATCGGCAGCTCCGTGGAGTTTGACTGGTGCGCTGTGGGATGTCTGCGAGAGCTGAGGAAGCTGGGCAAGAAGACCATCATGGTGAATTACAATCCCGARACGGTCTCGACGGACTACGACATGTGCGACCGACTGTACTTCGAGGAAATCTCTTT---------------AAAAGAGGTAAAAAGGAAACTTTTGGTACATTGGGAATAATTTATGCAATATTAGCTATTGGGTTATTAGGTTTCATTGTTTGGGCTCATCATATATTTACTGTTGGAATAGATGTAGATACTCGTGCTTACTTTACATCCGCAACTATAATTATTGCTGTTCCTACAGGAATTAAAATTTTTAGTTGACTAGCTACTTTACACGGAACACAATTAAATTACTCTCCTTCATTACTTTGAACTTTAGGTTTTGTATTCTTATTTACTGTTGGAGGATTAACTGGGGTAATTCTGGCTAATTCCTCTTTAGATATTGTTCTTCATGACACATATTATGTTGTTGCTCATTTTCATTATGTTTTATCTATAGGAGCTGTTTTTGCTATTATAGCCGGCTTTATTCATTGATATCCACTATTTACAGGATTAACAATAAATAAAAATTGATTAAAATCCCAATTTATTGTTATATTTTTAGGAGTAAACTTAACTTTTTTCCCTCAACATTTTTTAGGATTAGCTGGTATACCTCGACGTTACTCTGATTATCCTGATGCTTACACTTCTTGAAATGTAATTTCTACATTAGGGTCTACTATTTCATTACTAGGAATTTTAATATTTTTATTTATTATTTGAGAAAGT

**Maoridiamesa stouti** TATTCACGACTAAGACGGCCATGCACCACT--ACCCTTAATTTC-G-AGAAAGAGCTATTAA-TCTGTCTTACCCTATTAAGTTCGG-ACCTGGTAAGTTTTCCCGTGTTGAGTCAAATTAAGCCGCAGGCTCCAATCC--TGGTGTGCCCTTCCGTCAATTCCTTTAAGTTTCAACTTTGCAACCATACTTCCCCCGGAAACT--AG-CTTT-GGTTTCCCGGA-AGCTACTGAATGCGCCAT-AAT--AGTAGCGACATCCAATTGCTGGCTGTCATTGTTTACAGTTAGAACTAGGGCGGTATCTAATCGCCTTCGATCCTCTAACTTTCGTTCTTGATTAATGAAAACATCCTTGGCAAATGCTTTCGCTTTAATTAGACTTACGACGGTCTTAGAATTTNNNNNNNNNNNNNNNNNNNNNNNNNNNNNNNNNNNNNNNNNNNNNNNNNNNNNNNN---------NNNNNNNNNNNNNNNNNNNNNNNNNNNNNNNNNNNNNNNNNNNNNNNNNNNNNN-NNNN-NNNNNNNNNNNNNNNNNNNNNNNNNNNNNNNNNNNNNNNNNNNNNNNNNN-NNNNNNNNNNNNNNNNNNNNNNNNNNNNNNNNNNNNNNNNNNNNNNNNNNNNNNNNNNNNNNNNNNNNNNNNNNNNNNNNNNNNNCTATTCATTAATAGTGTTCAGTCATATAGTCCAAGTAA-TCGGAAATGATTACA-ACCA-TAATTTGATGGTGTAAC------ACCT-------G-TACTGGACAATAATCAACTTCGAACGTTTT-AACCGCAACAATTTTAATATACGCTAGTGGAGCTGGAATTACCGCGGCTGCTGGCACCAGACTTGCCCTCCACTAGATCCTTGTTAAAGGATTTATAAT-TAACTCATTCCAATTACAGAACATAGTTAACTAGTTCTATATTGTTATTTTTCGTCACTACCTCC-----TTCAATACTTCGTTAAACATAGTGTGCACTTTTCTCTTAGTAAGACGTTGTGATCCATTAGAAATTTGTATAGGATATTATTGGGCTATCAATAGACTGTTATGAAGTTCATCAGTAAACAGTAAGATCGTTTATGATTTTGCATATTATCTGGTGAGCATAACATGTTCTG--GTGTAGCTGTCTGATGGATTTTAAAGGTTATATCATTGCATTACCC-GGATAACCCGATCAACAACGGTTAAATTTGGGATACCTTTGGGACCCGTCTTGAAACACGGACCAAGGAGTCTGTCTAGTATGCAAGTAAATGGGGAAACCTATTTGCGTAAATAACTTGACTGATGGGATTACGAATGTTCCTCCATCCCGGGGTATTTTTATCAACATATA-AAAT-TATATGGGAAATATACCATGAGCATACTGGATATGACCCGAAAGATGGTGAACTATGCCTGATCAGGTTGAAGTCAGGGGAAACCCTGATGGAGGACCGAAGCAATTCTGACGTGCAAATCGTTGACTTATCCGTTAATTGGAAATTATGGCATTCCT---GATGATGCTGTGGATGAACATCAGTTGTCAATTAATTTTGAATCGAATAATAAAATATGGATATCAGCGTTGGTTGTTGGTGAAATGTGTGAGACACCATCACATTGGAGATTAAAATTAAAGTTATCTGAATGGATGACTAAACATGGAATACCTGGAATTTGTGGCATCGATACACGTGCACTGACCAAGAAGATACGTGAAAATGGTACAGTTTTGGGTAAAATTCTTCAACATCCAGTG------GCTGGTGTCTCTGAT---TTTATTTTTAATAATCAAAATCATAGRAATCTTGTGGATGAAGTTTCAATTAAAAAGCCAGTAACATACAATCCATCTGGAACACCAAGAATATGTGTTGTCGATTGTGGTTTAAAGTTAAATCAACTACGTTGTTTCATAAAACGTGGTGCACGTGTTGATGTTGTTCCATGGAATCATCAGTTAAATGTTACTGAATTTGATGGATTATTTTTATCAAACGGTCCTGGTGATCCAATTTTGTGTCGCAAAACTGTTGAYAATATAAGAAATGTATTGTCAGCGAATGTTGTGAAACCAATATTTGGTATTTGCTTGGGACATCAGCTCTTGTCCACAGCAATTGGTTGTAACACTTACAAGCTGAAATATGGTAATCGTGGACATAACTTGCCATGTCTTCATAATGGAACAAATCGTTGCTTCATGACATCCCAAAATCATGGATTTGCTGTTGATGTCACTAAAATTGATAAGAATTGGGAAGTTCTCTTTACAAATGCAAATGAT---------------------ATTGGTAGTTCNATGAAANGTGTTGGTGAGGTGATGGCAATTGGACGAAAATTTGAGGAAGCATTTCAGAAAGCATTAAGAATGGTTGATGAAAAYATAAATGGCTTTGATCCAAATATWAAAGATATAAATGATGATGAATTAACAGCACCAACTGATAAGAGAATGTTTGTTCTTGCTGCATCACTTAAAGCTGGCTATTCAGTTGATAAACTTTATGATCTTACAAAAATTGATCGTTGGTTTTTACAAAAAATGAAAAATATTAATGATTTAACAATGAAATTGGAAAATTTAAGY---TCACCATTGTCAGTAGATATGCTTGATAATGCAAAAAAAATGGGATTTTCTGATAAACAAATAGCARTTGCAATTAAA--TTCAGAATTGGCAGTAAGAAAGCAACGTCGTGAAAATAAAATATTACCATTTGTTAAGCAAATTGATACTGTTGCTGGTGAATGGCCAGCAACAACAAATTATCTTTACTTTACATATAATGCATCGGAACATGATATTGAATTTGAA---CATGAACATGTAATGGTTATTGGTTCTGGTGTTTATCGTATTGGAAGCTCAGTGGAATTTGATTGGTGCGCATGTGGATGTTTAAGGGAATTGAGAAAAATGGGACGTAAAACAATTATGGTAAATTATAATCCAGAAACAGTATCAACTGATTATGATATGTGTGATCGTCTTTATTTTGAAGAAATATCATT-------------------------------------------CTTTAGGAATAATCTATGCTATACTTGCAATTGGATTATTAGGATTTGTAGTTTGAGCTCACCATATATTTACAGTAGGAATAGACGTTGATACTCGAGCTTATTTTACATCTGCTACTATAATTATTGCAGTACCAACAGGAATTAAAATTTTTAGTTGACTTGCAACACTTCATGGAACACAGTTAAATTACTCCCCTTCTCTAATTTGAGCTTTAGGATTTGTTTTTCTTTTCACGGTAGGAGGTCTTACAGGAGTTGTATTAGCTAATTCTTCTATTGATATTGTTCTTCATGATACATATTATGTTGTTGCTCATTTTCATTATGTTCTCTCTATAGGAGCTGTATTTGCTATTATGGCAGGTTTTGTCCACTGATACCCTCTTTTTACAGGATTAACTTTAAATGAAGAATGATTAAAGTCTCAATTTGCAATTATATTTTTAGGAGTAAATTTAACATTTTTTCCACAACATTTTTTAGGATTAGCAGGAATACCTCGACGATATTCAGATTACCCAGATGCTTACACTTCATGAAATGTAGTGTCTACAGTAGGATCAACTATTTCTTTATTTGGAATTATTTTCTTCTTATTTATTATTTGAGAAAGA

**Mapucheptagyia brundini** ----------------GGCCATGCACCACT--ACCCTTAATTTC-G-AGAAAGAGCTATTAA-TCTGTCTTACCCTATTAAGTTCGG-ACCTGGTAAGTTTTCCCGTGTTGAGTCAAATTAAGCCGCAGGCTCCACTCCGTTGTGGTGCCCTTCCGTCAATTCCTTTAAGTTTCAACTTTGCAACCATACTTCCCCCGGAAACT--AG-CTTT-GGTTTCCCGGA-AGCTACTGAATGCGCCAT-AAT--AGTAGCGACATCCAATTGCTGGCTGTCATTGTTTACAGTTAGAACTAGGGCGGTATCTAATCGCCTTCGATCCTCTAACTTTCGTTCTTGATTAATGAAAACATCCTTGGCAAATGCTTTCGCTTTAGTTAGTCTTACGACGGTCTAAGAATTTCACCTCTCGCGCCGTAATACTAATGCCCCCAACTGCTTCTATTAATCATTACCTCT-TGATCTG-TATCAAACCAATAGAAACTAAATA---------------------------------TA-TAAGACCGAGGTCTTTTTCCATTATTCCATGCAAAAATATTCAAGGCGTATATAGCCTGCTTTGAGCACCTTAATTTGTTCAAGGTAATAGTAAGCTGAACTAAATAGACG-TTAACCTAATTAAAGGTGTCA-CGCTATTCATTAATAGTGTTCAGTCATATAGTCCAAGTAAATCGGAAATGATGGCA-ACCA-TAACTTGATGGTGCCAC------ACCC-------G-TACTGGACAATAATCAACTTCGAACGTTTT-AACCGCAACAATTTTAATATACGCTAGTGGAGCTGGAATTACCGCGGCTGCTGGCACCAGACTTGCCCTCCACTAGATCCTTGTTAAAGGATTTATAAT-TAACTCATTCCAATTACAGAACATAGTTAACTAGTTCTATATTGTTATTTTTCGTCACTACCTCC-----TTCAATACTCTTTTAAAAGCGGTGTGCACTTTTCTTTTAGTAAGACGTTGTGATCCATTAGGAATTCTTTTAGGATATTATTTGGCTATCAATAGACTGATATGAAGTTCATTATTACACAATATTATCGTTTATGGTTATATATAGTGACTAATGAGCATATTATGTTCTGT---GTAGCTGCCTGATGGATTTTAAAGGTCATATCATTGCATTATCCCAAATAACCCGATCAACAACGGTTAAATTTGGGATACCTTTAGGACCCGTCTTGAAACACGGACCAAGGAGTCTATCTAGTATGCAAGTAAATGGGGAAACCTATTTGCATAAATAACTTGACTGATGGGATTACGATTGATCCTCCATCCCGGGGTATTTTTATCAACATATA-AAAT-TATATGAAAAATATACCATGAGCATACTGGATATGACCCGAAAGATGGTGAACTATGCCTGATCAGGTTGAAGTCAGGGGAAACCCTGATGGAGGACCGAAGCAATTCTGACGTGCAAATCGTTAACTTATCCATTGATTGGAAATTATGGCATTCCC---GAAGAAGAGCTGGATAATTTTAAGATTTCCAAAYATTTTGAATCGAATAATAAAATTTGGACATCAGCATTAATTGTAGGTGAGCTTTGTGAATCGCCATCACATTGGCGTTACAGATATAAATTGTCTGAATGGATGAAGAAGTATAATATTCCTGGGATAAGCGGAATTGACACGCGAGCTTTAACAAAAAAAATTCGTGAAAATGGAACTGTACTAGGAAAAATTATCCAAGGGAATGAT------ATCATTATTCCAGAAACGTTGAATTTTAGTGATCAGAATGAGAGAAATCTTGTGAACGAAGTTTCTACAAAGCATAAGATAACGTACAATGAAACTGGATTCCCACGTATTTGTGCTGTAGATTGTGGACTTAAACTCAATCAAATACGTTGTTTCGTTAAACGGGGAGCCTGTGTAGATGTTGTTCCGTGGGATTATAACTTAAACAATAATGATTATGATGGATTATTTATAAGTAACGGTCCTGGAGATCCCATCATGTGTGAGAAGACAGTGAAAAACATTCAAGAAATTCTAAGTCTGAAAAATACGAAACCAATATTTGGTATTTGTTTGGGTCATCAACTTCTCGCTACAGCTATTGGTTGTAAGACTTACAAAATGAAATATGGGAATCGTGGTCATAACCTGCCATGTATTCATCACGGCACTAATCGTTGYTTTATGACTTCTCAAAACCATGGATTTGCAGTTGATGTTAATAACATGAACTCTAATTGGACATCATTATTTACTAATGCAAATGATTTTATTCGTGTCAGCAAAAATATTGGAAGCTCTATGAAGAGTGTTGGCGAAGTGATGGCCATTGGAAGAAAGTTTGAAGAAGCATTTCAGAAAGCACTTCGAATGGTTGATGAAAATGTGAATGGATTTGATCCTAACTTGAAGCCTGTTAAAGACGAGGAGTTGGAAACACCTACTGATAAGCGCATGTTTGTTCTTGCTGCTGCATTGAAAGCAGGTTATACTATTGATAAACTTTACGATCTTACCAAAATCGATCGTTGGTTTTTAGAAAAGATGAAGAACATTATTGATGTCACTTTAGAATTAGAACATTTGAAG---TCTAAGCTTCCAATTGATGCTTTGAGAAATGCAAAATCTCTTGGGTTTTCTGATAAGCAAATAGCAAATTGTATTAAA--GTCAGAGCTAGCAGTAAGAAAGTATCGACGTGATAACAAAATATTTCCATTTGTTAAGCAAATAGATACTGTTGCTGGTGAATGGCCTGCATCAACCAATTATTTATACTTAACATATAACGCTTCTGAACATGATATTACTTTTGAT---GATCAATATGTTATGGTTATTGGATCTGGCGTCTATCGCATTGGAAGCTCAGTGGAATTCGATTGGTGTGCTTGTGGATGTCTGACAGAATTGAGGAAGTTAGGTAAAAAGACAATTATGGTGAATTATAATCCTGAAACTGTTTCCACCGACTACGATATATGTGACCGTCTTTACTTTGAGGAGATATCATTCATATTATTAGCCAAGAAAGAGGAAAAAAAGAAACTTTCGGGTCTCTAGGGATAATTTATGCTATACTAGCAATTGGTCTTTTAGGTTTTGTTGTTTGAGCTCATCATATATTTACTGTAGGTATAGATGTTGATACCCGAGCTTATTTCACTTCGGCTACTATAATTATTGCTGTACCCACAGGAATTAAAATTTTCAGATGATTAGCAACACTACACGGTACCCAACTTACTTACTCTCCTTCACTTTTATGGGCCTTAGGATTTGTTTTTTTATTTACCGTAGGAGGTCTCACAGGGGTTATTTTAGCTAATTCTTCTATCGATATTGTTCTTCATGACACTTATTATGTAGTAGCCCATTTCCACTACGTTCTTTCTATAGGAGCCGTGTTCGCTATTATAGCTGGATTCATCCATTGGTACCCACTATTCTCAGGACTCACATTAAACGAAGAATGATTAAAGTCTCAATTTGTTATCATATTTTTAGGGGTTAATTTAACCTTTTTTCCTCAACATTTTTTAGGATTAGCAGGTATACCCCGACGATATTCTGATTACCCTGATGCTTACACATCTTGAAATGTAGTATCCACAATCGGATCTACCATCTCCTTATTCGGTATCATTTTCTTTTTATTTATTATCTGAGAAAGA

**Mesosmittia sp.** ---------------CGGCCATGCACCACT--ACCCTTAATTTC-A-AGAAAACGCTATCAA-GTTGTCATACCCTATTAAGTTCGG-ACCTGGTAAGTTTTCCCGTGTTGAGTCAAATTAAGCCGCAAAATCCAAACC-ATGGTGTGAATTTCCGTCAATTCCTTTAAGTTTCAACTTTGCAACCATACTTCCCCCGGAAACT--AG-CTTT-GGTTTCCCGGAAAGCTACTGAATGCACCATGAAA--AGTAGTGACATCCAATTGCTAGCTGTCATCGTTTACAGTTAGAACTAGGGCGGTATCTAATCGCCTTCGATCCTCTAACTTTCGTTCTTGATTAATGAAAACATCCTTGGCAAATGCCTTCGCTTTAGTTAGTCTTGCGACGGTCTAAGAATTTCACCTCTCGCGCCGCAATACTAATGCCCCCGACTGCTTCTATTAATCATTACCTCT-TGATCTG-TATCAAACCAATAGAAAG-CGGCC-------------GCACAGTTGCCCATACGACCTA-TAAGACCGAGGTCTTTTTCCATTATTCCATGCAAAAATATTCAAGGCATA-AGAGCCTGCTTTGAGCACCTTAATTTGTTCAAGGTAAAAGTAAGCCGAACTAAATAGACACCTAGCCTAGTAAAAGGCATCAATGCTATTCATTAA--GTGTTCAGTCATATAGTTCAAGCAA-CCGGAAATGATAGCA-GCCATTTACTTGATGGCGCTAC------ACCC-------G-TGCTGAACAACAATCAACTTCGAACGTTTT-AACCGCAACAATTTTAATATACGCTAGTGGAGCTGGAATTACCGCGGCTGCTGGCACCAGACTTGCCCTCCACTGGATCCTCATTAAAGGATTTATACT-TGATTCATTCCAATTACAGAAC-TAAATGATTAGTTCTATATTGTTATTTTTCGTCACTACCTCC-----TTCAATATTCCCTCAAAAGCAATGTGCACTTCTTCCTTAGTAAGACGCTGTGATCCATTAGAAAC-CATTCCAATGGTTATTTTGCTATCAATAGGCCGACACGAAGTTCTCCGACAAATGGCTTGGTCTTTGATGGCCGGGTGTATTGTTTGGGGAGCGTGCCGGTACCTGTGTTATAGCTGTCTGATGGATTTTAAAGGTCATATTATTGCACCACTCAGAATAACCCGATCAACAGCGGTTAAATTTGGGATACCTTTTGGACCCGTCTTGAAACACGGACCAAGGAGTCTATCTAACATGCGAGTAAATGAGGAAACTTATTTGCAGAAAAAACTTGACTGATGGGACTACGTGCGTACCTCCATCCCGTGGTATTTCTATCAACATACGCAAATGTGCATGGGAAATATACAATGAGCATGCTGGATGGGACCCGAAAGATGGTGAACTATGCCTGATCAGGTTGAAGTCAGGGGAAACCCTGATGGAGGACCGAAGCAGTTCTGACGTGCAAATCGCTGACGTATCCGCTGATCGGCAACTACGGCATCCCGAGTGACGAAGAGTTCGACGAGCACAAGTTGATGAAGCACTTCGAGTCGAACAACAAGATTTGGGTGTCGGGCCTGGTGGTCGGTGAGCTTTGCGAGACTCCCTCGCACTGGCGCCGGAAGTACAAACTCGCCGAGTGGATGAAGAAGCACAATGTGGTCGGTATCAGCGGCATTGACACGCGAGCCCTCACCAAGAAGATTCGGGAAGACGGAACCGTGCTGGGCAAAATAATCCATCAGTCGGCG------GGTCCTTTTCCGGGT---CTGAAGTTCGAGGACCAAAACCAACGTAACCTCGTGGATGAAGTGTCCATCAAGAAGCCGATAACTTACAACGCAAAAGGCTCGCCAAGAATCTGCGCAGTAGACTGCGGCTTGAAACTCAACCAGATTCGGTGCTTCGTCAAACGCGGTGCCCGTGTCGACCTCGTGCCATGGGATCACAAGCTGAATCCCGAAGACTTTGACGGTCTTTTTCTAAGCAACGGCCCTGGTGATCCCGTCATGTGCAAGAAGACTGTCGAGAACATTAAAAAGTACCTCGGTTCGAAGAACGTAAAGCCGGTTTTCGGCATCTGCTTGGGCCATCAGCTCCTCTCCAGTGCCGTCGGATGCAAAACCTACAAAATGAAGTACGGAAATCGTGGTCACAACCTCCCAGCGCTCCACCACGGCACCAATCGCTGCTACATGACCTCGCAGAATCATGGATTCGCGGTCGACGCAAATACCATCGGGAAAGACTGGGAGCCTCTCTTCACCAACCTCAACGACNNNNNNNNNNNNNNNNNNNNNNNNNNNNNNNNNNNNNNNNNNNNNNNNNNNNNNNNNNNNNNNNNNNNNNNNNNNNNNNNNNNNNNNNNNNNNNNNNNNNNNNNNNNNNNNNNNNNNNNNNNNNNNNNNNNNNNNNNNNNNNNNNNNNNNNNNNNNNNNNNNNNNNNNNNNNNNNNNNNNNNNNNNNNNNNNNNNNNNNNNNNNNNNNNNNNNNNNNNNNNNNNNNNNNNNNNNNNNNNNNNNNNNNNNNNNNNNNNNNNNNNNNNNNNNNNNNNNNNNNNNNNNNNNNNNNNNNNNNNNNNNNNNNNNNNNNNNNNNNNNNNNNNNNNNNNNNNNNNNNNNNNNNNNNNNNNNNNNNNNNNNNNNNNNNNNNNNNNNNNNNNNNNNNNNNNNNNNNNNNNNNNNNNNNNNNNNNNNNNNNNNNNNNNNNNNNNNNNNNNNNNNNNNNNNNNNNNNNNNNNNNNNNNNNNNNNNNNNNNNNNNNNNNNNNNNNNNNNNNNNNNNNNNNNNNNNNNNNNNNNNNNNNNNNNNNNNNNNNNNNNNNNNNNNNNNNNNNNNNNNNNNNNNNNNNNNNNNNNNNNNNNNNNNNNNNNNNNNNNNNNNNNNNNNNNNNNNNNNNNNNNNNNNNNNNNNNNNNNNNNNNNNNNNNNNNNNNNNNNNNNNNNNNNNNNNNNNNNNNNNNNNNNNNNNNNNNNNNNNNNNNNNNNNNNNNNNNNNNNNNNNNNNNNNNNNNNNNNNNNNNNNNNNNNCATATTATTAGTCAAGAAAGTGGAAAAAAGGAAACTTTTGGGGCATTAGGAATAATTTATGCTATATTAGCAATTGGGTTATTAGGATTTATTGTATGGGCCCATCATATATTTACTGTTGGCATAGATGTAGACACCCGAGCATATTTCACCTCCGCCACTATAATTATTGCAGTACCAACTGGGATTAAAATTTTTAGTTGATTAGCCACTCTTCATGGAACTCCTTTAAATAATTCACCTTCTTTATTATGAGCTTTAGGATTTGTATTTCTATTTACTGTAGGAGGTTTAACTGGAGTGATTTTAGCTAATTCTTCTCTTGATATTGTCCTTCATGACACTTATTATGTAGTTGCTCATTTTCATTATGTCCTTTCAATGGGGGCTGTGTTTGCTATTATAGCAGGGTTTGTTCATTGATACCCTTTATTTACTGGATTAGTAATAAATGAAAAATGATTAAAATCTCAATTTGCTATTATATTTTTAGGTGTTAATTTAACATTTTTCCCTCAACACTTTTTAGGTCTCGCAGGAATACCTCGGCGTTATTCTGATTATCCTGATGCTTATACTTCTTGAAATATTGTTTCAACAGTAGGGTCTACTATTTCTTTATTTGGTATTTTATTTTTTATTTTTATTATCTGAGAAAGT

**Monodiamesa sp.** ----CGAACTAAGAACGGCCATGCACCACT--ACCCTTAATTTC-A-AGAAACCGCTGTCAA-GGTGTCTAACCCTATTAAGTTCGG-ACCTGGTAAGTTTTCCCGTGTTGAGTCAAATTAAGCCGCAAATTCCAGACC-TGGTGGTGCCCTTCCGTCAATTCCTTTAAGTTTCAACTTTGCAACCATACTTCCCCCGGAAACT--AG-CTTT-GGTTTCCCGGAAAACTACTGAATGCACCATGAAT--AGTAGTGACATCCAATTGTTAGCTGTCATAGTTTACAGTTAGAACTAGGGCGGTATCTAATCGCCTTCGATCCTCTAACTTTCGTTCTTGATTAATGAAAACATCCTTGGCAAATGCTTTCGCTTTAGTTAGTCTTACGACGGTCTAAGAATTTCACCTCTCGCGCCGTAATACTAATGCCCCCAACTGCTTCTATTAATCATTACCTCT-TGATCTG-TATCAAACCAATAGAAAG-CGACC--CAGAGCACCCTTGCGAGCACCTACTGAG-CCTA-TAAGACCGAGGTCTTGTTCCATTATTCCATGCAAAAATATTCG-GGCATA-CGAGCCTGCTTTGAGCACCTTAATTTGTTCAAGGTAATAGTAAGCCGAACTAAATAGACACCTAGCCTAATGAAAGGCATCAGTGCTATTCATTAA-AGTGTTCGGTCATATAGTCCAAGTCA-GCGGAAATGATGGCA-ACCATTTACTTGATGGTGCCAC------ACCC-------G-TACTGGACAATAATCAACTTCGAACGTTTT-AACCGCAACAATTTTAATATACGCTAGTGGAGCTGGAATTACCGCGGCTGCTGGCACCAGACTTGCCCTCCACTTGATCCTCATTAAAGGATTTATACT-TGATTCATTCCAATTACAGAACATAGTTAACTAGTTCTATATTGTTATTTTTCGTCACTACCTCC-----TTCAATACTCCTTTAAACGTAGTGTGCACTTTTCCTTTAGTAAGACGCTGTGATCCATTAGAAATTCATACCAATGGTTATTGGGCTATCAATAGGCCATTATGAAGTTCAACGGCAAATGGCTCGATCTTTAATGGTTGGGTGTATTGCCTGGTGAGCATAATGATACCTGTA--ATAGCTGTCTGATGGATTTTAAAGGTCATATCATTGCATTACTCTGGATAACCCGATCAACAGCGGTTAAATTTGGGATACCTTTGGGACCCGTCTTGAAACACGGACCAAGGAGTCTATCTAGTATGCAAGTAAATGGGGAAACCTATTTGCATAAAAAACTTGACTGATGGGATTACGAGTACTTCTCCATCCCGGGGCATTTTCATCAACATATGCGAATGTATATGGGAAATGTACCATGAGCATACTGGATATGACCCGAAAGATGGTGAACTATGCCTGATCAGGTTGAAGTCAGGGGAAACCCTGATGGAGGACCGAAGCAATTCTGACGTGCAAATCG---------------------------------------------------GATGAAAACCAACTGATTAAGCATTTTGAATCCAATAACAAGATATGGGTGTCAGGCTTGATTGTTGGCGAAATTTGTGACACACCATCGCATTGGCGTCAGAAATATAAGCTCTCTGAATGGATGAAAAATCATAATATTCCTGGAATAAGTGGCGTTGACACCAGAGCTCTCACCAAGAAAATTCGTGAGAATGGAACCGTTTTGGGGAAAATTATTCAACAGCCATCA------GGACCATTCCCTGGA---TTGGAATTTATAGATCAGAACCAGCGTAATCTTGTGAATGAAGTGTCTATCATAAAGACCATCACTTACAACCAATCTGGATCGCCTCGTATTTGTGCTATTGATTGTGGATTGAAATTGAATCAAATTCGTTGTTTCATCCAACGTGGAGCTCGGGTTGATGTTGTGCCATGGGATCAAGAAGTGAATCCAAACAATTTCGATGGACTGTTCCTCAGCAATGGACCAGGGGATCCCGTTATGTGCAAAAAGACTGTGAAGAACATTCAAACATTTTTAGCATCGCCTAAAGTCAAGCCCATCTTTGGTATTTGCTTAGGCCACCAGCTCTTATCAACTGCAATTGGTTGTAAAACTTACAAAATGAAATATGGAAATCGTGGC------------------------------------------------------------------------------------------------------------------------------TTCATCAGGGTCAGTAAACACATTGGAAGTTCCATGAAAAGTGTTGGTGAAGTGATGGCGATTGGAAGAAAGTTTGAAGAAGCTTTCCAGAAAGCCTTGCGAATGGTCGATGAAAATGTCAACGGATTTGATCCGAACTTGAAGCCAGTTAAAGATGAAGAGCTGACTACACCAACTGATAAAAGGATGTTTGTCCTTGCCGCAGCTCTCAAAGCTGGTCACACTGTTGATCGTTTGTATGATCTGACGAAAATCGATCGCTGGTTCTTGCAAAAAATGAAAAACATCATTGATATCACACTGGAGCTAGAGAAACTAAAT---TGCGTCGTACCCGATTCGCTTTTATATCAAGCTAAAAAGTATGGGTTTTCAGATAAGCAAATTGCATCATACATCAAA--CTCTGAGTTGGCAGTTAGAAAACAGCGACGTGAATGCGGATTTTTGCCATTTGTAAAACAGATTGATACAGTTGCTGGTGAATGGCCAGCAACAACCAATTATCTGTATTTGACCTACAATGCTAGTGAACATGATATAGAATTTAAC---GATCAAATGGTAATGGTTATAGGATCGGGCGTGTATCGTATTGGAAGTTCAGTGGAGTTCGATTGGTGCGCTGTGGGATGTTTAAGAGAGCTGAGAAAATTGGGCAAGATGACAATAATGGTGAATTATAATCCAGAAACTGTGTCCACTGATTACGACATGTGCGATCGTTTGTATTTTGAAGAAATCTCATTCATATTATTAGTCAAGAAAGAGGGAAAAAGGAAACATTCGGGGCTTTAGGAATAATTTATGCAATATTAGCTATTGGTTTATTAGGTTTTGTTGTTTGAGCTCACCATATATTTACAGTAGGTATAGATGTTGATACTCGAGCCTATTTTACTTCAGCTACCATAATTATTGCTGTACCTACAGGAATTAAAATTTTTAGATGATTAGCTACTCTCCATGGAACACAATTAAATTACTCCCCTTCTCTTTTATGAGCTTTAGGATTTGTTTTTTTATTTACAGTTGGGGGACTAACAGGAGTTGTACTTGCTAATTCTTCTATTGACATTGTTCTTCACGACACATATTATGTAGTTGCCCATTTCCATTATGTTTTATCTATAGGAGCCGTATTTGCTATTATAGCAGGATTTGTCCACTGATATTCATTATTTACAGGATTAACAATAAATGAAGAATGATTAAAATCTCAATTTATAATTATATTTGTAGGAGTAAATTTAACATTTTTTCCCCAACATTTTCTTGGATTAGCAGGTATACCACGACGATACTCGGATTATCCAGATGCTTACACTTCATGAAATGTTATTTCTACCTTAGGATCATCAATTTCTTTAGTAGGGGTTTTATTTTTTCTTTTTATTATTTGAGAAAGT

**Nanocladius sp.** ----------------------------CT--ACCCTTTTTTTC-A-TGAAAGCGCTGTCAA-GCTGTCATACACAAAAAAGTTCTG-ACCTGGTAAGTTTCCCCGTGTTGAGTCAAATTAAGCCGCAAATTCCATTTC--TGGTGTGAAGCTCCGTCAATTCCTTTAAGTTTCAACTTTGCAACCATACTTCCCCCGGAAACT--AG-CTTT-GGTTTCCCGGAAAGCTACTGAATGCACCATGAAA--AGTAGTGACATCCAATTGCTAGCTGTCATCGTTTACAGTTAGAACTAGGGCGGTATCTAATCGCCTTCGATCCTCTAACTTTCGTTCTTGATTAATGAAAACATCCTTGGCAAATGCTTTCGCTTTAGTTAGTCTTGCGACGGTCTAAGAATTTCACCTCTCGCGCCGCAATACTAATGCCCCCAACTGCTTCTATTAATCATTACCTCT-TGATCTG-TATCAAACCAATAGAAAC-CGGTA------------GAACCCTTGCGAGAACYA-CCTA-TAAGACCGAGGTCTTTTTCCATTATTCCATGCAAAAATATTCAAGGCATT-AGAGCCTGCTTTGAGCACCTTAATTTGTTCAAGGTAAAAGTAAGCCGAACTAAATAGACACTTAGCCTAGTGAAAGGCATCGGTGCTATTCATTAA--GTGTTCAGTCATATAGTTCAAGTAA-TCGGAAATGATGGCA-GCCATTTACTTGATGGCGCCAC------ACCC-------G-TACTGAACAACAATCAACTTCGAACGTTTT-AACCGCAACAATTTTAATATACGCTAGTGGAGCTGGAATTACCGCGGCTGCTGGCACCAGACTTGCCCTCCACTTGATCCTCATTAAAGGATTTATACT-TGATTCATTCCAATTACAGAAC-TAAATAATTAGTTCTATATTGTTATTTTTCGTCACTACCTCC-----TTCAATATTCTTGCT---ACAATGTGCACTTCTCGCTTAGTAAGACGCTGTGATCCATTAGAAATTCATTGC---GGTTATTTGGCTATCAATAGGCCGGCATAAAGTTCATTGGCAAATAACTAGATCCTTTATGGTCTGGTATATTGCTTGGTGAGTTTGTCGTGTCCTGCTCTATAGCTGTCTGATGGATTTTAAAGGTCATAACATTGCATCACTCAGGTTA-CCCGATCAACAGCGGTTAAATTTGGGATACCTTTTGGACCCGTCTTGAAACACGGACCAAGGAGTCTATCTAGTATGCGAGTAAATGAGGAAACTTATTTGCGTAAAAAACTTGACTGATGGGATTACGAGTGCTCCTCCATCCCGGGGTATTTTCATCCACATATGCAAATGTATATGGAAAATATACCATGAGCATACTGGATATGACCCGAAAGATGGTGAACTATGCTTGATCAGGTTGAAGTCAGGGGAAACCCTGATGGAGGACCGAAGCAATTCTGACGTGCAAATCGCTGACTTATCCGCTTATTGGCAATTATGGCATTCCAAGTGACGAGGAGTTTGACGAGTTTAAGTTGGTGAAGAATTTAGAGTCGACCAACAAGATTTGGGTGTCGGCGCTGGTTGTCGGTGAGTTGTGTGAGACTCCATCGCACTGGCGACAGAAATTCAAATTGGCGGAATGGATGAAGAAACATAATGTGGTTGGAATAAGTGGAATCGATACCCGTGCGTTGACGAAAAAAATTCGAGAAGATGGAACAGTTCTTGGCAAGATCATTCAGCAGTCTTCG------GGACCTTTTCCAGGT---CTTGAGTTTGAGGATCAGAATTTGAGGAATCTTGTAGCTGAAGTTTCCATCAAAAAACCGATGACTTACAACCCCAATGGCTCACCGCGGATCTGCGCTGTTGATTGTGGATTGAAACTTAATCAAATTCGTTGCTTTCTCAAGCGTGGAGCACGTGTAGATGTTGTTCCATGGAATCATCCACTGAATCCTAATGATTTTGATGGTCTTTTCTTATCAAATGGTCCTGGTGATCCCGTCGTGTGTCAGGAAACTGTCAAAAATATTCAGAAAGTCATTGCAGCAAAACAAGTCAAACCAATTTTTGGAATCTGTCTCGGCCATCAGCTCTTGTCAACTGCTGTCGGATGCAAAACTTACAAGCTCAAATATGGTAATCGTGGTCATAACTTACCTGCAATCCATCATGGCACAAATCGATGTTTTATGACGTCACAAAATCATGGATTTGCCGTCGACGCCAAGACACTTGCTAAAGACTGGGAAGCTCTGTTCACGAATCTC------TTTATTCGAGTCAGCAAACACATCGGCAGCTCAATGAAATCCGTTGGTGAGGTCATGGCGATTGGTCGAAAGTTCGAAGAAGCTTTTCAGAAAGCTTTCCGCATGGTCGATGAGAATGTCAACGGATTTGATCCGAATCTTAAGCCTGTGTGCGATGAGGAGCTCAAGCAACCGACAGATAAACGAATGTTTGTGTTGGCTGCAGCTTTGAAGGCTGGTTACACAATTGATCGACTTTACGATCTCACGAAAATCGACCGCTGGTTCCTTGAAAAGATGAAGAACATCATTACAACAACTCAAGAGCTCGAAGCACTGAAC---TGCGTTATCCACAACGATCAATTGCGACGTGCAAAGTCCTTGGGATTTTCTGACAAGCAAATTGCCACWTGCATTAAA--TTCAGAGCTGGCGGTTCGCAAACARAGACGTGAATCTGGAATTTTTCCAATCGTTAAGCAGATTGATACCGTTGCTGGAGAATGGCCAGCTTCGACCAATTACTTGTATCTCACCTACAATGCAACAGAACACGACGTGGACTTCGAT---GAGCAAATGATCATGGTCATTGGATCTGGTGTTTACAGGATTGGAAGYTCAGTTGAGTTCGATTGGTGCGCTGTTGGATGTCTCAGGGAGCTCAGGAATCTCAGCAAAAAGACAATCATGGTCAATTACAATCCAGAAACCGTTTCAACGGACTATGACATGTGTGATCGCCTTTATTTCGAGGAAATATCATTCATATTATTAGTCAAGAAAGAGGGAAAAAAGAAACCTTCGGAGCATTAGGAATAATTTATGCAATATTAGCAATTGGTTTATTAGGATTTGTTGTCTGAGCTCATCATATATTTACAGTAGGAATAGATGTAGATACTCGAGCCTATTTTACTTCAGCTACTATAATTATTGCTGTGCCAACAGGAATTAAAATTTTTAGTTGATTAGCTACTTTACATGGAACACAACTTAACAACTCGCCTTCTTTATTATGAGCTTTAGGATTTGTGTTTTTATTTACTGTAGGAGGATTAACAGGAGTAGTTTTAGCTAATTCTTCTATTGATATTGTACTCCATGATACTTATTATGTAGTAGCTCACTTTCACTATGTTTTATCAATGGGAGCTGTTTTTGCTATTATAGCAGGATTTGTTCATTGATACTCATTATTTACAGGGCTTTCTATGAATGAACAATGATTAAAATCTCAATTTTTAATTATATTTGTAGGAGTAAATTTAACATTTTTTCCTCAACATTTTTTAGGCTTAGCTGGAATACCTCGACGATACTCTGACTATCCTGATGCTTATACTACTTGAAACATTATTTCAACTATCGGATCTACAATTTCTTTATTTGGGATTTTATTTTTTTTATTTATTATTTGAGAAAGA

**Naonella sp.** NNNNNNNNNNNNNNNNNNNNNNNNNNNNNNNNNNNNNNNNNNNNNNNNNNNNNNNNNNNNNNNNNNNNNNNNNNNNNNNNNNNNNNNNNNNNNNNNNNNNNNNNNNNNNNNNNNNNNNNNNNNNNNNNNNNNNNNNNNNNNNNNNNNNNNNNNNNNNNNNNNNNNNNNNNNNNNNNNNNNNNNNNNNNNNNNNNNNNNNNNNNNNNNNNNNNNNNNNNNNNNNNNNNNNNNNNNNNNNNNNNNNNNNNNNNNNNNNNNNNNNNNNNNNNNNNNNNNNNNNNNNNNNNNNNNNNNNNNNNNNNNNNNNNNNNNNNNNNNNNNNNNNNNNNNNNNNNNNNNNNNNNNNNNNNNNNNNNNNNNNNNNNNNNNNNNNNNNNNNNNNNNNNNNNNNNNNNNNNNNNNNNNNNNNNNNNNNNNNNNNNNNNNNNNNNNNNNNNNNNNNNNNNNNNNNNNNNNNNNNNNNNNNNNNNNNNNNNNNNNNNNNNNNNNNNNNNNNNNNNNNNNNNNNNNNNNNNNNNNNNNNNNNNNNNNNNNNNNNNNNNNNNNNNNNNNNNNNNNNNNNNNNNNNNNNNNNNNNNNNNNNNNNNNNNNNNNNNNNNNNNNNNNNNNNNNNNNNNNNNNNNNNNNNNNNNNNNNNNNNNNNNNNNNNNNNNNNNNNNNNNNNNNNNNNNNNNNNNNNNNNNNNNNNNNNNNNNNNNNNNNNNNNNNNNNNNNNNNNNNNNNNNNNNNNNNNNNNNNNNNNNNNNNNNNNNNNNNNNNNNNNNNNNNNNNNNNNNNNNNNNNNNNNNNNNNNNNNNNNNNNNNNNNNNNNNNNNNNNNNNNNNNNNNNNNNNNNNNNNNNNNNNNNNNNNNNNNNNNNNNNNNNNNNNNNNNNNNNNNNNNNNNNNNNNNNNNNNNNNNNNNNNNNNNNNNNNNNNNNNNNNNNNNNNNNNNNNNNNNNNNNNNNNNNNNNNNNNNNNNNNTTCAATATTCCCTTAAAAGCAATGTGCACTTCTCTCTTAGTAAGACGCTGTGATCCATTAGAAATTCATTCCAATGATTATTTGGCTATCAATGGGCCGATGTGAAGTTCATCAGCAAATGACTCGATCTTTTACGGTTGGGTGTATTGTCTGGTGAGCGCATTGTCACCCGT-CTATAGCTGTCTGATGGATTTTAAAGGTCATATTATTGCATCACTCAAAATAACCCGATCAACAGCGGTTAAATTTGGGATACCTTTTGGACCCGTCTTGAAACACGGACCAAGGAGTCTATCTAGTATGCAAGTAAATGAGGAAACTTATTTGCAGAAAAAACTTGACTGATGGGACTACGAGTGCTCCTCCATCCCGGGGTATTTTTATCAACATATGCAAATGTATATGGAAAATATACCATGAGCATACTGGATATGACCCGAAWGATGGTGAACTATGCCTGATCAGGTTGAAGTCAGGGGAAACCCTGATGGAGGACCGAAGCAATTCTGACGTGCAAATCG---------------------------GGAATACCGAGCGAYGAAGAATTCGACGAGCACAAGTTGATGAAGCATTTTGAGTCGAATAACAAGATTTGGGTTTCTGGTTTGGTGGTCGGTGAATTATGTGAAACACCGTCGCACTGGCGTCAAAAATACAAACTTGCCGAATGGATGAAGAAGCACAACGTGGTTGGCATCAGTGGAATTGACACACGCGCTTTGACCAAGAAAATTCGCGAGGACGGAACYGTTTTGGGTAAAATCATTCATCAATCTGCG------GGGCCATTTCCAGGR---TTGGAATTCAAGGATCAAAACAAGCGGAACCTCGTCGATGAAGTTTCAATTAAGAAGCCAATCACTTACAATGCAACCGGCTCGCCAAGAATCTGTGCCATTGATTGCGGATTGAAGTTGAATCAAATYCGWTGCTTYGTYAATCGAGGAGCTCGAGTYGATTTAGTGCCATGGAACCATGAACTGAAYCCTGACMATTTCGATGGTCTCTTCCTCAGCAATGGTCCCGGTGATCCTGTCATGTGCCAAAAGACCGTCGAAAACATTCAAAAATTCCTCGCATCACCCAAGGTGAAGCCGACKTTTGGTATCTGCCTTGGTCATCARTTGCTTGCGACTGCAATCGGTGGTAAAACTTACAAATTGAAATACGGAAACCGCGGCCATAATTTGCCGGCTCTTCACCATGGAACCAATCGYTGCTTCATGACCTCTCAAAATCACGGATTCGCTGTTGATGTTAAGACCATTGCCAAGGATTGGGAGCCGCTTTTCACGAACTTAAACGACTTCATCCGTGTCAGCAAGCACATTGGAAGCTCCATGAAGAGTGTCGGTGAAGTGATGGCGATTGGACGGAAATTCGAAGAAGCTTTTCAGAAAGCATTGCGAATGGTTGATGAGAATGTTGAYGGKTTYAATCCRAACTTGAAGCCAGTCTGYGATGAAGAATTGATGACTCCGACTGACAAGCGAATGTTTGTACTCGCAGCTGCTTTGAAAGCCGGTTACACYGTTGATCGTCTTTACGATTTAACAAAAATYGAYCGTTGGTTCCTCGAAAAGATGAAGAACATCAATGCAGTGACRATGGAGCTTGAGAAGCTYAAT---TGCTTAATTTCTGATGAACTTTTAGCAAAAGCCAAGCGACTCGGATTTTCTGATAAGCAAATTGCGAATTCAATCAAA--ATCTGAACTCGAAGTCCGAAAGCAACGTCGAGAAAGYGGAATTTTRCCTTTTGTYAAGCAAATCGACACGGTTGCTGGTGARTGGCCAGCGTCAACGAATTATCTTTACTTGACTTACAACGCAAGCACTCACGACATTGAATTCAAT---GATCAAATGGTGATGGTGATTGGTTCTGGTGTTTATCGCATTGGAAGCTCCGTTGAGTTTGATTGGTGTGCCGTTGGATGTTTGAGRGAGTTGAGAAACCTTGGCAAGAAGACAATCATGGTGAATTACAATCCTGAAACCGTKTCMACCGACTACGAYATGTGYGATCGCTTRTACTTTGAAGAAATTTCATTCATATTATTAGCCAAGAAAGTGGAAAAAAGGAAACTTTTGGGGCTTTAGGAATAATTTATGCTATACTAGCTATTGGGCTATTAGGATTTATTGTGTGAGCTCATCATATATTCACAGTAGGAATAGATGTTGATACCCGAGCTTACTTCACCTCAGCTACAATAATTATTGCTGTACCAACAGGTATTAAAATTTTTAGTTGATTAGCTACTCTTCACGGAACCCCTATAAATAACTCACCTTCTTTATTATGAGCATTAGGTTTCGTATTTTTATTTACAGTAGGAGGACTAACCGGGGTAATTCTTGCTAATTCATCTTTAGATATTGTCTTACATGATACTTATTATGTAGTTGCTCATTTTCATTATGTTCTTTCTATGGGAGCTGTTTTTGCTATTATGGCAGGATTTGTTCACTGATACACTTTATTCACAGGATTAATTATAAATGAAAAATGATTAAAATCTCAATTTGCTATTATATTCTTAGGTGTAAATTTAACATTTTTCCCTCAACATTTTTTAGGGTTAGCTGGAATGCCTCGACGTTACTCTGATTACCCCGATGCTTACACTTCATGAAATATTCTTTCTACTGTAGGATCAACTATTTCTCTTTTTGGTATTTTATTTTTTATTTTTATTATTTGAGAAAGT

**Notocladius sp.** -----------------------------------------------------------------------ACCCTATTAAGTTCTG-ACCTGGTAAGTTTTCCCGTGTTGAGTCAAATTAAGCCGCAACATCCACTGC-CGGTGATGATCTTCCGTCAATTCCTTTAAGTTTCAACTTTGCAACCATACTTCCCCCGGAAACT--AG-CTTT-GGTTTCCCGAAGAGCTACTGAATGCACCATGAAA--AGTAGTGACATCCAATCGCTAGCTGTCATCGTTTACAGTTAGAACTAGGGCGGTATCTAATCGCCTTCGATCCTCTAACTTTCGTTCTTGATTAATGAAAACATCCTTGGCAAATGCTTTCGCTTTAGTTAGTCTTGCAACGGTCTAAGAATTTCACCTCTCGCGCTGCAATACTAATGCCCCCAACTGCTTCTATTAGTCATTACCTCT-TGATCTG-TATTAAACCAACAGAAAA-TCACT------------TGCAGCTTTCACCACAAA-CGAA-TAAGACCGAGGTCTTTTTCCATTATTCCATGCAAAAATATTCAAGGCGTA-AGAGCCTGCTTTGAGCACCTTAATTTGTTCAAGGTAAAATTAAGCCGAACTAAATAGACACTTAGCCTAATGAAAGGCATCAGTGCTATTCATTTA--GTGTTCAGTCAAATAGTTCAAGTAA-TCGGAAATGATGGCA-GTCATGTACTTAATGACGCCAC------ACCC-------G-TACTGAACAATAATCAACTTCGAACGTTTTTAAACGCAACAATTTTAATATACGCTAGTGGAGCTGGAATTACCGCGGCTGCTGGCACCAGACTTGCCCTCCA--------------------------------------------------------------------------------------------------TTCAATATTTTGTAAAAGACAATGTGCACTTCTTTCTTAGTAAGATGCTGTGATTCATTAGAAATTCATTTCATTGATTATGAGGCTATCAATAGGCCAACATGAAGTTCATCAATGAATGATATGATCTTTAATGGTCGTGTGTATTGTATGGTGAGCATGTTGTGTTCTG--ATATAGCTGTCTGATGGATTTTAAAGTTCATATTATTACATCACTCAGAATAAACCGATCAACAGCA-CTAAATTTGGGATACCTTTTGGACCCGTCTTGAAACACGGACCAAGGAGTTTATCTAATATGCGAGTAAATGAGTAAACTTATTTGCGTAAATAAATTAACTGATGGGATTACGAGCACTCTTCCATCCCGGGGTATTTTTATCAACATATGCAAATGTATATGGAAAATATACCATGAGCATACTGGATATGACCCGAAAGATGGTGAACTATGCTTGATCAGGTTGAAGTCAGGGGAAACCCTGATGGAAGACCGAAGCAGTTCTGACGTGCAAATCGNNNNNNNNNNNNNNNNNNNNNNNNNNNNNNNNNNNNNNNNNNNNNNNNNNNNNNNNNNNNNNNNNNNNNNNNNNNNNNNNNNNNNNNNNNNNNNNNNNNNNNNNNNNNNNNNNNNNNNNNNNNNNNNNNNNNNNNNNNNNNNNNNNNNNNNNNNNNNNNNNNNNNNNNNNNNNNNNNNNNNNNNNNNNNNNNNNNNNNNNNNNNNNNNNNNNNNNNNNNNNNNNNNNNNNNNNNNNNNNNNNNNNNNNNNNNNNNNNNNNNNNNNNNNNNNNNNNNNNNNNNNNNNNNNNNNNNNNNNNNNNNNNNNNNNNNNNNNNNNNNNNNNNNNNNNNNNNNNNNNNNNNNNNNNNNNNNNNNNNNNNNNNNNNNNNNNNNNNNNNNNNNNNNNNNNNNNNNNNNNNNNNNNNNNNNNNNNNNNNNNNNNNNNNNNNNNNNNNNNNNNNNNNNNNNNNNNNNNNNNNNNNNNNNNNNNNNNNNNNNNNNNNNNNNNNNNNNNNNNNNNNNNNNNNNNNNNNNNNNNNNNNNNNNNNNNNNNNNNNNNNNNNNNNNNNNNNNNNNNNNNNNNNNNNNNNNNNNNNNNNNNNNNNNNNNNNNNNNNNNNNNNNNNNNNNNNNNNNNNNNNNNNNNNNNNNNNNNNNNNNNNNNNNNNNNNNNNNNNNNNNNNNNNNNNNNNNNNNNNNNNNNNNNNNNNNNNNNNNNNNNNNNNNNNNNNNNNNNNNNNNNNNNNNNNNNNNNNNNNNNNNNNNNNNNNNNNNNNNNNNNNNNNNNNNNNNNNNNNNNNNNNNNNNNNNNNNNNNNNNNNNNNNNNNNNNNNNNNNNNNNNNNNNNNNNNNNNNNNNNNNNNNNNNNNNNNNNNNNNNNNNNNNNNNNNNNNNNNNNNNNNNNNNNNNNNNNNNNNNNNNNNNNNNNNNNNNNNNNNNNNNNNNNNNNNNNNNNNNNNNNNNNNNNNNNNNNNNNNNNNNNNNNNNNNNNNNNNNNNNNNNNNNNNNNNNNNNNNNNNNNNNNNNNNNNNNNNNNNNNNNNNNNNNNNNNNNNNNNNNNNNNNNNNNNNNNNNNNNNNNNNNNNNNNNNNNNNNNNNNNNNNNNNNNNNNNNNNNNNNNNNNNNNNNNNNNNNNNNNNNNNNNNNNNNNNNNNNNNNNNNNNNNNNNNNNNNNNNNNNNNNNNNNNNNNNNNNNNNNNNNNNNNNNNNNNNNNNNNNNNNNNNNNNNNNNNNNNNNNNNNNNNNNNNNNNNNNNNNNNNNNNNNNNNNNNNNNNNNNNNNNNNNNNNNNNNNNNNNNNNNNNNNNNNNNNNNNNNNNNNNNNNNNNNNNNNNNNNNNNNNNNNNNNNNNNNNNNNNNNNNNNNNNNNNNNNNNNNNNNNNNNNNNNNNNNNNNNNNNNNNNNNNNNNNNNNNNNNNNNNNNNNNNNNNNNNNNNNNNNNNNNNNNNNNNNNNNNNNNNNNNNNNNNNNNNNNNNNNNNNNNNNNNNNNNNNNNNNNNNNNNNNNNNNNNNNNNNNNNNNNNNNNNNNNNNNNNNNNNNNNNNNNNNNNNNNNNNNNNNNNNNNNNNNNNNNNNNNNNNNNNNNCATATTATTAGTCAGGAAAGAGGAAAGAAAGAAACTTTTGGAGCTTTAGGAATAATCTATGCTATATTGGCAATTGGTTTATTAGGATTTGTAGTATGAGCACATCATATATTTACTGTAGGTATAGACGTGGACACTCGAGCTTATTTTACATCTGCTACAATAATTATTGCAGTACCAACCGGAATTAAAATTTTCAGTTGATTAGCAACTTTACACGGGACACAAATAAATAACTCCCCTTCAATATTATGAGCCCTAGGGTTTGTATTTTTATTTACAGTAGGAGGACTAACTGGGGTAGTACTAGCCAATTCTTCTATTGATATTGTCTTACATGATACTTATTATGTAGTTGCTCACTTTCATTATGTTCTGTCCATGGGGGCAGTATTTGCCATTATAGCTGGATTTGTACATTGATACCCTCTATTTACTGGACTAACTTTAAATGAAACTATATTAAAATCACAATTTTTTATAATATTCTTAGGAGTAAATTTAACATTTTTCCCCCAACATTTTTTAGGTCTTGCCGGGATACCCCGACGATACTCAGATTACCCAGACGTGTACACTTCATGAAATATTGTTTCTTCTATTGGATCTACAATCTCTTTTGTGGCTATTATATACTTTATTTTTATTATTTGAGACAGA

**Odontomesa sp.** TCCACGAACTAAGAACGGCCATGCACCACT--ACCCTTAATTTC-A-AGAAAACGCTGTCAA-GCTGTCTAACCCTATTAAGTTCGG-ACCTGGTAAGTTTTCCCGTGTTGAGTCAAATTAAGCCGCAAATTCCAAATC-TTGTTGTGCCCTTCCGTCAATTCCTTTAAGTTTCAACTTTGCAACCATACTTCCCCCGGAAACT--TG-CTTT-GGTTTCCCGGA-AGCTACTGAATGCACCATGAAT--AGTAGTGACATCCAATTGCTAGCTGTCATAGTTTACAGTTAGAACTAGGGCGGTATCTAATCGCCTTCGATCCTCTAACTTTCGTTCTTGATTAATGAAAACATCCTTGGCAAATGCTTTCGCTTTAGTTAGTCTTACGACGGTCTAAGAATTTCACCTCTCGCGCCGTAATACTAATGCCCCCAACTGCTTCTATTAATCATTACCTCT-TGATCTG-TATCAAACCAATAGAAAG-CGACA-------------TACACACAAGGTGAGTG-TCTA-TAAGACCGAGGTCTTGTTCCATTATTCCATGCAAAAATATTCG-GGCATT-TGAGCCTGCTTTGAGCACCTTAATTTGTTCAAGGTAATAGTAAGCCGAACTAAATAGGCACCTAGCCTAGTGAAAGGCATCAGTGCTATTCATTAA-AGTGTTCGGTCATATAGTCCAAGTCA-GCGGAAATGATAGCA-ACCATTTACTTGATGGTGCTAC------ACCC-------G-TACTGGACAATAATCAACTTCGAACGTTTT-AACCGCAACAATTTTAATATACGCTAGTGGAGCTGGAATTACCGCGGCTGCTGGCACCAGACTTGCCCTCCACTTGATCCTCATTAAAGGATTTATACT-TGATTCATTCCAATTACAGAACATAGTTAACTAGTTCTATATTGTTATTTTTCGTCACTACCTCC-----TTCAATACTCCGTTAAAAGTAGTGTGCACTTTTCCTTTAGTAAGACGCTGTGATCCATTAGAAATTCATATCAATGGTTATTAGGCTATCAATAGGCTGCTATGAAGTTCACTGGCAAATGATCTAATCTTTTATGGTTGGGTGTATTGCTTGGTGAGCATAGCGGTACCTGTA--ATAGCTGTCTGATGGATTTTAAAGGTCATATTACTGCATTACCC-TGATAACCCGATCAACAGCGGTTAAATTTGGGATACCTTTGGGACCCGTCTTGAAACACGGACCAAGGAGTCTATCTAGTATGCAAGTAAATGGGGAAACCTATTTGCATAAAAAACTTGACTGATGGGATTACGAGCGTTTCTCCATCCCGGGGCATTTTCATCAACATATGCGAACGTATATGGGAAATGTACCATGAGCATACTGGATATGACCCGAAAGATGGTGAACTATGCCTGATCAGGTTGAAGTCAGGGGAAACCCTGATGGAGGACCGAAGCAATTCTGACGTGCAAATCGTTAACTTATCCATTGATTGGAAATTATGGAATACCTGATGAAGAAGAATTTGATGACAACCAACTGATCAAGCATTTTGAATCGAATAATAAAATCTGGATTTCTGGCCTTATTGTTGGCGAAATATGTGACACACCTTCTCATTGGAGACAAAAGTATAAATTATCAGAATGGATGAATAAACACAATATTCCCGGAATAAGCGGAATTGATACTAGAGCTTTAACGAAGAAAATCCGTGAAAACGGAACAATTTTGGGAAAAATTATTCAAAAACCATCC------GGTCCATTTCCTGAT---CTTGAATTTAAAGATCAAAACGAACGTAATCTTGTTGATGAAGTTTCTGTAAAAAAGACTGTAACATACAATCAATCAGGATCACCTCGCATTTGTGCCATCGATTGTGGACTTAAATTGAATCAAATCCGATGCTTTGTTAAACGTGGAGCTCGTGTAGATGTGGTTCCATGGGATCATGAATTAAAGCCTGATACTTTCGATGGTCTTTTTCTCAGCAATGGTCCCGGCGATCCAGTAATGTGTCAAAAAACTGTTAAGAACATTCAAAAACTATTGGCGCTGCCAAAAGTAAAGCCGATTTTTGGTATTTGCTTGGGTCATCAGCTTTTATCTACCGCAATTGGATGTAAAACATACAAAATGAAATATGGTAATAGAGGCCACAACTTACCAGCTCTCCACCACGGTACTAATCGTTGCTTCATGACTTCACAAAACCATGGCTTTGCTGTAGATATCTCAAAAATTGATTCAAATTGGGAACCTCTCTTCACAAATTTAAATGAT------------------------------------------------GAAGTTATGGCAATTGGTAGAAAATTTGAAGAAGCATTTCAAAAAGCTTTAAGAATGGTTGATGAAAATGTAAATGGTTTTGATCCTAATTTAAAACAAGTTAAAGATGAAGAATTAACAACGCCTACTGACAAAAGAATGTTTGTGCTTGCAGCGGCTTTGAAAGCTGGTTATACAATTGATCGGCTTTATGATTTAACAAAAATTGATCGTTGGTTTCTTCAAAAAATGAAAAATATTATTGATATWACATTGGATCTGGAAAAACTTAAT---TGTATGGTACCTGATTCTCTTTTATATGAAGCCAAAAAATATGGATTTTCTGATAAACAAATTGCAACATTCATCAAG--TTCAGAGCTAGCTGTTAGAAAACAACGACGTGAATGTGGCATTTTGCCTTTTGTGAAACAAATTGATACTGTTGCTGGAGAATGGCCTGCTTCAACAAACTATCTCTATTTAACATACAATGCATGTGAACATGATATTGATTTCGAA---GATCAAATGGTGATGGTTATCGGTTCTGGTGTTTATCGCATTGGAAGTTCCGTAGAATTTGATTGGTGCGCTGTTGGATGTTTAAGAGAATTAAGAAAGTTGGGTAAAAAAACTATTATGGTGAATTACAATCCTGAAACA-----------------------------------------------------CATATTATTAGTCAAGAAAGTGGGAAAAAGGAAACTTTTGGAGCTTTAGGAATAATTTATGCTATACTAGCAATTGGTTTATTAGGTTTTGTTGTATGAGCTCATCATATATTTACTGTAGGTATAGACGTAGATACTCGAGCTTATTTTACATCAGCTACAATAATTATTGCTGTTCCTACAGGAATTAAAATTTTTAGTTGATTAGCTACACTTCACGGAACACAATTAAATTATTCCCCTTCTTTATTATGAGCATTAGGATTTGTATTTTTATTTACTGTTGGTGGATTAACAGGTGTTATTTTAGCTAATTCTTCTTTAGATATTGTATTACATGATACTTATTATGTAGTAGCTCATTTTCATTATGTTTTATCTATGGGAGCTGTATTTGCTATTATAGCCGGATTTGTTCATTGATACTCTTTATTTACAGGATTAACTATAAATGAAGAGTGACTAAAATCTCAATTTATAATTATATTTTTAGGAGTAAATTTAACATTTTTTCCACAACATTTTCTCGGATTAGCTGGTATGCCTCGTCGATATTCTGACTATCCAGATGCCTATACTTCATGAAATGTTGTATCAACTTTAGGATCAACTATTTCTTTATTTGGAATTTTATTTTTTCTTTTTATTATTTGAGAAAGT

**Orthocladius (Euorthocladius) luteipes** --------CTAAGAACGGCCATGCACCACT--ACCCTTAATTTA-G-CGAAAGCGCTATCAA-ACTGTCGTACCCTATTAAGTTCGG-ACCTGGTAAGTTTTCCCGTGTTGAGTCAAATTAAGCCGCAAATTCCAAATC--TGGTGTCGAATTCCGTCAATTCCTTTAAGTTTCAACTTTGCAACCATACTTCCCCCGGAAACT--AG-CTTT-GGTTTCCCTGAAAGCTACTGAATGCACCATATAA--AGTAGTGACATCCAATTGCTAGCTGTCATCGTTTACAGTTAGAACTAGGGCGGTATCTAATCGCCTTCGATCCTCTAACTTTCGTTCTTGATTAATGAAAACATCCTTGGCAAATGCTTTCGCTTTAGTTAGTCTTGCGACGGTCTAAGAATTTCACCTCTCGCGCCGCAATACTAATGCCCCCGACTGCTTCTATTAATCATTACCTCT-TGATCTG-TATCAAACCAATAGAAAG-CGATA------------TAACTCTTGCGAGCTACA-CCTA-TAAGACCGAGGTCTTTTTCCATTATTCCATGCAAAAATATTCAAGGCATT-AGAGCCTGCTTTGAGCACCTTAATTTGTTCAAGGTAAAAGTAAGCCGAACTAAATAGACACTTAGCCTAGTGAAAGGCATCGGTGCTATTCATTAA--GTGTTCAGTCATGTAGTTCAAGCAA-TCGGAAATGATGACA-GCCA-TTACTTGATGGCGTCAC------ACCC-------G-TACTGAACAAAAATGAACTTCGAACGTTTT-AACCGCAACAATTTTAACATACGCTAGTGGAGCTGGAATTACCGCGGCTGCTGGCACCAGACTTGCCCTCCACTTGATCCTCATTGAAGGATTTATACT-CGATTCATTCCAATTACAGAAC-TAAATAATTAGTTCTATATTGTTATTTTTCGTCACTACCTCC-----TTCAATACTTTGGCAACAATAGTGTGCACTTCTCACTTAGTAAGACGTTGTGATCCATTAGAAATCCATTCCAATGGTTAATTGGCTATCAATAGGCTGGCGTAAAGTTCTCTTGCAAATGGCTTGATCTTTAATGGTCGGGTGTATTGTTTGGGGAGTGCGTCATGTTCTGT-CTATAGCTGTCTGATGGATTTTAAAGGTCATAACATTGCATCACCCAGGTTAACCCGATCAACAGCGGTTAAATTTGGGATACCTTTTGGACCCGTCTTGAAACACGGACCAAGGAGTCTATCTATTGCGCGAGTAAATGGGGAAACCTATTTGCGGAAAAAACTTGACTGATGGGATTACGAGTGCTCCTCCATCCCGGGGTATTTTCATCAACACATGCAAATGTATGTGGAAAATATACCATGAGCGTAGTGGATATGACCCGAAAGATGGTGAACTATGCCTGATCAGGTTGAAGTCAGGGGAAACCCTGATGGAGGACCGAAGCAATTCTGACGTGCAAATCGCTGACGTATCCGTTAATTGGCAACTATGGAGTTCCTAGCGACGAAGAGTTTGATGAACACAAGTTGATGAAGCATTTTGAATCGAATAACAAGATTTGGGTGTCAGGGCTTGTTGTTGGCGAACTTTGTGAAACTCCGTCGCACTGGCGTCAAAAGTATAAACTCGCTGAATGGATGAAAAAACACAACGTGGTCGGAATCAGTGGAATTGACACGCGTGCGTTGACGAAAAAGATTCGCGAGAATGGAACGGTTCTGGGAAAAATCATTCAGCAGTCCGCT------GGACCATTTCCAGGT---CTCGAGTTCAAGGACCAAAATGAACGGAATCTTGTCGACGAAGTTTCCATTAAAAAACCGGTTACTTACAACGCATCTGGATCACCAAGAATTTGCGCCGTTGACTGTGGACTAAAATCTAATCAAATTCGCTGCTTCATCAATCGTGGAGCGAGAGTTGACTTAGTACCATGGGATCATCTACTGAATCCTAACGACTTTGATGGACTTTTTCTTTCCAATGGTCCAGGCGATCCCGTTATGTGCGAAAAAACAGTTAAGAACATTCAAAAAGTTCTATCGTCTTCACAAGTCAAACCTGTTTTTGGTATCTGCCTTGGCCATCAACTGCTTGCCACTGCTGTCGGATGTAAGACTTACAAAATGAAGTATGGCAACCGTGGTCACAACTTGCCAGCCCTTCACCATGGAACTAACCGATGCTTCATGACATCGCAAAACCACGGATTTGCTGTCGACAGCGCCACAATTGCCAAGGTT------------------------------TTTATTCGTGTCAGCAAACAYATCGGAAGCTCGATGAAGAGTGTCGGTGAAGTTATGGCAATCGGAAGGAAATTTGAAGAAGCTTTTCAAAAAGCTCTGCGCATGGTCGACGAAAACGTGAACGGTTTCGATCCTAACATCAAGCCGGTAAAAGACGAAGAACTGACACAGCCAACTGACAAACGAATGTTTGTGCTTGCGGCGGCTTTGAAAGCAGGCTACACCGTCGACCGACTTTATGACTTGACCAAAATCGACCGTTGGTTCCTCGAAAAAATGAAAAACATCATTGAAATCACTTTGGAGCTTGAAAAACTCAAC---TGCAACATTTCCGATGATCTGCTGAAACAATCGAAAAAGTTGGGATTCTCCGACAAGCAAATCGCAAAGTACATYAAA--GTCGGAACTTGCTGTGCGAAAGCAGCGGCGTGAAAGTGGAATTTTGCCATTTGTGAAGCAAATTGACACTGTCGCTGGCGAATGGCCGGCATCCACCAACTATCTTTACCTCACCTACAATGCAAGTTCTCACGACATCGAATTTAAT---GATCAGATGGTGATGGTTATTGGYTCRGGMGTTTATCGCATTGGAAGYTCAGTCGAGTTTGACTGGTGTGCTGTTGGATGTTTGCGCGAATTGAGAAATCTCGGCAAAAAGACAATCATGGTGAATTACAATCCCGAGACTGTTTCGACCGATTATGATATGTGTGACCGCTTGTACTTTG-------------CATATTATTAGTCAAGAAAGAGGTAAAAAGGAAACTTTTGGAGCCCTTGGAATAATTTATGCTATATTAGCTATTGGATTATTAGGTTTTGTTGTATGAGCTCATCATATATTCACAGTAGGTATAGATGTAGATACTCGAGCTTATTTTACTTCAGCTACAATAATTATTGCTGTTCCTACGGGAATTAAAATTTTTAGTTGACTAGCTACTTTACATGGATCTCAATTAAATAACTCCCCTTCATTATTATGAGCTTTAGGTTTTGTATTTTTATTTACTGTAGGAGGATTAACAGGAGTAGTATTAGCTAATTCTTCTATTGATATTATTTTACATGATACTTATTATGTAGTTGCTCATTTCCACTATGTTCTTTCAATAGGAGCAGTATTCGCTATTATAGCAGGGTTCGTTCATTGATATCCTTTATTTACGGGATTATCAATAAATGAAAAATGATTAAAGTCTCAATTTAGAATTATATTTTTAGGTGTAAACTTAACATTCTTCCCTCAACATTTCTTAGGGTTAGCTGGTATACCTCGACGATATTCTGATTATCCTGATGCTTACACTTCTTGAAATATTGTTTCTACTGTAGGATCAACAATTTCTTTATTTGGTATCTTATTCTTTCTTTTTATTATTTGAGAAAGT

**Parachaetocladius sp.** -------------------------------------------C-A-TGAAAACGCTATCAA-ATTGTCATACCCTATTAAGTTCGG-ACCTGGTAAGCTTTCCCGTGTTGAGTCAAATTAAGCCGCAAGGTCCACATC-ATGGTGTGATCTTCCGTCAATTCCTTTAAGTTTCAACTTTGCAACCATACTTCCCCCGGAAACT--AG-CTTT-GGTTTCCCGGAAAGCTACTGAATGCACCATGAAA--AGTAGTGACATCCAATTGCTAGCTGTCATCGTTTACAGTTAGAACTAGGGCGGTATCTAATCGCCTTCGATCCTCTAACTTTCGTTCTTGATTAATGAAAACATCCTTGGCAAATGCTTTCGCTTTAGTTAGTCTTGCGACGGTCTAAGAATTTCACCTCTCGCGCCGCAATACTAATGCCCCCAACTGCTTCTATTAATCATTACCTCT-TGATCTG-TATCAAACCAATAGAAAG-CGATA----------CGATGCCTTGCGACACCGCA-TCTA-TAAGACCGAGGTCTTTTTCCATTATTCCATGCAAAAATATTCAAGGCATA-AGAGCCTGCTTTGAGCACCTTAATTTGTTCAAGGTAAAAGTAAGCCGAACTAAATAGACACCTAGCCTAGTGAAAGGCATCAGTGCTATTCATTAA--GTGTTCAGTCATATAGTTCAAGTAA-TCGGAAATGATGGCA-GCCATTTACTTGATGGCGCCAC------ACCC-------G-TACTGAACAATAATCAACTTCGAACGTTTT-AACCGCAACAATTTTAATATACGCTAGTGGAGCTGGAATTACCGCGGCTGCTGGCACCAGACTTGCCCTCCACTTGATCCTCATTAAAGGATTTATACT-TGATTCATTCCAATTACAGAAC-TAATTAATTAGTTCTATATTGTTATTTT-------------------TTCAATATTCCCTTAAAAGCAATGTGCACTTCTCTCTTAGTAAGACGCTGTGATCCATTAGAAATTCATTCCAATGATCATTTGGCTGTCAATAGGCCGATGTGAAGTTCATCGGCAAATGACTCGATCTTTTACGGTTGGGTATATTGTTTGGTGAGCGCATCGTAACCTGTACAATAGCTGTCTGATGGATTTTAAAGGCCATAACATTGCATCACTCAGGTTT-CCCGATCAACAGCGGTTAAATTTGGGATACCTTTTGGACCCGTCTTGAAACACGGACCAAGGAGTCTATCTAGTATGCGAGTAAATGAGGAAACTTATTTGCATAAAAAACTTGACTGATGGGACTACGAGTGCTCCTCCATCCCGGGGTATTTTCATCAACATATGCAAATGTATATGGGAAATATACCATGAGCACACTGGATATGACCCGAAAGATGGTGAACTATGCCTGATCAGGTTGAAGTCAGGGGAAACCCTGATGGAGGACCGAAGCAATTCTGACGTGCAAATCGCTGACTTATCCGCTGATCGGCAACTACGGAATCCCGAGCGACGAAGAGTTCGACGAGCACGGACTGATGAAGAACTTCGAGTCCAACGGGAAAATTTGGGTCTCCGGACTGGTGGTCGGTGAATTGTGTGAGACTCCATCGCACTGGCGCCAGAAATACAAACTCGCCGAGTGGATGAAGAAGCACAACGTCGTCGGCATCAGTGGCATTGACACGCGCGCCTTGACCAAGAAGATCCGCGAGAACGGCACGGTTTTGGGCAAGATCATCCAGCAATCGAGC------GGGCCCTTCCCGGGC---TTGAAGTTCCAGGACCAGAACGAGAGGAATCTCGTCGACGAGGTGTCCATCAAGAAGCCGGTCACTTACAACGCTTCAGGCTCGCCGACAATTTGCGCGGTGGATTGCGGGCTGAAGTCGAATCAGCTCCGCTGCTTCATTAAGCGCGGAGCCCGCGTCGACTTGGTGCCATGGGACCATCCGCTCAACCCCGACAACTTCGACGGGCTTTTCCTCAGCAACGGCCCAGGCGACCCCGTCATGTGCCAGAAGACCGTCAAGAACCTTCAACAGTTTCTCGCGTCGCCGAAGGTGAAGCCGGTTTTTGGCATCTGCCTGGGCCACCAGCTCCTGGCGACCGCTGTCGGATGCAAAACGTACAAGATGAAGTACGGAAATCGCGGACACAACTTGCCGGCCTTGCACCACGGCACCAACCGGTGCTTCATGACGTCGCAGAATCACGGCTTCGCGGTCGATGCGGCCACGATTAACAAGGAGTGGGAGCCGCTCTTCACCAACCTGAACGACNNNNNNNNNNNNNNNNNNNNNNNNNNNNNNNNNNNNNNNNNNNNNNNNNNNNNNNNNNNNNNNNNNNNNNNNNNNNNNNNNNNNNNNNNNNNNNNNNNNNNNNNNNNNNNNNNNNNNNNNNNNNNNNNNNNNNNNNNNNNNNNNNNNNNNNNNNNNNNNNNNNNNNNNNNNNNNNNNNNNNNNNNNNNNNNNNNNNNNNNNNNNNNNNNNNNNNNNNNNNNNNNNNNNNNNNNNNNNNNNNNNNNNNNNNNNNNNNNNNNNNNNNNNNNNNNNNNNNNNNNNNNNNNNNNNNNNNNNNNNNNNNNNNNNNNNNNNNNNNNNNNNNNNNNNNNNNNNNNNNNNNNNNNNNNNNNNNNNNNNNNNNNNNNNNNNNNNNNNNNNNNNNNNNNNNNNNNNNNNNNNNNNNNNNNNNNNNNNNNNNNNNNNNNNNNNNNNNNNNNNNNNNNNNNNNNNNNNNNNNNNNNNNNNNNNNNNNNNNNNNNNNNNNNNNNNNNNNNNNNNNNNNNNNNNNNNNNNNNNNNNNNNNNNNNNNNNNNNNNNNNNNNNNNNNNNNNNNNNNNNNNNNNNNNNNNNNNNNNNNNNNNNNNNNNNNNNNNNNNNNNNNNNNNNNNNNNNNNNNNNNNNNNNNNNNNNNNNNNNNNNNNNNNNNNNNNNNNNNNNNNNNNNNNNNNNNNNNNNNNNNNNNNNNNNNNNNNNNNNNNNNNNNNNNNNNNNNNNNNNNNNNNNNNNNNNNNNNNNNNNNNNNNNNNCATATTATTAGACAGGAAAGAGGGAAAAAAGAAACTTTTGGTGCATTAGGAATAATTTATGCTATATTAGCAATTGGTTTACTAGGATTTGTTGTTTGAGCCCATCATATATTTACTGTAGGAATAGATGTGGATACTCGTGCTTACTTTACGTCTGCTACTATAATTATTGCAATTCCAACAGGAATTAAAATTTTTAGTTGATTAGCTACTTTACATGGAACTCAAATAAATAATTCTCCTTCTTTACTTTGAGCTTTAGGATTTGTTTTTTTATTCACAGTTGGAGGTTTAACAGGAGTAATTTTAGCTAACTCTTCTATTGATATTATTTTACATGATACTTATTATGTTGTTGCCCATTTTCATTATGTCCTATCAATAGGAGCAGTATTTGCAATTATAGCAGGATTTGTTCATTGATACCCTTTATTTACAGGTTTAACAATAAATGAAAAATGATTAAAATCTCAATTTTCTATTATATTTTTAGGTGTTAATTTGACATTTTTTCCACAACATTTTTTAGGATTAGCAGGCATACCTCGACGTTATTCAGATTACCCAGATGCCTATACTTCTTGAAATATTATTTCTACAGTAGGATCTACTATTTCATTATTTGGAATTTTATTTTTCTTATATATTATTTGAGAAAGA

**Paraheptagyia tonnoiri** ----CGAACTAAGAACGGCCATGCACCACT--ACCCTTAATTTC-G-AGAAAGAGCTATTAA-TCTGTCTTACCCTATTAAGTTCGG-ACCTGGTAAGTTTTCCCGTGTTGAGTCAAATTAAGCCGCAGGCTCCACTCC-TGGTGGTGCCCTTCCGTCAATTCCTTTAAGTTTCAACTTTGCAACCATACTTCCCCCGGAAACT--AG-CTTT-GGTTTCCCGGA-AGCTACTGAATGCGCCAT-AAT--AGTAGCGACATCCAATTGCTGGCTGTCATTGTTTACAGTTAGAACTAGGGCGGTATCTAATCGCCTTCGATCCTCTAACTTTCGTTCTTGATTAATGAAAACATCCTTGGCAAATGCTTTCGCTTTAGTTAGTCTTACGACGGTCTAAGAATTTCACCTCTCGCGCCGTAATACTAATGCCCCCAACTGCTTCTATTAATCATTACCTCT-TGATCTG-TATCAAACCAATAGAAA--CTAAA---------------------------------TA-TAAGACCGAGGTCTTTTTCCATTATTCCATGCAAAAATATTCAAGGCGTATATAGCCTGCTTTGAGCACCTTAATTTGTTCAAGGTAATAGTAAGCTGAACTAAATAGGCA-TTAACCTAGTGAAAGGTGTCA-TGCTATTCATTAATAGTGTTCAGTCATATAGTCCAAGTAA-TCGGAAATGATTACA-ACCA-TAACTTGATGGTGTAAC------ACCC-------G-TACTGGACAATAATCAACTTCGAACGTTTT-AACCGCAACAATTTTAATATACGCTAGTGGAGCTGGAATTACCGCGGCTGCTGGCACCAGACTTGCCCTCCACTTGATCCTTGTTAAAGGATTTATAAT-TAACTCATTCCAATTACAGAACATAGTTAACTAGTTCTATATTGTTATTTTTCGTCACTACCTCC-----TTCAATACTTCGTTAAACACGGTGTGCACTTTTCTCTTAGTAAGACGTTGTGATCTATTAGGAATTTGTATAGAATATCATTAGGTTACCAATAGTCTATTATGAAGTTCACCTACAAATAATATAGTCGTTTATGGTTGTGTATATTGTCTGGTGAGCATAATATGTACTG--GTGTAACTGCCTGATAGATTTTAAAGGTTATATCACTGCATTACCCCGGATAACCCGATCGACAATGGTTAAATTTGGGATACCTTTGGGACCCGTCTTGAAACACGGACCAAGGAGTCTATCTAGTATGCAAGTAAATGGGGAAACCTATTTGCATAAATAACTTGACTGATGGGATTACGAGTATTCCTCCATCCCGGGGTATTTTTATCAACATATA-AAAT-TATATGGAAAATATACCATGAGCATACTGGATATGACCCGAAAGATGGTGAACTATGCCTGATCAGGTTGAAGTCAGGGGAAACCCTGATGGAGGACCGAAGCAATTCTGACGTGCAAATCGTTGACTTATCCGCTGATTGGCAATTATGGCATTCCT---GATGAAGAAGTTGACGAACATCAACTGGCAGTAAACTTTGAATCCAACAATAAAATTTGGATATCGGCTTTAGTGGTTGGTGAACAGTGTGATACACCATCGCATTGGCGTTTGAAGTATAAATTATCGGAATGGATGAAGAAGCATGGAATTCCTGGAATTTCTGGTATTGACACTCGTGCACTGACAAAAAATATTCGTGAAAATGGTACGGTGTTGGGTAAGATCATTCAAAAACCTGCAAGTGACAATTTTACTATTGGA---TTGAAGTTTCAAGATCAAAATGAAAGAAATCTTGTTGATGAAGTTTCAATTAAGAAACCGATAACTTACAATGCTGGTGGATCGCCACGCATTTGTGCCGTTGATTGTGGATTAAAATTAAATCAAATTCGTTGTTTCATCAAACGTGGAGCTCGAGTCGATCTCGTACCGTGGGATTATAATTTGAATTCTAATGATTATGATGGACTTTTTCTATCGAATGGTCCCGGAGATCCCGTAATGTGTCGAAAAACCGTAGAGAAYATTAAAAGTGTAATTGCGTCTCCAAGTGCTAAACCTCTTTTTGGGATTTGCTTGGGACATCAGTTGTTATCGACAGCAATCGGTTGTAATACTTACAAACTGAAGTACGGTAATCGTGGTCATAATTTACCATGCCTTCATCATGGCACAAATCGTTGCTTCATGACTTCACAAAATCATGGATTTGCCGTTGACGTTTCGAAAATTGATAAAAATTGGGAACCATTATTTACAAATGCAAATGAT------------------------------------------------GAAGTGATGGCAATCGGTCGAACGTTTGAAGAAGCTTTTCAGAAAGCATTGCGAATGGTAGATGAAAACGTCAACGGTTTTGATCCAAACTTGAAAAAAGTAAATGATGAAGAACTTTCAACACCTACCGACAAGAGAATGTTTGTCTTGGCAGCAGCTTTAAAAGATGGTTATACTGTTGACAAACTGTACAATTTAACAAAAATTGATCGCTGGTTTTTAGAAAAAATGAAAAACATCATTGATGTTACATTAGAGTTAGAAACTTTAGAT---ACAAAACTATCGGTTGAACTTCTTACGATAGCTAAAAAAATGGGTTTCTCAGATAAACAGATTGCATCATACATTAAA--TTCTGAGTTGGCAGTAAGAAAGCAGCGTCGCGAAAATCGCATACTTCCGTTTGTAAAACAAATTGATACTGTTGCAGGTGAATGGCCAGCAACTACAAAYTATCTCTACTTTTCATACAATGCTTCAACTCATGACATTCAATTTGAA---GATCAACATGTTATGGTAATCGGATCGGGAGTATATCGTATTGGCAGTTCTGTTGAATTTGATTGGTGTGCTTGTGGATGTCTTCGTGAGTTAAGAAAAATCGGTAAAAAAACAATAATGGTTAATTACAATCCCGAAACT-----------------------------------------------------CACATTATTAGCCAAGAAAGAGGAAAAAAGGAAACATTCGGATCTTTAGGAATAATTTATGCTATGCTTGCAATTGGTTTATTAGGATTCGTTGTATGAGCTCACCACATATTTACTGTTGGAATAGATGTAGATACTCGAGCTTATTTCACTTCTGCAACTATAATCATTGCAGTTCCTACGGGAATTAAAATTTTTAGATGATTAGCCACTTTACATGGAGCTCAACTAAACTATTCTCCTTCTCTTTTATGAGCATTAGGATTTGTTTTCTTATTCACCGTAGGGGGATTAACCGGAGTTGTTTTAGCTAATTCTTCTATTGATATTGTTCTTCATGACACTTACTATGTTGTAGCCCACTTCCATTATGTACTTTCTATAGGAGCTGTTTTTGCTATTATAGCAGGATTTGTTCACTGATACCCTCTTTTTACTGGCTTAACTCTTAATGAAGAATGATTAAAATCTCAATTTATAATTATATTTTTAGGAGTAAATTTAACCTTTTTCCCACAACATTTCTTAGGATTAGCAGGAATACCTCGGCGTTACTCAGATTATCCAGACGCTTATACTTCATGAAACGTAGTTTCTACAATTGGGTCAACAATTTCTTTATTTGGAATTATCTTTTTTCTTTTTATTATTTGAGAAAGA

**Parakiefferiella sp. 1** ----------------GGCCATGCACCACT--ATCCTTAATTTC-A-TGAAAACGCTATCAA-ATTGTCATACCCTATTAAGTTCGG-ACCTGGTAAGTTTTCCCGTGTTGAGTCAAATTAAGCCGCAGGGTCCATTGC-CGGTGATAAACTTCCGTCAATTCCTTTAAGTTTCAACTTTGCAACCATACTTCCCCCGGAAACT--AG-CTTT-GGTTTCCCGGAAAGCTACTGAATGCACCATGAAA--AGTAGTGACATCCAATTGCTAGCTGTCATCGTTTACAGTTAGAACTAGGGCGGTATCTAATCGCCTTCGATCCTCTAACTTTCGTTCTTGATTAATGAAAACATCCTTGGCAAATGCTTTCGCTTTAGTTAGTCTTGCGACGGTCTAAGAATTTCACCTCTCGCGCCGCAATACCAATGCCCCCAACTGCTTCTATTAATCATTACCTCT-TGATCTG-TATCAAACCAACAGAAAG-CGACA---------AGCGCCCGTGAAGGCAACGAG-TCTA-TAAGACCGAGGTCTTGTTCCATTATTCCATGCAAAACTATTCAAGGCATA-AGAGCCTGCTTTGAGCACCTTAATTTGTTCAAGGTAAAGGTAAGCCGAACTAAATAGACGCCTAGCCTAATGAAAGGCATCAGCGCTATTCATTAA--GTGTTCAGTCATATAGTCCCATCAT-TCAGACATGAGAAAA-ACCGTTTTCT-GACGGAATCCC-----AATCT-------G-TGAGAGACAAAAATCAACTTCGAACGTTTT-AACCGCAACAATTTTAACATACGCTAGTGGAGCTAGAATTACCGCGGCTGCTGGCACTAGACTTGCCCTCCACTGGATCCTCATAGAAGGATTTAAAAT-CGATTCATTCCAATTACAGAGCTCAA--AAAGAGTTCTATATTGTTATTTTTCGTCACTACCTCC-----TTCAATATTCTGTCAAAAGCAATGTGCACTTCTCTCTTAGTAAGACGCTGTGATCCATTAGAAATTTATTCCAATAGTCGTTTGGCTATCAATAGGCTGGCATGAAGTTTATCGACAAATGGCTCGGTCTTTCACGGCTGGGTGTATTGTTTGGTAAGCATGCTGTTACCTGT---ATAGCTGTCTGATGGATTTTAAAGGCCATATCATTGCACCACTCAGGATA-CCCGATCAACAGCGGTTAAATTTGGGATACCTTTTGGACCCGTCTTGAAACACGGACCAAGGAGTCTATCTAGTATGCAAGTAAATGAGAAAACTTATTTGCGTAAATAACTTGACTGATGGGATTACGAGTATTCCTCCATCCCGGGGTATTTTCATCCACATATGCAAATGTATATGGAAAATATACCATGAGCATACTGGATACGACCCGAAAGATGGTGAACTATGCTTGATCAGGTTGAAGTCAGGGGAAACCCTGATGGAGGACCGAAGCAGTTCTGACGTGCAAATCGCTAACTTATCCGCTGATTGGAAATTATGGAATTCCGAGCGACGAAGAGTTCGACGAGCACAAACTGATGAAGCATTTTGAAGCCAACAACAAGATCTGGGTCGGTGGCTTGGTTGTCGGTGAATTGTGTGTAACTCCATCGCATTGGCGACAGAAGTACAAACTCGCTGAATGGATGAAGAAACACAACATTCCGGGCATCAGCGGCATCGACACGCGTGCTTTGACGAAGAAAATCCGCGAAAATGGAACTGTTTTGGGAAAAATAATTCAACGATCTTCT------GGACCGTTCCCAGGA---CTTGAGTTTAAAGATCAGAACGAGAGAAATCTTGTCGCTGAAGTGTCAACTAAATCGCCAGTTACTTACAATCCTTCCGGATCGCCTCGAATATGTGCAATCGACTGTGGCTTGAAATTGAATCAAATTCGCTGCTTCATTCAACGAGGAGCGCGAGTCGACGTTGTGCCATGGGACCATCCAGTCAATCCAGATGAATGTGATGGACTTTTTCTCTCCAACGGCCCCGGTGATCCCTTTATGTGCAAGAAAACTGTCGAAAACATCAAGAAGTTCCTTGCTTCACCGAAAGTCAAACCAATTTTTGGCATTTGTTTGGGACATCAGCTTCTTGCCACTGCAATCGGWTGCAAAACTTTCAAAATGAAGTACGGAAATCGAGGTCACAATTTGCCAGCTCTCCATCATGGCACAAATCGTTGCTTCATGACGAGCCAGAATCATGGTTTCGCGGTTGATGTTGAAACGATT------------------------------------------------------------------------------------------------------------------------------------------------------------------------------------------------------------------------------------TTTGTTCTTGCAGCAGCTCTCAAAGCTGGATACAGCGTTGATCGACTTTNCAATCTCACAAAAATCGATCGTTGGTTTTTGCAAAAGATGAAAAATATAATCGGAGTCACTTTGGAGCTGGAAAAGCTGAAT---TGTGTCATTTCTAATGAAATATTGGCTCATGCAAAGCGACTTGGTTTCAGCGATAAGCAAATCGCATCTTCGATAAAT--TTCTGAGCTCGCTGTGAGAAAGCAACGTCGTGAAGCAGGAATTTTGCCAGTWGTGAAGCAAATCGACACGGTTGCTGGTGAATGGCCTGCCTCAACAAATTATCTCTACTTGACCTACAATGGATCAGAACACGACATCGACTTCAAC---GAACAAATGGTGATGGTCATTGGATCAGGCGTTTACCGAATCGGCAGCTCAGTTGAATTCGATTGGTGTGCTGTTGGATGCCTTCGCGAGCTCAGAAATCTGGGAAAGAAGACGATTATGGTGAATTACAATCCGGAAACTGTGTCG-----------------------------------------------CACATTATTAGTCAAGAAAGAGGAAAAAAAGAAACTTTTGGAACATTAGGAATAATTTATGCTATATTAGCCATTGGACTATTAGGATTTGTTGTATGAGCCCACCACATATTTACAGTAGGTATAGATGTTGATACCCGGGCTTATTTTACATCAGCAACAATAATTATTGCAGTGCCTACAGGAATTAAAATTTTTAGATGACTAGCAACTCTTCATGGTACTCAATTAAGAAATTCCCCATCACTACTATGAACATTAGGGTTTTTATTTTTATTTACAGTAGGGGGCCTAACTGGGGTAATTCTAGCCAATTCTTCAGTAGACATTGTGCTTCATGATACCTATTATGTTGTTGCTCATTTTCACTACGTTTTATCAATAGGAGCTGTTTTTGCTATTATAGCAGGATTTATTCATTGATATTCATTATTTACTGGGTTAACTATAAATGAAAAATGATTAAAATCTCAATTTACAATTATATTTGTAGGAGTAAACTTAACTTTTTTTCCACAACATTTTTTAGGATTAGCTGGGATACCACGACGTTATTCTGATTATCCTGATGCATATATTTCATGAAATATTATTTCTACTATTGGATCAACTATTTCATTATTAGGAATTTTATTTTTTATTTTTATTATTTGAGAAAGA

**Parakiefferiella sp. 2** ---------------CGGCCATGCACCACT--ATCCTTAATTTC-A-TGAAAACGCTATCAA-ATTGTCATACCCTATTAAGTTCGG-ACCTGGTAAGTTTTCCCGTGTTGAGTCAAATTAAGCCGCAAATTCCAAGCC-ATGGTGTGAACTTCCGTCAATTCCTTTAAGTTTCAACTTTGCAACCATACTTCCCCCGGAAACT--AG-CTTT-GGTTTCCCGGAAAGCTACTGAATGCACCATGAAA--AGTAGTGACATCCAATTGCTAGCTGTCATCGTTTACAGTTAGAACTAGGGCGGTATCTAATCGCCTTCGATCCTCTAACTTTCGTTCTTGATTAATGAAAACATCCTTGGCAAATGCTTTCGCTTTAGTTAGTCTTGCGACGGTCTAAGAATTTCACCTCTCGCGCCGCAATACCAATGCCCCCAACTGCTTCTATTAATCATTACCTCT-TGATCTG-TATCAAACCAATAGAAAG-CGACA-----------CCAATTCTTGCAAACTGATGCCTA-TAAGACCGAGGTCTTTTTCCATTATTCCATGCAAAAATATTCAAGGCATA-AGAGCCTGCTTTGAGCACCTTAATTTATTCAAGGTAAAAGTAAGCCGAACTAAATAGACGCCTAGCCTAATGAAAGGTATCAGCGCTATTCATTAA--GTGTTCAGTCATATAGTTCAAATAA-TCGGAAATGATAGCA-GCCATTTACTTGATGGCGCTAC------ACCC-------G-TACTGAACAATAATCAACTTCGAACGTTTT-AACCGCAACAATTTTAATATACGCTAGTGGAGCTGGAATTACCGCGGCTGCTGGCACCAGACTTGCCCTCCACTTGATCCTCATTAAAGGATTTATACT-TGATTCATTCCAATTACAGAAC-TAAATAATTAGTTCTATATTGTTATTTTTCGTCACTACCTCC-----TTCAATATTCCTTTAAAAGCAATGTGCACTTCTCTTTTAGTAAGACGCTGTGATCCATTAGAAATTCATACCAATGATCATTCGGCTATCAATGGGCCGGCATGAAGTTCATCGACAAATAACTAGATCCTTTACGGTTTGGTATATTGTTTGGTGAGCATGTCGATACTCGT-CAATAGCTGTCTGATGGATTTTAAAGGTCATATCATTTCATCACTCAGGATA-CCCGATCAACAGCGGTTAAATTTGGGATACCTTTTGGACCCGTCTTGAAACACGGACCAAGGAGTCTATCTAGTATGCAAGTAAATGAGGAAACTTATTTGCGTAAAAAACTTGACTGATGGGATTACGTGTATACCTCCATCCCGGGGTATTTTTATCAACATATGCAAATGTATATGGAAAATATACCATGAGCATACTGGATATGACCCGAAAGATGGTGAACTATGCCTGATCAGGTTGAAGTCAGGGGAAACCCTGATGGAGGACCGAAGCAATTCTGACGTGCAAATCGTTGACTTATCCGCTGATCGGCAATTATGGCATTCCAAGTGATGAGGAGTTCGACGAGCACATGTTGATGAAGAACTTCGAGTCGAACAACAAGGTTTGGGTTTCGGGACTCGTTGTCGGTGAATTGTGTGAAACTCCATCGCACTGGCGTCAGAAATACAAACTCGCCGAATGGATGAAGAAGCACAACGTGGTCGGAATCAGCGGAATTGACACTCGCGCTTTGACGAAGAAAATTCGCGAAAATGGAACCGTTTTAGGAAAAATCATTCAAAAGTCTTGC------GGACCTTTCCCCGGT---TTGGAATTTAAAGATCAGAACGAAAGAAATCTTGTCGCCGAAGTTTCCATCAAGAAGCTAAAAACTTACAATGCTTCCGGCTCGCCGAGAATCTGCGCTGTCGATTGTGGCTTGAAGTTGAACCAGATTCGCTGCTTCATTCAACGTGGGGCTCGAGTTGATGTTGTGCCATGGGATCATCCAATCAATCCTGACGACTGTGATGGACTTTTCCTATCGAACGGACCAGGCGATCCCGTCGTGTGTGAAAAAACTGTTGCGAATCTAAAGAAATTCCTCGCTTCGCCAAAAGTCAAACCAATCTTCGGTATTTGCTTGGGTCATCAGCTCTTGTCAACCGCTGCTGGATGCAAAACTTACAAGCTAAAGTACGGAAATCGTGGTCACAACTTGCCTGCTCTTCATCATGGAACCAATCGCTGCTTTATGACGAGTCAAAATCACGGATTCGCTGTTGATGTCGATTCAATCGGCAAAGATTGGGAGCCTCTCTTCACCAACCTCAACGACTTCATCCGCGTCAGCAAGCACATCGGAAGCTCGATGAAAAGTGTCGGCGAAGTGATGGCGATCGGTCGGAAGTTTGAAGAAGCTTTTCAGAAAGCACTTCGAATGGTCGACGAGAATGTTTATGGTTTCGATCCCAACTTGAAGCCGGTGAATGATGAAGAATTGACAACGCCGACCGACAAGCGAATGTTTGTGCTCGCAGCTGCTCTGAAAGCTGGCTACACAGTCGATCGTATCTACGATTTGACGAGAATCGATCGTTGGTTCCTTGAGAAGATGAAAAACATCATCGGCGTCACGATGGAGCTCGAGAAGCTCAAC---TGCATGATTTCTGATGATTTGCTGAAGCAAGCAAAGCAACTTGGATTCTCCGACAAGCAAATCGCGGCTTCAATCAAA--CTCGGAACTAGCTGTTCGCAAACAACGTCGCGAAGCTGGAATTCTGCCGGTTGTGAAGCAAATCGACACAGTCGCTGGTGAGTGGCCGGCATCGACGAATTATCTTTACTTGACATACAACGGAACCTCACMCGMCSTCGAGTTCAAC---GWCCAGATGGTGATGGTGATTGGCTCCGGCGTTTATCGCATCGGCAGCTCTGTCGAGTTCGATTGGTGNGCAGTTGGTTGTTTGCGTGAATTGCGCAAWCTCGGCAAAAAGACGATCATGGTGAACTACAATCCGGAGACTGTGTCGACCGACTACGACATGTG------------------------------CATATTATTAGTCAAGAAAGAGGAAAAAAGGAAACATTTGGAGCTTTAGGAATAATTTATGCTATATTAGCTATTGGTTTATTAGGGTTTGTTGTATGAGCTCATCATATATTCACTGTTGGAATAGATGTAGATACACGGGCTTATTTTACTTCTGCAACTATAATTATTGCTGTTCCCACAGGAATCAAAATTTTTAGTTGATTAGCTACCCTTCATGGAACTCAATTGAATAATTCTCCATCATTATTATGATCTTTAGGATTTGTATTTTTATTTACAGTAGGAGGGTTAACAGGTGTAATTTTAGCTAATTCTTCTATTGATATTGTTCTTCATGATACTTATTATGTTGTTGCTCATTTTCATTATGTTCTTTCAATAGGAGCCGTATTTGCTATTATAGCAGGGTTTATTCATTGATATACTCTTTTCACTGGATTAACTATAAATGAAAAATGATTGAAATCTCAATTTATTATTATATTTTTAGGTGTAAATTTAACTTTTTTCCCCCAACATTTCTTAGGATTAGCTGGAATGCCTCGACGATATTCAGATTACCCGGATGCTTATATTTCATGAAATATTGTATCTACTATTGGATCAACTATTTCATTAATAGGAATTTTATTTTTTTTATTTATTATTTGAGAAAGA

**Parakiefferiella sp. 3** ------------GAACGGCCATGCACCACT--ATCCTTAATTTC-A-TGAAAACGCTATCAA-ATTGTCATACCCTATTAAGTTCGG-ACCTGGTAAGTTTTCCCGTGTTGAGTCAAATTAAGCCGCAAATTCCAAGCC-ATGGTGTGAACTTCCGTCAATTCCTTTAAGTTTCAACTTTGCAACCATACTTCCCCCGGAAACT--AG-CTTT-GGTTTCCCGGAAAGCTACTGAATGCACCATGAAA--AGTAGTGACATCCAATTGCTAGCTGTCATCGTTTACAGTTAGAACTAGGGCGGTATCTAATCGCCTTCGATCCTCTAACTTTCGTTCTTGATTAATGAAAACATCCTTGGCAAATGCTTTCGCTTTAGTTAGTCTTGCGACGGTCTAAGAATTTCACCTCTCGCGCCGCAATACCAATGCCCCCAACTGCTTCTATTAATCATTACCTCT-TGATCTG-TATCAAACCAATAGAAAG-CGACG--------CCCAACCCTTGCGAGCTGAACG-CCTA-TAAGACCGAGGTCTTTTTCCATTATTCCATGCAAAAATATTCAAGGCATA-AGAGCCTGCTTTGAGCACCTTAATTTATTCAAGGTAAAAGTAAGCCGAACTAAATAGACGCCTAGCCTAGTGAAAGGTATCAGCGCTATTCATTAA--GTGTTCAGTCATATAGTTCAAGTAA-TCGGAAATGATGGCA-GCCATTTACTTGATGGCGCCAC------ACCC-------G-TACTGAACAATAATCAACTTCGAACGTTTT-AACCGCAACAATTTTAATATACGCTAGTGGAGCTGGAATTACCGCGGCTGCTGGCACCAGACTTGCCCTCCACTTGATCCTCATTAAAGGATTTATACT-TGATTCATTCCAATTACAGAAC-TAAATAATTAGTTCTATATTGTTATTTTTCGTCACTACCTCC-----TTCAATATTCCCTTAAAAGCAATGTGCACTTCTTTCTTAGTAAGACGCTGTGATCCATTAGAAATTCATTTCCATGTTCGTTTGGCTATCAATAGGCCGGCATGAAGTTCATTGACAAGTAACTAGATCTTTTACGGTTTGGTATGTTGTTTGGTGAGCATGTTGTGTCCTGT-CAATAGCTGTCTGATGGATTTTAAAGGTCATATTATTGCACCACTCATGATAACCCGATCAACAGCGGTTAAATTTGGGATACCTTTTGGACCCGTCTTGAAACACGGACCAAGGAGTCTATCTAGTATGCGAGTAAATGAGGAAACTTATTTGCATAAAAAACTTGACTGATGGGATTACGAGTATTCCTCCATCCCGGGGTATTTTCATCAACATATGCAAATGTATATGGAAAATATACCATGAGCATACTGGATATGACCCGAAAGATGGTGAACTATGCCTGATCAGGTTGAAGTCAGGGGAAACCCTGATGGAGGACCGAAGCAATTCTGACGTGCAAATCGCTGACGTATCCGTTGATCGGCAACTATGGCATTCCAAGCGACGAAGAGTTCGATGAGCACAAGTTGATGAAGAACTTCGAGTCGAACAACAAGATTTGGGTTTCGGGACTCGTCGTCGGTGAATTGTGTGAAACTCCATCGCACTGGCGTCAGAAATACAAGCTCGCCGAATGGATGAAGAAGCACAATGTGGTTGGAATCAGCGGCATCGACACACGCGCTTTGACGAAGAAAATTCGTGAAGATGGAACCGTTTTGGGGAAAATCATTCAAAAATCTTGT------GGACCATTTCCTGGC---TTGGAATTCAAGGATCAGAACGAAAGAAATCTCGTCGCTGAAGTTTCCATCAAGAAGCCGCAAACTTACAATGCATCCGGATCACCGAGAATCTGCGCTGTTGACTGTGGCTTGAAGTTGAATCAAATTCGCTGCTTCATTCAACGTGGTGCTCGCGTTGACCTTGTGCCATGGGATCATCCACTCAATCCAGACGACTTCGACGGTCTATTCCTCTCGAACGGTCCCGGTGATCCAGTTGTTTGTGCTAAAACCGTCGCGAATCTCAAGAAATTCCTCGCTACACCGAAAGTCAAACCAATTTTCGGTATTTGCTTGGGTCATCAGCTCTTGGCGACTGCAGCTGGATGCAAAACCTACAAACTAAAGTACGGAAACCGTGGACACAACTTACCGGCGATTCACCATGGAACGAATCGTTGCTTTATGACAAGCCAGAATCATGGATTCGCTGTCGATGTCGATACAATCGGCAAAGATTGGGAGCCACTTTTCACTAATCTCAACGATTTCACTCGCGTCAGCAAACACATCGGCAGCTCGATGAAAAGTGTCGGTGAAGTCATGGCGATTGGTCGAAAATTCGAAGAAGCTTTTCAGAAAGCTTTGCGAATGGTTGATGAGACTGTGAATGGTTTCGATCCAAACTTGAAGCCGGCGCGTGACGAAGAACTCACAACACCAACTGACAAGAGAATGTTTGTCCTTGCCGCGGCTTTGAAAGAAGGTTACACGGTCGATCGACTGTATGATTTGACGAAAATCGATCGTTGGTTCCTCGAGAAGATGAAGAATATTATTGGCGTGACGATGGAGCTCGAGAAGCTCAAC---TGCATGATTTCCGACGATCTTTTGAAGCAAGCAAAGCAACTTGGATTTTCYGACAAGCAAATCGCTTCATCAATCAAA--TTCTGAGTTGGCGGTTCGCAAACAACGTCGCGAAGCTGGAATTCTTCCGGTTGTCAAGCAGATCGACACGGTTGCCGGTGAGTGGCCAGCATCGACAAACTATCTTTATTTGACATACAATGGATCTACTCACGACATTGAGTTCACC---GATCAAATGGTCATGGTGATTGGATCCGGTGTTTATCGCATCGGCAGCTCAGTTGAATTTGATTGGTGCGCAGTTGGATGTTTACGTGAGTTACGAAACCTCGGCAAAAAGACAATCATGGTGAACTACAATCCGGAGACAGTCTCGACCGACTACGACATGTGCGATCGATTGTACTTCGAAGAAATCTCGTTCATATTATTAGACAAGAAAGAGGAAAAAAGGAAACTTTTGGAAACTTAGGAATAATTTATGCTATGTTAGCTATTGGTCTATTAGGATTTATTGTTTGAGCCCATCATATATTTACAGTGGGAATAGATGTAGATACTCGAGCATATTTTACTTCAGCTACAATAATTATTGCCGTACCAACAGGAATTAAAATTTTTAGATGACTTGCAACTCTACATGGAACTCAATTAAACAACTCTCCTTCTCTATTATGAGCATTAGGGTTTGTTTTTTTATTTACTGTTGGAGGTTTAACTGGAGTAATTTTAGCTAATTCTTCTATTGATATTGTACTTCATGACACTTATTACGTTGTAGCCCATTTTCATTATGTTCTTTCCATAGGAGCAGTATTTGCAATTATAGCAGGATTTATCCATTGATATTCTTTATTTACAGGACTAACAATAAATGAAAAACTTTTGAAATCTCAATTTATTATTATATTTATTGGAGTAAATTTAACATTTTTTCCTCAACATTTCTTAGGATTAGCCGGAATACCTCGACGTTACTCAGACTATCCAGATTCTTATATTTCTTGAAATATTTTATCTACTATTGGTTCTACAATTTCTTTAATAGGAATTTTATTTTTTCTTTATATTATTTGAGAAAGT

**Parametriocnemus sp.** ---ACGAACTAAGAACGGCCATGCACCACT--ACCCTTAATTTC-G-CGAAAACGCTATCAA-ATTGTCGCACCCTATTAAGTTCGG-ACCTGGTAAGCTTTCCCGTGTTGAGTCAAATTAAGCCGCAAATTCCACGCC-ATGGTGTGGTCTTCCGTCAATTCCTTTAAGTTTCAACTTTGCAACCATACTTCCCCCGGAAACT--AG-CTTT-GGTTTCCCGGAAAGCTACTGAATGCACCATGAAA--AGTAGTGACATCCAATTGCTAGCTGTCATCGTTTACAGTTAGAACTAGGGCGGTATCTAATCGCCTTCGATCCTCTAACTTTCGTTCTTGATTAATGAAAACATCCTTGGCAAATGCTTTCGCTTTAGTTAGTCTTGCGACGGTCTAAGAATTTCACCTCTCGCGCCGCAATACTAATGCCCCCAACTGCTTCTATTAATCATTACCTCT-TGATCTG-TATCAAACCAATAGAAAG-CGACA------------TACACCGTAAAGCATATG-TCAA-TAAGACCGAGGTCTTTTTCCATTATTCCATGCAAAAATATTCAAGGCATA-AGAGCCTGCTTTGAGCACCTTAATTTGTTCAAGGTAAAAGTAAGCCGAACTAAATAGACACCTAGCCTAGTGAAAGGCATCGATGCTATTCATTAA--GTGTTCAGTCATATAGTTCGAGTAA-TCGGAAATGATGACA-ACCATTTACTTGATGGAGTCAC------ACCC-------G-TACTGAACAATAATCAACTTCGAACGTTTT-AACCGCAACAATTTTAATATACGCTAGTGGAGCTGGAATTACCGCGGCTGCTGGCACCAGACTTGCCCTCCACTTGATCCTCATTAAAGGATTTATACT-TGATTCATTCCAATTACAGAAC-TAAATAATTAGTTCTATATTGTTATTTTTCGTCACTACCTCC-----TTCAATATTCCCTTAAAAGCAATGTGCACTTCTCTCTTAGTAAGACGCTGTGATCCATTAGAAATTTGTTTCAACAATCGTATGGCTGTCAATAGGCCGGTGTGAAGTTCACTGACAAATAACTCAATCATTTACGGTTGGGTATATTGTTTGGTGAGCTCACCGTCACCTGTACAATGGCTGTCTGATGGATTTTAAAGGCTATGATATTGCATCACTCGGGTCA-CCCGATCAACAGCGGTTAAATTTGGGATACCTTTTGGACCCGTCTTGAAACACGGACCAAGGAGTCTATCTAGTATGCGAGTAAGTGAGTAAACTTATTTGCGTAAAAAACTTGACTGATGGGACTACGAGTTCTCTTCCATCCCGGGGTATTTCTATCAACATATGCAAATGTATATGGGAAATATACCATGAGCATACTGGATATGACCCGAAAGATGGTGAACTATGCCTGATCAGGTTGAAGTCAGGGGAAACCCTGATGGAGGACCGAAGCAATTCTGACGTGCAAATCG---------------------------------------------------------------TTGATGAAAAATTTCGAGTCGAATGACAAGATTTGGGTTTCGGGATTAGTGGTTGGTGAACTTTGCGAAACGCCTTCGCACTGGAGGCAAAAGTATAAGCTCGCTGAATGGATGAAGAAGCATAATGTCGTCGGAATCAGTGGAATCGACACGCGTGCTTTAACCAAGAGAATTCGAGAGAACGGAACGATTTTAGGAAAGATTATTCAACAATCGGAG------GGTCCGTTTCCAGGC---TTAGAGTTCAAAGATCAAAATGAAAGGAATTTGGTTGACGAAGTGTCGCTCAAGAAGGTTGTAACTTACAACCCCAAAGGGTCACCAAGAATCTGTGCTGTTGATTGTGGACTGAAATCGAATCAACTTCGATGCTTTATTCAACGTGGAGCTCGTGTTGATTTGGTGCCATGGGACCATCCTCTGAATCCTAATGATTTCGATGGACTCTTCCTTAGCAACGGTCCTGGCGATCCCGTTATGTGTAAAAAAACTGTTACAAATCTTCAAAATTTTCTCGCTTCCAATCAAATTAAGCCAATATTTGGCATTTGCTTAGGCCATCAATTGCTTGCCACTGCTGCTGGATGCAAAACTTACAAATTGAAGTATGGAAATCGTGGTCATAATCTGCCAGCTCTMCATCATGGYACCAATCGATGTTTTATGACATCGCAAAATCATGGATTTGCTGTTGACACTAAGACAATTGGAAAGGATTGGGAACCGCTTTTTACGAACTTA------TTCACGAGAGTCAGCAAAAATATCGGCAGCTCTATGAAGTCTGTGGGTGAAGTGATGGCGATTGGTAGGAAGTTCGAAGAAGCTTTTCAAAAAGCTCTTCGAATGGTTGACGAAAACGTTAATGGATTCGATCCCAACCTGAAGCCTGTGGTTGATGAAGAATTGAAGTCACCGACTGATAAGAGAATGTTTGTGTTGGCTGCTGCACTCAAAGCGGGTTACACCGTTGACAGGCTCTACGATTTGACGAAGATCGACCGATGGTTCCTCGTAAAAATGAAAAATATTATCGATATTACTTTGGATCTGGAGAAGCTCAAT---TGCGTAATTCCAGATGAACTGCTGAGGCTGTCAAAGAAATTGGGATTTTCTGATAARCAAATTGCGACWTACATCAAG--CTCCGAGTTGGCGGTGCGCAAACAGAGAAGAGAAGCTGGAATACTTCCATTTGTTAAGCAAATCGACACTGTYGCTGGTGAATGGCCTGCATCGACAAACTACCTTTACCTAACTTATAACGCAAGCGAACATGACATTGAGTTCAAT---GAYCAGATGGTGATGGTGATTGGATCTGGAGTTTATCGAATCGGCAGCTCAGTGGAATTCGATTGGTGCGCTGTTGGTTGTTTGAGGGAATTGAGAAACCTTGGAAAGAAAACGATTATGGTCAACTACAACCCCGAAACTGTTTCCACCGAYTACGACATGTGCGATCGACTTTATTTTGAAGAAATCTCGTTCATATTATTAGACAAGAAAGTGGAAAAAAGGAAACTTTTGGAGCCTTAGGAATAATTTATGCAATATTAGCAATTGGTTTATTAGGGTTTGTTGTATGAGCTCATCATATATTTACTGTAGGAATAGATGTGGATACCCGCGCATATTTTACATCAGCTACTATAATTATTGCAGTACCTACTGGAATTAAAATTTTTAGTTGATTGGCAACTTTACATGGAACTCAATTGAATAATTCTCCTTCTTTATTATGAGCATTAGGATTTGTATTTTTATTTACTGTAGGGGGTTTAACAGGAGTAATCCTTGCAAATTCTTCTATTGATATTGTTCTTCATGATACTTATTATGTTGTAGCTCACTTTCACTATGTATTATCAATAGGAGCTGTATTTGCTATTATAGCAGGATTTGTGCACTGATACTCATTATTTACAGGTCTAACAATAAATGAAAAATGATTAAAATCTCAATTTGCAATTATATTTTTAGGAGTCAATTTAACATTTTTTCCTCAACATTTTTTAGGATTAGCCGGGATACCTCGACGATACTCAGACTATCCTGACGCTTACACTTCTTGAAATATTATTTCAACTGTAGGATCAACAATTTCATTATTTGGGATTTTATTTTTCATTTTTATTATTTGAGAAAGA

**Parametriocnemus sp. 2** --------CTAAGAACGGCCATGCACCACT--ACCCTTAATTTC-G-CGAAAACGCTATCAA-ATTGTCGCACCCTATTAAGTTCGG-ACCTGGTAAGCTTTCCCGTGTTGAGTCAAATTAAGCCGCAAATTCCACGCC-ATGGTGTGGTCTTCCGTCAATTCCTTTAAGTTTCAACTTTGCAACCATACTTCCCCCGGAAACT--AG-CTTT-GGTTTCCCGGAAAGCTACTGAATGCACCATGAAA--AGTAGTGACATCCAATTGCTAGCTGTCATCGTTTACAGTTAGAACTAGGGCGGTATCTAATCGCCTTCGATCCTCTAACTTTCGTTCTTGATTAATGAAAACATCCTTGGCAAATGCTTTCGCTTTAGTTAGTCTTGCGACGGTCTAAGAATTTCACCTCTCGCGCCGCAATACTAATGCCCCCAACTGCTTCTATTAATCATTACCTCT-TGATCTG-TATCAAACCAATAGAAAG-CGACA------------TACACCGTGAAGCATACA-TCAA-TAAGACCGAGGTCTTTTTCCATTATTCCATGCAAAAATATTCAAGGCATA-AGAGCCTGCTTTGAGCACCTTAATTTGTTCAAGGTAAAAGTAAGCCGAACTAAATAGACACCTAGCCTAGTGAAAGGCATCGATGCTATTCATTAA--GTGTTCAGTCATATAGTTCGAGTAA-TCGGAAATGATGACA-ACCATTTACTTGATGGAGTCAC------ACCC-------G-TACTGAACAATAATCAACTTCGAACGTTTT-AACCGCAACAATTTTAATATACGCTAGTGGAGCTGGAATTACCGCGGCTGCTGGCACCAGACTTGCCCTCCACTTGATCCTCATTAAAGGATTTATACT-TGATTCATTCCAATTACAGAAC-TAAATAATTAGTTCTATATTGTTATTTTTCGTCACTACCTCC-----TTCAATATTCCCTCAAAAGCAATGTGCACTTCTCTCTTAGTAAGACGCTGTGATCCATTAGAAATTTGTTCCAACAATCGTATGGCTGTCAATAGGCCGGTGTGAAGTTCACTAGCAAATAACTCGATCCTTTACGGTTGGGTATATTGTCTGGTGAGCTCACCGTAACCTGTACCATGGCTGTCTGATGGATTTTAAAGGCTTTGACATTGCATCACTCAGGTCA-CCCGATCAACAGCGGTTAAATTTGGGATACCTTTTGGACCCGTCTTGAAACACGGACCAAGGAGTCTATCTAGTATGCGAGTAAGTGAGGAAACTTATTTGCGTAAAAAACTTGACTGATGGGACTACGAGTTCTCTTCCATCCCGGGGTATTTCTATCAACATATGCAAATGTATATGGGAAATATACCATGAGCATACTGGATATGACCCGAAAGATGGTGAACTATGCCTGATCAGGTTGAAGTCAGGGGAAACCCTGATGGAGGACCGAAGCAATTCTGACGTGCAAATCG---------------------------------CCGAGCGATGAAGAGTTTGACGAGCATAAATTGATGAAGAATTTTGAGTCGAATAACAAGATTTGGGTTTCGGGTTTRGTGGTTGGTGAACTTTGTGAAACTCCNTCGCACTGGAGGGCGAAGTATAAGCTTGCCGAATGGATGAAGAAGCACAATGTCGTTGGCATCAGTGGAATTGACACGCGCGCATTGACCAAGAAGATTCGAGAGAATGGAACGATTTTAGGAAAGATTATTCAGCAATCTTCG------GGTCCATTTCCTGGG---TTGGAATTTAAAGATCAGAACGAGAGGAATTTRGTTGATGAGGTTTCGATTAAGAAGACGGTAACGTACAACGCTAAAGGATCGCCGAGAATTTGTGCTGTTGATTGTGGATTAAAGTCGAATCAGCTTCGATGCTTTATTCAACGTGGAGCTCGTGTTGATTTGGTGCCATGGGATCATCCTTTGAACCCCGATGACTTTGATGGACTCTTTCTTAGCAATGGTCCRGGCGATCCCGTTATATGTCAGAAAACTGTTAAAAACCTTCAGAAATTTCTCGCTTCCAATAAAGTCAAGCCAACTTTTGGTATTTGCTTAGGTCATCAATTGCTGGCWACTGCTGTCGGATGCAAAACCTACAAATTGAAATACGGAAATCGTGGTCATAATCTGCCAGCTCTCCATCATGGCACTAATCGATGCTTTATGACGTCACAGAATCATGGATTCGCTGTTGAYGCTACGACCATTGGAAAGGATTGGGAGCCGCTTTTCACGAATTTAAATGACTTCACCAGAGTCAGTAAGAACATCGGCAGCTCAATGAAATCGGTTGGTGAGGTGATGGCGATTGGCAGAAAGTTCGAGGAAGCTTTTCAAAAAGCTCTGCGAATGGTTGATGAAAATGTCAATGGATTCGATCCCAACTTGAAGCCGGTCATTGATGAAGAGTTGAARACACCGACGGATAAGAGAATGTTTGTTCTTGCCGCTGCACTTAAAGCTGGTTATACTGTTGATAGACTTTATGACTTAACGAAGATCGATCGATGGTTTTTGGTGAAGATGAAGAATATTATTGATATAACTTTAGACCTTGAGAAGCTTAAC---TGCGTAATTCCTGACGAGCTTTTAAAGCAAGCAAAGAAATTGGGATTTTCTGATAAGCAAATTGCGACTTATATTCGA--TTCTGAGTTGGCGGTTCGCAAACAACGAAGGGAAGCTGGAATTTTGCCATTTGTCAAGCAAATTGATACCGTCGCAGGTGAATGGCCGGCATCAACAAATTATCTTTACTTGACTTACAATGCCAGCGAACATGACATTGAGTTTGAT---GACCAGATGGTGATGGTTATTGGATCTGGAGTTTATAGAATTGGCAGCTCAGTGGAGTTCGATTGGTGCGCTGTTGGATGTTTGAGGGAGTTGAGGAATTTGGGRAAGAAAACAATTATGGTCAACTACAACCCAGAAACTGTTTCAACTGATTACGACATGTGCGATCGACTTTATTTCGARGAAATTTCATTCATATTATTAGACAAGAAAGTGGAAAAAAAGAAACCTTTGGAGCATTAGGAATAATTTATGCTATGCTTGCAATTGGTTTATTAGGCTTTGTTGTATGAGCTCATCATATATTTACTGTAGGAATAGACGTAGACACACGAGCTTATTTTACATCTGCTACAATAATTATTGCAGTTCCTACAGGAATTAAAATTTTTAGATGATTAGCTACTATTCATGGAACTCAATTAAATAACTCCCCTTCTTTATTATGAGCATTAGGTTTTGTTTTCTTATTTACTGTAGGAGGATTAACAGGAGTAATTTTAGCTAATTCATCTATTGACATTGTCCTACATGATACTTACTATGTTGTTGCTCATTTTCACTATGTATTATCTATAGGAGCAGTATTTGCTATTATAGCAGGATTTGTACATTGATATTCATTATTTACAGGATTAACAATAAATGAAAAATGATTGAAATCTCAATTTGCAATAATATTTTTAGGGGTAAATTTAACCTTTTTTCCTCAACATTTTTTAGGATTAGCAGGAATACCACGACGATATTCTGATTACCCTGACGCATATACATCTTGAAATATTGTTTCAACTGTAGGATCAACAATTTCTTTATTTGGAATTTTATTTTTTATTTTCATTATCTGAGAAAGA

**Parapsectrocladius acuminatus** ------------------------------------------------------------------------CCCTATTAAGTTCGG-ACCTGGTAAGTTTTCCCGTGTTGAGTCAAATTAAGCCGCAAATTCCAATTT-TTGTAGTAAACTTCCGTCAATTCCTTTAAGTTTCAACTTTGCAACCATACTTCCCCCGGAAACT--AG-CTTT-GGTTTCCCTGAAAGCTACTGAATGCACCATGAAA--AGTAGTGACATCCAATTGCTAGCTGTCATCGTTTACAGTTAGAACTAGGGCGGTATCTAATCGCCTTCGATCCTCTAACTTTCGTTCTTGATTAATGAAAACATCCTTGGCAAATGCTTTCGCTTTAGTTAGTCTTGCGACGGTCTAAGAATTTCACCTCTCGCGCCGCAATACTAATGCCCCCGACTGCTTCTATTAATCATTACCTCT-TGATCTG-TATCAAACCAATAGAAAG-CGGCG------------CAACCCTTGCAGATTACG-CCTA-TAAGACCGAGGTCTTTTTCCATTATTCCATGCAAAAATATTCAAGGCATT-AGAGCCTGCTTTGAGCACCTTAATTTGTTCAAGGTAAAAGTAAGCCGAACTAAATAGACACTTAGCCTAGTGAAAGGCATCGGTGCTATTCATTAA--GTGTTCAGTCATATAGTTCAAGTAA-TCGGAAATGATGACA-GCCATTTACTTGATGGCGTCAC------ACCC-------G-TACTGAACAATAATCAACTTCGAACGTTTT-AACCGCAACAATTTTAATATACGCTAGTGGAGCTGGAATTACCGCGGCTGCTGGCACCAGACTTGCCCTCCACTTGATCCTCATTTAAGGATTTATACT-AGATTCATTCCAATTACAGAAC-TAAATAA----------------------------------------TTCAATATTAAGGTAACTTCAATGTGCACTTCTTACTTAGTAAGACGCTGTGATCCATTAGAAATTCATTCCAATGGTTATTTGGCCATCATTGGGTTGACGTTAAGTTCACTAGCAAATGACTTGATCTTATATGGTTGGGTGTATTGTATGGTGAGTTCGTTGAATCCCGT---ATGGCTGTCTGATGGATTTTAAAGGTTATAACATTGTATCACCCAGGTTAACTCGATCAACAGCGGTTAAATTTGGGATACCTTTTGGACCCGTCTTGAAACACGGACCAAGGAGTCTATCTATTACGCAAGTAAATGAGGAAACTTATTTGCATAAAAAACTTGACTGATGGGATTACGAGCGCTTCTCCATCCCGGGGTATTTTTATCAACACATGCAAATGTATGTGGAAAATATACCATGAGCGTAGTGGATATGACCCGAAAGATGGTGAACTATGCCTGATCAGGTTGAAGTCAGGGGAAACTCTGATGGAGGACCGAAGCAGTTCTGACGTGCAAATCGTTAACTTATCCATTGATTGGGAACTACGGAATTCCGAATGACGAAGAATTTGATGAAGACAATTTAATGAAACACTTCGAGTCCAACGGGAAGATTTGGGTTTCTGGCCTGGTCGTCGGGGAATTGTGCGACACACCATCGCATTGGCGTCAGAAGTACAAGCTTGCCGTATGGATGAAGAAGCACAACATTGTTGGCATCAGTGGCGTCGACACMCGCGCATTGACGAAGAAAATTCGCGAGAGTGGAACATTCTTGGGAAAAATCATCCAGCAATCGAGC------GGCCCTTTTCCAGGG---TTAGAATTTGTCGACCAAAATCAACGGAACTTAGTTGAAGAAGTTTCAATTAAAACTCCAATCACTTACAACGCCACTGGATCKCCAAGAATYTGTGCTGTTGACTGTGGGTTGAAATTAAATCAAATTCGTTGTTTTACCAAACGCGGCGCTCGTGTGGATTTGGTGCCTTGGGATTATCCGTTAAAAGTTGGAGATTTCGATGGACTTTTCTTGTCTAACGGACCTGGAGATCCCGTCATGTGCAAGACAACTGTAGAAAACATCCGGAAAATTGTTGTCGGCTCGCAGGTAAAACCGATTTTCGGGATTTGCCTTGGCCATCAATTGCTGGCATCGGCGATTGGTTGTGAAACTTATAARATGAAATATGGCAATCGAGGACACAATTTACCTGCTTTACATAATGGAACCAATCGCTGCTTTATGACGTCTCAGAACCATGGATTTGCTGTTGATGCAGCGACTCTTCCTGATGATTGG---------------------------TTCATCCGTGTGAGTAAACATATCGGAAGTTCAATGAAATCTGTCGGTGAAGTCATGGCAATTGGTCGGAAGTTCGAAGAAGCTTTCCAGAAAGCGTTGCGGATGGTTGACGAGAATGTCAATGGATTCGATCCGAATTTAAAGCAAGTCGTTGATGAAGAACTGACGTCACCGACTGATAAACGAATCTTCGTTCTTGCTGCGGCATTGAAAGCCGGATACAGTATCGATCGGCTGTACGAACTCACGAAAATTGATCGATGGTTCTTAAATAAGATGAGAAAAATCATCGAAGTGACGGTGGAACTTGAGAAGCTTAAT---TGTGTCGTTTCAAACGATCTCCTCCGACAAGCGAAGACAATTGGATTTTCTGACAAACAAATCGCTCAATTTATCAAA--ATCCGAATTGGCAGTACGAAAGCAACGTCGGGAATGTGGAATCTTGCCATTTGTGAAGCAAATCGACACTGTTGCCGGTGAATGGCCAGCGTCGACTAATTACCTGTACTTAACCTACAATGCAAGCAGTCACGACGTAGAATTCAAT---GATCAGATGGTCATGGTGATTGGATCTGGAGTTTATCGCATCGGAAGTTCCGTGGAGTTTGATTGGTGTGCGGTTGGATGTCTGCGGGAATTACGGAAACTCGGGAAGAAGACAATCATGGTTAATTACAATCCAGAGACGGTGTCGACTGATTACGACATGTGCGATCGTTTGTATTTCGAGGAAATTTCCTTCATATTATTAGACAAGAAAGTGGAAAAAAAGAAACTTTTGGAGCTTTAGGAATAATTTATGCAATACTAGCAATCGGACTTTTAGGTTTTGTTGTATGAGCCCATCATATATTTACAGTTGGAATAGATGTTGATACACGAGCTTATTTTACTTCTGCTACAATAATTATTGCAGTACCAACAGGAATTAAAATTTTTAGATGATTAGCAACTTTACATGGAACTCAATTAAAAAACTCCCCTTCTTTATTATGAGCTTTAGGATTTGTTTTTCTTTTTACTGTTGGGGGGCTAACTGGTGTAGTACTAGCAAATTCTTCTATTGATATTGTTCTACACGATACCTACTATGTAGTAGCTCATTTTCATTATGTTCTTTCTATAGGAGCAGTATTTGCTATTATAGCAGGATTTACTCATTGATATACATTATTTACAGGCCTTTCATTAAATGAAAAATGGTTAAAATCTCAATTTGGAATTATATTTTTAGGGGTTAATTTAACTTTTTTCCCTCAACATTTTTTAGGTTTAGCAGGAATACCTCGACGATACTCAGATTATCCTGATGCCTATACTTCATGAAATATTATTTCAACTATTGGATCAACTATCTCTTTATTTGGAATTTTATACTTTATTTTTATTATATGAGAAAGA

**Paratrichocladius sp.** --------------------------------------AATTTA-G-CGAAAGCGCTATCAA-ACTGTCGTACCCTATTAAGTTCGG-ACCTGGTAAGTTTTCCCGTGTTGAGTCAAATTAAGCCGCAAATTCCAAATC-ATTGTGTCAAATTCCGTCAATTCCTTTAAGTTTCAACTTTGCAACCATACTTCCCCCGGAAACT--AG-CTTT-GGTTTCCCTGAAAGCTACTGAATGCACCATATAA--AGTAGTGACATCCAATTGCTAGCTGTCATCGTTTACAGTTAGAACTAGGGCGGTATCTAATCGCCTTCGATCCTCTAACTTTCGTTCTTGATTAATGAAAACATCCTTGGCAAATGCTTTCGCTTTAGTTAGTCTTGCGACGGTCTAAGAATTTCACCTCTCGCGCCGCAATACTAATGCCCCCGACTGCTTCTATTAATCATTACCTCT-TGATCTG-TATCAAACCAATAGAAAA-TCACA------------------TTATTGCTAATGCGATA-TAAGACCGAGGTCTTTTTCCATTATTCCATGCAAAAATATTCAAGGCATT-AGAGCCTGCTTTGAGCACCTTAATTTGTTCAAGGTAAAATTAAGCCGAACTAAATAGACACTTAGCCTAGTGAAAGGCATCGGTGCTATTCATTAA--GTGTTCAGTCATGTAGTTCAAGCAA-TCGAAAGTGATAGCA-GTCATTTACTTGATGACGCCAC------ACTC-------G-TGCTGAACAAAAATGAACTTCGAACGTTTT-AACCGCAACAATTTTAACATACGCTAGTGGAGCTGGAATTACCGCGGCTGCTGGCACCAGACTTGCCCTCCACTTGATCCTCATTGAAGGATTTATACT-CGATTCATTCCAATTACAGAAC-TAAATAATTAGTTCTATATTGTTATTTTT------------------TTCAATATTTTGGCAACAACAATGTGCACTTCTCATTTAGTAGGACGTTGTGATCCATTAGAAATTTGTATCAACAATTATGAGGTTATCAATAGACTGATGTAAAGTTCATCAATAAATGATTTGATCTTTAATGGTTGGGTGTATTATATGGTGAGTGCGTCATGTACTGC-TTATAACTGTCTGATGGATTTTAAAGGCTATAATATTGCATCACCCTGATTA-CCCGATCAACAACGGTTAAATTTGGGATACCTTTTGGACCCGTCTTGAAACACGGACCAAGGAGTCTATCTATTGCGCGAGTAAATGGGGAAACCTATTTGCGTAAATAACTTGACTGATGGGATTACAAGT-CTTCTCCATCCCGGGGTATTTTTATCAACACATGCAAATGTATGTGGAAAATATACCATGAGCGTAGTGGATATGACCCGAAAGATGGTGAACTATGCCTGATCAGGTTGAAGTCAGGGGAAACCCTGATGGAGGACCGAAGCAATTCTGACGTGCAAATCGCTTACGTATCCTTTGATCGGCAATTATGGGATCCCCAGCGACGAAGATTTTGACGACCACAAATTGATGAAACATTTCGAATCGAACAACAAGATTTGGGTTTCAGGTCTCGTTGTCGGTGAATTGTGTGAGACTCCGTCGCACTGGCGTCAAAAGTACAAACTCGCTGAATGGATGAAGAAGCACAATATTCCTGGTATCAGTGGAATTGACACTCGCGCATTGACCAAGAAAATTCGCGAGAATGGAACAATTTTAGGGAAAATTATTCAACAATCCGCT------GGACCTTTCCCTGAT---CTCGAGTTCAAGGATCAAAATCAGAGGAATTTAGTTGATGAAGTTTCCATTAAAAATCCAATTACATACAACGAATCAGGCTCACCTCGAATTTGTGCTGTTGATTGTGGTTTGAAGCTAAATCAAATTCGCTGCTTCGTGAAACGAGGTGCAAGAGTTGATGTCGTGCCATGGAATCATTCTCTAAACCCGAAAGACTTTGACGGCCTCTTCCTCTCCAATGGGCCAGGAGATCCCGTTGTGTGCGCTAAGACTGTTGAGAACATTCAGAAAGTTTTGTCTTCGCCGCAACTCAAACCAGTTTTCGGAATTTGTCTTGGACATCAACTGCTAGCTACTGCTGTCGGCTGCAAAACTTACAARATGAAATATGGTAATCGTGGTCACAATCTACCAGCGCTACATCACGGTACTAATCGCTGCTATATGACATCTCAGAACCACGGATTTGCTGTCGATACAAGCTCGATGCCGAAGGATTGGGAGCCACTCTTCACAAATCTTAACGACTTCATCAGAGTCAGCAAAAACATCGGAAGTTCGATGAAAAGTGTCGGCGAGGTGATGGCGATTGGAAGAAATTTCGAAGAAGCTTTCCAGAAAGCGTTGAGGATGGTGGATGAGAATGTGAATGGATTTGATCCAAATCTAAAGCCAGTGGTGGATGAGGAACTTAAGACACCGACAGATAAAAGAATTTTTGTACTTGCTGCTGCGTTGAAAGCTGGCTACACGATTGACCGTCTCTATGACCTCACAAAAATCGATCGTTGGTTCCTCGAAAAAATGAAAAACATCATAAATGTCACATTAGAGTTGGAAAAGCTCAAC---TGCATCGTTTCCAATGAACTTCTTAGTCAATCGAAAAAAATGGGTTTCTCYGACAAGCAAATCGCCAAGTACATAAAA--ATCGGAACTTGCGGTGAGAAAGCAGCGACGTGAGAGTGGAATTCTTCCGTTTGTAAAGCAAATCGACACTGTGGCTGGTGAATGGCCAGCATCTACCAACTATCTCTACTTGACTTACAATGCATCAGCTCATGATATCGACTTTAAG---GAACCGATGGTGATGGTGATTGGATCTGGTGTTTATCGCATCGGGAGCTCAGTTGAGTTCGATTGGTGTGCTGTTGGGTGTTTGAGGGAGTTGAGGAATCTTGGCAAAAAGACAATTATGGTGAACTACAATCCTGAAACGGTTTCAACTGATTATGACATGTGCGATCGTTTGTACTTTGAGGAAATATCTTTCACATTATTAGTCAAGAAAGAGGAAAAAAAGAAACATTTGGTTCTTTAGGTATAATTTATGCTATATTAGCTATTGGATTACTAGGATTTGTAGTTTGAGCCCATCATATATTTACTGTAGGAATAGATGTAGATACCCGAGCTTATTTCACTTCAGCCACAATAATTATTGCTGTACCTACAGGAATTAAAATTTTTAGTTGATTAGCAACTTTACATGGAACTCAATTAAATAATTCTCCCTCTCTTCTCTGAGCCTTAGGGTTTGTTTTTTTATTTACTGTAGGGGGTTTAACAGGAGTAATTTTAGCCAATTCTTCTATTGATATTGTACTTCATGATACTTATTATGTTGTTGCTCATTTTCATTATGTTCTTTCAATAGGAGCTGTATTTGCTATTATAGCTGGATTTGTTCATTGATACTCTTTATTTACAGGATTAACAATAAATGAAAAATGATTAAAATCTCAATTTTCAATTATATTCTTGGGTGTAAATTTAACCTTTTTCCCCCAACATTTTTTAGGCCTAGCTGGAATGCCTCGTCGATATTCAGATTATCCAGATGCTTATACATCTTGAAATATTATTTCTACAATCGGTTCAACGATTTCTTTATTTGGAATTTTATTTTTTTTATTTATTATTTGAGAAAGA

**Paulfreemania pictipennis** TCCACGAACTAAGAACGGCCATGCACCACT--ACCCTTAATTTC-A-AGAAAACGCTATCAA-GTTGTCATACCCTATTAAGTTCGG-ACCTGGTAAGTTTTCCCGTGTTGAGTCAAATTAAGCCGCAAATTCCAAGTC-ATGGTGTGAACTTCCGTCAATTCCTTTAAGTTTCAACTTTGCAACCATACTTCCCCCGGAAACT--AG-CTTT-GGTTTCCCGGAAAGCTACTGAATGCACCATGAAA--AGTAGTGACATCCAATTGCTAGCTGTCATCGTTTACAGTTAGAACTAGGGCGGTATCTAATCGCCTTCGATCCTCTAACTTTCGTTCTTGATTAATGAAAACATCCTTGGCAAATGCTTTCGCTTTAGTTAGTCTTGCGACGGTCTAAGAATTTCACCTCTCGCGCCGCAATACTAATGCCCCCAACTGCTTCTATTAATCATTACCTCT-TGATCTG-TATCAAACCAATAGAAAG-CGACG------------CTACGGTTGCCCGCAACG-CCTA-TAAGACCGAGGTCTTTTTCCATTATTCCATGCAAAAATATTCAAGGCATA-AGAGCCTGCTTTGA--------------------------------------------------------------------------------------------------------------------------------------------------------------------------------------------------------------------------------------------------------------------------------------------------------------------------------------------------------------------------------TTCAATATTCCCTCAAAAGCAATGTGCACTTCTCCCTTAGTAAGACGCTGTGATCCATTAGAAATTCATTCCAATGATTATTTGGCTATCAATGGGCCGGTATGAAGTTCTTCAGCAAATAACTCGATCCTTTACGGTTGGGTATATTGTTTGTTGAGCATGCCGTCACCCGT-CTATAGCTGTCTGATGGATTTTAAAGGTCATATTATTGCATCACTCAGAATAACCCGATCAACAGTGGTTAAATTTGGGATACCTTTTGGACCCGTCTTGAAACACGGACCAAGGAGTCTATCTAGTATGCAAGTAAATGAGGAAACTTATTTGCAGAAAAAACTTGACTGATGGGACTACGAGTGCTCCTCCATCCCGGGGTATTTTTATCAACATATGCAAATGTATATGGAAAATATACCATGAGCGTACTGGATATGACCCGAAAGATGGTGAACTATGCCTGATCAGGTTGAAGTCAGGGGAAACCCTGATGGAGGACCGAAGCAATTCTGACGTGCAAATCG---------------ATTGGAAATTATGGGATTCCGAGTGATGAAGATTTCGATGAGCACAAGATGATGCAGCACTTTGAGTCCAATAATAAAATCTGGGTTTCCGGTCTTGTTGTTGGTGAGTTATGCGAGACACCGTCGCATTGGAGACAGAAATACAAGCTCGCGGAATGGATGAAGAAGCACAATGTCACTGGCATCAGTGGAATCGACACTCGCGCTTTGACTAAACGAATTCGTGAAGATGGAACGGTTCTTGGTAAAATTATTCATCAATCTGCT------GGTCCATTTCCAGGA---TTGGAGTTTAAGGATCAAAACCAGCGTAATCTCGTTGATGAAGTTTCGATTAAAAAGCCAATCACGTACAATTCGAAAGGCTCGCCGACAATTTGTGCCATCGATTGCGGACTGAAATTGAATCAGATTCGTTGCTTTGTCAATCGCGGAGCTCGTGTCGATGTGGTGCCACGGGACCATCTATTGAATCCCGATGCTTTTGATGGACTCTTTCTCAGCAACGGTCCTGGTGATCCCGTCGTGTGCCAAAAGACTGTCAAAAATATTCAAAATTTCCTCGCATCGCCTAAAGTGAAACCCATTTTTGGGATCTGCTTGGGTCATCAATTGCTCTCAACTGCCATCGGCTGTAAAACTTACAAATTAAAATATGGAAATCGAGGTCACAATCTTCCAGCTCTCCATCATGGAACCAATCGTTGCTTCATGACCTCTCAAAATCACGGATTCGCTGTTGATGTCAAAACAATTGCTAAGGATTGGGAGCCGCTCTTCACCAATCTCAATGACNNNNNNNNNNNNNNNNNNNNNNNNNNNNNNNNNNNNNNNNNNNNNNNNNNNNNNNNNNNNNNNNNNNNNNNNNNNNNNNNNNNNNNNNNNNNNNNNNNNNNNNNNNNNNNNNNNNNNNNNNNNNNNNNNNNNNNNNNNNNNNNNNNNNNNNNNNNNNNNNNNNNNNNNNNNNNNNNNNNNNNNNNNNNNNNNNNNNNNNNNNNNNNNNNNNNNNNNNNNNNNNNNNNNNNNNNNNNNNNNNNNNNNNNNNNNNNNNNNNNNNNNNNNNNNNNNNNNNNNNNNNNNNNNNNNNNNNNNNNNNNNNNNNNNNNNNNNNNNNNNNNNNNNNNNNNNNNNNNNNNNNNNNNNNNNNNNNNNNNNNNNNNNNNNNNNNNNNNNNNNNNNNNNNNNNNNNNNNNNNNNNNNNNNNNNNNNNNNNNNNNNNNNNNNNNNNNNNNNNNNNNNNNNNNNNNNNNNNNNNNNNNNNNNNNNNNNNNNNNNNNNNNNNNNNNNNNNNNNNNNNNNNNNNNNNNNNNNNNNNNNNNNNNNNNNNNNNNNNNNNNNNNNNNNNNNNNNNNNNNNNNNNNNNNNNNNNNNNNNNNNNNNNNNNNNNNNNNNNNNNNNNNNNNNNNNNNNNNNNNNNNNNNNNNNNNNNNNNNNNNNNNNNNNNNNNNNNNNNNNNNNNNNNNNNNNNNNNNNNNNNNNNNNNNNNNNNNNNNNNNNNNNNNNNNNNNNNNNNNNNNNNNNNNNNNNNNNNNNNNNNNNNNNNNCATATCATTAGTCAAGAAAGAGGAAAAAAGGAAACTTTTGGAGCTTTAGGAATAATTTATGCCATGCTTGCCATTGGGTTGTTAGGGTTTATTGTTTGAGCCCATCACATATTTACAGTAGGAATAGATGTTGATACCCGAGCCTACTTCACATCTGCCACTATAATTATTGCCGTACCAACAGGTATCAAAATTTTTAGTTGACTTGCTACTCTTCACGGAACTCCTCTAAATAATTCACCTTCTTTATTATGAGCTTTAGGATTTGTTTTCCTTTTTACGGTTGGGGGGTTAACAGGAGTAATTTTAGCTAATTCTTCCATCGACATTGTTCTTCATGACACTTACTATGTAGTTGCTCATTTTCACTATGTTTTATCTATAGGGGCAGTCTTTGCTATTATGGCCGGATTTGTACACTGATACACTTTATTTACTGGTTTAATTATGAATGAAAAATGATTAAAGTCTCAATTTGCTATTATATTTTTAGGAGTAAATCTAACTTTCTTCCCTCAACATTTTTTAGGTTTAGCAGGGATACCTCGACGTTACTCAGACTATCCAGATGCCTATACTTCTTGAAATATTTTGTCTACTGTTGGCTCAACAATTTCTCTATTTGGGATTCTATTTTTTATTTTTATCATTTGAGAAAGC

**Prodiamesa sp.** ---------------CGGCCATGCACCACT--ACCCTTAATTTC-A-AGAAAACGCTGTCAA-GCTGTCTAACCCTATTAAGTTCGG-ACCTGGTAAGTTTTCCCGTGTTGAGTCAAATTAAGCCGCAAATTCCAGACC-TGGTGGTGCCCTTCCGTCAATTCCTTTAAGTTTCAACTTTGCAACCATACTTCCCCCGGAAACT--AG-CTTT-GGTTTCCCGGAAAGCTACTGAATGCACCATGAAT--AGTAGTGACATCCAATTGCTAGCTGTCATAGTTTACAGTTAGAACTAGGGCGGTATCTAATCGCCTTCGATCCTCTAACTTTCGTTCTTGATTAATGAAAACATCCTTGGCAAATGCTTTCGCTTTAGTTAGTCTTACGACGGTCTAAGAATTTCACCTCTCGCGCCGTAATACTAATGCCCCCAACTGCTTCTATTAATCATTACCTCT-TGATCTG-TATCAAACCAATAGAAAG-CGAT-------AAGCTAAACGCACACCATTGCTGG------------------------NNNNNNNNNNNNNNNNNNNNNNNNNNNNNNNNNNNNNNNNNNNNNNNNNNNNNNNNNNN------------NNNNNNNNNNNNNNNNNNNNNNNNNNNNNNNNNNNNNNNNNNNNNNN---NNNNNNNN--NNNNNNNNNNNNNNNNNNNNNNNNN-NNNNNNNNNNNNGCA-ACCATTTACTTGAAGGTGTCAC------ACCC-------G-TACTGGACAATAATAAACTTCGAACGTTTT-AACCGCAACAATTTTAATATACGCTAGTGGAGCTGGAATTACCGCGGCTGCTGGCACCAGACTTGCCCTCCACTTGATCCTTATTAAAGGATTTATACT-TGATTCATTCCAATTACAGAACATAGTTAACTAGTTCTATATTGTTATTTTTCGTCACTACCTCC-----TTCAATACTCCGTTAAACGCAGTGTGCACTTTTCCCTTAGTAAGACGTTGTGATCTATTAGAAATTCATTCCAATGGTTATTGGGCTATCAATAGGCTCTCATAAAGTTCTTCGGCAAATGACTCGATCTTTAATGGTTGGGTGTATTGCTCGTTGAGTGTGAGATGTCCTGTA--ATAGCTGTCTGATAGATTTTAAAGGCCATATTACTACATTACCC-TGATA-CCCGATCAACAACGGTTAAATTTGGGATACCTTTGGGACCCGTCTTGAAACACGGACCAAGGAGTCTATCTAGTATGCAAGTAAATGGGGAAACCTATTTGCGTAAATAACTTGACTGATGGGATTACAAGTACTTTTCCATCCCGGGGCATTTTCATCAACATATGCAAATGTATATGGGAAATGTACCATGAGCATACTGGATATGACCCGAAAGATGGTGAACTATGCCTGATCAGGTTGAAGTCAGGGGAAACCCTGATGGAGGACCGAAGCAATTCTGACGTGCAAATCGCTGACTTTTCCTCTCATCGGAAATTATGGAATTCCTGATGATAATGAATTTGATGAACATAATTTGATCAAGAATTTTGAATCCAACAATAAGATATGGATTTCTGCTTTAGTTGTTGGAGAAATTTGTGATACTCCATCTCACTGGCGGGCAAAATATAAGCTGTCTGAATGGATGGAAAAGCATAATATCCCGGGTATAAGCGGAATTGATACACGAGCTCTTACTAAGAARATTCGAGAAAATGGAACAATTCTGGGAAAGATCATTCAGCAACCATCG------GGACCATTTCCAGGT---TTGGAATTCAAGGATCAAAATAAGCGTAACCTTGTGGATGAAGTTTCCATTAAAAAGCCTGTTACATACAACAAATCCGGGTCTCCACGCATTTGTGCAATCGATTGTGGACTGAAATTGAATCAAATTCGATGCTTCATCAATCGAGGAGCTCGTGTAGATATAGTTCCATGGGATTATGAACTTAATCCTGATGATTTTGATGGACTCTTCCTCAGCAATGGTCCCGGCGACCCTGTTATGTGTCAAAAAACTGTTAAGAACATTCAAAAGTTTTTGTCATCTAAAAACGTTAAGCCAATTTTTGGAATTTGTTTAGGACATCAACTTCTGTCCTCCGCAATTGGATGCAAAACTTACAAAATGAAATATGGTAATCGAGGCCACAACTTACCTGCAATTCATCATGGAACCAACCGTTGTTTTATGACTTCTCAGAATCATGGATTTGCTGTTGATGTCAGCAATATTGATCCTAATTGGGAACCCCTGTTCACAAATTTGAACGAT---------------------------AGCTCTATGAAAAGTGTAGGTGAAGTAATGGCAATTGGAAGAAAGTTTGAAGAAGCTTTTCAGAAGGCATTAAGAATGGTTGATGAAAATGTAAATGGCTTCGATCCGAAYCTTAAGCCTGTAAAAGATGAAGAATTGACAACACCAACCGATAAAAGAATGTTTGTTCTTGCAGCAGCTTTGAAAGCTGGGTACACAATCGATAAACTTTATGACTTGACTAAAATTGACCGTTGGTTTTTGTCAAAAATGAAGAACATCATCAACGTCACATTGGAGCTAGAAAAACTTAAT---TGCGTCGTCTCAGATTCACTTTTGTGGGAAGCGAAAAAGTTAGGATTTTCTGATAAGCAAATTGCGTCATTTATTAAG--GTCAGAACTGGCAGTGAGAAAACAGCGTCGCGAATATGGAATTTTGCCTTTTGTAAAACAAATTGATACTGTTGCTGGTGAGTGGCCTGCATCCACTAACTATTTGTATTTGACATACAATGCCAATACTCATGACATCGAATTCGAT---GATCAAATGGTGATGGTAATTGGAAGTGGTGTTTATCGAATTGGAAGCTCAGTCGAGTTTGATTGGTGTGCTGTTGGATGTCTAAGGGAACTGAGAAAGTTGGGCAAGAAGACAATCATGGTTAATTATAATCCAGAAACTGTATCAACCGATTATGACATGTGCGATAGATTGTACTTTGAAGAGATTTCTTTCATATTATTAGCCAAGAAAGTGGGAAAAAAGAAACTTTTGGGGCTTTAGGAATAATTTACGCTATACTAGCGATTGGTTTATTGGGGTTTATTGTTTGAGCTCATCATATATTTACAGTAGGAATAGATGTAGACACTCGAGCTTATTTCACTTCTGCTACTATAATTATTGCTGTGCCAACAGGAATTAAAATTTTCAGTTGACTAGCTACTCTTCATGGAACACAACTTAATTATTCTCCTTCTCTATTATGAGCTTTAGGGTTTGTATTTTTATTTACTGTAGGAGGATTAACTGGAGTAGTCTTAGCTAACTCTTCTTTAGATATTGTTCTTCATGATACTTATTATGTAGTAGCCCATTTTCATTATGTATTATCAATAGGAGCTGTATTTGCCATTATAGCAGGATTCGTTCATTGATACTCTTTATTTACAGGGTTAACAATAAATGAAGAATGATTAAAATCTCAATTTATAATTATATTCTTAGGAGTAAATTTAACATTTTTCCCTCAACACTTTCTTGGGCTTGCTGGGATGCCTCGTCGTTATTCAGATTACCCCGATGCATATACTTCATGAAATGTAGTATCAACTTTAGGATCAACTATTTCTTTATTTGGTATTTTATTTTTTCTTTTTATTATTTGAGAAAGT

**Propsilocerus akamusi** NNNNNNNNNNNNNNNNNNNNNNNNNNNNNNNNNNNNNNNNNNNNNNNNNNNNNNNNNNNNNNNNNNNNNNNNNNNNNNNNNNNNNNNNNNNNNNNNNNNNNNNNNNNNNNNNNNNNNNNNNNNNNNNNNNNNNNNNNNNNNNNNNNNNNNNNNNNNNNNNNNNNNNNNNNNNNNNNNNNNNNNNNNNNNNNNNNNNNNNNNNNNNNNNNNNNNNNNNNNNNNNNNNNNNNNNNNNNNNNNNNNNNNNNNNNNNNNNNNNNNNNNNNNNNNNNNNNNNNNNNNNNNNNNNNNNNNNNNNNNNNNNNNNNNNNNNNNNNNNNNNNNNNNNNNNNNNNNNNNNNNNNNNNNNNNNNNNNNNNNNNNNNNNNNNNNNNNNNNNNNNNNNNNNNNNNNNNNNNNNNNNNNNNNNNNNNNNNNNNNNNNNNNNNNNNNNNNNNNNNNNNNNNNNNNNNNNNNNNNNNNNNNNNNNNNNNNNNNNNNNNNNNNNNNNNNNNNNNNNNNNNNNNNNNNNNNNNNNNNNNNNNNNNNNNNNNNNNNNNNNNNNNNNNNNNNNNNNNNNNNNNNNNNNNNNNNNNNNNNNNNNNNNNNNNNNNNNNNNNNNNNNNNNNNNNNNNNNNNNNNNNNNNNNNNNNNNNNNNNNNNNNNNNNNNNNNNNNNNNNNNNNNNNNNNNNNNNNNNNNNNNNNNNNNNNNNNNNNNNNNNNNNNNNNNNNNNNNNNNNNNNNNNNNNNNNNNNNNNNNNNNNNNNNNNNNNNNNNNNNNNNNNNNNNNNNNNNNNNNNNNNNNNNNNNNNNNNNNNNNNNNNNNNNNNNNNNNNNNNNNNNNNNNNNNNNNNNNNNNNNNNNNNNNNNNNNNNNNNNNNNNNNNNNNNNNNNNNNNNNNNNNNNNNNNNNNNNNNNNNNNNNNNNNNNNNNNNNNNNNNNNNNNNNNNNNNNNNNNNNNNNNNNNNNNNNNNNNNNNNNNTTCAATACCTCGTTAAACTTAGTGTGCACTTTTCTCTTAGTAAGACGTTGTGATCCATTAGAAATGCATATCAATGATTGTTGGGCTATCAATAGGCCGTTATGAAGTTCTTCGGCAAATGGCGCGATCTTTCACGTTTGCGTGTATTGCTCGGTGAGCGTAGCGGTACCTGTA--ATAGCTGTCTGATGGATTTTAAAGGCCATATTACTGCATAACCCCTGATA-CTCGATCAACAACGGTTAAATTTGGGATACCTTTTGGACCCGTCTTGAAACACGGACCAAGGAGTCTATCTAGTATGCGAGTAAATGGGGAAACCTATTTGCATAAAAAACTTGACTGATGGGATTACGAGTGCTCTTCCATCCCGGGGCATTTTCATCAACATATGCAAATGTATATGGAAAATGTACCATGAGCATACTGGATATGACCCGAAAGATGGTGAACTATGCCTGATCAGGTTGAAGTCAGGGGAAACCCTGGTGGAGGACCGAAGCAGTTCTGACGTGCAAATCGCTGACTTACCCATTAATTGGAAACTATGGAATCCCGAGTGATGAAGAATTTGATGAGCATAAGCTTGCAAAGCACTTCGAGTCTAATAACAAAATTTGGGTATCGGCGTTGGTTGTTGGTGAAATTTGTGAAACTCCCTCGCACTGGCGTCAGAAATATAAACTTTCTGAATGGATGAAAAAACATAACATTCCTGGAATAAGTGGTATTGACACAAGAGCTCTCACCAAGAAGATCCGTGAGAATGGAACAGTTCTGGGAAAGATAATTCAACAGCCGTCT------GGACCGTTTCTTGGG---CTAGATTTTAAAGATCAAAATCAAAGGAATTTGGTCGATGAAGTTTCTACCAAAATGCCAATCACTTATAACGAGTCAGGATCGCCTCGCATTTGTGCTATTGATTGTGGCCTGAAGCTGAATCAAATTCGTTGCTTCATTCAACGTGGGGCTCGCGTTGATATTGTTCCTTGGGACCAAGAGCTTAATCCTGATTTATTCGATGGTCTCTTCTTGAGTAACGGTCCTGGCGATCCAGTTATGTGCAAAAATACAGTGAAGAACATTCAAAAGCTTTTGGCTTCACCGAAAGTAAAGCCAATTTTCGGCATCTGCCTGGGGCATCAGCTCTTATCAACAGCTATTGGATGTAAAACCTACAAAATGAAATATGGTAATCGTGGTCACAACCTRCCAGCTATTCATCATGGAACTAAGCGTTGTTTCATGACATCCCAAAATCATGGATTTGCTGTTGACGTCTCACGAATCGATGCRAATTGGGAGCCATTGTTCACAAACCTAAATGAT---------GTGAGCAAACACATAGGAAGTTCAATGAAATCTGTTGGTGAAGTCATGGCTATTGGAAGAAAATTCGAAGAAGCTTTTCAGAAAGCTCTGAGAATGGTTGATGAAACTGTAAATGGATTTGATCCAAATTTGAAATCAGTGAGAGATGAAGAATTGACAATGCCAACTGATACTAGAATGTTTGTACTCGCAGCAGCTCTAAAAGCTGGTTACACAATTGATCGTATCTACGATCTTACAAAAATCGACCGTTGGTTTTTGGCAAAAATGAAGCACATTATTGACATCACATTGGAGCTTGAAAAGTTAAAT---TGCGTTGTTTCAGATTCTCTTTTGTATCAAGCGAAGAAATATGGATTTTCTGACAAACAGATTGCATCTTATATTAAA--ATCAGAACTAGCTGTGAGAAAACAGCGACGTGAATGCGGATTTTTACCGTTTGTTAAACAAATTGACACCGTTGCTGGTGAATGGCCAGCGTCTACAAACTATTTATATCTAACGTACAATGCAACATCTCATGATGTTGAGTTTGAT---GATCAAATGACAATGGTTATTGGATCTGGAGTGTATCGCATTGGAAGTTCTGTAGAATTTGATTGGTGTGCTGTGGGATGCTTGCGAGAATTAAGAAAATTGGGAAAAAAGACAATTATGGTGAATTACAATCCAGAAACGGTGTCCACCGATTACGATATGTGYGATCGATTGTATTTTGAAGAGATCTCATTCATATTATTAGTCAAGAAAGAGGGAAAAAAGAAACTTTTGGGGCTTTAGGAATAATTTATGCAATACTTGCTATTGGTCTTTTAGGTTTTATTGTTTGAGCTCACCATATATTTACAGTAGGTATAGATGTTGATACCCGTGCTTATTTTACATCTGCTACTATAATTATTGCAGTACCAACAGGAATTAAAATTTTTAGCTGATTAGCTACTTTACATGGTACACAGTTTAATCTTTCTCCGGCTTTATTATGGGCTCTAGGATTTGTCTTTCTATTTACTGTTGGAGGGCTCACAGGAGTAGTCTTGGCAAACTCTTCAATTGATATCATATTACATGACACTTATTATGTTGTTGCTCATTTTCATTATGTTCTTTCTATGGGAGCAGTATTTGCCATTATAGCTGGATTTATTCATTGATACACTCTTTTAACAGGGTTAATTTTAAATGATGAATGATTAAAGGCCCAATTTATTGTTATATTTATTGGGGTTAATTTAACTTTTTTCCCACAACATTTTTTAGGGTTAGCTGGGATACCTCGTCGTTATTCAGATTATCCAGATGCTTATACTTCTTGGAATGTAGTTTCAACTATTGGATCCACAATTTCTCTTTTTGGAACTCTTTTTTTTCTATTTATTATTTGAGAAAGA

**Psectrocladius (Allopsectrocladius) sp.** ----------------GGCCATGCACCACT--ACCCTTAATTTC-A-TGAAAGCGCTGTCAA-GCTGTCATACTCTATCAAGTTCGG-ACCTGGTAAGTTTTCCCGTGTTGAGTCAAATTAAGCCGCAAATTCCAAAAC--TTGTGTGAACTTCCGTCAATTCCTTTAAGTTTCAACTTTGCAACCATACTTCCCCCGGAAACT--AG-CTTT-GGTTTCCCGGAAAGCTACTGAATGCACCATGAAA--AGTAGTGACATCCAATTGCTAGCTGTCATCGTTTACAGTTAGAACTAGGGCGGTATCTAATCGCCTTCGATCCTCTAACTTTCGTTCTTGATTAATGAAAACATCCTTGGCAAATGCTTTCGCTTTAGTTAGTCTTGCGACGGTCTAAGAATTTCACCTCTCGCGCCGCAATACTAATGCCCCCAACTGCTTCTATTAATCATTACCTCT-TGATCTG-TATCAAACCAATAGAAAG-CGATG------------CAACCCTTGCGAGCTACA-CCTA-TAAGACCGAGGTCTTTTTCCATTATTCCATGCAAAAATATTCAAGGCATT-AAAGCCTGCTTTGAGCACCTTAATTTGTTCAAGGTAAAAGTAAGCCGAACTAAATAGACACTTAGCCTAGTGAAAGGCATCGGTGCTATTCATTAA--GTGTTCAGTCATATAGTTCAAGTAA-TCGGAAATGATGGCA-GCCATTTACTTGATGGCGCCAC------ACCC-------G-TACTGAACAATAATCAACTTCGAACGTTTT-AACCGCAACAATTTTAATATACGCTAGTGGAGCTGGAATTACCGCGGCTGCTGGCACCAGACTTGCCCTCCACTTGATCCTCATTAAAGGATTTATACT-TGATTCATTCCAATTACAGAAC-TAAATAATTAGTTC---------------------------------TTCAATATT-----AAGTTCAATGTGCACTTCTCGCTTAGTAAGACGCTGTGATCCATTAGAAATTCATTCCAATGGTTATTTGGCTATCAATAGGTCGACGTAAAGTTCATTAGCAAATGACTCGATCCTTTATGGTTGGGTGTATTGTTTGGTGAGTGCGTCGTGTCCTGT-CTATAGCTGTCTGATGGATTTTAAAGGTCATATCATTGCATCACTCAGGATA-CCCGATCAACAGCGGTCAAATTTGGGATACCTTTTGGACCCGTCTTGAAACACGGACCAAGGAGTCTATCTAGTATGCGAGTAAATGAGGAAACTTATTTGCGTAAAAAACTTGACTGATGGGATTACGAGTGCTCCTCCATCCCGGGGTATTTTCATCCACATATGCAAATGTATATGGAAAATATACCATGAGCATACTGGATATGACCCGAAAGATGGTGAACTATGCTTGATCAGGTTGAAGTCAGGGGAAACCCTGATGGAGGACCGAAGCAATTCTGACGTGCAAATCGCTGACGTATCCGCTGATTGGYAACTACGGAATCCCGAGCGATGAGGAATTCGACGAAAACATGTTGATGAAGCACTTYGAGTCGAATAACAAGATCTGGGTGGCTGGATTAGTTGTTGGTGAATTGTGTACAACTCCGTCGCACTGGCGTCAGAAGTACAAACTAGCMGAGTGGATGAAGAAGCACAATGTGGTCGGCATCAGTGGAATTGACACTCGAGCGCTGACRAAGAAGATTCGCGAGGACGGCACGGTGCTGGGCAAAATCATCCAGCARTCGTCT------GGACCTTTTCCGGGG---TTGGAGTTCAAGGACCAGAACCAGAGAAACCTCGTCGATGAAGTCTCCATCAAGAAACCGATAACGTACAATGCCAAGGGATCCCCAAGGATCTGCGTGRTGGACTGCGGCCTGAAGTTGAATCAGCTWCGTTGYTTCATCAAGCGGGGAGCTCGGGTCGATGTCGTGCCATGGGATCACCCACTCGATCCAAAAGACTTTGACGCGCTCTTCTTGTCAAACGGTCCTGGTGACCCCGTCATGTGCCAGAAAACTGTGAATAACATCAAACAAGTCCTTGCMGCACCTCAGGTCAAACCTGTGTTCGGTATCTGCTTGGGTCACCAGCTCTTGTCAACTGCYGTAGGCTGTAAAACTTACAARATGAAGTACGGAAACCGTGGCCACAACTTGCCGGCGCTTCACCATGGCACCAACCGCTGYTTCATGACATCCCAAAATCACGGATTYGCTGTTGACGTGAAGACTCTCGGCAAGGACTGGGAGCCACTG------------------TTCACGAGAGTCAGCAAAAATATCGGCAGCTCTATGAAGTCTGTGGGTGAAGTGATGGCGATTGGTAGGAAGTTCGAAGAAGCTTTTCAAAAAGCTCTTCGAATGGTTGACGAAAACGTTAATGGATTCGATCCCAACCTGAAGCCTGTGGTTGATGAAGAATTGAAGTCACCGACTGATAAGAGAATGTTTGTGTTGGCTGCTGCACTCAAAGCGGGTTACACCGTTGACAGGCTCTACGATTTGACGAAGATCGACCGTTGGTTCCTCGTAAAAATGAAAAATATTATCGATATTACTTTGGATCTGGAGAAGCTCAAT---TGCGTAATTCCAGATGAACTGCTGAGGCTGTCAAAGAAATTGGGATTTTCTGATAAACAAATTGCGACTTACATCAAG--CTCCGAGTTGGCGGTGCGCAAACAGAGAAGAGAAGCTGGAATACTTCCATTTGTTAAGCAAATCGACACTGTTGCTGGTGAATGGCCTGCATCGACAAACTACCTTTACCTAACTTATAATGCAAGCGAACATGACATTGCGTTCAAT---GACCAGATGGTGATGGTGATTGGATCTGGAGTTTATCGAATCGGCAGCTCAGTGGAATTCGATTGGTGCGCTGTTGGTTGTTTGAGGGAATTGAGAAACCTTGGAAAGAAAACGATTATGGTCAACTACAACCCCGAAACTGTTTCCACCGACTACGACATGTGCGATCGACTT--------------------CATATTATTAGTCAAGAAAGAGGGAAAAAGGAAACTTTCGGAGCTTTAGGAATAATTTATGCTATATTAGCTATTGGTCTATTAGGTTTTGTTGTATGAGCTCATCATATATTTACTGTAGGTATAGACGTTGACACTCGAGCTTATTTTACTTCTGCTACTATAATTATTGCTGTTCCTACAGGAATTAAAATTTTTAGTTGGTTAGCAACTCTTCACGGAACACAACTTAATAATTCTCCATCTTTACTTTGAGCTTTAGGGTTTGTATTTTTATTTACTGTTGGAGGATTAACCGGAGTAGTTTTAGCTAATTCTTCTATTGATATTGTACTTCACGATACTTATTATGTTGTAGCCCATTTCCATTATGTACTTTCTATAGGAGCTGTATTTGCTATTATGGCAGGATTTGTTCATTGATACCCTTTATTTACTGGATTAGCTTTAAATGAAAAATGATTAAAGTCTCAATTTGCAATCATATTTTTAGGAGTAAATCTTACATTCTTCCCTCAACATTTCTTAGGATTAGCCGGGATACCTCGACGATACTCAGACTACCCAGATGCTTATACAACATGAAATATTGTATCAACAGTAGGTTCGACCATTTCATTATTTGGAATTTTATTTTTCTTATTTATCATTTGAGAAAGT

**Psectrocladius (s. str.) sp.** --------------------------------------------------------TGTCAA-GCTGTCATACCCTATTAAGTTCGG-ACNTGGTAAGTTTTCCCGTGTTGAGACAAATTAAGCCGCAAATTCCAAAAC--GGGGGTGAACTTCCGTCAATTYCTTTAAGTTTCCACTTTGCAACCATACTTCCCCCGGAAACT--AR-CTTT-ANTTTCNNGGAAAGCTACGGAATGCACCATGAAA--AGTANNGACATCCACTTGCTANCTGTCATCGTTTACAGTTAGAACTAGGGCGGTATCTAATCGCCTTCGATCCTCTAACTTTCGTTCTTGATTAATGAAAACATCCTTGGNAAATGCTTTCGCTTTAGTTGGTCTTGCGACGGTCTAAGAATTTCTCCTCTCGCGCCGCAATACTAATGCCCCCAACTGCTTCTATTAATCATTACCTCT-TGATCTG-TATCAAACCAATAGAAAG-CGATN----NNNNNNNNNNNNNNNNNNNNNNTGCA-CGTA-TAAGACCGAGGTATTTTTCCATTATTCCATGCAAAAATATTCAAGGCATT-ANAGCCTGCTTTGAGCACCTTAATTTGTTCAAGGTAAAAGTAAGCCGAANTAAATAGACAATTAGCCTAGTTAAAGGCATTGGTGNTATTCATTAA--GTGTTCAGTCATATAGTTCAAGTAA-TCGGAAGAGATGGCA-GCCATTTACTTGATGGCGCCAC------ACCC-------G-TACTGAACAATAATCAACTTCGAACGTTTT-AACCGCAACAATTTTAATATACGCTAGTGGAGCTGGAATTACCGCGGCTGCTGGCACCAGACTTGCCCTCCACTTGATCCTCATTAAAGAATTTATACT-TGATTCATTCCAATTACAGAAC-TAAATAATTAGTTCTATATTGTTATTTTTCGTCACTACCTCC-----TTCAATATT-----AAGCTCAATGTGCACTTCTCGCTTAGTAAGACGCTGTGATCCATTAGAAATTCATTCCAATGGTTATTTGGCTATCAATAGGTCGGCGTAAAGTTCATTAGCAAATGACTCGATCTTTTATGGTTGGGTGTATTGTTTGGTGAGTGCGTCGTGTCCTGT-CTATAGCTGTCTGATGGATTTTAAAGGTCATATCATTGTATCACTCAGGATA-CCCGATCAACAGCGGTTAAATTTGGGATACCTTTTGGACCCGTCTTGAAACACGGACCAAGGAGTCTATCTAGTATGCGAGTAAATGAGGAAACTTATTTGCATAAAAAACTTGACTGATGGGATTACGAGTGCTCCTCCATCCCGGGGTATTTTCATCAACATATGCAAATGTATATGGAAAATATACCATGAGCATACTGGATATGACCCGAAAGATGGTGAACTATGCTTGATCAGGTTGAAGTCAGGGGAAACCCTGATGGAGGACCGAAGCAATTCTGACGTGCAAATCG---------CCATTGATTGGAAATTATGGAATTCCGAGCGATGAAGAATTCGATGAAAACCAATTGATGAAACATTTTGAGTCGAACAACAAGATTTGGGTTTCTGGACTAGTTGTTGGTGAATTATGTGAGACTCCGTCCCATTGGCGTTTGAAGTACAAATTGGCTGAGTGGATGAAGAAGCATAATGTAGTCGGCATCAGTGGAATTGACACGCGAGCTTTGACCAAGAAAATTCGCGAGAATGGAACGGTTTTGGGAAAAATCATTCAGAAATCCTGT------GGACCTTTCCCTGGA---CTAGAGTTCAAAGACCAGAATCAGAGAAACCTCGTCGATGAAGTCTCAATTAAAAAACCGGTTACTTACAACGTTAAGGGATCACCGAGAATTTGCATGATTGACTGTGGAATGAAGTTRAATCAAGTTCGYTGTTTCATCAAGCGTGGTGCTCGAGTAAGAGTTGTAAAACATAAAAAA------------------------------------------------------------------------------------------------------------------------------------------------------------------------------------------------------------------------------------------------------------------------------------------------------------------------------------------------AGAGTCAGCAAACACATCGGCAGCTCCATGAAAAGTGTCGGCGAAGTRATGGCGATCGGTAGAAAGTTTGAAGAAGCTTTTCAGAAAGCACTGAGGATGGTTGACGAGAATGTGACCGGTTTTGATCCAAATCTGAAACCAGTAGTTGAAGAGGAATTAACRACACCGACAGATAAAAGAATGTTTGTTCTGGCAGCTGCATTGAAAGCTGGTTACACCGTTGATCGTCTTTATGATTTGACAAAAATCGATCGTTGGTTTTTAGAAAAGATGAAAAACATCATCAAACTGACACTGGAGCTGGAAAATTTGAAT---TGCGCCATCGACGACGATCTTTTGAGAGAAGCAAAAATGCTCGGTTTCTCTGACAAGCAAATAGCTTCRTTCACCAAA--ATCAGAACTGGCGGTGMGAAAACAAAGACGAGARGGYGGRATTTTACCGTTTGTCAAGCAGATCGATACTGTCGCTGGTGAGTGGCCTGCATCGACTAACTATCTCTATCTGACATACAATGCTTCRACCCACGATATCGATTTCAAC---GAGCAGATGGTTATGGTCATTGGCAGCGGTGTTTATCGAATTGGGAGTTCTGTGGAGTTTGATTGGTGTGCCGTTGGCTGCTTGAGAGAGCTGAGAAATCTCGGCAAGAAAACTATYATGGTGAATTACAACCCAGAAACTGTTTCCACCGACTACGACATGTGCGACCGTCTTTACTTTGAGGAAATATCTTTCATATTATTAGCCAAGAAAGAGGAAAGAAGGAAACTTTTGGAGCTTTAGGAATAATTTATGCAATGTTAGCTATTGGCCTATTAGGGTTCGTTGTATGAGCTCATCATATATTTACAGTTGGTATAGATGTAGACACTCGGGCTTATTTTACTTCAGCAACTATAATTATTGCCGTTCCCACAGGAATTAAAATTTTCAGTTGATTAGCAACATTACATGGAACCCAATTAAATAACTCGCCTTCTTTACTATGAGCATTAGGGTTTGTGTTTTTATTCACCGTAGGAGGTCTAACAGGAGTAGTATTAGCTAACTCCTCTATTGATATTGTTCTACACGATACTTATTATGTAGTAGCTCATTTCCATTATGTACTATCGATAGGAGCTGTCTTTGCTATTATAGCAGGCTTTGTTCACTGATACCCTCTATTTACAGGACTAACAATAAATGAAAGATGATTGAAATCTCAATTTGCAATTATATTCTTAGGGGTAAATTTAACCTTTTTCCCTCAACACTTCCTAGGTTTAGCAGGAATGCCTCGTCGTTATTCTGATTACCCAGATGCTTACACAACATGAAATATTGTTTCAACAGTAGGGTCCACTATCTCATTATTTGGGATTTTATTTTTTATCTTTATTATTTGAGAAAGA

**Pseudosmittia sp.** NNNNNNNNNNNNNNNNNNNNNNNNNNNNNNNNNNNNNNNNNNNNNNNNNNNNNNNNNNNNNNNNNNNNNNNNNNNNNNNNNNNNNNNNNNNNNNNNNNNNNNNNNNNNNNNNNNNNNNNNNNNNNNNNNNNNNNNNNNNNNNNNNNNNNNNNNNNNNNNNNNNNNNNNNNNNNNNNNNNNNNNNNNNNNNNNNNNNNNNNNNNNNNNNNNNNNNNNNNNNNNNNNNNNNNNNNNNNNNNNNNNNNNNNNNNNNNNNNNNNNNNNNNNNNNNNNNNNNNNNNNNNNNNNNNNNNNNNNNNNNNNNNNNNNNNNNNNNNNNNNNNNNNNNNNNNNNNNNNNNNNNNNNNNNNNNNNNNNNNNNNNNNNNNNNNNNNNNNNNNNNNNNNNNNNNNNNNNNNNNNNNNNNNNNNNNNNNNNNNNNNNNNNNNNNNNNNNNNNNNNNNNNNNNNNNNNNNNNNNNNNNNNNNNNNNNNNNNNNNNNNNNNNNNNNNNNNNNNNNNNNNNNNNNNNNNNNNNNNNNNNNNNNNNNNNNNNNNNNNNNNNNNNNNNNNNNNNNNNNNNNNNNNNNNNNNNNNNNNNNNNNNNNNNNNNNNNNNNNNNNNNNNNNNNNNNNNNNNNNNNNNNNNNNNNNNNNNNNNNNNNNNNNNNNNNNNNNNNNNNNNNNNNNNNNNNNNNNNNNNNNNNNNNNNNNNNNNNNNNNNNNNNNNNNNNNNNNNNNNNNNNNNNNNNNNNNNNNNNNNNNNNNNNNNNNNNNNNNNNNNNNNNNNNNNNNNNNNNNNNNNNNNNNNNNNNNNNNNNNNNNNNNNNNNNNNNNNNNNNNNNNNNNNNNNNNNNNNNNNNNNNNNNNNNNNNNNNNNNNNNNNNNNNNNNNNNNNNNNNNNNNNNNNNNNNNNNNNNNNNNNNNNNNNNNNNNNNNNNNNNNNNNNNNNNNNNNNNNNNNNNNNNNNNNNNNNNNNNNNNNNTTCAATATTCCGTTAAAAGCAATGTGCACTTCTCCCTTAGTAAGACGCTGTGATCCATTAGAAATTCATTCCAATGGTTATTTGGCTATCAATAGGCCGGTATGAAGTTTATCGACAAATAACTCGATCTTTAATGGCTGGGTATATTGTTTGGTAAGCATGCCGTCACCTGT---ATAGCTGTCTGATGGGTTTTTAAGGTCATATTATTGCATCACTCAGAATAACCCGATCAACAGCGGTTAAATTTGGGATACCTTTTGGACCCGTCTTGAAACACGGACCAAGGAGTCTATCTAGTATGCAAGTAAATGAGGAAACTTATTTGCGGAAAAAACTTGACTGATGGGACTACGAGTGCTCCTCCATCCCGGGGTATTTTCATCAACATACGAAAATTTGTATGGAAAATATACCATGAGCATACTGGATATGACCCGAAAGATGGTGAACTAAACGCGAACAGGTTGAAGTCAGGGGAAACCCTGATGGAGGACCGAAGCAGTTCTGACGTGCAAATCGTTGACATATCCCCTGATCGGCAATTACGGAGTCCCGAGCGACGAAGAATTCGACGAGAATAAGCTGATTAAGCATTTTGAGTCGGAGAACAAGATTTGGGTGTCCGGCCTGGTGGTCGGTGAACTGTGCGAGACTCCGTCTCACTGGCGTCAGAAATACAAACTTGCCGAATGGATGAAGAAGCATGGAGTGGTCGGAATCAGTGGCATCGACACCCGCGCTTTGACCAAGAAAATCCGGGAGAACGGAACTGTCTTGGGCAAAATCATTCATCAGTCGGCG------GGCCCTTTTCCCGGC---TTGGARTTCAAGGATCAGAACATGCGGAATTTGGTCGACGAAGTCTCGATTAAGAAGCCGCGCGTGTTCAACGAGAAAGGCTCTCCGAGRATCTGCGCCATCGACTGCGGCTTGAAGTTRAATCAAGTCCGCTGCTTCATCAATCGAGGCGCACGTGTCGAGTTGGTTCCTTGGGATCATCCTCTRAACCCGAAGGACTTCGATGGACTYTTCATCAGCAACGGTCCTGGCGATCCCGTTATGTGCGAGAAGACTGTGAAGAACATCAAAAGCTTCTTAGCTTCGCCGAACGTTCGACCGGTTTTCGGCATTTGCTTGGGGTTCGTCACT------------------------------------------------------------------------------------------------------------------------------------------------------------------------------------------------------------------------AAGAGTGTCGGTGAAGTGATGGCGATCGGCCGAAAGTTCGAGGAAGCTTTCCAGAAAGCTCTGCGTATGGTCGACGAGAACGTGACCGGCTTCGATCCGAACTTGAAGCGCGTTTGCGACGAAGAACTGGAAATTCCCACTGACAAGCGGATTTTCGTTCTCGCGGCTGCTTTGAAAGCMGGCTACACTGTCGATCGTCTCTACGACTTGACGAAAATCGACCGCTGGTTCCTCGAGAAGATGAAGAACATCAACGCCATCACGCTCGAGCTCGAAGAGCTGAAC---TGCGCTGTCTCGAATGAGCTRTTAAAGCGAGCGAAAGCATTCGGCTTCTCCGACAAGCAAATCTCGAAGCTGACGAAG--TTCCGAGCTGGAAGTACGCAAGCAGCGCCGCGAGAGCGGAATCATTCCGTTTGTCAAGCAAATCGACACAGTCGCTGGCGAGTGGCCGGCCTCCACCAACTATCTCTATCTCACTTACAACGCGAGCGCACACGACATTGAGTTCAAC---GAGCAGATGACGATGGTGATCGGATCGGGTGTGTACAGAATCGGCAGCTCGGTGGAGTTCGATTGGTGCGCCGTTGGCTGCTTG------------------------------------------------------------------------------------------------------------CGCATATTATTAGACAAGAAAGTGGTAAAAAGGAAACATTTGGTGCTTTAGGAATAATTTATGCTATATTATCAATTGGTTTATTAGGTTTTATTGTATGAGCCCATCATATATTTACAGTTGGAATAGATGTAGATACCCGAGCCTACTTTACTTCTGCAACTATAATTATTGCTGTTCCTACAGGTATTAAAATTTTTAGTTGATTAGCTACAATTCATGGAACCCCTTTAAATAATTCCCCTTCATTATTGTGAGCTCTTGGATTTGTTTTCTTATTTACAGTAGGAGGATTAACGGGGGTTATTCTTGCTAACTCTTCAATTGACATTGTATTACACGATACATACTATGTAGTAGCTCATTTTCATTATGTTCTTTCTATGGGGGCTGTATTTGCTATTATAGCAGGATTTGTACACTGATATACTTTATTTACAGGACTAACAATAAATGAAAAATGACTTAAATCCCAATTTAGAATCATATTCTTAGGAGTAAATTTAACGTTTTTCCCACAACATTTTTTAGGATTAGCTGGTATACCACGACGATACTCTGATTATCCTGATGCTTACACCTCATGAAATATTATATCAACTGTTGGTTCTACTATTTCTTTAATCGGAATTTTATTTTTTATTTTTATTATTTGAGAAAGA

**Rheocricotopus sp.** ---------------------------------------------A----------------------------CTAAAAAGTTCGGGACCTGGTAAGTTTTCCCGTGTTGAGTCAAATTAAGCCGCAAAATCCAAATC--CGGTATGATCTTCCGTCAATTCCTTTAAGTTTCAACTTTGCAACCATACTTCCCCCGGAAACT--AG-CTTT-GGTTTCCCGGAAAGCTACTGAATGCACCATGAAA--AGTAGTGACATCCAATTGCTAGCTGTCATCGTTTACAGTTAGAACTAGGGCGGTATCTAATCGCCTTCGATCCTCTAACTTTCGTTCTTGATTAATGAAAACATCCTTGGCAAATGCTTTCGCTTTAGTTAGTCTTGCAACGGTCTAAGAATTTCACCTCTCGCGCTGCAATACCAATGCCCCCAACTGCTTCTATTAATCATTACCTCT-TGATCTG-TATCAAACCAATAGAAAA-CGGTA-----------CAACCCTTGCGAGCTGATA-CCTA-TAAGACCGAGGTCTTTTTCCATTATTCCATGCAAAAATATTCAAGGCATT-AGAGCCTGCTTTGAGCACCTTAATTTGTTCAAGGTAAAAGTAAGCCGAACTAAATAGACACTTAGCCTAGCTAAAGGCATCGGTGCTATTCATTAA--GTGTTCAGTCATATAGTTCAAGTAA-TCGGAAATGATGACA-GCCATTTACTTGATGGCGTCAC------ACCC-------G-TACTGAACAATAATCAACTTCGAACGTTTT-AACCGCAACAATTTTAATATACGCTAGTGGAGCTGGAATTACCGCGGCTGCTGGCACCAGACTTGCCCTCCACTTGATCCTCATTGAAGGATTTATACT-CGATTCATTCCAATTACAGAAC-TAAAT------------------------------------------TTCAATACTTTGGCAACAACAGTGTGCACTTCTCGCTTAGTAAGACGTTGTGATCCATTAGAAATTTATTCCAATAGCTATGAGGTTATCAATAGGCTGGTGTAAAGTTCATCAGTAAATGTCTAGATCTTTAATGGTCGAGTGTATTATATGTTGAGTGCATCATG-TCTGC-CTATAACTGTCTGATGGATTTTAAAGATCATAACATTGCATCACCCAGGTTAATCCGATCAACAACGGTTAAATTTGGGATACCTTTTGGACCCGTCTTGAAACACGGACCAAGGAGTCTATCTATTGCGCGAGTAAATGGGGAAACCTATTTGCGGAAAAAACTTGACTGATGGGATTACGAGT-CTCCTCCATCCCGGGGTATTTTCATCAACACATGCAAATGTATGTGGAAAATATACCATGAGCGTAGTGGATATGACCCGAAAGATGGTGAACTATGCCTGATCAGGTTGAAGTCAGGGGAAACCCTGATGGAGGACCGAAGCAATTCTGACGTGCAAATCGCTGACCTATCCGCTNATCGGAAATTACGGCGTTCCGGGCGATGACGAGTTCGACGAGCATCAAATCGTAAAGAATTTCGAATCCAACAACAAAATTTGGGTTTCGGCTTTGGTCGTCGGTGAAGTTTGCGAGACTCCATCGCATTGGCGAATGCGATACAAGTTGGCCGAATGGATGAAGAAGCACAACGTAGTTGGGATTAGCGGAATCGACACTCGAGCTTTGACGAAGAAGATTCGAGAAGACGGAACTGTATTGGGAAAAATCATCCAGAAGTCTTCG------GGCCCGTTTGTTGGA---TTGGAATTCAAAGATCAGAACCTGAGGAACTTGGTCGATGAAGTTTCAACCAAGAAGCCAGTCACGTACAATCCGAAGGGATCCCCAAGGATCTGCGCTGTCGATTGCGGACTCAAACTCAACCAGATTCGATGCTTCTTGCAACGAGGAGCTCGAGTTGATGTTGTGTCTTGGGACCACCCACTGAATCCTGATGATTTCGATGGACTTTTCCTCTCCAATGGTCCCGGCGATCCCGTCATGTGTCAGAAAACAGTCGCAAACATCCAGAAAGTCCTTGGATCGAAGCAAGTCAARCCAGTTTTCGGAATTTGCTTGGGTCACCAGCTGCTGTCAACGGCTGTCGGCTGTAAAACTTACAAACTGAAATATGGAAATCGAGGACACAACCTGCCAGCTCTACATCATGGCACCAAACGCTGCTTCATGACTTCACAAAATCACGGATTTGCTGTTGATGTGAAGACGCTGAACAAAGACTGG---------------------------TTCATTCGTGTCAGYAAGCACATCGGAAGTTCGATGAAAAGCGTTGGAGAGGTCATGGCCATAGGTCGCAAGTTTGAAGAAGCTTTTCAGAAAGCTTTGAGGATGGTCGACGAGAACGTAATTGGCTTCGATCCAAACTTGAAGCCTGTGAACGACGCTGAGCTGGTGGCTCCAACTGACAAACGAATGTTCGTCCTCGCTGCAGCTTTAAAAGCTGGCTACAGTGTCGATAGGCTGTATGACTTGACTAAAATCGATAAATGGTTTTTGGAGAAGATGAAGAACATCATCACGTTGACTGTGGAGCTAGAAAAGCTCAAC---TGCGTGATTAGTGATGACATGCTGAGGGAAGCGAAGATTCTGGGATTCTCGGATAAGCAGATCGCGACGCATATCAAGGTTTCGGAGCTTGCAGTGAGAAAACAGCGTCGTGAGAGCAAAATTCTTCCTTTCGTCAAGCAAATCGACACTGTCGCTGGGGAATGGCCGGCATCCACCAACTACCTCTACTTGACCTACAACGCAAGTGATCACGACGTCGAATTCAGT---GAGGAAATGGTGATGGTCATCGGTTCGGGTGTTTATCGCATTGGAAGCTCAGTAGAATTTGATTGGTGTGCGGTTGGATGTCTCAGGGAACTGAGGAACTTGGGAAAGAAGACAATCATGGTGAACTACAACCCGGAAACGGTGTCCACTGACTACGACATGTGCGACCGACTTTACTTCGAAGAAATTTCCTTCATATTATTAGTCAAGAAAGAGGAAAAAAGGAAACTTTCGGATCTTTAGGAATAATTTATGCTATATTAGCAATTGGTTTATTAGGATTTGTTGTATGAGCACATCATATATTCACTGTTGGAATAGACGTTGATACTCGAGCATACTTTACTTCAGCTACTATAATTATTGCTGTACCAACAGGTATTAAAATTTTCAGTTGATTAGCAACCTTACATGGAGCCCAATTAAATAACTCCCCATCTTTATTATGGGCTTTAGGGTTTGTATTTTTATTTACAGTTGGGGGACTAACTGGAGTTATTTTAGCTAACTCCTCTATTGATATTGTACTACATGACACATATTATGTTGTTGCTCATTTCCATTATGTATTATCTATAGGAGCAGTATTTGCTATTATAGCAGGATTTGTTCACTGATACCCTTTATTCACAGGATTAACTATAAATGAAAAATGATTAAAGTCACAATTTTCAATTATATTTTTAGGAGTAAATTTAACATTTTTCCCACAACATTTCTTAGGATTAGCTGGAATACCTCGACGATATTCAGACTATCCCGATGCATATACTTCATGAAATATTGTTTCAACAGTAGGATCTACAATTTCTTTATTTGGTATTTTATTTTTCATTTTTATTATTTGAGAAAGT

**Stictocladius pictus** -------------------------------------------------------------------------CCTATAAAGTTCTG-ACCTGGTAAGTTGTCCCGTGTTGAGTCAAATTAAGCCGCAAACTCCAAAAC--TTGTGTGCCCTTCCGTCAATTCCTTTAAGTTTCAACTTTGCAACCATACTTCCCCCGGAAACT--AA-CTTT-AGTTTCCCGGAAAGCTACTGAATGCACCATGAAT--AGTAGTGACATCCAATTGCTAGTCGTCATCGTTTACTGTTTAAACTAGGACGGTATCTAATCGTCTTCGATCCTTAAACTTTCGTTCTTGATTAATGAAAACATCCTTGACAAATGCCTTCGCTTTAGTTAGTCGTACGACGGTCTAAGAATTTCACCTCTCGCGCCGTAATACTAATGCCCCCGACTGCTTCTATTAATCATTACCTCT-TGATCTG-TATCAAACCAATAGAAAG-CGGCA----------TGAACACTTGCGTGCACACA-CCTA-TTAGACCGAGGTCTTTTTCCATTATTCCATGCAAAAATATTCAAGGCATATAGAGCCTGCTTTGAGCACCTTAATTTGTTCAAGGTAATAGTAAGCCGAACTAAACAGGCACCTAGCCTAATGAAAGGCATCAGTGCTGTTCATTAA--GTGTTCAGTCATATAGTCCAAGTAA-CCGGAAATGATGGCA-ACCATTTACTTGATGGTGCCAC------ACCC-------G-AACTGGACAAAAATCAACTTCGAACGTTTT-AACCGCAACAATTTTAACATACGCTAGTGGAGTCGGAATTACCGCGGCTGCTGGCACCGAACTTGCCCTCCACTGGATCCTCATTGAGGAATTTATATT-CGATTCATTCCAATTACAGAACATAGTTAACTAGTTCTATATTGTTATTTTTCGTCACTACCTCC-----TTCAATATTCCCTTAACAGCAATGTGCACTTTTTTCTTAGTAAGACGCTGTGATCCATTAGAAATTCATTCCAATGGTTATTGGGCTATCAATAGGCCAACATGAAGTTCAATGGCAAATGGTATGATCTTTTATGGTTGCACGTATTGCCCGTTGAGCATGTTGGTACCTGTA--ATAGCTGTCTGATGGATTTTAAAGGTCATATTACTGCATTACCCATGATA-CCCGATCAACAGCGGTTAAATTTGTGATACCTTTGGGACCCGTCTTGAAACACGGACCAAGGAGTCTATCTAGTATGCAAGTAAATGAGTAAACTTATTTGCGTAAAAAACTTGACTTATGGGATTACGAGTGCTCCTCCATCCCGGGGTATTTTTGTCAACATATGCAAATGTGTATGGAAAATATACCATGAGCATACTGGATGCGACCCGAAAGATGGTGAACTATGCCTGTTCAGGTTGAAGTCAGGGGAAACCCTGATGGAGGACCGAAGCAATTCTGACGTGCAAATCGTTGACGTTTCCTCTCATCGGAAACTATGGAGTCCCTTCTGACACAGAATTGGACGAATTTGGTTTGATAAAAAATTTTGAGTCGAATGGAAAAATCTGGATTTCTGGGTTAGTTGTCGGCGAATTGTGCGAAACCCCATCCCACTGGCGTCAGAAGTATAAGCTATCGGAATGGATGAAAAAGCACAATATTCCCGGCATCAGCGGCATTGACACGCGCAGTTTGACGAAGAAAATTCGGGAGGATGGTACAGTTCTGGGCAAAATCATTCAGCAGTCTTCT------GGGCCTTTTACCGGT---TTACAGTTTAGGGATCAAAATGAAAGGAACTTGGTCGACGAGGTATCCCTCAAGAAAGCTGTTGTTTATAACCCGACTGGCTCTCCTAAAATCTGTGCAGTGGACTGTGGATTGAAATTGAACCAAATTCGATGCTTCGTTAAAAGAGGAGCTCGAGTTGATGTTGTGCCATGGAATCAAGAACTGAAAGCTAAAGACTTTGATGGACTGTTTCTCTCTAATGGTCCTGGGGATCCCGTAATGTGCCAAGCTACTGTCAAGAACATTCAAAATTTTTTAAACTCTTCGAAAACAAAGCCCATTTTTGGCATATGTCTGGGGCATCAGTTGCTAGCAAGTGCTATTGGATGCAAAACTTACAAGATGAAGTACGGCAATCGTGGACATAATTTACCTGCTATCCACCACGGATCCAATCGCTGCTTCATGACATCCCAAAATCATGGATTTGCGGTTGATGTTACAAATATCAATGACGATTGGGAACCCCTTTTTACAAACTTGAATGATNNNNNNNNNNNNNNNNNNNNNNNNNNNNNNNNNNNNNNNNNNNNNNNNNNNNNNNNNNNNNNNNNNNNNNNNNNNNNNNNNNNNNNNNNNNNNNNNNNNNNNNNNNNNNNNNNNNNNNNNNNNNNNNNNNNNNNNNNNNNNNNNNNNNNNNNNNNNNNNNNNNNNNNNNNNNNNNNNNNNNNNNNNNNNNNNNNNNNNNNNNNNNNNNNNNNNNNNNNNNNNNNNNNNNNNNNNNNNNNNNNNNNNNNNNNNNNNNNNNNNNNNNNNNNNNNNNNNNNNNNNNNNNNNNNNNNNNNNNNNNNNNNNNNNNNNNNNNNNNNNNNNNNNNNNNNNNNNNNNNNNNNNNNNNNNNNNNNNNNNNNNNNNNNNNNNNNNNNNNNNNNNNNNNNNNNNNNNNNNNNNNNNNNNNNNNNNNNNNNNNNNNNNNNNNNNNNNNNNNNNNNNNNNNNNNNNNNNNNNNNNNNNNNNNNNNNNNNNNNNNNNNNNNNNNNNNNNNNNNNNNNNNNNNNNNNNNNNNNNNNNNNNNNNNNNNNNNNNNNNNNNNNNNNNNNNNNNNNNNNNNNNNNNNNNNNNNNNNNNNNNNNNNNNNNNNNNNNNNNNNNNNNNNNNNNNNNNNNNNNNNNNNNNNNNNNNNNNNNNNNNNNNNNNNNNNNNNNNNNNNNNNNNNNNNNNNNNNNNNNNNNNNNNNNNNNNNNNNNNNNNNNNNNNNNNNNNNNNNNNNNNNNNNNNNNNNNNNNNNNNNNNNNNNNCATATTATTGCCCAAGAAAGAGGAAAAAAAGAAACTTTTGGAACTTTAGGTATAATTTATGCTATACTAGCTATTGGTCTCTTAGGGTTTATTGTATGAGCTCATCACATATTTACAGTAGGGATAGACGTTGACACCCGGGCTTACTTTACCTCAGCCACAATAATTATTGCCGTCCCCACAGGTATCAAAATTTTTAGATGATTGGCTACTTTACATGGAACCCAATTAAATTATTCCCCCTCTCTACTATGAGCCTTAGGCTTCGTGTTTTTGTTCACAGTGGGGGGATTAACTGGAGTAATTTTAGCAAACTCTTCATTAGATATCGTTCTCCATGATACTTATTATGTTGTAGCCCACTTTCACTACGTTTTATCTATAGGAGCGGTATTTGCTATTATGGCAGGTTTTATTCACTGGTACCCCCTATTTACTGGCCTTATTATAAATAAAAAATGATTAAAATCTCAATTTATAGTAATATTCTTAGGAGTAAACCTAACCTTTTTCCCCCAACATTTCTTAGGATTAGCCGGGATACCCCGACGTTACTCCGACTACCCTGACGCTTACACCTCCTGGAATATTATCTCAACTATAGGGTCAACCATTTCTTTTTTTGGAATCCTATTTTTTTTACTTATTTTATGAGAAAGC

**Stictocladius sp. 1** ----------------------------CT--ACCCTTTATTTC-A-AGCAAGCGCTATCAA-GCTGGCTAACCCTATAAAGTTCTG-ACCTGGTAAGTTGTCCCGTGTTGAGTCAAATTAAGCCGCAAGCTCCATCGC-CGGTGATAGTCTTCCGTCAATTCCTTTAAGTTTCAACTTTGCAACCATACTTCCCCCGGAAACT--AA-CTTT-GGTTTCCCGGA-AGCTACTGAATGCACCATGAAT--AGTAGTGACATCCAATTGCTAGTTGTCATCGTTTACTGTTTAAACTAGGACGGTATCTAATCGTCTTCGATCCTTAAACTTTCGTTCTTGATTAATGAAAACATCCTTGACAAATGCCTTCGCTTTAGTTAGTCGTGCGACGGTCTAAGAATTTCACCTCTCGCGCCGCAATACTAATGTCCCCGACTGCTTCTATTAATCATTACCTCT-TGATCTG-TATCAAACCAATAGAAAG-CGAAT--------------GCCCGGTGAAGAGCAT-CACA-TAAGACCGAGGTCTTTTTCCATTATTCCATGCAAAAATATTCAAGGCATCGAGAGCCTGCTTTGAGCACCTTAATTTGTTCAAGGTAAAAGTAAGCCGTACTAAACGTGCACCTATCCTAGTGAAAGGCATCAGTGACGTTCATTAA--GTGTACGGTCGAATAGTT-GAGTAA------------------CCCGCCRCCAGGTGGCGCCACTTGGGAACCCCTGGGAGGGTCCTCAACAAAAATCAACTTCGAACGTTTT-AACCGCAACAATTTTAACATACGCTAGTGGAGTCGGAATTACCGCGGCTGCTGGCACCGAACTTGCCCTCCACTTGATCCTAATAGAAGGATTTAAACT-CGATTCATTCCAATTACAGAACATAGATAACTAGTCCTATATTGTTA-----------------------TTCAATATCTCCTTAACAACGATGTGCACTTTTCTCCCAGTAAGACGCTGTGATCCATTAGAAATCCATGCCAATGGTTAGTGGGCTATCAAAAGGCTGACATGAAGTTCGGTGGCAAATGGCGCGATCATATACGGTTGTGTGTATTGCCCATTGAGCATGTTGTGTCCTGAA--ATAGCTGTCTGATGGATTTTAAAGGTCATATTACTCCATCACCCAGGATA-CCCGATCAACAGCGGTTAAATTTGTGATACCTTTGGGACCCGTCTTGAAACACGGACCAAGGAGTCTATCTAGTATGCAAGTAAATGGGGAAACCTATTTGCAGAAAAAACTTGACTTATGGGATTACGAGTGCTCCTCCATCCCGGGGTATTTTCATCAACATATGCAAATGTATATGGAAAATATACCATGAGCGTACTGGATACGACCCGAAAGATGGTGAACTATGCCTGATCAGGTTGAAGTCAGGGGAAACCCTGATGGAGGACCGTAGCAATTCTGACGTGCAAATCGTTGACGTATCCTTTGATCGGCAATTACGGCATTCCGGACGACAATGAGTTCGATGAGTTCAAGTTGATCAAGAATTTCGAGTCGAACGGCAAGATTTGGGTGTCGGGATTGGTGGTGGGCGAATTGTGTGATACGCCTTCCCACTGGCGTATGAAATACAAACTATCGGATTGGATGAAGAAGCACAATATTCCTGGCATCAGCGGCATCGACACTCGGGCTTTGACGAAGAAGATTCGTGAGAATGGAACGGTTTTGGGGAAAATTATCCAGCAACCATCT------GGACCCTTCCCGGGA---ATGGAATTCAAGGACCAGAACGAGAGGAACTTGGTGGACGAAGTTTCTATTAAGAAGACCGCCGTCTACAATCCAAACGGCTCTCCTAAGATTTGCGCCGTAGACTGTGGACTGAAATTGAATCAAATTCGCTGTTTTGTGAAACGCGGAGCTCGCGTTGATGTCGTGCCATGGAATCAAGAATTAAATGCAAAAGACTTTGATGGACTATTTTTGTCGAATGGTCCTGGTGATCCCGTTATGTGCCAAGCGACTGTGCAGAACATTCAGAAGTTTCTCAAGTCGCCCAAACCCAAACCAGTTTTTGGTATCTGCCTTGGCCATCAGCTCTTGTCAACTGCTATTGGCTGCAAAACGTATAAGATGAAATACGGTAATCGCGGCCACAACTTGCCGGCCATACATCATGGCTCCAACCGCTGCTTCATGACCTCGCAGAATCATGGATTTGCTGTTGATGTTTCGAGCATTAACCCCGACTGGGAGCCGCTCTTCACCAACTTGAATGATNNNNNNNNNNNNNNNNNNNNNNNNNNNNNNNNNNNNNNNNNNNNNNNNNNNNNNNNNNNNNNNNNNNNNNNNNNNNNNNNNNNNNNNNNNNNNNNNNNNNNNNNNNNNNNNNNNNNNNNNNNNNNNNNNNNNNNNNNNNNNNNNNNNNNNNNNNNNNNNNNNNNNNNNNNNNNNNNNNNNNNNNNNNNNNNNNNNNNNNNNNNNNNNNNNNNNNNNNNNNNNNNNNNNNNNNNNNNNNNNNNNNNNNNNNNNNNNNNNNNNNNNNNNNNNNNNNNNNNNNNNNNNNNNNNNNNNNNNNNNNNNNNNNNNNNNNNNNNNNNNNNNNNNNNNNNNNNNNNNNNNNNNNNNNNNNNNNNNNNNNNNNNNNNNNNNNNNNNNNNNNNNNNNNNNNNNNNNNNNNNNNNNNNNNNNNNNNNNNNNNNNNNNNNNNNNNNNNNNNNNNNNNNNNNNNNNNNNNNNNNNNNNNNNNNNNNNNNNNNNNNNNNNNNNNNNNNNNNNNNNNNNNNNNNNNNNNNNNNNNNNNNNNNNNNNNNNNNNNNNNNNNNNNNNNNNNNNNNNNNNNNNNNNNNNNNNNNNNNNNNNNNNNNNNNNNNNNNNNNNNNNNNNNNNNNNNNNNNNNNNNNNNNNNNNNNNNNNNNNNNNNNNNNNNNNNNNNNNNNNNNNNNNNNNNNNNNNNNNNNNNNNNNNNNNNNNNNNNNNNNNNNNNNNNNNNNNNNNNNNNNNNNNNNNNNNNNNNNNNNNNNNNNNNNCATATTATTGCTCAAGAAAGAGGAAAAAAAGAAACCTTTGGTACTCTAGGTATAATTTATGCCATATTAGCTATTGGATTACTAGGATTTATTGTATGAGCCCACCACATATTTACTGTCGGGATAGATGTTGACACTCGAGCCTATTTTACCTCTGCAACTATAATTATTGCTGTACCAACAGGAATTAAAATTTTTAGATGATTAGCCACTCTTCACGGTACCCAATTAAATTATTCTCCTTCTTTACTATGAGCTTTAGGATTTGTATTTTTATTTACAGTCGGAGGTTTAACAGGAGTAATTCTAGCTAATTCTTCTATTGATATTGTTCTTCATGATACATATTATGTCGTAGCTCATTTTCATTACGTACTTTCTATAGGAGCAGTATTTGCAATTATAGCTGGATTTATCCATTGATACCCTTTATTTACTGGTATAGTAATAAATAAAAATTGATTAAAATCTCAATTTATTGTTATATTTTTAGGAGTAAATTTAACATTTTTTCCTCAACATTTTTTAGGTTTAGCTGGTATACCTCGCCGATACTCTGATTACCCTGATGTTTATACTTCCTGAAATATTATTTCCACATTAGGGTCTACAATTTCATTATTAGGAATTATTATATTTCTTTTTATTATCTGAGAAAGA

**Synorthocladius sp.** -------------------------------------CAAATTCCA--------------AA--------------------------AACTGGT-----------------GTCAAATT--------------------------------TCCGTCAATTCCTTTAAGTTTCAACTTTGCAACCATACTTCCCCCGGAAACT--AG-CTTT-GGTTTCCCTGAAAGCTACTGAATGCACCATATAA--AGTAGTGACATCCAAT-GCTAGCTGTCATCGTTTACAGTTAGAACTAGGGCGGTATCTAATCGCCTTCGATCCTCTAACTTTCGTTCTTGATTAATGAAAACATCCTTGGCAAATGCTTTCGCTTTAGTTAGTCTTGCGACGGTCTAAGAATTTCACCTCTCGCGCCGCAATACTAATGCCCCCGACTGCTTCTATTAATCATTACCTCT-TGATCTG-TATCAAACCAATAGAAAG-CGATG------------CAACCCTTGCGAGCTACA-CCTA-TAAGACCGAGGTCTTTTTCCATTATTCCATGCAAAAATATTCAAGGCATT-AGAGCCTGCTTTGAGCACCTTAATTTGTTCAAGGTAAAAGTAAGCCGAACTAAATAGACACTTAGCCTAATAAAAGGCATCGGTGCTATTCATTAA--GTGTTCAGTCATGTAGTTCAAGTAA-TCGAAAATGATGACA-GCCATTTACTTGATGGCGTCAC------ACTC-------G-TACTGAACAAAAATGAACTTCGAACGTTTT-AACCGCAACAATTTTAACATACGCTAGTGGAGCTGGAATTACCGCGGCTGCTGGCACCAGACTTGCCCTCCACTTGATCCTTATTGAAGGATTTATACT-CGATTCATTCCAATTACAGAAC-TAAATAATTAG------------------------------------TTCAATATTTTGGCAACAACAATGTGCACTTCTCGCTTAGTAAGACGTTGTGATCCATTAGAAATTCATTCCAATGGTTATTTGGCTATCAATAGGCTGGTGTAAAGTTCATTAGCAAATGGCTGGATCTTTAATGGTCTGGTGTATTGTTTAGTGAGTTCATCATGTTCTGT-CTATAGCTGTCTGATGGATTTTAAAGGTCATAGCATTGCATCACCCAGGTTAACCCGATCAACAGCGGTTAAATTTGGGATACCTTTTGGACCCGTCTTGAAACACGGACCAAGGAGTCTATCTATTGCGCGAGTAAATGGGGAAACCTATTTGCGTAAAAAACTTAACTGATGGGATTACGAGTGCTCCTCCATCCCGGGGTATTTTCATCAA-ACATGCAAATGTATGTTGAAAATATACCATGAGCGTAGTGGATATGACCCGAAAGATGGTGAACTATGCCTGATCAGGTTGAAGTCAGGGGAAACCCTGATGGAGGACCGAAGCAATTCTGACGTGCAAATCGCTCACCTACCCACTTATCGGCAATTACGGCATTCCYAGCGATGAAGAATTTGACGACATCAAGTTGATGAAGCATTTTGAGTCGAACAACAAGATTTGGGTTTCGGGTCTTGTCGTGGGTGAATTGTGCGAGACGCCCTCGCACTGGCGGCGAAAGTACAAACTTGCTGAGTGGATGAAGAAACATAATGTRGTGGGCATCAGTGGCATCGACACGCGAGCTTTGACGAAGAAAATTCGCGAAAATGGAACAATTTTGGGGAAAATTATTCAGCAGTCGGCT------GGGCCGTTTCCAGGG---YTGAAATTTGAGGATCAAAACTTAAGGAATCTCGTGGCGGAAGTTTCAATCAAGAAGTCGGTGACTTACAATCCTTCAGGTGCACCCAGAATTTGTGCCGTTGACTGCGGACTGAAATCGAATCAAATTCGCTGCTTCGTCAACCGCGGTGCCCGGGTCGATGTCGTTCCATGGGATCATCCATTGAACCCGAATGACTTTGACGGACTTTTTCTTTCCAACGGCCCTGGCGATCCCGTCAAATGCGCAAAAACCGTYAAAAACATTCAGAAAGTTCTCGCGTCGCCACAAGTCAAACCAGTTTTYGGGATCTGCCTCGGTCAYCAACTCTTGGCCAGCGCGGTTGGCTGTAAAACTTACAAAATGAAGTAYGGTAATCGTGGACATAATTTGCCGGCAATTCACAACGGCACCAAACGGTGCTTCATGACATCACAAAATCATGGGTTTGCTGTTGATGCCAACACATTGGGCAAGGACTGGGAAGTTCTTTTCACCAACTTGAACGACTTCATTCGCGTGTCCAAGCACATCGGGTCGTCGATGAAAAGTGTCGGCGAGGTCATGGCGATTGGCAGGAAATTCGAGGAAGCTTTTCAGAAAGCGCTGCGGATGGTCGATGAGAATGTGAATGGGTTCGACCCGAACCTCAAGCCGGTGAATGACGAAGAACTCACATCGCCGACAGACAAAAGAATTTTCGTTCTTGCCGCTGCATTGAAGGCTGGCTACACCGTCGACCGACTGTATGATTTGACCAAAATCGACCGTTGGTTCCTCGAGAAAATGAAGAACATCATCGGAATCACAGTTGAACTTGAAAAGCTGAAT---TGCATGATCCCCGATGACCTTTTGACGCAAGCAAAGAAATTTGGATTTTCCGACAAACAAATCGCGACGTACATCAAA--ATCGGAATTGGCTGTGAGGAAGCAGCGCCGCGAGAGTGGAATTTTGCCATTTGTGAAGCAAATTGACACTGTGGCGGGTGAGTGGCCAGCATCGACGAATTACCTTTACTTGACCTACAATGCCAGCACTCACGACATCGAGTTCAAG---GAACAAATGGTCATGGTCATCGGATCAGGAGTTTACCGCATTGGAAGTTCAGTGGAATTTGATTGGTGCGCTGTCGGCTGCCTTCGTGAGCTGAGAAATCTTGGCAAGAAAACCATCATGGTCAACTACAACCCTGAAACCGTGTCCACTGACTACGACATGTGTGACCGTCTTTAC-----------------CATATTATTAGCCAAGAAAGTGGAAAAAAGGAAACTTTCGGGGCCTTAGGTATAATTTATGCTATGCTTGCTATTGGCTTATTAGGATTTGTTGTATGAGCACATCATATATTTACAGTGGGAATAGACGTGGATACCCGAGCCTATTTTACATCTGCCACTATAATTATTGCTGTTCCCACAGGAATTAAAATTTTTAGTTGACTTGCTACACTTCATGGAGCCCAATTAAATAATTCACCTTCTTTATTGTGAGCCTTAGGATTTGTATTCTTATTTACTGTAGGAGGATTAACAGGAGTTGTACTTGCTAATTCTTCTATTGATATTATTCTTCATGATACATATTATGTTGTTGCCCATTTCCATTATGTTCTTTCTATAGGAGCAGTATTTGCCATTATAGCAGGATTTGTTCACTGATACCCCCTATTTACTGGATTAACTATAAATGAAAAGTGATTAAAGTCTCAATTTAGAATTATATTCCTTGGGGTAAATTTAACATTTTTCCCACAACATTTCTTAGGGTTAGCCGGAATACCACGACGATACTCAGACTACCCAGACGCTTATACTTCATGAAATATTATTTCTACAGTAGGATCAACAATTTCATTGATTGGTATTTTATTCTTTCTTTTTATTATTTGAGAAAGT

**Thalassosmittia sp.** --------CTAAGAACGGCCATGCACCACT--ACCCTTAATTTC-A-TGAAAACGCTGTCAA-ATTGTCATACCCTATCAAGTTCGG-ACCTGGTAAGTTTTCCCGTGTTGAGTCAAATTAAGCCGCAAATTCCACGCC-ATGGTGTGGTCTTCCGTCAATTCCTTTAAGTTTCAACTTTGCAACCATACTTCCCCCGGAAACT--AG-CTTT-GGTTTCCCGGAAAGCTACTGAATGCACCATGAAA--AGTAGTGACATCCAATTGCTAGCTGTCATCGTTTACAGTTAGAACTAGGGCGGTATCTAATCGCCTTCGATCCTCTAACTTTCGTTCTTGATTAATGAAAACATCCTTGGCAAATGCTTTCGCTTTAGTTAGTCTTGCGACGGTCTAAGAATTTCACCTCTCGCGCCGCAATACTAATGCCCCCAACTGCTTCTATTAATCATTACCTCT-TGATCTG-TATCAAACCAATAGAAAG-CGATA-------------CTGCCGTTAAGCAAGCA-TCTA-TAAGACCGAGGTCTTTTTCCATTATTCCATGCAAAAATATTCAAGGCATA-AGAGCCTGCTTTGAGCACCTTAATTTGTTCAAGGTAAAAGTAAGCCGAACTAAATAGACGCTTAGCCTAATTAAAGGCATCAGCGCTATTCATTAA--GTGTTCAGTCATATAGTTCAAGCAA-TCGGAAATGATGACA-GCCATTTACTTGATGGCGTCAC------ACCC-------G-TGCTGAACAATAATCAACTTCGAACGTTTT-AACCGCAACAATTTTAATATACGCTAGTGGAGCTGGAATTACCGCGGCTGCTGGCACCAGACTTGCCCTCCACTGGATCCTCATTAAAGGATTTATACT-TGATTCATTCCAATTACAGAAC-TAAATAATTAGTTCTATATTGTTATTTTTCGTCACTACCTCC-----TTCAATATTCCTTTAAAAGCAATGTGCACTTCTCTCTTAGTAAGACGCTGTGATCCATTAGAAATTTGTTCCCACATTCATTTAGCTGTCAATAGGCCAATGTGAAGTTCATTGGCAAATAGCATGATCTTTTACGGTTTTGTATATTGTTTGATGAGCACATTGTAACCTGTACAATAGCTGTCTGATGGATTTTAAAGGTCATATTATTGCATCACTCGGAATAACCCGATCAACAGCGGTTAAATTTGGCATACCTTCTGGACCCGTCTTGAAACACGGACCAAGGAGTCTATCTAGTATGCAAGTAAATGAGGAAACTTATTTGCGTAAAAAACTTGACTGATGGGATTACGAGTATTCTTCCATCCCGGGGTATTTTTATCAACATAT-----TTT-TATGGAAAATATACCATGAGCATACTGGATATGACCCGAAAGATGGTGAACTATGCCTGATCAGGTTGAAGTCAGGGGAAACCCTGATGGAGGACCGAAGCAATTCTGACGTGCAAATCGCTAACTTATCCACTCATCGGAAACTACGGAATACCGAGCGACGAAGAATTCGACGAACACAAATTGATGAAAAACTTCGAGTCTAACAATAAGATTTGGGTATCAGGGCTGGTTGTTGGTGAATTATGTGAGACTCCATCGCATTGGCGTCAAAAGTACAAGCTTGCTGAATGGATGAAGAAGCACAACATAGTGGGAATCAGTGGAATTGACACTAGAGCTTTGACGAAGAATATTCGAGAAAATGGAACCATCTTAGGAAAAATCATCCAACAATCTTCG------GGGCCGTTTCCAGGA---CTCGAATTTAAAGATCAAAACCAAAGGAATCTAGTTGACGAGGTTTCTTTGATAAAGCCGATAACTTACAACGCTAAGGGATCTCCTCGAATTTGTGCTGTTGATTGTGGTCTGAAGTTAAACCAGATTCGTTGCTTTGTTCAACGCGGAGCTCGAGTTGATGTTGTGCCATGGAATCATCCTCTTAAAGCTGAAGACTTCGATGGTCTCTTCTTGAGCAACGGTCCAGGAGATCCAGTCATGTGTAAAACCACCGTTAAAAATCTTCAAAATTTCCTSACGAAGCCGAACACTAAACCAATTTTTGGTATTTGCTTGGGTCATCAGCTTCTTGCGACTGCTATTGGATGTAAGACTTACAAGCTGAAATATGGAAACCGTGGTCACAATTTACCAGCATTRCAYCACGGTTCTAAGCGTTGTTTCATGACTTCTCAGAACCATGGATTCGCTGTTGATGTCACGTCTATAAGCAAGGATTGGGAACCGCTTTTCACCAATCTCAACGAT---------------------------------------AGTGTCGGAGAAGTAATGGCAATCGGAAGAAAGTTTGAAGAAGCTTTCCAAAAAGCTTTGCGAATGGTTGACGAGAATGTAACTGGATTCGATCCGAACTTGAAGCCTGTCAGTGACGAAGAACTTGAGACTCCAACAGACAAACGAATGTTTGTTCTTGCGGCAGCTTTGAAAGCTGGATACTCTGTCGATCGTCTTTATGATTTAACAAAGATTGATCGATGGTTCTTGGAGAAGATGAAGAACATCAACAATGTGACGTTAGAGCTTGAAAACCTGAATTGGTCAGTGATGCCAGACGAACTTCTGAGGAAAGCGAAGCAACTTGGTTTCTCTGATAAGCAAATCGCGAACTGTATTAAA--TCAAGAGCTCGTAGTTCGTAAACAACGTCGCGAAAGTGAAATCACTCCTTTCGTGAAACAGATTGACACTGTCGCCGGTGAATGGCCCGCATCAACGAATTATCTTTATTTGACTTACAATGCAAGCGCTCATGATATTGAATTTAAACAAGAAAAAATGATAATGGTCATTGGTTCAGGAGTTTATCGCATCGGAAGTTCGGTCGAGTTCGACTGGTGTGCCGTCGGTTGCTTGCGTGAATTGAGAAATTTGGGAAAGAAAACGATTATGGTCAACTACAATCCCGAGACTGTTTCTACTGATTACGACATGTGCGATCGCTTGTACTTCGAAGAGATTTCTTTCATATTATTAGACAAGAGAGAGGAAAAAAAGAAACATTTGGTTCTTTAGGTATAATTTACGCAATACTAGCTATTGGATTACTTGGGTTTGTAGTTTGAGCTCACCATATATTTACAGTTGGGATAGATGTTGATACCCGAGCTTATTTTACTTCAGCTACAATAATTATTGCTGTACCCACAGGGATTAAAATTTTTAGTTGATTAGCAACTTTACATGGAGCACAATTAAATAATTCTCCCTCCTTACTTTGAGCTTTAGGCTTTGTATTTTTATTTACAGTTGGAGGGTTAACGGGAGTAATTCTCGCAAATTCTTCTATTGATATTGTATTACATGACACTTATTATGTAGTTGCCCATTTTCACTATGTATTATCTATAGGTGCAGTATTTGCAATTATAGCAGGATTTGTGCATTGATATTCATTATTTACAGGATTATCAATAAATGAAAAATGATTAAAATCTCAATTTGCTATTATATTTTTGGGGGTGAATTTAACATTTTTCCCTCAACACTTTTTAGGTTTAGCTGGAATACCTCGACGTTATTCAGATTATCCAGACGCTTATATTTCATGAAATGTTGTTTCTACTGTGGGATCAACAATTTCTTTACTTGGTATTTTATATTTCTTATTTATTATTTGAGAAAGA

**Thienemanniella sp.** ---------------CGGCCATGCACCACT--ACCCTTAATTTC-A-TGAAAGCGCTGTCAA-GCTGTCAAACCCTATTAAGTTCTG-ACCTGGTAAGTTGTCCCGTGTTGAGTCAAATTAAGCCGCAACATCCACTGC-CGGTGATGATCTTCCGTCAATTCCTTTAAGTTTCAACTTTGCAACCATACTTCCCCCGGAAACT--AG-CTTT-GGTTTCCCGAAGAGCTACTGAATGCACCATGAAA--AGTAGTGACATCCAATCGCTAGCTGTCATCGTTTACAGTTAGAACTAGGGCGGTATCTAATCGCCTTCGATCCTCTAACTTTCGTTCTTGATTAATGAAAACATCCTTGGCAAATGCTTTCGCTTTAGTTAGTCTTGCAACGGTCTAAGAATTTCACCTCTCGCGCTGCAATACTAATGCCCCCAACTGCTTCTATTAATCATTACCTCT-TGATCTG-TATCAAACCAACAGAAAATCAATG------------CCAAACTTGAGCTTGACA-TTAAACAAGACCGAGGTCTTTTTCCATTATTCCATGCAAAAATATTCAAGGCATA-AGAGCCTGCTTTGAGCACCTTAATTTGTTCAAGGTAAAATTAAGCTGAACTAAATAGACACTTAGCCTAATAAAAGGCATCGGTGCTATTCATTTA--GTGTCCAGTCAAATAGTTCAAGTAA-TCGAAAATGATAGTA-GCCATGTACTTTATGACACTAC------ACTC-------G-TACTGAACAAAAATCAACTTCGAACGTTTT-AAACGCAACAATTTTAATATACGCTAGTAGAGCTGGAATTACCGCGGCTGCTGGCACCAGACTTGCCCTCTACTTGATCCTCAACAAAGGATTTATACT-TGATTCATTCCAATTACAGAAC-TAAATGATTAGTTCTATATTGTTATTTTTCGTCACTACCTCC-----TTCAATATTCCATTAAAAACAATGTGCACTTCTTTCTTAGTAAGACGCTGTGATCCATTAGAAATTCATTTCAATGATTATGAGGCTATCAATAGGCCAATATGAAGTTCATTAACAAATAATATTATCTTTAATGGTTTTATATATTGTATGATGAGCATGTTGTGTTCTG--CTATAGCTGTCTGATGGATTTTAAAGGTCATGTTATTGCATCACTCATGACATCCTGATCAACAGCGGTTAAATTTGGGATACCTTTTGGACCCGTCTTGAAACACGGACCAAGGAGTTTATCTAATATGCGAGTAAATGAGGAAACTTATTTGCGTAAATAAATTAACTGATGGGATTACGAGTATTCTTCCATCCCGGAGTATTTTCATCAACATATGCAAATGTATATGGAAAATATATCATGAGCATACTGGATATGACCCGAAAGATGGTGAACTATGCCTGATCAGGTTGAAGTCAGGGGAAACCCTGATGGAAGACCGAAGCAATTCTGACGTGCAAATCG---------------------AATTATGGAATACCGCGTGATGATGAATTTGATGAATTTGGACTGATGAAGAATTTTGAATCGAATAACAAGATTTGGATCTCTGCATTAATCGTAGGCGAGCTTTGTGAGACACCCTCGCATTGGCGTGTTAAATATAAATTGGCTGAATGGATGAAAAAGCACAACGTGGTCGGTATAAGTGGCATTGACACTCGAGCACTCACTAAAAAGATTCGAGAAAATGGAACAATTCTTGGAAAAATCATTCAAAAGTCATCA------GGGCCTTTCTATGAC---CTTGATTTTAAGGACCAAAATAAACGTAATCTTGTCGATGAAGTTTCTACTAAAAAGACAATCACTTACAATTCCAAAGGTTCACCGAGAATTTGTGCAGTTGATTGTGGATTAAAATTGAATCAAATTCGTTGTTTTACTAAACGTGGAGCTCGTGTTGATATTGTGCCATGGGATGCTGACTTAAATCCTCAAAATTATGATGGTCTGTTCTTATCAAATGGTCCTGGTGATCCTGCCATTTGCGAAAAAACTGTGAAAAATTTACAAAAAGTTCTGGAATCTAAAATTACTAAGCCAATTTTTGGCATTTGTCTTGGACATCAACTTCTCTCYACTGCTGCYGGATGCAAAACTTATAAGTTAAAATATGGAAATCGTGGTCATAACTTACCAGCTCTTCATCATGGAACCAATCGTTGTTTTATGACATCTCAAAATCATGGATTTGCTGTAGATGTAGCAACACTTAGCTGTGATTGGGAGCCACTTTTTACAAATCTCAATGATNNNNNNNNNNNNNNNNNNNNNNNNNNNNNNNNNNNNNNNNNNNNNNNNNNNNNNNNNNNNNNNNNNNNNNNNNNNNNNNNNNNNNNNNNNNNNNNNNNNNNNNNNNNNNNNNNNNNNNNNNNNNNNNNNNNNNNNNNNNNNNNNNNNNNNNNNNNNNNNNNNNNNNNNNNNNNNNNNNNNNNNNNNNNNNNNNNNNNNNNNNNNNNNNNNNNNNNNNNNNNNNNNNNNNNNNNNNNNNNNNNNNNNNNNNNNNNNNNNNNNNNNNNNNNNNNNNNNNNNNNNNNNNNNNNNNNNNNNNNNNNNNNNNNNNNNNNNNNNNNNNNNNNNNNNNNNNNNNNNNNNNNNNNNNNNNNNNNNNNNNNNNNNNNNNNNNNNNNNNNNNNNNNNNNNNNNNNNNNNNNNNNNNNNNNNNNNNNNNNNNNNNNNNNNNNNNNNNNNNNNNNNNNNNNNNNNNNNNNNNNNNNNNNNNNNNNNNNNNNNNNNNNNNNNNNNNNNNNNNNNNNNNNNNNNNNNNNNNNNNNNNNNNNNNNNNNNNNNNNNNNNNNNNNNNNNNNNNNNNNNNNNNNNNNNNNNNNNNNNNNNNNNNNNNNNNNNNNNNNNNNNNNNNNNNNNNNNNNNNNNNNNNNNNNNNNNNNNNNNNNNNNNNNNNNNNNNNNNNNNNNNNNNNNNNNNNNNNNNNNNNNNNNNNNNNNNNNNNNNNNNNNNNNNNNNNNNNNNNNNNNNNNNNNNNNNNNNNNNNNNNNNNNNNNNNNNNNNNNNNNNNNNNNNNNNNNNNNNNNNNNNNNNNNNNNNNNNNNNNNNNNNNNNNNNNNNNNNNNNNNNNNNNNNNNNNNNNNNNNNNNNNNNNNNNNNNNNNNNNNNNNNNNNNNNNNNNNNNNNNNNNNNNNNNNNNNNNNNNNNNNNNNNNNNNNNNNNNNNNNNNNNNNNNNNNNNNNNNNNNNNNNNNNNNNNNNNNNNNNNNNNNNNNNNNNNNNNNNNNNNNNNNNNNNNNNNNNNNNNNNNNNNNNNNNNNNNNNNNNNNNNNNNNNNNNNNNNNNNNNNNNNNNNNNNNNNNNNNNNNNNNNNNNNNNNNNNNNNNNNNNNNNNNNNNNNNNNNNNNNNNNNNNNNNNNNNNNNNNNNNNNNNNNNNNNNNNNNNNNNNNNNNNNNNNNNNNNNNNNNNNNNNNNNNNNNNNNNNNNNNNNNNNNNNNNNNNNNNNNNNNNNNNNNNNNNNNNNNNNNNNNNNNNNNNNNNNNNNNNNNNNNNNNNNNNNNNNNNNNNNNNNNNNNNNNNNNNNNNNNNNNNNNNNNNNNNNNNNNNNNNNNNNNNNNNNNNNNNNNNNNNNNNNNNNNNNNNNNNNNNNNNNNNNNNNNNNNNNNNNNNNNNNNNNNNNNNNNNNNNNNNNNNNNNNNNNNNNNNNNNNNNNNNNN

**Tonnoirocladius commensalis** TCCACGAACTAAGAACGGCCATGCACCACT--ACCCTTAATTTC-A-AGAAAACGCTATCAA-GTTGTCATACCCTATTAAGTTCGG-ACCTGGTAAGTTTTCCCGTGTTGAGTCAAATTAAGCCGCAAATTCCAAGTC-ATGGTGTGAACTTCCGTCAATTCCTTTAAGTTTCAACTTTGCAACCATACTTCCCCCGGAAACT--AG-CTTT-GGTTTCCCGGAAAGCTACTGAATGCACCATGAAA--AGTAGTGACATCCAATTGCTAGCTGTCATCGTTTACAGTTAGAACTAGGGCGGTATCTAATCGCCTTCGATCCTCTAACTTTCGTTCTTGATTAATGAAAACATCCTTGGCAAATGCTTTCGCTTTAGTTAGTCTTGCGACGGTCTAAGAATTTCACCTCTCGCGCCGCAATACTAATGCCCCCAACTGCTTCTATTAATCATTACCTCT-TGATCTG-TATCAAACCAATAGAAAG-CGACG------------CCACGGTTGCCCGCAACG-CCTA-TAAGACCGAGGTCTTTTTCCATTATTCCATGCAAAAATATTCAAGGCATA-AGAGCCTGCTTT----------------------------------------------------------------------------------------------------------------------------------------------------------------------------------------------------------------------------------------------------------------------------------------------------------------------------------------------------------------------------------TTCAATATTCCGTTAAAAGCAATGTGCACTTCTCCCTTAGTAAGACGCTGTGATCCATTAGGAATTCATTCCAATGATTATTTGGCTATCAATGGGCCGATGTGAAGTTCATTAGCAAATAACTCGATCCTTTACGGTTGGGTATATTGTTTGGTGAGCGCATTGTCACCCG-ATTATAGCTGTCTGATGGATTTTAAAGGTCATATTATTGCATCACTCACAATAACCCGATCAACAGCGGTTAAATTTGGGATACCTTTTGGACCCGTCTTGAAACACGGACCAAGGAGTCTATCTAGTATGCAAGTAAATGAGGAAACTTATTTGCAGAAAAAACTTGACTGATGGGACTACGAGTGCTCCTCCATCCCGGGGTATTTTTATCAACATATGCAAATGTATATGGAAAATATACCATGAGCATACTGGATATGACCCGAAAGATGGTGAACTATGCCTGATCAGGTTGAAGTCAGGGGAAACCCTGATGGAGGACCGAAGCAATTCTGACGTGCAAATCGTTGACTTATCCGTTGGTTGGAAATTACGGGATTCCGAGCGATGAAGAGTTCGACGAACATAAACTGATGAAACATTTCGAGTCGAATAATAAGATTTGGGTTTCTGGTTTGGTGGTTGGTGAACTGTGTGAAACACCATCGCATTGGCGCCAGAAGTATAAGCTGGCTGAATGGATGAAGAAACACAACGTTGTTGGCATCAGTGGAATTGACACCCGAGCTTTAACTAAGAAAATTCGGGAAAACGGAACCGTTTTGGGTAAAATTATCCATCAATCTGCT------GGACCCTTTCCGGAT---ATGAAGTTCAAAGATCAGAACCAACGCAACCTCGTCGACGAAGTCTCAATCAAAAAGCCCATAACATACAACGAGAAAGGTTCGCCAAGAATCTGTGCCATCGACTGCGGTTTGAAGTTGAATCAAATTCGCTGTTTCATCAACCGAGGAGCCCGCGTAGATTTAGTGCCATGGGATCAACCATTGAATCCCGATGAATTTGACGGTCTCTTCCTCAGCAACGGTCCAGGTGACCCGGTCATGTGTCAAAAGACTGTCAAAAACATTCAGAACTTCCTTGCGTCACCTAAGGTTAAGCCAACTTTTGGTATCTGCTTGGGCCATCAGTTACTTTCAACTGCAATAGGCTGTAAAACTTACAAATTGAAATACGGAAATCGCGGGCACAATCTTCCAGCTCTTCACCATGGAACTAATCGCTGCTTCATGACCTCTCAAAACCATGGATTTGCTGTTGATGTGGAGACAATTGTCAAGGATTGGGAGCCTCTTTTCACCAACTTAAACGACTTCATTCGAGTCAGCAAGAATATTGGAAGTTCCATGAAGAGCGTCGGTGAAGTGATGGCAATTGGACGAAAGTTTGAAGAAGCTTTTCAGAAAGCCTTGCGAATGGTCGATGAGAATGTCGATGGTTTCAACCCGAATTTGAAGCCAGTTTGCGATGAAGAACTCATGACGCCGACCGACAAGCGAATGTTTGTTCTTGCTGCTGCTCTGAAAGCTGGTTACACAGTGGATCGTCTTTACGATTTGACAAAGATCGACCGTTGGTTCCTTGAAAAGATGAAAAATATCAACGCAGTCACGTTGGAACTTGAGACGCTCAAC---TGCGTAATCTCGGATGAACTTTTAGCTCGAGCAAAGAAACTCGGATTTTCGGACAAGCAAATCGCAAATTCAATCAAA--ATCAGAACTTGAAGTGAGAAAGCAACGTCGTGATAGTGGAATTTTGCCGTTTGTGAAGCAAATTGACACAGTTGCTGGTGAATGGCCGGCATCTACAAATTATCTGTATTTGACTTACAACGCGAGCAGCCATGACATTGAGTTCACC---GAACAAATGGTGATGGTTATTGGATCTGGGGTTTATCGTATTGGAAGCTCAGTTGAATTTGATTGGTGTGCAGTTGGATGTTTGAGAGAACTTCGAAAGCTTGGCAAAAAGACCATCATGGTGAACTACAATCCTGAAACTGTATCAACCGACTACGACATGTGCGACCGCTTGTACTTTGAGGAAATTTCATTCATATTATTAGCCAAGAAAGAGGAAAAAAAGAAACCTTTGGTTCTTTAGGAATAATCTATGCTATACTTGCTATTGGTTTACTAGGTTTCATTGTTTGAGCACACCATATATTTACAGTAGGAATAGACGTAGATACCCGAGCTTACTTTACTTCAGCTACAATAATTATTGCAGTACCTACGGGAATTAAAATTTTTAGTTGACTAGCTACATTACACGGAACCCCTATAAATAATTCACCCTCTCTTTTATGAGCCTTAGGGTTTGTATTTTTATTTACCGTTGGAGGATTAACAGGAGTTATTTTAGCTAATTCTTCTCTTGATATTGTTCTTCATGACACTTATTATGTAGTTGCCCATTTTCATTATGTACTTTCAATGGGAGCTGTATTTGCAATTATGGCAGGATTTGTTCATTGATACACTTTATTTACAGGATTAATCATAAATGAAAAATGATTAAAATCTCAATTTACAATTATATTTTTAGGAGTAAATTTAACATTTTTCCCACAACACTTTTTAGGGTTAGCTGGGATACCCCGACGATACTCTGATTATCCAGATGCTTACACTTCATGAAATATTGTATCTACAGTTGGGTCTACAATTTCTTTATTTGGAATTTTATTTTTTATTTTTATTATTTGAGAAAGA

**Tvetenia sp.** --------CTAAGAACGGCCATGCACCACT--ACCCTTAATTTC-A-AGAAAACGCTATCAA-GTTGTCATACCCTATTAAGTTCGG-ACCTGGTAAGTTTTCCCGTGTTGAGTCAAATTAAGCCGCAAATTCCACATC--TTGTGTGATCTTCCGTCAATTCCTTTAAGTTTCAACTTTGCAACCATACTTCCCCCGGAAACT--AG-CTTT-GGTTTCCCGGAAAGCTACTGAATGCACCATGAAT--AGTAGTGACATCCAATTGCTAGCTGTCATCGTTTACAGTTAGAACTAGGGCGGTATCTAATCGCCTTCGATCCTCTAACTTTCGTTCTTGATTAATGAAAACATCCTTGGCAAATGCTTTCGCTTTAGTTAGTCTTGCGACGGTCTAAGAATTTCACCTCTCGCGCCGCAATACTAATGCCCCCAACTGCTTCTATTAATCATTACCTCT-TGATCTG-TATCAAACCAATAGAAAG-CACCC-------------ACCTCACGGTAAGGTGA------TAAGACCGAGGTCTTTTTCCATTATTCCATGCAAAAATATTCAAGGCATA-AGAGCCTGCTTTGAGCACCTTAATTTGTTCAAGGTAAAAGTAAGCCAAACTAAATAGACGCCTAGCCTAATTAAAGGCATCAGCGCTATTCATTAA--GTGTTCAGTCATATAGTTCGAGTAA-TCGGAAATGATGACA-GCCATTTACTTGATGGCGTCAC------ACCC-------G-TACTGAACAATAATCAACTTCGAACGTTTT-AACCGCAACAATTTTAATATACGCTAGTGGAGCTGGAATTACCGCGGCTGCTGGCACCAGACTTGCCCTCCACTTGATCCTCATTAAAGGATTTATACT-TGATTCATTCCAATTACAGAAC-TAAATAATTAGTTCTATATTGTTATTTTTCGTCACTACCTCC-----TTCAATATT-TATTAATCATAATGTGCACTTCTCTCTTAGTAAGACGCTGTGATCCATTAGAAATTTATTCTAATAATTATTTGGCTATCAATGGGCCGATATGAAGTTCATTAGCAAATAACTTGATCTTTAATGGTTGGGTATATTGCTTGGTGAGCATATTGTCACTCG-ATTATAGCTGTCTGATGGATTTTAAAGGTTGTATTATTGCATCACTCAGAATA-CCCGATCAACAGCGGTTAAATTTGGGATACCTTTTGGACCCGTCTTGAAACACGGACCAAGGAGTCTATCTAGTACGCAAGTAAATGAGGAAACTTATTTGCGGAAAAAACTTGACTGATGGGACTACGAGTGCTCCTCAATCCCGGGGTATTTTTATCAACATATGCAAATGTATATGGAAAATATACCATGAGCGTACTGGATATGACCCGAAAGATGGTGAACTATGCCTGATCAGGTTGAAGTCAGGGGAAACCCTGATGGAGGACCGAAGCAATTCTGACGTGCAAATCGTTGACTTATCCACTTATCGGCAATTATGGCATCCCGAGCGACGAAGAATTCGATGAACATCAACTGATGCGCCACTTTGAATCGAACAATAAGATTTGGGTGTCGGGACTAGTTGTGGGTGAACTGTGTGAGACACCGTCTCACTGGCGCACAAAGTACAAGCTCGCCGAATGGATGAAAAAGCATAACATAGTTGGAATCAGTGGAATCGACACACGAGCTTTGACGAAGAAAATTAGAGAGAATGGAACGGTGCTGGGGAAAATCATTCAGAAGTCGTCT------GGGCCGTTCCCTGAT---CTAGCGTTCAAGGATCAAAATGAAAGGAATCTAGTTGATGAGGTGTCCATCAAAAAACCGGTGACTTACAACGCAAAGGGAACACCGAGAATTTGCGCTGTAGACTGCGGTTTGAAGCTGAATCAAATTCGATGCTTTATAGCAAGAGGAGCACGAGTTGACCTGGTTCCGTGGGATTATCCTCTGAACCCCGACAACTTTGATGGTCTCTTTCTGAGCAATGGTCCAGGTGATCCCGTCATGTGCAAGAAGACTGTGAAAAACATCCAAAACTTCCTTTCTTTGCCAAAGACGCGACCGATTTTTGGTATCTGCTTAGGACACCAGCTTCTGGGTACAGCGATCGGATGCAAGACATACAAGCTAAAGTATGGCAACCGTGGTCATAACTTGCCAGCACTCCACCATGGCACTAACAGGTGTTTTATGACTTCGCAGAATCATGGATTCGCTGTTGATGTCAAGTCTTTAGGTAAAGATTGGGAGCCTCTCTTCACCAACTTGAACGAC------------------------------------AAAAGTGTGGGGGAGGTGATGGCCATTGGCCGAAAGTTTGAGGAAGCTTTTCAGAAAGCGTTGCGAATGGTTGACGAAAACGTTAATGGATTCGATCCGAATTTGAAAGCWGTGAATGATGAGGAATTGACGACTCCGACAGACAAGCGAATGTTTGTTCTAGCAGCGGCTTTGAAAGCTGGCTATTCCGTCGATAGGTTATACGATTTGACGAAGATCGATCGGTGGTTTCTCGAAAAGATGAAGAACATCAACGGTATCACGATCGAGCTGGAAAAATTGAAT---TGCGTTGTGTCTGATGAGTTGTTGTTGCAGGCTAAGAAACTAGGCTTTTCCGACAAGCAGATCGCGTCGCTCATCAAC--ATCAGAACTCGAAGTGAGAAAGCAGAGACGTGGCAGTGGGATTCTCCCATTTGTAAAGCAGATCGACACGGTTGCCGGTGAATGGCCAGCATCGACCAATTATCTCTACTTGACATATAACGCAAGCAGCCACGATATTGAGTTTGAC---GAGCAAATGGTGATGGTCATTGGATCTGGTGTTTATCGCATTGGAAGCTCTGTTGAGTTTGACTGGTGTGCTGTTGGGTGCTTAAGAGAATTGAGAAATCTTGGAAAGAAAACTATCATGGTGAATTATAATCCCGAAACAGTATCGACAGATTACGACATGTGCGATCGACTCTACTTTGAAGAGATTTCATTCATATTATTAGCCAAGAAAGAGGAAAAAAAGAAACATTCGGCGCATTAGGAATAATTTATGCAATATTAACAATTGGTCTTTTAGGGTTTGTTGTTTGAGCTCACCATATATTCACAGTCGGCATAGATGTTGACACACGAGCTTATTTTACTTCAGCCACAATAATTATTGCCGTACCAACAGGCATTAAAATTTTTAGATGATTAGCAACTTTACATGGAACTCAAATGAATAATTCTCCTTCTATACTATGAGCCTTAGGATTTGTATTTTTATTTACTGTCGGGGGACTAACTGGAGTCATTCTAGCAAATTCTTCTATTGATATTGTATTACATGATACTTACTACGTAGTAGCTCACTTTCACTATGTTTTATCTATAGGAGCAGTATTCGCAATTATAGCAGGATTTGTGCATTGATACCCTTTATTTTCCGGCCTATCAATAAATGAAAAATGATTAAAATCTCAATTTCTAATTATATTCTTAGGAGTCAATTTAACATTTTTTCCCCAACATTTTTTAGGATTAGCTGGAATACCTCGACGATACTCAGACTACCCCGACGCTTACACTTCTTGAAACATTGTTTCTACGATCGGATCTACAATTTCATTATTTGGAATTTTATTTTTTATTTTTATTATTTGAGAAAGA

**Undet. Orthocladiinae** ---------------------------------------------A-AGAAAACGCTATCAA-GTTGTCATACCCTATTAAGTTCGG-ACCTGGTAAGTTTTCCCGTGTTGAGTCAAATTAAGCCGCAAAATCCAAGCC-ATGGTGTGAACTTCCGTCAATTCCTTTAAGTTTCAACTTTGCAACCATACTTCCCCCGGAAACT--AG-CTTT-GGTTTCCCGGAAAGCTACNGAATGCACCATGAAA--AGTAGTGACATCCAATTGCTAGCTGTCATCGTTTACAGTTAGAACTAGGGCGGTATCTAATCGCCTTCGATCCTCTAACTTTCGTTCTTGATTAATGAAAACATCCTTGGCAAATGCTTTCGCTTTAGTTAGTCTTGCGACGGTCTAAGAATTTCACCTCTCGCGCCGCAATACTAATGCCCCCAACTGCTTCTATTAATCATTACCTCT-TGATCTG-TATCAAACCAATAGAAAG-CGAAA----------GTGCGTTTTAAGGCGCNCNA-TCTA-TAAGACCGAGGTCTTTTTCCATTATTCCATGCAAAAATATTCAAGGCATT-AAAGCCTGCTTTGAGCACCTTAATTTGTTCAAGGTAAAATTAAGCTGAACTAACTAGACACCTGTCCTAATTAAAGGCATCAGTGCTAACCATTAA--GTGTTCAGTCAAATAGTTCAAAAAA-TCGGAAATGAAGTCA-ACCA--AACNTGATGGAGACTC------ACCC-------GCTATTGAACAACAATCAACTTCGAACGTTTT-AACCGCAACAACTTTNATATACGCTAGTGGAGCTGGAATTACCGCGGCTGCTGGCACCAGACTTGCCCTCCACTTGATCCTAATTGAAGGATTTAAACT-CGATTCATTCCAATTACAGAAC-TAAATGATTAGTTCTATATTGTTATTTT-------------------TTCAATATTCCCTTAAAAGCAATGTGCACTTCTTCCTTAGTAAGACGCTGTGATCCATTAGAAATTCATTCCAATGATTATTTAGCTATCAATGGGCCGACATGAAGTTCATC-GTAAACAACTTGATCCTTAATGGTTGGGTAYATTGCATGATGAGCGTGTTGATACCTGCATTATAGCTGTCTGATGGATTTTAAAGATCATATTATTGCATCACTCATGATAATCCGATCAACAGCGGTTAAATTTGGGATACCTTTTGGACCCGTCTTGAAACACGGACCAAGGAGTCTATCTAGTATGCAAGTAAATGAGGAAACTTATTTGCATAAAAAACTTGACTGATGGGACTACGAGTGCTTCTCCATCCCGGGGTATTTTCATCAACATATGCAAATGTATATGGAAAATATACCATGAGCATACTGGATATGACCCGAAAGATGGTGAACTATGCCTGATCAGGTTGAAGTCAGGGGAAACCCTGATGGAGGACCGAAGCAATTCTGACGTGCAAATCGCTGACTTATCCGTTGATTGGAAATTATGGAATCCCAAGCGATGAAGAATTCGACGAACACAAATTGATGAAACATTTTGAGTCGAACAACAAGATTTGGGTTTCGGGTTTGGTCGTCGGTGAATTGTGTGAAACTCCATCGCACTGGCGTCAGAAATACAAACTCGCCGAATGGATGAAGAAGCACAACATCGTCGGAATCAGCGGCATTGACACTCGCGCTTTGACCAAGAAGATTCGTGAGAATGGAACTGTTTTGGGAAAAATCATTCAGCGATCTTGT------GGACCGTTCCCGGAT---TTGGTGTTTAAAGATCAAAACAAACGGAACCTTGTCGATGAAGTGTCGGTGAAGAAGCCGGTGACTTACAACGCGAAAGGCTCGCCAAGAATTTGTGCCATTGACTGCGGATTGAAATTGAATCAAATCCGTTGTTTCATTCAGCGCGGAGCTCGCGTTGATGTCGTGCCATGGGATTACGAGCTTAATCCCGATAACTTTGATGGTTTATTTTTAAGCAACGGTCCAGGTGACCCCGTAATGTGCCAAAAAACCGTCCAAAACATTCAGAAATTCCTCGCTTTGCCAAAAGTCAGACCGATTTTCGGTATTTGCTTGGGTCATCAGCTCCTGTCCACTGCTGTCGGTTGCAAAACCTACAAAATGAAATACGGAAATCGCGGCCACAATCTCCCAGCGGTTCATCACGGCACCAAACGATGCTTCATGACGTCGCAAAATCACGGATTTGCGGTTGATGTGAAGACGCTCGGCAAGGATTTTGAGCCGCTGTTC---------------NNNNNNNNNNNNNNNNNNNNNNNNNNNNNNNNNNNNNNNNNNNNNNNNNNNNNNNNNNNNNNNNNNNNNNNNNNNNNNNNNNNNNNNNNNNNNNNNNNNNNNNNNNNNNNNNNNNNNNNNNNNNNNNNNNNNNNNNNNNNNNNNNNNNNNNNNNNNNNNNNNNNNNNNNNNNNNNNNNNNNNNNNNNNNNNNNNNNNNNNNNNNNNNNNNNNNNNNNNNNNNNNNNNNNNNNNNNNNNNNNNNNNNNNNNNNNNNNNNNNNNNNNNNNNNNNNNNNNNNNNNNNNNNNNNNNNNNNNNNNNNNNNNNNNNNNNNNNNNNNNNNNNNNNNNNNNNNNNNNNNNNNNNNNNNNNNNNNNNNNNNNNNNNNNNNNNNNNNNNNNNNNNNNNNNNNNNNNNNNNNNNNNNNNNNNNNNNNNNNNNNNNNNNNNNNNNNNNNNNNNNNNNNNNNNNNNNNNNNNNNNNNNNNNNNNNNNNNNNNNNNNNNNNNNNNNNNNNNNNNNNNNNNNNNNNNNNNNNNNNNNNNNNNNNNNNNNNNNNNNNNNNNNNNNNNNNNNNNNNNNNNNNNNNNNNNNNNNNNNNNNNNNNNNNNNNNNNNNNNNNNNNNNNNNNNNNNNNNNNNNNNNNNNNNNNNNNNNNNNNNNNNNNNNNNNNNNNNNNNNNNNNNNNNNNNNNNNNNNNNNNNNNNNNNNNNNNNNNNNNNNNNNNNNNNNNNNNNNNNNNNNNNNNNNNNNNNNNNNNNNNNNNNNNNCATATTATTAGTCAAGAAAGAGGGAAAAAGGAAACTTTTGGGGCTTTAGGTATAATTTATGCTATACTTTCTATTGGATTATTAGGATTTATTGTATGAGCCCACCATATATTTACAGTAGGAATAGATGTCGATACCCGAGCATATTTTACTTCAGCTACTATAATTATTGCGGTTCCTACAGGTATTAAAATTTTTAGTTGATTAGCAACACTTCACGGAACCCCCTTAAATAATTCAGCTTCTTTATTATGAGCTTTAGGATTTGTTTTTTTATTTACTGTAGGTGGATTAACGGGAGTAATTTTAGCTAATTCTTCTATTGATATTGTTCTTCACGACACGTATTATGTTGTTGCCCATTTTCATTATGTCCTATCTATGGGGGCTGTATTTGCTATTATAGCTGGGTTTGTACATTGATATTCTCTATTTACAGGACTAAGAATAAATGAAAAATGATTAAAGTCTCAATTTGCTATTATATTTTTAGGAGTAAATTTAACATTTTTCCCTCAACATTTTTTAGGACTAGCCGGGATACCCCGTCGTTATTCAGACTATCCAGATTCTTACACTTCATGAAATATTATTTCTACAGTAGGGTCTACAATTTCTTTATTTGGAATTTTATTTTTTATTTTTATTATTTGAGAAAGA

**Xylotopus par** ---------------CGGCCATGCACCACT--ACCCTTAACTTC-G-AGAAATCGCTCTCAA-AATGTCTTACCCTGTTAAGTTCGG-ACCTGGTAAGCTTCCCCGTGTTGAGTCAAATTAAGCCGCATATTCCACTTCATTGTTGTGCTCTTCCGTCAATTCCTTTAAGTTTCAACTTTGCAACCATACTTCCCCCGGAAACT--AG-CTTT-GGTTTCCCGGAAAGCTACTGAATTCACCATAAATA-AGTAGTGAAATCCAATTGCTAGCTGTCATCGTTTACAGTTAGAACTAGGGCGGTATCTAATCGCCTTCGATCCTCTAACTTTCGTTCTTGATTAATGAAAACATCCTTGGCAAATGCTTTCGCTTTAGTTAGTCTTACGACGGTCTAAGAATTTCACCTCTCGCGCCGTAATACTAATGCCCCCGACTGCTTCTATTAATCATTACCTCTGTGATCTG-TATCAAACCAATAGAAAAGCAATA--------------CACACGTAAATGTGCA-TTTA-TAAGACCGAGGTCTTTTTCCATTATTCCATGCAAAAATATTCAAGGCATTTAGAGCCTGCTTTGAGCACCTTAATTTGTTCAAGGTAAAAGTAAGCCGAACTAAATAGACATCTAGCCTAATAAAAGGCATCAATGCTATTCATTTA--GTGTTCAGTCATATAGTCCAAGTAA-ACGAAAATGATAGCATACCA--TACTTGATGGAGCTCC------ACTC-------G-TACTGGACAAAAATCAACTTCGAACGTTTT-AACCGCAACAATTTTAATATACGCTAGTGGAGCTGGAATTACCGCGGCTGCTGGCACCAGACTTGCCCTCCACTTGATCCTCATTAAAGGATTTATACT-TGATTCATTCCAATTACAGAACATAGTTAACTAGTTCTATATTGTTATTTTTCGTCACTACCTCT-----TTCAATATTTGCTTGCGCGCAATGTGCACTTTTCCTTTAGTAAGACGTTGTGATCCATTAGAAATTCATTCCAATGATTATTGGGCTACCAAAAGGCTGGCGTGAAGTTCATGAGTAAATAATTGAATCGCTTATGGTTTGATATATTGCTTCGTGAGCATGTCGTGTCCTG--TTGTAGCTGTCTGATGGATTTTAAAGATTATATTATTGCATTATCCATAATAATCCGATCAACAACGGTTAAATTTGGGATACCTTTTGGACCCGTCTTGAAACACGGACCAAGGAGTCTATCTAGTATGCAAGTAAATGAGGAAACTTATTTGCGTAAAAAACTTAACTGATGGGATTACGAGTGCTCTTCCATCCCGGGGTATTTTCATCAACATATGC-AA-GTATATGGAAAATATACCATGAGCATACTGGATATGACCCGAAAGATGGTGAACTATGCCTGATCAGGTTGAAGTCAGGGGAAACCCTGATGGAGGACCGAAGCAGTTCTGACGTGCAAATCGCTGACGTATCCATTGATCGGCAATTACGGCATTCCCGACGACGAAGAGTTCGACATTGACGGACTGATGAAGAACTTTGAATCGAATAATAAGATTTGGATTACGGCGCTTGTTGTGGGCGAGCTATGCGAAGTTCCATCTCACTGGCGTCAAAAGTACAAACTGTCGGAATGGATGAAGAAGCATAGCATTCCTGGCATCAGTGGAATCGACACTCGTGCTTTGACTAAAAACATCCGAGAAAATGGAACCGTCTTGGGGAAAATCATTCAGCAGCCGTCG------GGACCCTTTGCCGGT---TTGGAATTCAAAGATCAGAACGAGAGGAACCTCGTTGATGAAGTTTCCATCAAAACGCCAGTGACCTACAATCCTTCTGGATCGCCTCGCATTTGCGCCATCGATTGTGGCTTGAAACTCAACCAGATCCGATGCTTTACGAAACGTGGTGCTCGCGTTGACGTCGTTCCGTGGAATTACGAAATCAATCCACGTGACTGTGATGGGCTTTTCTTGAGCAACGGCCCTGGCGATCCCGTTACATGCCAGTCGACAGTAAGGAACATCCAAAACTTCCTAAACAGCGATAACGTCAAACCGGTTTTTGGCATTTGCTTGGGCCATCAACTGCTGGCGACTGCGATTGGCTGCAAAACATACAAAATGAAGTATGGAAATCGAGGACACAACTTGCCTGCTCTCCATCATGGAACTAATCGCTGTTTCATGACGTCACAGAATCATGGATTTGCCGTTGACGTTGCTACAATCAACAAGGACTGGGAAGCGCTATTCACAAACCTGAATGACTTCATTCGCGTCAGTAAGCACATCGGCAGCTCAATGAAGAGTGTCGGCGAGGTGATGGCGATTGGCAGAAAGTTTGAAGAAGCTTTCCAAAAGGCACTGCGAATGGTCGATGAAACTGTCAATGGATTTGATCCCAACCTAAAGCCGGTTAATGATGAAGAGTTGAAAATGCCAACCGACAAGCGAATGTTTGTTCTTGCTGCTGCTCTCAAGGCAGGGTACACGGTGGATCGTCTCTACGACTTGACTAAAATCGATCGTTGGTTCCTCGAAAAAATGAAGAACATCATCACAATTACCACACAACTAGAAGAGCTCAAC---TGCATCATACCCAACGAACTGCTATCCAAAGCCAAGCAGCATGGCTTCTCAGACAAACAAATCGCGACATTCATCAAA--TTCTGAGTTGGCAGTGCGAAAGCAGCGACGCGAATCAAAAATTTTGCCCTTCGTGAAGCAAATCGACACTGTAGCTGGTGAGTGGCCTGCGGCMACTAACTACTTGTACTTGACGTACAACGCCAGTGCACATGACATCGAATTTGAT---GAGGAGATGGTGATGGTCATCGGCTCAGGAGTTTATCGCATAGGCAGTTCAGTGGAATTCGATTGGTGTGCCGTTGGTTGTTTGCGAGAACTGCGCAAGTTGGGTAAGAAGACAGTGATGGTGAANNNCAATCCGGAAACTGTGTCCACCGACTACGATATG--------------------------------CATATTATTAGTCAAGAAAGAGGAAAAAAGGAAACTTTTGGAGCTTTAGGAATAATTTATGCAATATTAGCTATTGGTTTATTAGGATTTGTTGTTTGAGCTCACCACATATTTACTGTCGGAATAGACGTAGATACTCGCGCTTATTTTACATCTGCTACTATAATTATTGCCGTACCTACGGGTATTAAAATTTTTAGATGATTGGCCACTCTTCACGGTACCCAAATTAATTACTCTCCAGCCTTATTATGAGCATTAGGATTTGTTTTTTTATTTACTGTTGGTGGTTTAACCGGGGTGGTTCTCGCTAACTCTTCTATTGATATTGTATTACACGATACTTACTATGTAGTAGCTCATTTTCATTATGTACTTTCAATAGGGGCGGTCTTTGCTATTATGGCAGGATTTGTCCACTGATTCCCTCTTTTTACCGGATTAAATTTAAATGAAAAATGATTAAAATCTCAATTTTTAATAATATTTTTAGGAGTAAATTTAACATTTTTTCCTCAACATTTTTTAGGATTAGCCGGGATGCCTCGTCGTTACTCTGACTACCCCGACGCTTATACTTCATGAAATATTGTATCTACAATCGGATCCACAATCTCTCTTTTAGGAGTTCTATTTTTTCTTTTTATTATTTGAGAAAGT

**Gen. “Australia” sp.** ---------------------------ACT--ACCCTTAATTTC-A-AGAAAACGCTATCAA-GTTGTCATACCCTATTAAGTTCGG-ACCTGGTAAGTTTTCCCGTGTTGAGTCAAATTAAGCCGCAAATTCCAAACC--TGGTGTGAACTTCCGTCAATTCCTTTAAGTTTCAACTTTGCAACCATACTTCCCCCGGAAACT--AG-CTTT-GGTTTCCCGGAAAGCTACTGAATGCACCATGAAA--AGTAGTGACATCCAATTGCTAGCTGTCATCGTTTACAGTTAGAACTAGGGCGGTATCTAATCGCCTTCGATCCTCTAACTTTCGTTCTTGATTAATGAAAACATCCTTGGCAAATGCTTTCGCTTTAGTTAGTCTTGCGACGGTCTAAGAATTTCACCTCTCGCGCCGCAATACTAATGCCCCCAACTGCTTCTATTAATCATTACCTCT-TGATCTG-TATCAAACCAATAGAAAA-CGATA----------------------------------------------------------------------------------------------------------------------------------------------------------------------------CCA---------------CAGTC------------------------------------------------------------ACCC-------A----------------------------------------------------------------------------------------------------------TGAT--------------------------------------------------------------------------------------------TTCAATATTCCTTTAAAAGCAATGTGCACTTCTCCCTTAGTAAGACGCTGTGATCCATTAGAAATTCATTCCAATGATTATTTGGCTATCAATGGGCCGATATGAAGTTCATGAGCAAATAATTCAATCTTATAAGGTTGGGTATATTGTATAATGAGCATATTGTAACCCG-ATTATAGCTGTCTGATGGATTTTAAAGGTCATATTATTGCATCACTCAAAATAACCCGATCAACAGTGTTTAAATTTGGGATACCTTTTGGACCCGTCTTGAAACACGGACCAAGGAGTCTATCTAGTATGCAAGTAAATGAGGAAACTTATTTGCGGAAAAAACTTGACTGATGGGACTACGAGTGCTTCTCCATCCCGGGGTATTTTTATCAACATATGCAAATGTATATGGAAAATATACCATGAGCATACTGGATATGACCCGAAAGATGGTGAACTATGCCTGATCAGGTTGAAGTCAGGGGAAACCCTGATGGAGGACCGAAGCAATTCTGACGTGCAAATCGCTGACTTATCCATTAATTGGAAATTATGGGATTCCGAGTGATGAAGATTTCGATGAGCACAAGATGATGCAGCACTTTGAGTCCAATAATAAAATCTGGGTTTCTGGTCTTGTTGTTGGTGAGTTATGCGAGACACCGTCGCATTGGAGACAGAAATACAAGCTCGCGGAATGGATGAAGAAGCACAATGTCACTGGYATCAGTGGAATCGACACTCGCGCTTTAACTAAACGAATTCGTGAAGACGGAACGGTTCTCGGTAAAATTATTCATCAATCTGCT------GGTCCATTTCCAGGA---TTGGAGTTTAAGGATCAGAACCAGCGTAATCTCGTTGATGAAGTTTCGATTAAAAAGCCAATCACGTACAATTCGAAAGGCTCGCCGACAATTTGTGCCATCGATTGCGGACTGAAATTGAATCAGATTCGTTGCTTTGTTAATCGCGGAGCTCGTGTCGATGTGGTGCCATGGGACCATCCATTGAATCCCGATGCTTTTGATGGACTCTTTCTCAGCAACGGTCCTGGTGATCCCGTCGTGTGCCAAAAGACTGTCAAAAATATTCAAAATTTCCTCGCATCGCCTAAAGTGAAGCCCATTTTTGGGATCTGCTTGGGTCATCAATTGCTCTCAACTGCCATCGGCTGTAAAACTTACAAATTAAAATATGGAAATCGAGGTCACAATCTTCCAGCTCTCCATCATGGAACCAATCGTTGCTTCATGACCTCTCAAAATCACGGATTCGCTGTCGATGTCAAAACAATTGCTAAGGATTGGGAGCCGCTCTTCACCAATCTCAAT---TTCATTAGAGTCAGTAAACATATTGGAAGCTCTATGAAAAGTGTTGGTGAAGTGATGGCAATTGGACGAAAGTTTGAAGAAGCTTTTCAAAAAGCTCTCCGTATGGTTGATGAAAACGTTGATGGATTTAATCCGAACTTGAAGCCAGTGTGTGATGAAGAGCTGATGACACCAACAGATAAAAGAATGTTCGTCCTTGCTGCTGCATTAAAAGCTGGCTATTCAATCGATCGGCTATACGATTTAACGAAAATTGATCGTTGGTTTCTTGCAAAAATGAAAAACATCAACGAAGTCACTTTGGAGCTTGAAAAGCTCAAC---TGCGTAATTACCGATGAGCTTTTGGGTCGAGCTAAGAAGCTCGGATTCTCTGATAAGCAAATTGCAAATTCGATTAAA--ATCGGAGCTTGAAGTGAGAAAACAAAGACGTGAGAGTGGAATTCTGCCATTCGTTAAGCAGATCGACACAGTAGCTGGTGAATGGCCGGCATCGACCAACTACCTGTACCTGACTTACAACGCAAACACTCACGACATTGAATTTAAT---GATCAAATGGTGATGGTGATTGGCTCGGGAGTTTATCGAATTGGTAGTTCTGTTGAATTCGATTGGTGTGCTGTTGGATGTCTGAGGGAGCTGAGAAAGTTGGGAAAGAAGACAATCATGGTGAACTACAATCCAGAAACTGTTTCAACTGACTACGACATTTGCGATCGTTTATACTTTGAAGAAATTTCATTCATATTATTAGTCAAGAAAGAGGAAAAAAGGAAACTTTTGGAGCTTTAGGAATAATTTATGCTATACTTGCTATTGGACTATTAGGATTTATTGTTTGAGCTCATCATATATTTACTGTAGGTATAGATGTAGATACTCGAGCCTATTTTACTTCCGCTACAATAATTATTGCTGTTCCTACAGGAATTAAAATTTTTAGATGACTTGCTACATTACACGGAACTTCTCTAAATAACTCACCTTCTCTTTTATGATCTTTAGGATTTGTATTTTTATTTACTGTTGGAGGATTGACAGGAGTAATTCTTGCTAATTCTTCGATTGATATTGTTCTTCATGATACTTATTATGTTGTAGCTCATTTTCACTATGTTCTTTCTATAGGAGCAGTTTTCGCTATTATAGCAGGATTCGTTCATTGATACCCCTTATTTACTGGACTTTCAATAAATGAAAAATGATTAAAATCTCAATTTACAATTATATTTTTAGGGGTAAATTTAACATTTTTCCCTCAACACTTTTTAGGTCTAGCAGGAATGCCTCGTCGTTATTCTGATTACCCTGATGCCTACACTTCTTGAAATATCGTTTCAACCGTTGGATCAACAATTTCATTATTAGGAATTATCTTTTTCATTTTTATTATTTGAGAAAGA

**Gen. “Australia” sp. 1** ---------------------------ACT--ACCCTTAATTTC-A-AGAAAACGCTATCAA-GTTGTCATACCCTATTAAGTTCGG-ACCTGGTAAGTTTTCCCGTGTTGAGTCAAATTAAGCCGCAAATTCCAAACC--TGGTGTGAACTTCCGTCAATTCCTTTAAGTTTCAACTTTGCAACCATACTTCCCCCGGAAACT--AG-CTTT-GGTTTCCCGGAAAGCTACTGAATGCACCATGAAA--AGTAGTGACATCCAATTGCTAGCTGTCATCGTTTACAGTTAGAACTAGGGCGGTATCTAATCGCCTTCGATCCTCTAACTTTCGTTCTTGATTAATGAAAACATCCTTGGCAAATGCTTTCGCTTTAGTTAGTCTTGCGACGGTCTAAGAATTTCACCTCTCGCGCCGCAATACTAATGCCCCCAACTGCTTCTATTAATCATTACCTCT-TGATCTG-TATCAAACCAATAGAAAA-CGATA----------------------------------------------------------------------------------------------------------------------------------------------------------------------------CCA---------------CAGTC------------------------------------------------------------ACCC-------A----------------------------------------------------------------------------------------------------------TGAT--------------------------------------------------------------------------------------------TTCAATATTCCTTTAAAAGCAATGTGCACTTCTCCCTTAGTAAGACGCTGTGATCCATTAGAAATTCATTCCAATGATTATTTGGCTATCAATGGGCCGATATGAAGTTCATGAGCAAATAATTCAATCTTATAAGGTTGGGTATATTGTATAATGAGCATATTGTAACCCG-ATTATAGCTGTCTGATGGATTTTAAAGGTCATATTATTGCATCACTCAAAATAACCCGATCAACAGTGTTTAAATTTGGGATACCTTTTGGACCCGTCTTGAAACACGGACCAAGGAGTCTATCTAGTATGCAAGTAAATGAGGAAACTTATTTGCGGAAAAAACTTGACTGATGGGACTACGAGTGCTTCTCCATCCCGGGGTATTTTTATCAACATATGCAAATGTATATGGAAAATATACCATGAGCATACTGGATATGACCCGAAAGATGGTGAACTATGCCTGATCAGGTTGAAGTCAGGGGAAACCCTGATGGAGGACCGAAGCAATTCTGACGTGCAAATCGCTGACTTATCCATTAATTGGAAATTATGGGATTCCGAGTGATGAAGATTTCGATGAGCACAAGATGATGCAGCACTTTGAGTCCAATAATAAAATCTGGGTTTCCGGTCTTGTTGTTGGTGAGTTATGCGAGACACCGTCGCATTGGAGACAGAAATACAAGCTCGCGGAATGGATGAAGAAGCACAATGTCACTGGCATCAGTGGAATCGACACTCGCGCTTTGACTAAACGAATTCGTGAAGACGGAACGGTTCTTGGTAAAATTATTCATCAATCTGCT------GGTCCATTTCCAGGA---TTGGAGTTTAAGGATCAAAACCAGCGTAATCTCGTTGATGAAGTTTCAATTAAAAAGCCAATCACGTACAATTCGAAAGGCTCGCCGACAATTTGTGCCATCGATTGCGGACTGAAATTGAATCAGATTCGTTGCTTTGTCAATCGCGGAGCTCGTGTCGATGTGGTGCCATGGGATCATCCATTGAATCCCGATGCTTTTGATGGACTCTTTCTCAGCAACGGTCCTGGTGATCCCGTCGTGTGCCAAAAGACTGTCAAAAATATTCAAAATTTCCTCGCATCGCCTAAAGTGAAACCCATTTTTGGGATCTGCTTGGGTCATCAATTGCTCTCAACTGCCATCGGCTGTAAAACTTACAAATTAAAATATGGAAATCGAGGTCACAATCTTCCAGCTCTCCATCATGGAACCAATCGTTGCTTCATGACCTCTCAAAATCACGGATTCGCTGTTGATGTCAAAACAATTGCTAAGGATTGGGAGCCGCTCTTC---------------TTCATTAGAGTCAGTAAACATATTGGAAGCTCTATGAARAGTGTTGGTGAAGTGATGGCAATTGGACGAAAGTTTGAAGAAGCTTTTCAAAAAGCTCTCCGTATGGTTGATGAAAACGTTGATGGATTTAATCCGAACTTGAAGCCAGTGTGTGATGAAGAGCTGATGACACCAACAGATAAAAGAATGTTCGTCCTTGCTGCTGCATTAAAAGCTGGCTATTCAATCGATCGGCTATACGATTTAACGAAAATTGATCGYTGGTTTCTTGCRAAAATGAAAAACATCAACGAAGTCACTTTGGAGCTTGAAAAGCTCAAC---TGCGTAATTACCGATGAGCTTTTGGGTCGAGCTAAGAAGCTCGGATTCTCTGATAAGCAAATTGCAAATTCGATTAAA--ATCGGAGCTTGAAGTGAGAAARCAAAGACGTGAGAGTGGAATTCTGCCATTCGTTAAACAGATCGACACAGTAGCTGGTGAATGGCCGGCATCGACCAACTACCTGTACCTGACTTACAACGCAAACACTCACGACATTGAATTTAAT---GATCAAATGGTGATGGTGATTGGCTCGGGAGTTTATCGAATTGGTAGTTCTGTTGAATTCGATTGGTGTGCTGTTGGATGTCTGAGGGAGCTGAGAAAGTTGGGAAAGAAGACAATCATGGTGAACTACAATCCAGAAACTGTTTCAACTGACTACGACATGTGCGATCGTTTATACTTTGAAGAAATTTCATTCATATTATTAGTCAAGAAAGAGGAAAAAAGGAAACTTTTGGAGCTTTAGGAATAATTTATGCTATACTTGCTATTGGACTATTAGGATTTATTGTTTGAGCTCATCATATATTTACTGTAGGTATAGATGTAGACACTCGAGCCTATTTTACTTCAGCTACAATAATTATTGCTGTTCCTACAGGAATTAAAATTTTTAGATGACTTGCTACATTACACGGAACTTCTCTAAATAACTCACCCTCTCTATTATGATCTTTAGGATTTGTATTTTTATTTACGGTTGGAGGATTAACAGGGGTAATTCTTGCTAATTCATCAATTGATATTGTTCTTCATGATACTTATTATGTTGTTGCTCACTTTCACTATGTTCTTTCAATAGGAGCAGTTTTCGCTATTATGGCAGGATTCGTTCATTGATACCCTTTATTTACTGGACTTTCAATAAATGAAAAATGATTAAAATCTCAATTTACAATTATATTTTTAGGAGTAAATTTAACATTTTTCCCTCAACACTTTTTAGGTCTAGCAGGAATACCTCGTCGTTACTCTGATTACCCTGATGCCTACACTTCTTGAAATATCGTTTCAACTGTTGGATCAACAATTTCATTATTAGGAATTATCTTTTTCATTTTTATTATTTGAGAAAGA

**Gen. n. nr. Nanocladius** --------------------------CACT--ACCCTTTTTTTC-G-TGAAAGCGCTGTCAA-GCTGTCACACACAAAAAAGTTCTG-ACCTGGTAAGTTTTCCCGTGTTGAGTCAAATTAAGCCGCAAATTCCAAATC--CGGTATGAACTTCCGTCAATTCCTTTAAGTTTCAACTTTGCAACCATACTTCCCCCGGAAACT--AG-CTTT-GGTTTCCCGGAAAGCTACTGAATGCACCATGAAA--AGTAGTGACATCCAATTGCTAGCTGTCATCGTTTACAGTTAGAACTAGGGCGGTATCTAATCGCCTTCGATCCTCTAACTTTCGTTCTTGATTAATGAAAACATCCTTGGCAAATGCTTTCGCTTTAGTTAGTCTTGCGACGGTCTAAGAATTTCACCTCTCGCGCCGCAATACTAATGCCCCCAACTGCTTCTATTAATCATTACCTCT-TGATCTG-TATCAAACCAATA------------------------------------------------------------------------------------------------------------------------------------------------------------------------------------------------------------------------------------------------------------------------------------------------------------------------------------------------------------------------------------------------------------------------------------------------------------------------------------------------TTCAATATTTATGCAAATACAATGTGCACTTCTCATTTAGTAGGACGCTGTGATCCATTAGAAATTCATTGC---GGTTATTTGGCTATCAAAAGGCCGACATAAAGTTCATTGGCAAACAACTCGATCCTTTATGGTTGGGTATATTGCTTGATGAGTTTGTCGTGTCCTGT-CTATAGCTGTCTGATGGATTTTAAAGGTCATAACATTGTATCACTCAGGTTAACCCGATCAACAGCGGTTAAATTTGGGATACCTTTTGGACCCGTCTTGAAACACGGACCAAGGAGTCTATCTAGTATGCGAGTAAATGAGTAAACTTATTTGCATAAAAAACTTGACTGATGGGATTACGAGTGCTCCTCCATCCCGGGGTATTTTCATCAACATATGCAAATGTATATGGAAAATATACCATGAGCATACTGGATATGACCCGAAAGATGGTGAACTATGCTTGATCAGGTTGAAGTCAGGGGAAACCCTGATGGAGGACCGAAGCAATTCTGACGTGCAAATCGCTGACGTATCCACTCATTGGCAACTACGGAATTCCGAGTGACGAAGAGTTCGACGAGTTCAAGTTGATGAAGAATTTCGAGTCAACCGGTAAGATTTGGGTGTCAGGACTGGTTGTTGGTGAGTTGTGTGAGACTCCGTCGCACTGGAGGCAAAAGTATAAACTGGCTGAGTGGATGAAGAAGCACAATGTAGTTGGAATTAGTGGAATTGACACTCGTGCATTGACTAAGAAGATCCGAGAGGATGGAACGGTGCTTGGAAAAATAGTCCAACAATCTCAA------GGACCATTTCCGGGT---CTCGAGTTCACTGATCAAAATTTGAGGAATCTCGTGGCAGAAGTTTCTATCAAGAAACCGATTACTTACAACCCGAAGGGAACTCCGAGGATCTGTGCTGTCGACTGCGGCTTGAAACTCAATCAAATTCGATGCTTTCTCAAACGTGGAGCTCGAGTTGATCTCGTGCCATGGAACCATCCATTAAATCCCAATGATTTTGATGGTCTTTTCCTGTCCAACGGTCCCGGTGATCCTGTCGTGTGCCAAGAAACTGTCAAAAACATTCAAAAAGTCATTTCAGCTCCTCAAGTCAAACCAATTTTTGGTATTTGTCTTGGTCATCAACTTTTGTCGACGGCAGTTGGATGCACAACTTACAAGCTGAAATATGGAAATCGAGGTCACAATTTACCGGCAATTCATCATGGTACCAATCGCTGCTTCATGACATCCCAGAATCATGGATTTGCTGTTGATGCC------------------------------------------------------------------------------------------------------------ATTGGACGGAAGTTTGAAGAAGCTTTTCAGAAAGCTTTGCGTATGGTTGACGAGAATGTCAATGGATTTGATCCCAACTTAAAGCCAGTGTGTGATGAAGAATTGAAACAGCCAACTGACAAGAGAATGTTTGTACTGGCAGCTGCTTTGAAAGCTGGTTATACCATCGACAGGCTGTATGATCTTACAAAAATCGACCGTTGGTTCTTAGAAAAGATGAAAAACATCATTACAACTACAATTGAACTTGAAGCCTTGAAT---TGTGTCATTGACAATGACCATCTGAGACGTGCCAAGTGTTTGGGTTTCTCTGACAAGCAGATTGCGGCTTGTATTAAG--ATCTGAACTTGCAGTTCGCAAACAACGACGTGAAGCTGGAATTTTACCGATTGTCAAGCAAATTGATACTGTTGCAGGTGAATGGCCGGCTTCGACCAATTACTTGTATCTCACCTACAATGCCATCGAACATGATGTAGAATTCGAC---GAACAGATGGTCATGGTGATTGGATCTGGAGTTTATAGAATTGGAA----------------------------------------------------------------------------------------------------------------------------------------------------CATATTATTAGACAAGAAAGAGGAAAAAAGGAAACTTTTGGTGCATTAGGAATAATTTATGCTATATTAGCTATTGGTCTATTAGGGTTTGTAGTATGAGCTCATCATATATTTACTGTAGGAATAGATGTAGATACTCGTGCATATTTTACTTCAGCTACAATAATTATTGCTGTTCCTACAGGAATTAAAATTTTTAGCTGACTAGCTACTCTTCACGGAACTCAAATAAATAACTCTCCATCTTTATTATGAGCTTTAGGATTTGTATTTTTATTTACAGTAGGAGGATTAACAGGAGTTGTATTAGCAAATTCTTCTATTGATATTGTTTTACATGATACTTATTATGTTGTAGCCCACTTTCATTATGTATTATCTATAGGAGCAGTATTTGCTATTATAGCTGGATTTGTTCACTGATACTCTCTATTTACAGGACTATCTATAAATGAACAATGATTAAAATCACAATTTTTAATTATATTTGTTGGAGTAAATTTAACATTCTTTCCACAACATTTCTTAGGATTAGCAGGTATGCCTCGACGATACTCTGATTATCCAGATGCTTATACAACATGAAATATTGTATCAACAGTTGGTTCAACAATTTCCTTATTTGGGATTTTATTTTTTTTATTTATTATTTGAGAAAGT
