# Supplementary material for: Blind Flight? A New Troglobiotic Orthoclad (Diptera, Chironomidae) from the Lukina Jama – Trojama Cave in Croatia
Source: PLoS One. 2016 Apr 27;11(4):e0152884. doi: 10.1371/journal.pone.0152884 (PMC4847865; doi:10.1371/journal.pone.0152884)
Supplement: S3 Text — (DOCX) [file pone.0152884.s005.docx]

**S5 text – Reference sequence with secondary structure annotation for the alignment of 18s rDNA.**

**Corynoneura sp. 1**

--------------------------------------------------AGAACGGCCA

TGCACCACT--ACCCTTAATTTC-A-TGAAAGCGCTATCAA-GCTGTCAAACCCTATTAA

GTTCTG-ACCTGGTAAGTTGTCCCGTGTTGAGTCAAATTAAGCCGCAACATCCACTGC-C

GGTGATGATCTTCCGTCAATTCCTTTAAGTTTCAACTTTGCAACCATACTTCCCCCGGAA

ACT--AG-CTTT-GGTTTCCCGAAGAGCTACTGAATGCACCATGAAA--AGTAGTGACAT

CCAATCGCTAGCTGTCATCGTTTACAGTTAGAACTAGGGCGGTATCTAATCGCCTTCGAT

CCTCTAACTTTCGTTCTTGATTAATGAAAACATCCTTGGCAAATGCTTTCGCTTTAGTTA

GTCTTGCAACGGTCTAAGAATTTCACCTCTCGCGCTGCAATACTAATGCCCCCAACTGCT

TCTATTAATCATTACCTCT-TGATCTGGTATCAAACCAACAGAAAATCGACA--------

------ACATTTTGCAACATTG-CCGAATAAGACCGAGGTCTTTTTCCATTATTCCATGC

AAAAATATTCAAGGCATA-AGAGCCTGCTTTGAGCACCTTAATTTGTTCAAGGTAAAATT

AAGCCGAACTAAATAGACACTTAGCCTAATGAAAGGCATCGGTGCTATTCATTTA--GTG

TTCAGTCAAATAGTTCAAGTAA-TCGGAAATGATGGTA-GTCATGTACTTTATGACACCA

C------ACCC-------G-TACTGAACAATAATCAACTTCGAACGTTTT-AAACGCAAC

AATTTTAATATACGCTAGTGGAGCTGGAATTACCGCGGCTGCTGGCACCAGACTTGCCCT

CCACTTGATCCTCAACAAAGGATTTATACT-TGATTCATTCCAATTACAGAACATAGTTA

ACTAGTTCTATATTGTTATTTTTCGTCACTACCTCC-

**Secondary structure annotation**

xxxxxxxx39'xxxxxx41'xxxxxx{41}x{40}xxxx{40}xx39-x{-------38-

--------}--xxx{---------3-7'----}xxxxxxxx-{-----------37----

-}xxxx{-36--}xxxxxxxx-35'xxxxxxxx{35}xxxx{-----34---}{33-}-x

xxxxx{---32------}xx{-2}xxx{--31'---}xxxx{--31---}xx{-22'}x{

--28'---}xxxx30xxx30xxxx{-----29'---------}xxxx{---------29-

--------}x{-28-}xxxxxxxxxx{-------27'---}xxxxxxxxx{----27---

-------}xxxx{----23'-}{-------24'-------}xxx26xxxx26xxxxxxx{

----25'------------}xxxxxx{-------25-------}xx{--------24---

-----}xxxxxxxx{-E23--14'}x---------------xxxxxx{------------

--E23-13--------------------}x{-E23-14}xxxx{-E233--}xx{-E23-

7'-----------}xxxx-xx-{--E23-7--}x{E23-6'}xxxxx{E23-6-}-----

-----------------------------------------------E23-4----E23-

5--E23-1----------------------------------------------------

--------------------------------------23----xxxxxx-x{-22-}xx

xxxxx{---3'---}x{19'-}{21}xxxxxxxxx20'xxxxxxxx{21}xxxx{20{-1

9-}xxxxxxxxx18xxxxxxxxxxxxxxxxxx18xxxxxx{--17'--------}xxx{-

-----17------}xxxxxxxx{4'}xx{--16'--}
